# Properties and Distribution of Pure GA-Sequences of Mammalian Genomes

**DOI:** 10.1371/journal.pone.0003818

**Published:** 2008-11-27

**Authors:** Guenter Albrecht-Buehler

**Affiliations:** Department of Cell and Molecular Biology, Feinberg School of Medicine, Northwestern University, Chicago, Illinois, United States of America; Pasteur Institute, France

## Abstract

The article describes DNA sequences of mammalian genomes that are longer than 50 bases, but consist exclusively of G's and A's (‘pure GA-sequences’). Although their frequency of incidence should be 10^−16^ or smaller, the chromosomes of human, chimpanzee, dog, cat, rat, and mouse contained many tens of thousands of them ubiquitously located along the chromosomes with a species-dependent density, reaching sizes of up to 1300 [b]. With the exception of a small number of poly-A-, poly-G-, poly-GA-, and poly-GAAA-sequences (combined <0.5%), all pure GA-sequences of the mammals tested were unique individuals, contained several repeated short GA-containing motifs, and shared a common hexa-nucleotide spectrum. At most 2% of the human GA-sequences were transcribed into mRNAs; all others were not coding for proteins. Although this could have made them less subject to natural selection, they contained 160 times fewer point mutations than one should expect from the genome at large. As to the presence of other sequences with similarly restricted base contents, there were approximately as many pure TC-sequences as pure GA-sequences, but many fewer pure AC-, TA, and TG-sequences. There were practically no pure GC-sequences. The functions of pure GA-sequences are not known. Supported by a number of observations related to heat shock phenomena, the article speculates that they serve as genomic sign posts which may help guide polymerases and transcription factors to their proper targets, and/or as spatial linkers that help generate the 3-dimensional organization of chromatin.

## Introduction

### Genome navigation as a requirement for gene regulation

Obviously, cells cannot regulate the expression of a gene unless they can find it. Therefore, genome navigation is a necessary, albeit still little understood, early step of gene regulation. Especially the often huge mammalian genomes may require a sophisticated system of genome navigation to enable polymerases, transcription factors and other DNA binding proteins to zoom quickly into their proper target areas without having to search base-by-base along sequences of many billion bases.

Many facets of the mechanisms of genome navigation are presumably already known. For example, the distribution and binding of transcription factors along the genome and the pausing of polymerases [1] will undoubtedly play important roles. On the other hand, it is not fully understood what mechanism are able to transport these components to their respective target sites. The often tacit assumption that they travel along the genome by molecular diffusion is doubtful, as molecules that have to diffuse randomly throughout the large and dense chromatin matrix may not provide a sufficiently fast and accurate genomic search mechanism. This objection applies especially to the cases of stress responses such as heat shock, where a cell needs to mount a very rapid multi-component response.

In order to learn some of the most basic requirements for the navigation of large genomes, one may look to our own information technology which, similar to genomes, operates under the constraints of relatively limited storage space and the need for short access times. In this case one of the most basic requirements is the dense distribution of formatting markers. They are needed to tell the mechanisms of data-storage and data-retrieval at every moment their present locations in memory during their search for target loci.

I submit that large genomes may require the presence of similar sign posts. If this is correct, and they can be found in mammalian genomes, they would provide an important starting point for the search for the mechanisms of genome navigation.

### Pure GA-sequences as candidates for genomic sign posts

A simple way to search for natural genomic sign posts would be to look for unexpected, non-coding sequences that exist in large numbers. Here, we describe a specific type, namely sequences between 50 and 1000 bases long that consist exclusively of only 2 bases.

Indeed, such sequences would be quite unexpected. Crudely estimated, the probability p that a sequence of 50 bases contained only (say) A's and G's would be p = (1/2)^50^ = 0.000,000,000,000,000,09. In other words, such sequences should never be found.

Nevertheless, as will be reported here, the chromosomes of human, chimpanzee, dog, cat, rat, and mouse contain many tens of thousands of sequences similar to the sequence of Example 1. They will be called ‘pure GA-sequences’. The article describes their frequency, distribution, composition from smaller motifs, and apparent protection from point mutation, and speculates about their potential functions as genomic sign posts of genomes and spatial linkers of chromatin. In addition, it discusses several results from the field of heat shock biology that could be viewed as experimental support for this interpretation.

## Results

### The phenomenon of pure GA-sequences

**Figure 1:**
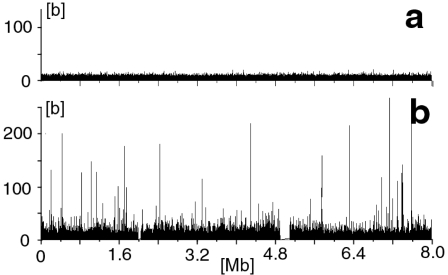
Length of pure GA-sequences as a function of their starting position along an 8 [Mb] long stretch of DNA sequence. (Abscissa: position in [Mb]; Ordinate: length of pure GA-sequence in [b]. a. Computer generated random sequence of 20% G's and 30% A's (similar to the human genome). b. Human chr. 1 between positions 24 [Mb] and 32 [Mb]. (Gaps in the density of GA-sequences are due to un-sequenced regions).

**Figure 2:**
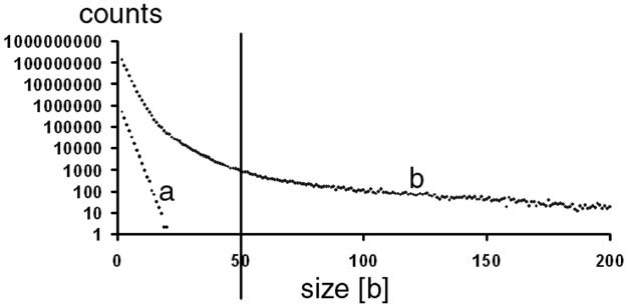
Distribution of GA-sequence lengths. Abscissa: Ga-sequence length [b]. Ordinate logarithm of counts. The vertical line indicates the defining threshold of 50 [b] for pure GA-sequences. a. Exponential distribution of the computer-generated GA-sequence of Fig. 1a. b. The Pareto-distribution of GA-sequence lengths of the entire human genome.

Plotting the sizes of all pure GA-sequences as a function of their position along a random, computer-generated DNA strand, showed that such sequences are hardly ever longer than 15 bases (Fig. 1a, Fig. 2 (graph a)).

In stark contrast, a similar plot of an 8 Mb large section of human chr. 1 between 24 Mb and 32 Mb, displayed numerous much longer sequences, including 76 which were between 50 and 269 bases long (Fig. 1b).

### The complementary pure TC-sequences

Every pure GA-sequence was naturally paired with a pure TC-sequence on the opposite strand. Likewise, each pure TC-sequence on a strand corresponds to a pure GA-sequence on the complementary strand. In view of the actions of countless inversions in the evolutionary past of each chromosome, one may expect that there are approximately as many pure GA-sequences as there are pure TC-sequences on each chromosome. After all, each inversion that contained a GA-sequence simply exchanged it with its reverse complementary TC-sequence from the opposite strand, and vice versa, thus asymptotically equalized their numbers on each strand [see 2]. Indeed, human chr. 1, which contained 1667 pure GA-sequences also contained 1734 pure TC-sequences. The corresponding numbers for human chr3 were 1155 and 1118, and for the human X chromosome 1115 and 1059.

### The size-distribution of pure GA-sequences

The probability that several A's and G's occur N times in a row should be a rapidly diminishing exponential function of N. Indeed, a logarithmic plot of the frequencies of the lengths of the pure GA-sequences of a computer-generated, random DNA sequence yielded a straight line (Fig. 2).

In contrast, a logarithmic plot of the frequency of the actual probability of the size of pure GA-sequences yielded a power law distribution. Figure 2 shows the example of the human genome. After normalization for the values of size = 1 to ∞ by dividing each raw count by the sum of all raw counts the probability density function became
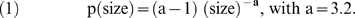
The exponent a = 3.2 was determined by a double logarithmic plot of the normalized function. Equation (1) fitted with high accuracy especially the values >50. Thus, it seemed that the sizes of GA-sequences follow a Pareto-distribution [5]. Initially defined by the Italian economist Vilfredo Pareto to describe the distributions of income and wealth, a great many other real-world phenomena such as the size distributions of sand or meteorites, the sizes of human settlements and many others were found to follow this distribution. Compared to exponential distributions including Poisson-distributions for small probabilities, it is characterized by a much slower reduction of frequencies at large size values.

### The exclusion of poly-A and poly-G sequences

Obviously, one may consider poly-A and poly-G sequences as special cases of pure GA-sequences that contain only one of the 2 bases. As these are well-described in the literature, the present article intended to exclude them while focusing specifically on pure GA-sequences that contain both nucleotides. As it turned out, this could be accomplished quite simply by setting a length threshold, because poly-A and poly-G sequences were very rarely longer than 50 bases.

For example, among the mentioned 76 pure GA-sequences in the 8 Mb large section of human chr. 1 between 24 Mb and 32 Mb there were no poly-A or poly-G sequences that were larger than 50 bases. Even within the entire 240 Mb large human chr. 1, which contained 1667 such GA-sequences (see Appendix S1), there were no poly-G sequences and only 7 poly-A sequences longer than 50 bases. Therefore, in the following,

1. A DNA sequence is defined as a ***pure GA-sequence***, if it consists exclusively of G's and A's and is longer than 50 bases.

It should be noted, that this definition reduces drastically the number of poly-A and poly-G sequences among the pure GA-sequences, but it does not eliminate them completely.

### Range of lengths

By the definition [2], the minimum length of pure GA-sequences was 51 [b]. The average length in the human genome was found to be 93 [b], but pure GA-sequences could be much larger. For example, the sizes of the 3 longest pure GA-sequence found in the human genome were 1305 [b] (chr. 7; position 717,235), 869 [b] (chr. 5; position 12,025,708), and 734 [b] (chr. 2; position 196,277).

The 1305 [b] long pure GA-sequence at position 717,235 in chr. 7 is listed here as an example:

### Example 1

AGGGAAAGGGAAAGGGAAAGGAGAAGGAGAAGGAGAAGGAGAAGGAGAAGGGAGAAAGAGAAGGAGAAGGAGAAGGGAGAAGGGAGAAGGGAAAGGAGAAGGAGAAGGGAAAGGAGAAGGAGAAGGGAGAAGGGAGAAGGGAGAAGGGAGAAGGGAGAAGGGAGAAGGGAGAAGGGAGAAGGGAGAAGGAGAAGGAGAAGGAGAAGGAGAAGGAGAAGGAGAAGGAGAAGGAGAAGGAGAAGGGAAAGGAGAAGGGAAAGGAGAAGGGAAAGGAGAAGGAGAAGGAGAAGGGAAAGGAGAAGGAGAAGGAGAAGGAGAAGGAGAAGGAGAAGGGAAAGGAGAAGGGAAAGGAGAAGGGAGAAGGGAGAAGGGAGAAGGAGAAGGAGAAGGAGAAGGAGAAGGAGAAGGAGAAGGAGAAGGGAGAAGGGAGAAGGGAGAAGGGAGAAGGGAGAAGGGAGAAGGGAGAAGGGAGAAGGGAGAAGGAGAAGGAGAAGGAGAAGGAGAAGGGAAAGGAGAAGGGAGAAGGAGAAGGGAGAAGGAGAAGGGAGAAGGAGAAGGGAGAAGGAGAAGGGAAAGGAGAAGGAGAAGGAGAAGGAGAAGGGAAAGGAGAAGGAGAAGGGAAAGGAGAAGGAGAAGGAGAAGGGAGAAGGAGAAGGGAAAGGAGAAGGGAGAAGGAGAAGGGAGAAGGAGAAGGGAGAAGGAGAAGGGAGAAGGAGAAGGGAAAGGAGAAGGAGAAGGAGAAGGGAAAGGAGAAGGAGAAGGAGAAGGAGAAGGAGAAGGGAAAGGAGAAGGGAGAAGGAGAAGGGAGAAGGAGAAGGGAGAAGGAGAAGGGAGAAGGAGAAGGGAAAGGAGAAGGAGAAGGAGAAGGAGAAGGGAAAGGAGAAGGAGAAGGGAAAGGAGAAGGGAGAAAGAGAAGGAGAAGGAGAAGGAGAAGGAGAAGGGAAAGGAGAAGGGAGAAGGGAAAGGAGAAGGAGAAGGAGAAGGAGAAGGGAAAGGAGAAGGAGAAGGAGAAGGAGAAGGGAAAGGAGAAGGGAGAAGGAGAAGGAGAAGGGAAAGGAGAAGGAGAAGGGAGAAGGGAGAAGGGAAAGGAGAAGGGAAAGGAGAAGGAGAAGGAGAAGGGAGAAGGGAGAAGGGAAAGGAGAAGGAGAAGGAGAAGGGAAAGGAGAAGGGGG

The sequence appears to be a chain of a few repeated motifs, such as AGAAGG (154 times) and GAAAGG (31 times). Together, the 2 motifs cover 85% of the sequence. As will be described further below, all pure GA-sequences of mammals seemed to contain in a similar way chains of short, repeated motifs.

### Relationship between chromosome size and numbers of pure GA-sequences

**Figure 3:**
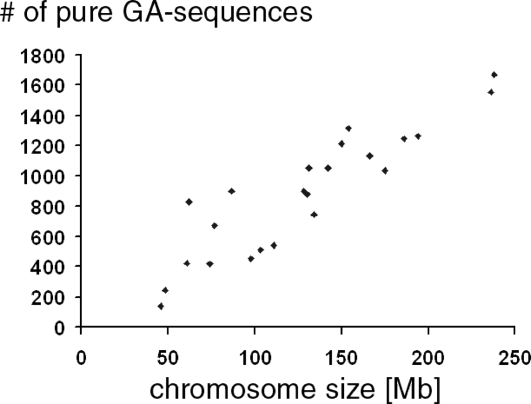
Relationship between chromosome size and number of pure GA-sequences among the 23 human chromosomes (Y-chromosome was omitted).

The numbers of pure GA-sequences were approximately proportional to the size of the chromosome to which they belonged. A correlation plot between the sizes of each human chromosome and its number of pure GA-sequences yielded a correlation coefficient of 0.907 (Fig. 3). Its slope corresponded to an average density of GA-sequences of 6.9 [sequences/[Mb].

### Base composition

The G- and A-composition of the pure GA-sequences was not necessarily the same as the G- and A-composition of the whole genome. For example, the pure GA-sequences of human Chr. 1 contained 91,001 A's and 62,215 G's, corresponding to a ratio of A/G = 1.46. In contrast, the entire chromosome contained 31,037,602 A's and 25,588,336 G's, yielding a ratio A/G = 1.213. It may suggest that pure GA-sequences were subject to different evolutionary and selective mechanisms than the genome at large.

Obviously, each pure GA-sequence must terminate in either a T or a C. In the case of human Chr. 1, 889 pure GA-sequences terminated in a T and 778 in a C. Again, the ratio of terminators T/C = 1.14 was different from the overall genomic ratio T/C = 1.214 of the entire chromosome. (Note that the ratios A/G = 1.213 and T/C = 1.214 are the same for the entire chromosome, as it complies accurately with Chargaff's second parity rule [2]).

### Spatial density and total fraction of pure GA-sequences

As was suggested by Fig. 1b, pure GA-sequences were present in every region of the chromosomes. Even though the lengths of GA-sequences followed a Pareto-distribution [5], for the most part the distances between consecutive GA-sequences appeared to be distributed exponentially. For example, in the case of human chr. 3, 26% of all distances fell into the interval between 0 and 20 [Kb]. The remaining 74% of the distances that were larger than 20 [Kb] could be fitted quite well to the exponential function
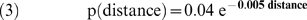



In these cases ‘distance’ was measured as the number of intervening bases in [Kb]. Bases on the actual data and not on any fitting curves, the average distance was 145 [Kb]. Other human chromosomes yielded similar data.

It should be noted that pure GA-sequences represented only a small fraction of every chromosome, in spite of their abundance. For example, the total sequence length of the mentioned 1667 pure GA-sequences of human chr. 1 amounted to as little as 0.0642 [%] of this chromosome.

### The species-dependent spatial density of pure GA-sequences

**Figure 4:**
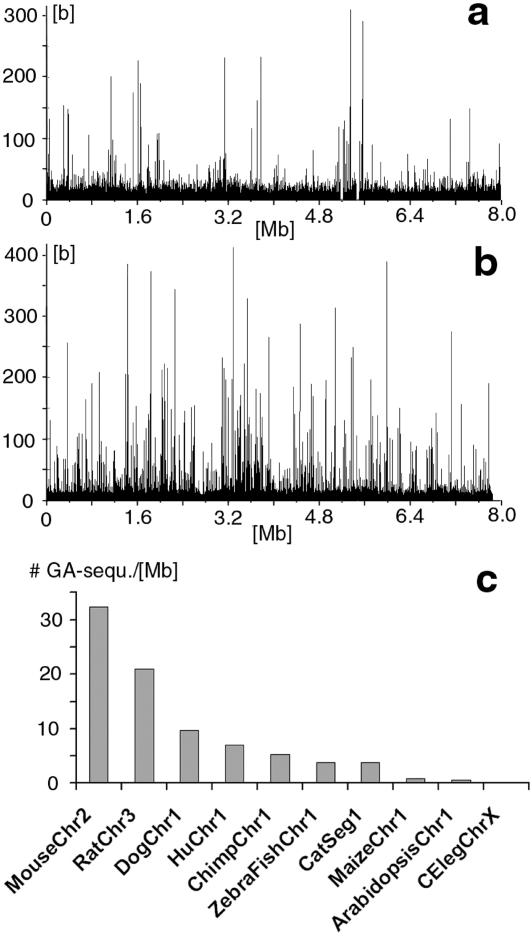
Density of pure GA-sequences for various mammals and other organisms. a. Human chr. 1 between positions 8 [Mb] and 16 [Mb]. (axes as in Fig. 1). b. Mouse chr. 2 between positions 16 [Mb] and 24 [Mb]. (axes as in Fig. 1). c. Density of pure GA-sequences for individual chromosomes of various mammalian and non-mammalian chromosomes (Ordinate: number of pure GA-sequences per [Mb] sequence.). The tags along the abscissa indicate the various chromosomes in the order from left to right: mouse chr. 1, rat chr. 1, dog chr1, human chr. 1, chimpanzee chr. 1, zebrafish chr. 1, cat genome segment 1, maize chr. 1, Arabidopsis chr. 1, and caenorhabditis elegans chr. X.

The densities of pure GA-sequences from chromosomes of different species differed to a much greater degree than the densities of different chromosomes from the same species. For example, the average density of the 23 human chromosomes (excluding the Y-chromosome) was 6.9 [sequences/Mb] (std.dev. = 2.1 [sequences/Mb]). In contrast, the density of mouse chr. 2 and rat chr. 3 were 3 and 4.7 times larger (Fig. 4a,b). On the other hand, maize, arabidopsis and C.elegans had 10 to 50 times smaller densities of pure GA-sequences than human chr. 1. (Fig. 4c). Thus densities could vary up to 250-fold between species. It should be noted that maize, arabidopsis, and C.elegans did not only contain very few pure GA-sequences, they were effectively simply poly-GA sequences.

### The individuality of pure GA-sequences

There are 2^93^≈10^28^ different ways to generate different pure GA-sequences that are on average 93 bases long. This astronomically large number would be able to afford each pure GA-sequence its own, individual sequence.

**Figure 5:**
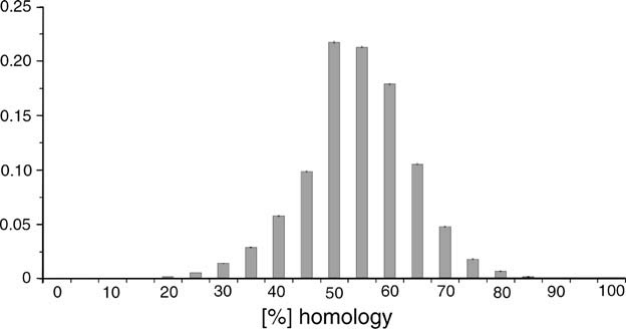
Histogram of the homologies between all pairs of the pure GA-sequences on human chromosomes 1, 2, 3, 7, 17, and X (34,123,491 tests) using the Needleman-Wunsch algorithm [3)).

In order to test whether each pure GA-sequence was in fact an individual I measured the degree of homology between every pure GA-sequence and every other that was found in human chromosomes 1, 2, 3, 7, 17 and X. The tests used the Needleman-Wunsch algorithm [3]. The resulting frequency distribution of a total of 34,123,491 individual tests is shown in Figure 5.

On average there were only 0.5% (stddev: 0.4%) cases of identity among the pure GA-sequences within the same chromosomes, and 0.2% (stddev: 0.1%) cases between different chromosomes of the same species. Most other pairs of pure GA-sequences were approximately 50% homologous, as one would expect statistically from sequences of only 2 bases.

Similarly, I tested for the homologies between pure GA-sequences of chromosomes of human, chimp, dog, mouse, rat, and cat (97,151,955 tests). In this case there were on average 0.5% (stddev: 0.4%) cases of identical pure GA-sequences among the chromosomes of different mammalian species.

The rare cases of 100% homology belonged uniformly to 4 special types of pure GA-sequences, namely poly-A, poly-G, poly-GA, or poly-GAAA sequences. All other pure GA-sequences were unique individuals. Please note that the mentioned poly-A and poly-G sequences were left among the pure GA-sequences because the size restriction for pure GA-sequences of 50 bases or longer, which was used in the definition [2], eliminated most but not all of them.

### The common mammalian frequency spectrum of hexamers

As mentioned earlier, most pure GA-sequences contained several short, repeating motifs (e.g. Example 1). This finding could be trivial, if the motifs in question were only 2, 3, or 4 bases long, because there are only 2^k^ different motifs of size k and, if k<5, they are very likely to occur multiple times in well-mixed sequences of G's and A's longer than 50 bases.

In contrast, the probability is very low that motifs, 6 bases or longer, are repeated tens of times, as in the case of Example 1. In this case, even the probability of only a 3 fold repetition is less than 0.2%. Nevertheless, testing several human chromosomes, I found that many of their pure GA-sequences contained 6 and more repeated motifs of hexa-nucleotides.

In order to test whether there were preferential motifs among them, I determined the hexa-nucleotide distributions for various species and chromosomes and normalized them by dividing each raw count by the sum of all raw counts. Like in earlier studies [2], [4], I used ‘running’ distributions (i.e. frame shift = 1).

**Figure 6:**
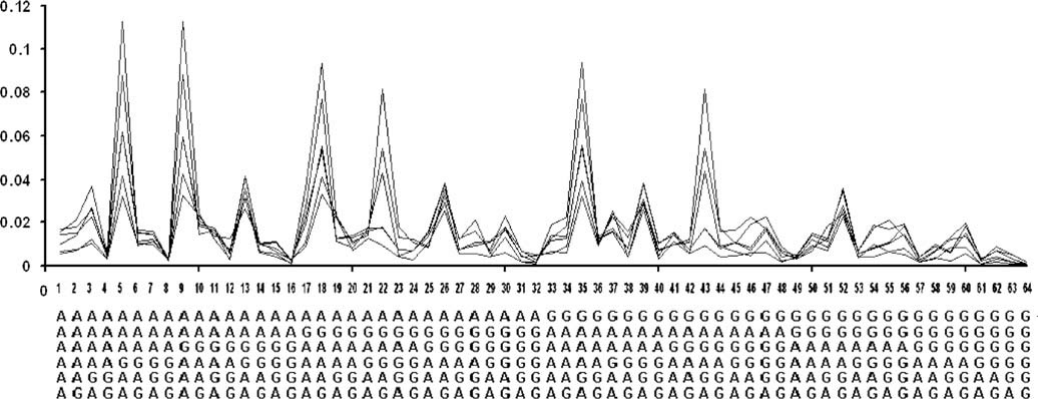
Similarity of the normalized hexa-nucleotide frequency spectra of the pure GA-sequences of chromosomes of various mammals (human chr. 1, chimpanzee chr. 1, mouse chr. 2, rat chr. 3, dog chr. 1, and cat segment 1). Abscissa: hexa-nucleotide (read from top to bottom) and its number; Ordinate: fraction of total counts of the particular hexa-nucleotide in the sequences.

Determining the distributions of hexa-nucleotides of different chromosomes for human, chimpanzee, mouse, rat, dog, and cat yielded 2 surprising results (Fig. 6):

1. They displayed sharp peaks.
2. The distributions of the various mammals were quite similar to each other.

Such spectral resemblances appear significant. In the first place, the hexa-nucleotide distribution of a randomly constructed set of pure GA-sequences would not show any peaks, but, of course, would display the same frequency for each hexa-nucleotide. Analysis of many such computer-generated pure GA-sequences confirmed this.

**Figure 7:**
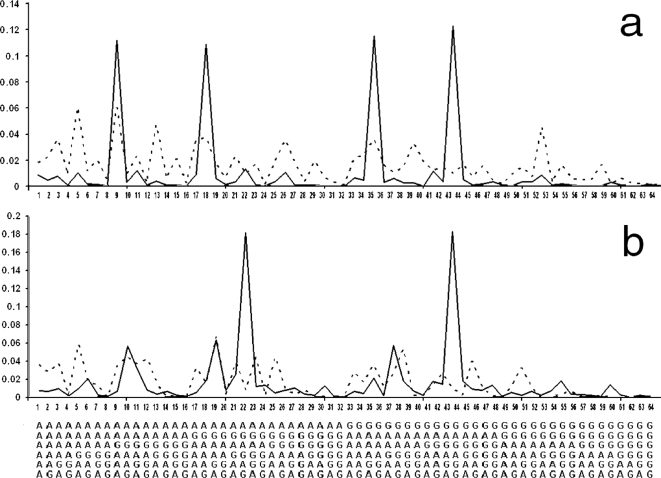
Dissimilarity of the normalized hexa-nucleotide frequency spectra of the pure GA-sequences of chromosomes of various non-mammals. (axes as in Fig. 6). a. Examples of non-mammalian vertebrates (continuous.: Zebrafish chr. 1; dotted: Chicken chr. 1). b. Examples of a plant and an insect (continuous: arabidopsis whole genome; dotted: drosophila chr. 2, 3, and X combined).

Secondly, the hexa-nucleotide distributions of other vertebrates such as zebrafish or chicken, and of non-vertebrates such as arabidopsis and drosophila melanogaster were not only very different from the mammalian ones, but also very different from each other (Fig. 7).

It appeared, therefore, that pure GA-sequences were not so much random chains of individual G's and A's, as varied concatenations of a set of short, repeated motifs.

These specific motifs are yet to be identified. It should be noted, though, that they need not be hexa-nucleotides themselves despite the similarity of the hexa-nucleotide spectra for different mammals. Instead, they could be smaller motifs of penta-, tetra-, tri- or bi-nucleotides. After all, it is easy to show mathematically that the described similarities between the hexa-nucleotide spectra of the GA-sequences of different mammals imply that their lower ranking penta-, tetra-, tri- and bi-nucleotide spectra are similar, as well.

### Are pure GA-sequences coding?

There are 8 codons that consist exclusively of G's and A's, namely AAA, AAG, AGA, AGG, GAA, GAG, GGA, and GGG. They code for Arg, Gly, Glu, and Lys. As it seemed conceivable that some pure GA-sequences coded for proteins that contained chains of these 4 amino acids, I tested how many pure GA-sequences were coding for mRNAs. Searching all human transcripts (440 [Mb]) for pure GA-sequences longer than 50 bases and excluding, of course, all poly-A sequences, I found 394 cases. In contrast, the entire human genome contained a total of 19,139 pure GA-sequences, indicating that at most 1.95% of them are transcribed into messenger RNAs. Thus, for all practical purposes the pure GA-sequences may be considered non-coding for proteins.

### Point mutations within of pure GA-sequences

Pure GA-sequences are subject to only six types of point mutations, namely [A→G], [G→A], [A→T], [A→C], [G→T], and [G→C]. The first 2 mutations would merely change the sequence of every pure GA-sequence it affected. In contrast, any of the last 4 point mutations would break it up into two adjacent pure GA-sequences with a characteristic property: The distance between the end of the previous and the beginning of the next pure GA-sequence would be exactly = 1, as demonstrated in Example 2.

### Example 2

If e.g. a T was inserted somewhere into a pure GA-sequence, as in

S_1_ = …GAAAGGAGAAGGAGAAG**T**AGAAGGAGAAGGAGA…,

it would create the 2 pure GA-sequences

S_2_ = …GAAAGGAGAAGGAGAAG and
S_3_ = AGAAGGAGAAGGAGA…

with S_3_ starting one base after S_2_ ended.

Conversely, if 2 adjacent pure GA-sequence were separated by a single T or C, it is hard to see how anything but a point mutation could have created this situation, especially since the average distance between pure GA-sequences of 145 [Kb] is so much larger.

Searching for such adjacent sequences among the pure GA-sequences of the entire human genome (total length of its 19,139 pure GA-sequences = 1,782,543 [b]) I found only 26 cases. Based on my previous results of 3.5 point mutations/[Kb] in vertebrate genomes [6] and the fact that only 4 out of 6 point mutations are able to interrupt pure GA-sequences, one would expect a total of 4213 point mutations, or more than 160 times as many as were actually found.

Of course, point mutations could also occur at the very ends of pure GA-sequences. In this case they would simply shorten it. However, basic statistics predicts that events at the exact ends are very much less likely, than the same events occurring somewhere in between. In view of the very low incidence of point mutations in the middle of pure GA-sequences, one can safely ignore any incidents at the ends.

One possible interpretation of this result may be that point mutations in pure GA-sequences are lethal even though 98% of them are not coding for proteins. This could mean that they are indispensable for certain control function. Alternatively, they may be protected from the mechanisms of point mutations [see 6], or exquisitely well repaired. Either way, the unknown functions of pure GA-sequences appear to be vital.

### Other kinds of pure base-restricted sequences

In order to place the above findings in a larger perspective, other kinds of sequences should be mentioned that, like pure GA-sequences, are restricted in their base composition. They must belong to one of the following cases:

- The sequence is restricted to 3 bases, i.e. exactly 1 base is missing (e.g. pure GAT-sequences).
- The sequence is restricted to 2 bases, i.e. exactly 2 bases are missing (e.g. pure GA-restricted sequences, such as pure GA-sequences).
- The sequence is restricted to 1 base, i.e. exactly 3 bases are missing (e.g. pure A-restricted sequences which, of course, are also known as poly-A sequences).

The following gives a brief overview of their numbers of occurrence in the example of human chr. 1, without going into the same details as in the case of pure GA-sequences.

### Pure A-, C-, G-, and T-sequences

I found only 7 poly-A sequences >50 bases at positions 10,975,771; 14,646,611; 23,614,091; 47,547,205; 63,200,867; 76,090,021; 223,258,125 in human chr. 1. The longest among them measured 69 [b]. Likewise, there were 11 poly-T sequences at positions 1,464,267; 8,188,809; 62,195,786; 108,001,574; 112,908,729; 151,285,300; 167,181,732; 187,136,220; 193,261,313; 214,206,557; 220,719,157 with the longest measuring 57 [b]. There were no poly-C or poly-G sequences longer than 50 bases.

### Pure AC-, AT-, GC-, CT-, and GT-restricted sequences

With the exception of pure GC-restricted sequences all other kinds were found in large numbers. As mentioned earlier, the complements of pure base-restricted sequences were probably base-restricted sequences that were placed there by a previous inversion. Therefore, the following overview of their numbers counts them together, unless they were already combined because they were their own complements such as pure GC- and TA-restricted sequences.

**Figure 8:**
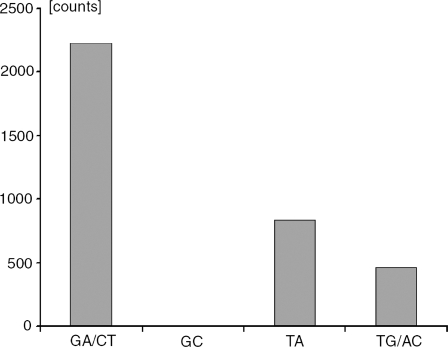
Numbers of the 4 possible pure 2-base-restricted sequences in human chr. 1. The counts of each are combined with the counts of its complementary sequences. It appears that the pure GA/CT-sequences are the most frequent while pure GC-sequences are too few to appear on the scale of the figure.

Counting the numbers of such sequences in human chr. 1 and 3, I found that pure GA/TC sequences were by far the most frequent (2273 cases), and that pure GC sequences effectively did not exist (5 cases) (Fig. 8). The predominance of pure GA-sequences was one of the reasons, this article focused on them. As mentioned earlier, random computer-generated control sequences contained not a single case of any of these base-restricted sequences.

### Pure CGT-, ACG-, AGT-, and ACT-restricted sequences

Their defining property, namely to miss exactly one base, is much less restricting than the requirements of the pure 1- and 2 base-restricted sequences. Consequently, they were found in much larger numbers. Again combining their numbers with the numbers of their complements, there were 4 times as many pure 3-base-restricted sequences that were missing the C or the G (37,271 cases), than there were sequences that missed the A or the T (9601 cases) in human chr. 1. There average length was 73 [b] (std.dev. 39 [b]).

## Discussion

Admittedly, the unknown functions of pure GA-sequences can only be determined experimentally. Yet, their extremely improbable existence, their ubiquity, their individuality, and the virtual absence of point mutations within them speak for their roles as essential parts of mammalian genomes. The following discussion tries to sort through some of the theoretical possibilities in order to propose the hypothesis that pure GA-sequences are possibly genomic sign posts.

### 1. Stabilizing-bracket hypothesis

Pure GA-sequences are purine homo-polymers on one strand and pyrimidine homo-polymers on the other. According to the Calladine rules [7], [8] this property renders them the most stable domains of the B-form of DNA. These rules are based on clashes caused by the propeller twists of neighboring pairs. The cumulative effect of many such clashes along stretches of ‘normal’ DNA sequences may put a certain strain on the double helix and leave it vulnerable to structural perturbations. Pure GA-sequences, placed every so often into the double helix, may help reduce the strain by providing stabilizing ‘brackets’.

This interpretation is consistent with the predominance of pure GA-sequences over all other 2-base-restricted sequences. Especially, it would explain the virtual absence of pure GC-sequences, as they are the most unstable sequences by the standards of Calladine [7], [8]. The need to place stabilizing ‘brackets’ at somewhat regular intervals would also explain that pure GA-sequences are ubiquitous and that their numbers seem to increase with chromosome size.

Further support for the above stabilizing-bracket hypothesis could be derived from the electrical dipole moment of the 4 nucleotides. G and C have the largest ground state dipole moment of about 7 [D], whereas A and T have lower moments of 2–4 [D] [9]. Thus the addition of electrical dipole moments along a purine homo-polymer would generate the same average value as along a pyrimidine homo-polymer and, in this way, minimize the electrical forces between the strands.

On the other hand, there are at least three strong reasons to doubt this interpretation. (A). It does not explain why the sequences in mammals should be individuals that share a common hexa-nucleotide spectrum. Uniform poly-A, poly-G, or poly-GA sequences as in the cases of maize, arabidopsis and C.elegans seem to be quite sufficient for the task of GA-mediated stabilization. (B). It would not explain why genomes seem to prevent or repair point mutations so carefully, since a few C's or T's among hundreds of G's and A's would not seriously compromise the postulated bracketing functions. (C). The stabilizing-bracket hypothesis does not explain why rodents like mouse and rat should have a 4–5 times higher spatial density of pure GA-sequences than primates like human and chimpanzee. After all, the sequences of mouse and rat are hardly so different from those of human and chimpanzee that they would require 4–5 times more brackets to stabilize them.

### 2. Sign-post hypothesis

#### Outline of the hypothesis

One may derive an alternative hypothesis for the function of pure GA-sequences based on the very failure of the stabilizing-bracket hypothesis to explain their individuality and the apparent protection from point mutations. These two properties could be considered as the defining qualities of natural genomic sign posts that could help guide transcription factors and polymerases prior to transcription or even during replication to their proper targets.

As pointed out in the Introduction, a necessary part of gene regulation would be the navigation of transcription factors and polymerases to the site of the gene. Especially, mammalian genomes that have sizes in the range of Giga-bases placing a large number of sign posts along the genome (e.g. 20,000 pure GA-sequences, or about 1000 per chromosome as in the case of the human genome) would create divisions into smaller domains that would be much easier to navigate.

Obviously, the sequences representing such genomic sign posts must be unique individuals, as were pure GA-sequences, lest their guidance would be ambiguous. They must also be protected from point mutations, as were pure GA-sequences, lest they would guide numerous transcription factors and polymerases to the wrong targets with presumably lethal results. In all likelihood, the presumable lethality itself would eliminate all such mutations.

Furthermore, all genomic sign posts should be as stable as possible, or else their guiding function would become unreliable. According to the above stabilizing bracket-hypothesis, pure GA-sequences would, indeed, provide this quality. In this sense, the sign-post hypothesis does not contradict the stabilizing bracket-hypothesis but supersedes it. In this way, all the supporting arguments for the stabilizing bracket-hypothesis would automatically become supportive for the sign-post hypothesis, as well.

Assuming that pure GA-sequences were, indeed, genomic sign posts, their presence may still not completely solve the problem of navigating the huge mammalian genomes. The average distance of 145 [Kb] between them may still appear to be rather large. Therefore, there may be a point where, similar to the case of modern air-traffic control, the polymerases (and perhaps even transcription factors) are literally ‘handed over’ to a finer resolving DNA-navigation system [see below and 14]. It is conceivable that some species use higher densities of pure GA-sequences than others before they advance to this sort of finer resolving system. Thus, the observation of a species-dependent spatial density of pure GA-sequences appears to be compatible with the sign-post hypothesis.

#### Supporting evidence from heat shock biology

The biology of heat shock seems to be a promising area to search for experimental support for the sign-post hypothesis. After all, one may expect that genomes under heat shock conditions must find genomic target sites very efficiently in order to mount a rapid stress response. The sign-post hypothesis would predict that under these circumstances the pure GA-sequences should rapidly become sites of interaction with transcription factors and polymerases on the way to their proper targets.

Indeed, HSP26 has been shown to require a large segment of (CT)_n_ repeats (i.e. (GA)_n_ repeats on the complementary strand) for full heat shock inducibility [10]. Furthermore, the heat shock factors HSF1 and HSF2 are themselves transcription factors, which bind to GAA repeats [11]. Upon heat shock, HSF1 rapidly and reversibly redistributes to the so-called HSF1 granules which are localized in the q12 heterochromatic region of human chr. 9 [12]. This region, ranging from about 65–73 [Mb] contains the average number of 55 pure GA-sequences. However, among them is one of the 3 longest in the chromosome (size = 423 [b]). It contains 50 GAGA motifs and 32 GAA motifs that could serve as binding sites for the GAGA factor and HSF1, respectively.

A recent report by Petesch and Lis [14] may even suggest a fundamental mechanism for the above postulated ‘handing over of polymerases to a finer resolving navigational system’. The authors discovered that the activation of the Hsp70 gene lead to rapid loss of nucleosomes that ‘freed’ the gene and sequences further downstream for unobstructed access of the polymerase to initiate transcription. The heat shock factor, known to bind to GAA motifs, and GAGA factor were required for this action.

### 3. Linker-hypothesis

The sign-post hypothesis also requires a genomic mechanism to detect and ‘read’ the sign posts. Based on known precedents of DNA binding proteins, one may speculate that there are a number of zinc finger proteins that bind specifically to different GA-motifs (or/and TC-motifs on the complementary strand), such as the GAGA factor in Drosophila that binds to (GA)_n_ repeats [13]. By ‘decorating’ individual pure GA-sequences, they would be forced into close proximity of each other and, thus, create a specific spatial line-up of adjacent proteins. The interactions between the links of such specific chains of GA-motif-binding proteins may, in turn, generate the functions of pure GA-sequences.

The common hexa-nucleotide spectrum of mammals could help identify the recognition sites of the GA-motif binding proteins. It could also suggest that all mammals use the same or similar sets of GA-motif-binding proteins.

It seems also possible, that some of the ‘decorated’ pure GA-sequences are able to bind to each other across space via specific linker proteins that bind to the GA-motif-binding proteins and, in this way, link different parts of chromosomes and even different chromosomes into a reproducible 3-dimensional structure. In other words, pure GA-sequences may not only serve as genomic sign posts, but perhaps also contribute to the reproducible formation of chromatin. This idea has been suggested earlier, based purely on data related to heat shock [10].

In summary, the article proposes the hypothesis that pure GA-sequences in mammals serve three functions. Predominantly they may serve as sign posts for genome navigation of polymerase, transcription factors and other DNA-binding proteins. Their exclusive composition from G's and A's may also render them as stabilizing brackets for the double helix. Finally, a sub-set of them may provide linkage sites between chromosomes to help create the 3-dimensional organization of chromatin.

## Materials and Methods

The genome sequences of human, chimpanzee, rat, mouse, and zebrafish were obtained from the UCSC site. The genome sequences of maize and arabidopsis were obtained from the TIGR site.

The analysis program, “GA_dnaorg.exe”, was written by G.A.-B. using Visual C++ (Microsoft, Redmond, WA). It split large genome sequences into segments of 8 Mb, searched them individually for pure GA-sequences and re-combined the data. It also offered various statistical evaluations of the data files. It implemented the Needleman-Wunsch algorithm [3] for the determination of sequence homologies in order to be able to perform rapid homology measurements on large numbers of sequence pairs. The program will be made available upon request.

## Supporting Information

Appendix S1 Pure GA-sequences of human chr. 1. The appendix lists all pure GA-sequences in human chromosome 1
(1.62 MB DOC)
