# Correction: Properties and Distribution of Pure GA-Sequences of Mammalian Genomes

**DOI:** 10.1371/annotation/7126ab55-ca2a-4202-9e66-fac899bd52ee

**Published:** 2009-01-21

**Authors:** Guenter Albrecht-Buehler

In the abstract, the number 160 is incorrect due to a logical flaw in its calculation. The number 160 should be replaced with "many". In addition, the Results section, the second and third paragraphs under the subheading "Example 2" contain a logical flaw. Please consider the following alternate text:

In short, each point mutation inside a pure GA-sequence would split it into 2 smaller GA-sequences. As a result, one may conclude that pure GA-sequences are conspicuously protected from point mutations based on the following argument.

My previous study of point mutations in vertebrate genomes [6] suggested that their incidence is proportional to the length of a DNA sequence at a rate of 3.3 point mutations/[Kb]. Thus, the long GA-sequences should become more likely fragmented by point mutations than the short ones. (In quantitative terms, the number of GA-sequences of size L should decay exponentially with increasing numbers M of mutations (Exponent = - r L M )). In contrast, the Pareto-distribution of the sizes of pure GA-sequences (eq. 1 and Fig. 2), is characterized by a conspicuous presence of many, very long sequences. Clearly, many million years of evolution failed to fragment these sequences into many small ones.

