# Supplementary material for: Properties and Distribution of Pure GA-Sequences of Mammalian Genomes
Source: PLoS One. 2008 Nov 27;3(11):e3818. doi: 10.1371/journal.pone.0003818 (PMC2585066; doi:10.1371/journal.pone.0003818)
Supplement: Appendix S1 — Pure GA-sequences of human chr. 1. The appendix lists all pure GA-sequences in human chromosome 1 (1.62 MB DOC) [file pone.0003818.s001.doc]

**Pure GA-sequences of human chr.1 (the terminating base is included)**

| index | SEQUENCE | POSITION |
| --- | --- | --- |
| 1 | AAAAAAAAGAAAGAGAGAGAGAGAGAAAGAGAAAGAAAGAAAGAGAAAGAAAGAAAGAAAGAAAGAAAGAAAGAAAGAAAGAAAGAAGAGAAAGAAAGAAAGAAGAAAGAGAAAGAAAGAAAGAAAGAGAGAGAGAAAGAGAGAGAAAGAAAAAGAAGGAAAGAAAGAAAGAAAGAAAAAAGAAAGAAAAAGAAAGAAAGAAAGAAAGAAAGAAAGAAAGAAAGAAAGAAAGAAAGAAAGAAAGAAAGAAAGAAAAGC | 73651 |
| 2 | AAAAAGGAAAAAAGGAAGGAAAGAAAAAAGGAAGGGAGGAGGGAAGGAGGGAAAAAGGGAAGGAGGGAAGGAAAGGAAGGAAGGGAAAGAAGGAAAGGAAGGAAGGGAAAGAGAGAAAGAGGGAAGGAGGAAGGGAGGGAAGGAGGGAGGGAGGGAGAGAGAGAGGGAGGGAGGGGAAGGGAAGAAAAGGGAAGAGAAGGGAAAGGAGGAAGAAAAGGAAAGGAAAGGAAT | 152034 |
| 3 | AAAAAAAAAAAAAAAAAAAAAAAAAAAAAAAGAGAGAGAGAGAAAGAAAGAAAGAGGGC | 164767 |
| 4 | AAAAAAAAAAGAGAGAGAGAAAGAGAAAGAAAGAAAGAAGAGAGAGAAAGAAAGGGAGAGAAAGAAAAAGAAAGAAAGAAAGAAAGAGAAAGAAAGAAAGAAAGAAAGAAAGAAAGAAAGAAAGAAAGAAAGAAAGAAAGAAAGAAAGAAAAAGAAAGAAAAGAAAGAAAGAAAAGC | 628861 |
| 5 | AAAAAGGAAAAAAGGAAGGAAAGAAAAAAGGAAGGAAGGAGGGAAGGAGGGAAAAAGGGAAGGAGGGAAGGAAAGGAAGGAAGGGAAAGAAGGAAAGGAAGGAAGGGAAAGAGAGAAAGAGGGAAGGAGGAAGGGAGGGAAGGAGGGAGGGAGGGAGAGAGGGAGGGAGGGGAAGGGAAGAAAAGGGAAGAGAAGGGAAAGGAGGAAGAAAAGGAAAGGAAAGGAAT | 737878 |
| 6 | GAGAGAGAGAGAAGGAGAGAAGGAAGGAAGGAGGGAGAGAAAGGGAAAGAAGGAAAGAAAC | 863687 |
| 7 | AGGGAGGGAGAGAAAGAGGAAGGGAGAGGAAGGAAGGAGGGAGAGAAAGAGGGAAAGGGAAAGAAGGAAGGAAAGAAAC | 863756 |
| 8 | AGGGAGGGAGAGAAAGAGGAAGGGAGAGGAAGGAGGGAGGGAGGGAAAGGAGAC | 863843 |
| 9 | GAGAAAGAAAAAAGAGAGAAGAGAGAGAGGAGAAGAGAGAAAAGAAGAGAAGAGAGAAGAGAAGAGAAGGAAAGAGAGAGAGAGAAT | 950544 |
| 10 | AGGGGAGGGGAAGAGAGGGGAGGGGAGGGGAAGAGAGGGGAGGGGAGGGGGAGAGAGGGGAGGGGAGGGGAGGGGAAGAGGGGAGGGGAGGGGAAGAGAGGGGAGGGGAGGGGAAGAGAGGGGAGGGGAGGGGAAGAGAGGGGAGGGGAGGGGAAGAGGGGAGGGGAGGGGAGGGGAGGC | 1448613 |
| 11 | AAGAAAGAAAGAAGGAAAGAAGGAAAGAAGGAAAGAAGGAAAGAAAGAAAGGAAGAAAGGAAGAAAGGAAGAAAGGAAGAAAGGAAAGAAAGGAAAGAAAGGAAAGAAAGAAAGAAAAGAAAGAAAGAGAGAAAGAAAGAAAGAAAGAC | 1864708 |
| 12 | AAGAAAGAAAGAAAAAAGGAAGGAAGGAAGGGAGGGAAAGAGAGAAAGAAGC | 1916135 |
| 13 | GGAGGAAGGAAGGGAGGGAGGGGAAGGAGGGAGGAAAGGAGGAAGGAAGGAAGGAAGGAGAGGAAGAAAGGAAGGGAGGC | 2798907 |
| 14 | AGGGAGGAAGGAAGGAGGAAGGAGGAAGGAAGGAAGAAAGGAAGAAGGAAAGAGGAAGGAAGAAAAGAGGAAAGAAAGGGAGGAAAGGAAGGAAGGAGGAAGGAAGGAGAAAGGGAGGAGGAAGGAAGGAAAAAAGGAGGAAAAAAGAAGGAAGGAGAAAGAAGGAGGGGGAAGGAGGAAGAC | 2799002 |
| 15 | AAGAGGGAGGAAGGAGGGAGGAAAGAGGGAGGAAGAGGAAGGAAGGAGGGAGGAAGGAGGGAGGAAGAGGAAGGAAGGAGGGAGGAAGGAGGGAGGAAGAGGAAGGAAGGAGGGAGGGAGGAC | 2799185 |
| 16 | AGAGAGGGAGAGAGGGAGGAGAGAGGAAGGGAAGGAGGGGGGAGAGAGGAAAGGAAGGAGGGAGGAGAGAGGAAGGGAAT | 2956101 |
| 17 | AGAGAGGGAGAGAGGGAGGAGAGAGGAAGGGAAGGAGGGGGGAGAGAGGAAAGGAAGGAGGGAGGAGAGAGGAAGGGAAT | 2956204 |
| 18 | AGGAAGGAGGAAGGAGAGAGAAGAGGGAGAAGAGGAGGAGGAAGAGGAGGGGGAGGAAGGGGAAGAGGAGGAGGAC | 3140659 |
| 19 | GGAGAGAGAGAGAAGAGGAGAGGAGAAGAGGAGAGGAGAGGAGAAGAGGAGAGGAGAGGAGAGGAGAGGAGAGGAGAGGAGAGGAGAGGAGAGGAGGAGAAGAGAAGAGAAGAAAGAAAAGAAAGAAAAGT | 3179735 |
| 20 | AAAAAAAAAAAAAAAAAGAAAAAAAAAAAGGAAGGAAGGAGGAAAAGAAAGC | 3241055 |
| 21 | AGGGAGAGGAAGGGGAGGAAGGAGGAGGAAGGAGGGAAGAGGAGGAGGAAGAGGAGAAC | 4017912 |
| 22 | GGAGAGGGGGGAGAAGGAGAGAGGGAAAGAGAGGGGGAAGGAGGGGGAGAAAGAGGGAGGAAGGGGAGAGGGAGAAGAGAGGGAGAGAAAT | 4020174 |
| 23 | AGAGAGAGGGAGAAGGAGAGAGAGGGAAAGAGGGAGAAGGAAGGGGGAGAAAGAGGGAGAAGC | 4020340 |
| 24 | AAAAAGAAGAAGAAGAAAGAAGAAGAAGGAGGAGAAGAAGAAGGAGAAGGAGGAGGAGGAGGAGGAGGAGGAAGAT | 4349435 |
| 25 | AGAGGGAGGGGAGGAGGAGGGAGGAGAGGAGGAGGAGGGAGGAGAGAGAGGAGGAGGGAGAGGAGGAGGAGGGAC | 4722577 |
| 26 | AGAAGGGGAGGGGGAAGGGGAGGGAAGGGAAGGGGGAAGAGAAGAGAAGGGAAGAGGAGGGGAGGAGAGGGGAGGGGAGGAC | 4930303 |
| 27 | GGAGGGGAAGGGAGGGGAGGGGAAGGGAGGGGAGGGAGGGGAGGGGAAGGGAGGGGAGGAGAAGGGAGGGGAGGGGAGGGC | 4939599 |
| 28 | GGGGAGGGAAGGAGGGAGGAAGGAAGGAAGGAAGAAAGGAAGGAAGGAAGGAAGGAAGAAAT | 5182664 |
| 29 | AAAGAGAGAAAGAAAGAAAGAAAGAAAGAAAGAAAGAAAGAAAGAAAGAAAGAAAGAAAGAAAGAAAGGAAGAAAGAAAGAGAAAGAAAGAGAGAAAGAGAGAGAAAGAAAAAGAAGGAAAGAAAGAGAGAGAGAAAGAGAC | 5285663 |
| 30 | AGAGAGAAAGAGAGAGAGAAGGAAGGAAGGGAGGGAAGGAAAAGGAAAGGAAAGGAAAGAAGAGAGGGAGGGAGT | 5285805 |
| 31 | GAAGGAAGGAAGGAAAAGGAAAGGAAAGAAGAGAGGGAAGGAGGGAAGGAAGGAAGGAAGGGAAGAAAGAAAAAGAAAGAAAGAAAGAAAGAAAGAAAGAAAGAAAGAAAGAAAGAAAGAAAGAGAAGAAAGGAAGAT | 5285880 |
| 32 | AGAGGAAGGGAGGGAGAGGGGGGGAGGAAGAGAGAGAGAGAGAGAGAGAAGC | 5423199 |
| 33 | GAGAAAGGAAGGAAGGAAGGAAGGAAGGAAGGAAGGAAGGAAGGAAGGAAGGAAGGAAGGAAGGAAGGAAAGAAAGAAGGAAGGAAGGAAAGAAGGAAGGAAGGGAAGGAAC | 5650162 |
| 34 | GGAAAGAGAGAGAGAAAGGAGAAGGAAGGAAGGAGAGAGAGAAAAAGAAAGAGAAAGT | 5650338 |
| 35 | GGGAGGGAAGGGAGGAGAGAAGGAGGGAGAGAGGGAGGGAGGGGAGGGAGGGAAGGAAGGAAGGGAGAGAGGGAGGGAGGGAGGGAAAGAGAGAAT | 5650511 |
| 36 | AAAAGAAAGAGAGAGAGGGAAAAAAAGAAAGAAAGAAAGAGAGAGAGAGAAAGAAAGAAAGAAAGAAAGAAAGGAGAGAAAGAAAGAGAAAGAGAGAGAGAAAGAGAT | 5696034 |
| 37 | AAAGAGAGAAAGAGAGGAAGGAAGGGAGGAAGGAAGGAAGGAAGGAAAGAAGGGAGGGAGGAAGGAAGGAAGGAAGGAAGGAAGGAAGGAAGGAAGGAAAGT | 5696142 |
| 38 | GGAAGGAAAGGAAGGGAAGAAAAGGGAAGGAAGGGAAGGAAGGGAAGGAGGGAAGAGGGAAGGAAGGAGGGAAGGAGAAGGAAGT | 5705041 |
| 39 | GGAAGGGAGGAAGGAAGGGAGGAAGGAAGAAGGGAGGGAGGGAGGGAAGGAAAAAGAT | 5705126 |
| 40 | GAAGGAAGGAAGGAAGGAAAGAGGGAAGGAAGGAAGGAAAGAAGGAAGGAAGGAAGGAAGAAAAAGAAAAGAGAGAAAGAGAAAGAAAAAGAGAGAGAGAGAT | 5730520 |
| 41 | AGAGGGAGGGAGGAAGAGAGGGAGGGAGGAGAGGGAGGGAGGAGGAGAGGGAGGAAGGAGAGGGAGAGAGAGGGAGAAAGAGAGGAGGAAGAAGGAGAT | 5741956 |
| 42 | GGAGGAGGGAGAAAGGAAGGGAGGGAGAAAGGGAGGAGGGAGGGAGGGAAGGAAGGAGGGAGGGAGAGAGAAAGGAGAAAGAGGGAGGAAGGGAGGGAGGAAT | 5742055 |
| 43 | GAGAAAGAAAGAAAGGAAAGAAAGGAAAGAAAGAGAGAGAGAGAGAGAGGGAGGGAGGGAGGGAGGGAGGGAGGGAGGGAAGGAGGGAAGGAAGGAAGGAAGGAAGGAAGGAAGGAAGGAAGGAAGGAGAGAGAGAAAGAAAAGAAAGAAAGAAAGGGAGAGGGAAGGAAGGAAGGAAGGAAAGC | 5815624 |
| 44 | GAAAGGGAAAGGGAGGGGGAGGGGAGGGGGAAGGAGAGGGGAGGGGAGAGGAGGAGAGGGAGC | 5972197 |
| 45 | AAGAAAGAAAGAGAAAGAAAGAAAAGGAAGGAAAGAAGGAAGGAAAAAGAAAAGAAAAGAAAGAGC | 6026935 |
| 46 | AGGGAGGAGAGAGGGAGGGAGGGAGGGGGAGAGAGAGAGAGAGAGAGAGAGAGAGT | 6564324 |
| 47 | AGAAAAGAAAAGAAAAGAAAAGAAAAGAAAAGAAAAGAAAAGAAAAGAAAAGAAAAGAAAAGAAGGC | 6663841 |
| 48 | GAGGGAGGGAGGAAGGGAGAGAGAGAGAGAGAGAAAGAAAGAAGGAGAAGAAGGAGGAGGAGGAGGAGAC | 7100567 |
| 49 | AGGAGGAGAGGGGGAAGGAGAGGAGGAAGGAGAGAAGGAGGAGAGGGGAGGAGGC | 7223940 |
| 50 | AGAGAGAGAGAGAGAGGAAAGAAGAAAGAAAGAAAGAGAGAAAGAAAGAAAGAAAGAAAGAAGGAAGGAAGGAAAGAAGGAAGGAGAAAGGAAAGAAAGAAAAGAC | 7588407 |
| 51 | AAGAAAGGAGGGAGGGGAAGGAAGGAAGGAAGGAAGGAAGGAAGGAAGGAAGGAAGAAAGAAAAGAAGGAC | 7588513 |
| 52 | GGGGAGAGGGAGAGGGAGAGGGAGAGGGAGAGGAGGGAGAGGAGGGAGAGGGAGAGGAGGGAGAGGGAGAGGAGGAGC | 7596316 |
| 53 | AAGGGAGGGAAGGAAGGGAGGGAGGAAGGGAAGGAAGGAGGGAGGGAGGGAGGGAAGGAGGGAGGT | 7686271 |
| 54 | AAAAAAAAAAAAAAAAAAAAGGAAAAGAAAAAAAAAGAAAAAGAAAAAAAAGAAAAAGAAAT | 7866627 |
| 55 | AAGAAAGAAAGAAAAGAAAGAAAGAAAGAAAGAAAGAAAGAAAGAAAGAAAGAAAGAAAGAAAGAAAGAAAGAAAAT | 7885852 |
| 56 | AAGAAAGAAAGAAAGAAAAGAAAGAAAGGAAGAAAGAAAGAAAGAAAAAGAGAAAGAAAGAAAGAAAGAAAGAAAGAAAGAAAGAAAGAAAGAAAGAAAGAAAGAAAGAAAAGAGGGAGGGAGGGAGGGGAAAC | 7900727 |
| 57 | AAAAAAAAAAAAAAAAAAAAGGAAAAGAAAAAAAAAGAAAAAGAAAAAAAAGAAAAAGAAAT | 8023493 |
| 58 | AAGAAAGAAAGAAAAGAAAGAAAGAAAGAAAGAAAGAAAGAAAGAAAGAAAGAAAGAAAGAAAGAAAGAAAGAAAAT | 8042718 |
| 59 | AAGAAAGAAAGAAAGAAAAGAAAGAAAGGAAGAAAGAAAGAAAGAAAAAGAGAAAGAAAGAAAGAAAGAAAGAAAGAAAGAAAGAAAGAAAGAAAGAAAGAAAGAAAGAAAAGAGGGAGGGAGGGAGGGGAAAC | 8057593 |
| 60 | GGAAGAGGAGGAGGAAGAAGAAGGAGAAGAAGGAAGAGGAAGAGGAGGAGGAAGAAGGAGAAGAAGAAGGAAGAGGAAGAGGAGGAGGAAGAAGAAAGAAGGAGGAGGAGGAGAAGGAGAAAGAAGAAAGAAGGAAGAAGAAAAGGAGGAGAAGGT | 8309037 |
| 61 | AAGGGAAAGAAAGGGAGAGGGAGGGAAAAAGGAGAGGGAAGAGAGAGAGAGAC | 8320974 |
| 62 | AAAAAAAGAAAGAAAGAAGGAAAGAAGGAAAGAAAGAAAGAAAGAAAGAAAGAAAGAAAGAAAGAAAGAAAGAAAGAGGGAGGGAGGGAGGGAGGGAGGGAGGGAGGGAGGGAGGGAGGGAGGGAGGGAAAAAAAGAGAGAAAGAGAGGC | 8380084 |
| 63 | AAGAAAAAAAGAAAGGAAGGAAAGAAAGAAGAAAAAGAAAGAAAGAAAGAGAGAAAGAAAGAAAGAAAGAAAGAGAAAGAAAGAAAGAGAGAGAGAGGAAGC | 8380234 |
| 64 | AAAAGAAGAGAAGGGAAGGGGAGGGGAGGGGAGGGGAGGGGAGGGGAGGGGGAGAAAGGAAGAAAGAAAGAAAGAAAGAAAGAAAGAAAGAAAGAAAGAAAGAAAGAAAGAAAGAAAGAAAGAAAGAAAGAAAGAAGGAAAT | 8394383 |
| 65 | AAAAAAAAAAAAAAAGAAGAAAGGAAGGAAGGAAAGAAGGAAGGAAGGGAGGAAGGAAGGAAGGAAGGAGGGAAAT | 8459765 |
| 66 | AAAGGAAGAAAAAAAGAAGAGGAGGAAGAAGGGGAGAAAGGGAGGAGGAAGAC | 8661220 |
| 67 | AAAAAAAGAAGGAAGGAAGGAAGGAAGGAAGGGAGGGAGGGAGGGAGGGAGGGAGGAAGGAAGGAAGGAAGGAAGGAAGGAAGGAAGGAAGGAAGGAAGGAAGGAAGT | 8755762 |
| 68 | AAAAAAGAAAGAAAGAGAGAAAGAGAGAAAGAAAAGAAAGAGAGAAAGGAAGAAAGAAAGAGAAAAAAGAAAAGGAAGGAAAGC | 9095752 |
| 69 | AAAAAAGAAAGAAAGAAAGAAAGAAAGAAAGAAAGAAAGAAAGAAAGAAAGAAAGAAAGAAAGAAAGAAAGAAAC | 9128890 |
| 70 | AAAAAAGAAAGAAAGAGAGGGAGGGGGAGGGAGAGAGAGAGAGAGAAAGAAGGAAGGAAGGAGAAAGAAAGAAAAAGAAAGAAAGAGAGGGAGGGAGGGGGGAGAGAGAAGGAAGGAAGGAGAAAGAAAGAAAAAGAAAGGAAGGAAGAAAAGAAAGAAAAAAGAGAAAGAAAAGGAAGGAAGGAGAAAGAAAGAGAGAAAGC | 9135447 |
| 71 | AGGAAGGAAGGAAGGAAGGGAAGGAGGGAGGGAGGGAAGAAGAGAGAGAGGAAGGAAGGAAGGAAGGAAGGAAAGGAGAGAGGAAGGAAGGAAGGAAAAC | 9167794 |
| 72 | AAAAAGAAAGAAAGAAAAAGAGAAGAAGGAAGGAGGGAAAAAAAGAAAAGGAAGGAAGGAAGGAAT | 9202370 |
| 73 | AGGGAGAGAGGGAGGGAGGGAGAGAGGGAGGGAGGGAGGGAAAGAAGGGGAAGAAGGAAGGAAGGAAGGAAGGAC | 9228930 |
| 74 | AAAAAAAAAAAAAAAAAAAAAAAAGAAAAAAAGAAAAGAAAAGAAAGAAAAAAGAAAAAGT | 9385138 |
| 75 | AAAAAGGAAAGAAAGGAAGGAAGGAAGGAAGGAAGGAAGGAAGGAAGGAAGGAAGGGAGGAAGGAAGGAAAGAAAGAAGAAAGAAAGGAAAGAAAGAAAGAAAGAAAGAAAGAAAGAAAGAAAGAAAGAAAGAAAGAAAGAAAGAAAGAAAGAAAGAAAGAAAGAAAGT | 9530873 |
| 76 | AAGAAGAAGAAGAAGAGGAAGAAGAAGAAGAAGAAGAAGAGGAAGAGGAAGAGGAGGAGGAGGAGGAGGAGGAGGAGGAGGAAGAGGAGGAAGAGGAGGAAGAGGAGGAAGAGGAGGAGGAGGAGGAGGAGGGAAGAAGAAGAAGAAGAAGAAGAAGAAAAGAAGAAGAAGAAGAAC | 9531395 |
| 77 | AGAGAGAAAGGAAAGAAAGGAAGGAAGGAAGGAAGGAAGGAAGGAAGGAAGGAAGGAAGGAGAAAGAAGAAAGAAGAAAGAAAGAAAGAAAGAAAGAAGAAAGAAAGAAAAGAAAGAAAGAAAGAAAGAAAGAAAGGAAGGAAGAAAGAAAGGAAAGAAAGAAAGAAGAAAGAAAGAAGAAAGAGAGGGAGGAAGGGAGGGAGGGAGGGAAAGAAAGAAAGAAAAGAAC | 9621243 |
| 78 | AAAAAAAAAAAAAAAGAAGAAGAAAAGGAAAGAAAGAAGAAAAAGAAGAAAAAAGAGAGAAAGAAGAAAGAAAGAAAGAAAGAAGGGAGGGAGGGAGGGAGGGAGGGAAGGAAGGAAGGAAGGAAGGAAGGAAGGAAGGAAGGAAGGAAGGAAGGAAGGAGGGAGGGAAGGAAGAAAGAAAGAAAGAAAAGT | 9654070 |
| 79 | AAAAAAGAAAGAGAGAGAGAGAAAAAAAAAGAGAAAGAGAGAGAGGGAGGGAGGGAGAGAGGGAGGAAGAAAGGAAGGAAGGAAGGAAAGAAGGAGGGAAGGAAGGAAGGAAGAAAAAGAT | 9667293 |
| 80 | AAAGAAAAGAGGAAGGAAGGAAGGAAGGAAAAGAAAGAAAGAGAGAGAAAGAT | 9775460 |
| 81 | AAAGAAAAGAAAAGAAAGAAGAGAAAGGAAGAAAAGAAAGAGAAAGAAGGAAAGGC | 9794265 |
| 82 | GAGAGAGAGAGAGAGAGGGGAAGGAAGGAAGGGAGGGAGGGAGGGAAGGAAGGGAGGGAGGGAGGGAGGGAGGGAAGGAAGGGAAGAAAGAT | 9794615 |
| 83 | AAAAAAAAGAAAAGAAAAAAGAAAAAGAAAGAAAAGAAAAGAAAAAAAGGAAT | 9801813 |
| 84 | AAAGGAGAAAGGGAAAGAGGGAGGGAGGGAGGGAGGAAAAGGGGGAAGGAGGGAAGAAGGGAT | 9920112 |
| 85 | GAGGGAGAGAGGGAGAAAGGGGAAAAGGGAGGGAGGGAGAGGGAGGGAGAGAGGAAGGAAAGGAC | 9920212 |
| 86 | AAAGAAAGAGAGAGGGAGAGAGGGAGAGAGGGGAAGAGGGGAAGAGGGGAAGAGGGAGGGGGAGGGGAAGAGGGAGGGGGAGGGGAGGGAAAAAGAAAGAAAAGAAAAAC | 9949290 |
| 87 | GAGAAAGAGAGAGAGAGAAAGAGAGAGAAAGAGAGGGAGAGAGAGAGAGAGAAAGGAAGGAGGGAGGGAGGGAGAGAGGGAAGGAAGGAAGGGAGAAGGC | 9961225 |
| 88 | AAAGAAAGGAAAGAGAGAGAGAGAAGAAAGAAAAGAAAGAAAGGAAAGAAAGAGGAAGGAAGGGAAAGGAGGAAGGGAGGAAGGAAGGAGGGAAGGAAGGAAGGAAAGAAC | 9989060 |
| 89 | GAAAGAAAAGGAAAGGAAGAAGAAAGAAAGAAAGAAAGAAAAAGAAAGAGAAAGAAAAAGAGAAAAT | 9989171 |
| 90 | AAAAAGAAAGAGAGAGAGAGAGAGAGAGAGAGGGAGGGAGGGAGGAAGGGAGGGAAGAAAAGAAAGC | 10068041 |
| 91 | AAAAAAAAAAAAAAAAAAAAAAAAAGAAAGAAAGAAAAGAAAAGAAAAAAGAAAAAAGAT | 10654472 |
| 92 | GGGAGAGGGGGAGAGAGAGAGAGGGAGAGAGAGAAAGAGAGGAGAAAAGAAAGC | 10860804 |
| 93 | AAAGAAAGAAAGAAAGAAAGAAAGGGAAGAAAGGAAAGAAAGGAAAGAAAGAAAGAAAC | 10865498 |
| 94 | AAAAAAAGAGAGAGAGAAAAAGAGGGGGAGAGAGAGAGAGAGAAGAGAGAGAT | 10942908 |
| 95 | AAAAAAAAAAAAAAAAAAAAAAAAAAAAAAAAAAAAAAAAAAAAAAAAAAAGAGAGAGAGAGAC | 10975823 |
| 96 | AAAGAGAGAGAGAGAGAAAGGGAGGAAGGGAGGGAGGAAGGAAGAAAGGAAGGAAGGAAGGAAGGGT | 11050763 |
| 97 | AGAAGAGAGAAAGAAAAAAAGAAAGGAAGGAAGGAAGGAGAAGAAAAAAGGAAAGAAAAGAAAC | 11068847 |
| 98 | AGAAAGAAAGGAAAGAAAGAAAAAAGAAAGGAAGGAAGGAAGGAAGAAAGAAGGAAAAAGAAAGAAAGAAAAT | 11068911 |
| 99 | GGGGAAGGGAGGGAGGAAGGAAGGAAGGAAGGAAGAAAGGAAGGAAGGAAGGAAGGAAAAGAAGGAGC | 11121312 |
| 100 | AGAGAGGGAGAGAGAGAGAGAAAGAGAAAAGAAAGGAAAGGGAAGGAAGGAAGGAGAAAGGAAGGAAGGC | 11132657 |
| 101 | GAAAAAAAAGAAAGAAAGAAAGAGAGAAAGAAAGAGAGAAAGAGAGAAAGAGAAAGAGAAAGAAAGAAAGAAAGAAAGGAAAGAAAGAAAGAGAAAGAAAGAAAAGAAAGAAAGAAAGAAAGGAAGGAAGGAAGGAAGGAAGGAAGGAAGGAAGGAAGGAAGAAAGAAAGAAAGAAAGAAAGAAAGAAAGAAAGAAAGAAAGAAAGAAAGAAAGAAAGAAAGAAAGAAAGAAGC | 11138492 |
| 102 | AGAGAGGGAGAGAGAGAGAGAAAGAGAGAGAGAGAGAGAGGAAGGAAGGAAGGAAGGAAGGAAGGAAGGAAGGAAGGAAAGAAGGGAGGAAGGAAAGAT | 11139208 |
| 103 | AAAGAAAGAAAAGGAAGGAAGGAAGGAAGGAAGGAAGGAAGGAAGGAAGGAAGGAAGGAAGGAAGGAAGGAAGGAAAGC | 11143311 |
| 104 | AAAAAAAAAAAAAAAAAGAAAAGGAAAGAAAGAAAGAAAAGAAAAAGAAAAC | 11156595 |
| 105 | GGGAAGGGAGGGAAGGGGGGGAAAGGGAGGGAAGGGAGGAAAGGAAGGGAAGGAAAGGAAAGAAAGGAAAGAAAAGAT | 11162505 |
| 106 | AAGAAAGAAAGAAAGAAAGAAAGAAAGAAAGAAAGAAAGAAAGAAAGAAAGAAAGAGAGAGAGAGAGAGAGAGAGAGAGAGAGAGAGAGAGAGAGAGAGAGAGAGAGAGAAAGAAAGAC | 11616330 |
| 107 | AGAGAAAGGAGAGAGAGAGAGAGAAAGGAGAGAGAGAGAAAGAAAGAAAGAGAGAAAGAGAAAGAAAGGGAGAAAGAGAGAAAGAAGGAAAGAGAGAAAGAGAGAGAGAGAGAAAGAGAGAGAGAGAAAGAAAGGAGAAAAGAAAGGAGAAAAGAAAAGAAAGC | 11713029 |
| 108 | GAAGGAAGGAAGGAAGGAAGGAAGGAAGAAAGAAAGAAAGAAAGAAAGAAAGAAAGAAAGAAAGAAAGAAGGAAGAAAGAAAGAAAGAAAGAGAAAAAGAAAGAAAGAGAAAGGGAGGAAGGGGAAGGGAAGGGAAGGGGAGAGGGAGGGGAGGGAGGGAAGAAGGAAGGAAGGAAGGAAGGAAGGAAGGAAGGAAGGAAGGAAGGAAGGAAGGAAGGAAGGAAAAAGAAAAAGT | 11783063 |
| 109 | GGGAAGGAGGGAGGAAGGAAGGAAGGAAGGAAGGAAGGAAGGAAGGAAGGAAGGAAGGAAGGAAGGAAGGAAGGAAGGAAAAGGAAGT | 11783635 |
| 110 | GAAAAAAGAAAGAAAGAAAGAAGGAAAGAAAGAAAGAAAGAAAGAAAGAAAAC | 12011323 |
| 111 | AAAAAAAAAAAAAAAAAGAAAGAAAGAAAGAAAAAAGAAAAGAAAAGAAAAAGAAAAAT | 12053343 |
| 112 | GGAGGAAAAAAAAAGGAGAGAGAAGAAGAGGAAGAAAGAAGAAGAAAGGAAAAGGGAGGAGGAGGGGAAGGGGAAC | 12078326 |
| 113 | AAAAGAGAAAGAAAAAAAAGAAAGAAAGAAAGAAAGGAAAGAAAGAAAAAGAC | 12199580 |
| 114 | AGGAAGGAAGGAAGGAAGGGAGGAAGAGAGGGAGGGAGGGAGGGAAGGAGGAT | 12576872 |
| 115 | AAAAAAAAAAAAAAGAAAGAAAGAAAAAAGAAAAAGAAAGAAGAAAGAAAGAAAGAAGAAAGAAAGAAAGAAAGAAAGAAAAT | 12694654 |
| 116 | AAAAAAAAAAAAAAAAAAAAAAAAAAAAAAAGAAAAAGAAAAAGGAGAGAGAGAGAGC | 13096944 |
| 117 | AAAAAAAGAAAAAGAAAGAAAGAAAGAGAGAGAGAGAGAGAGAGAGAAAGAAAGAAAGAAAAAC | 13131214 |
| 118 | AAAAAAAAGGAAGGAAAGAAGGAAGGAAGGGAGGGAGGGAGGGAGGGAGGGGAGAGAAAAAGAC | 13149623 |
| 119 | AAGAAAAAGAAAGAAAGAAAGAAAAAGAAGGAAAGAAGGAAGGAAAGAAAAAGAAAGAAAGAAGAAAGAAAGAAAGAAAGAAAGAAAGAAAGAAAGAAAGAAAGAAAGAAAGAAAAAGAGC | 13149741 |
| 120 | AAAAAAAAAAAAAAAAAAAAGAAAAGAAAAGAGAGAGAGAGAGAGAGAGAGAGAAC | 13154850 |
| 121 | GGAAAGAAAGAAAGAAAGAAAGAAAGAAAGAAAGAAAGAAAGAAAGAAAGAAAGAAAGAAAGAAAGAAAGAGAAAGAAAGAGAAAGAAAGAAAGAAAGAGAT | 13228324 |
| 122 | GGAAGGAAGGAAGGAAAGAAAGAAAGAAAGAAAGAAAGAAAGAAAGAAAGAAAGAAAGAAAGAAAGAAAGAGAAAGAGAGAGAAGGAAAGAAAGAAAGAAAGAGAAAGAGAGAGAAC | 13228440 |
| 123 | GAAAGAAAGAAAGAGAGAGGGAGAGAGGGAGGGAGGGGGAGAGAAAGAGAGAGAGAAGAAAAGAAAAGAGAAGAGAAGAAAAGAAAAGAAAAGAAAAGAAAAGAAAAGAAAAGAAAAGAAAAGAAAAGAAT | 13247590 |
| 124 | AAAGAAAGAAGGGAGGGAGAGAGGGAAAGAAGGAAAGAAGAAAGAGAGAGAAAGAGAAAGC | 13266524 |
| 125 | AGAGGAAGGAAGGAAGGAAGGGAGGGAGGAAGGAAGGAAGGAAGGGAGGGAGGGAGGGAGGGAGGGAAGGAGGGAGGGAGGGAGGGAAGGAAGGAAAT | 13293893 |
| 126 | GAAAGAAAGAAAGAAAGGAAAGAAAGAAAGAAAGAAAGAAAGAAAGAAAGAAAGAAAGAAAGAAAGAGAGAGAGAGAAAC | 13293991 |
| 127 | AAGAAAGAAAGAAGGGAGAGAGAGGGGGAAAGAAAGAAAGAAGGGAGGGAGAGAGGGAAAGAAGGAAAGAAGAAAGAGAGAGAAAGAGAAAGC | 13325610 |
| 128 | GGAAAGAAAGAAAGAAAGAAAGAAAGAAAGAAAGAAAGAAAGAAAGAAAGAAAGAAAGAAAGAAAGAAAGAAAGAAAGAAGAAAGAAAGAGAAAGAAAGAAAGAAAGAAAGAAAGAAAGAAAGAAAGAAAGAAAGAAAGAAAGAAAGAAAAGAC | 13344310 |
| 129 | AAGAAAGAAAGAAAGAAAGAAAGAAAGAAAGAAAGAAAGAAAGAAAGAAAGAAAGAAAGAAAGAAAGAAAGAAAGAAGGAAGGAAGGAAGGAAGGAAGGAAGGAAGGAAAGAAAGGAAAGAAAGAAAGAGAAAGAAAGGAAGAAAGAAAGAAAGAAAGAAAGAAAGAAAGAAAGAAAGAAAGAAAAAGAAAGAAAGAAAGAAAGAAAGAAAGAAAGAAAGAAAGAAAGAAAGAAAGAAAGAAAGGAAGGAAGGAAGGAAGGAAGGAAGGAAGGAAGGAAGGAAGGAAGGAAGGAAGGAAAAGAAAGAAAGAC | 13356770 |
| 130 | AGAGGAAGGAAGGAAGGAAGGGAGGGAGGAAGGAAGGAAGGAAGGGAGGGAGGGAGGGAGGGAGGGAAGGAGGGAGGGAGGGAGGGAAGGAAGGAAAT | 13514678 |
| 131 | GAAAGAAAGAAAGAAAAGAAAGAAAGAAAGAAAGAAAGAAAGAAAGAAAGAAAGAAAGAAAGAAAGAAAGAAAGAAAGAGAGAGAGAGAAAC | 13514776 |
| 132 | AAGAAAGAAAGAAGGGAGAGAGAGGGGGAAAGAAAGAAAGAAGGGAGGGAGAGAGGGAAAGAAGGAAAGAAGAAAGAGAGAGAAAGAGAAAGC | 13546407 |
| 133 | GGAAAGAAAGAAAGAAAGAAAGAAAGAAAGAAAGAAAGAAAGAAAGAAAGAAAGAAAGAAAGAAAGAAAGAAAGAAAGAAAGAAAGAAGAAAGAAAGAGAAAGAAAGAAAGAAAGAAAGAAAGAAAGAAAGAAAGAAAGAAAGAAAGAAAGAAAGAAAGAAAAGAC | 13565153 |
| 134 | AAGAAAGAAAGAAAGAAAGAAAGAAAGAAAGAAAGAAAGAAAGAAAGAAAGAAAGAAAGAAAGAAAGAAGGAAGGAAGGAAGGAAGGAAGGAAAGAAAGGAAAGAAAGAAAGAGAAAGAAAGGAAGAAAGAAAGAAAGAAAGAAAGAAAGAAAGAAAGAAAGAAAGAAAGAAAGAAAGAAAAAGAAAGAAAGAAAGAAAGAAAAGAAAGAAAGAAAGAAAGAAAGAAAGGAAGGAAGGAAGGAAGGAAGGAAGGAAGGAAGGAAGGAAGGAAGGAAGGAAAAGAAAGAAAGAC | 13577625 |
| 135 | GAAGGGGGGAGGGGAGGGGAAGGGAGGGGAGGGGAGGGGAGGGGAAGGGAGGGGAGGGGAAGGGAGGGGAGAGGGAAAGAAAGAAGAGAC | 13733350 |
| 136 | AGAGAGAAAGAAAGAGAAAAGAAGAAAAGAGAAAGAAGAAAGAAAGAAAAGAAAAGAAAAAAGAAAAAGAGAAAGAGAAAGAAAAGAGAAAC | 13733440 |
| 137 | AAAAGAAAAAAGAAAGAAAGAGAAGGAAAGAAAGAGAGAAAAAGAGAGAGAGAAAGAAAGAAAT | 13883587 |
| 138 | AAAAAAAAAAAAAAAGAAAGAAAGAAAGAAAGAAAGAAAGAAAGAAAGAAAGAAAGAAAGAAAGAAAGAAAGAAAAT | 14362034 |
| 139 | AAAAAAAAAAAAAAAAAAAAAGAAGAAGAAGAAGAAGAAAGGGGAGAAGAGGAGAAAC | 14483788 |
| 140 | AAAAAAAAAAAAAAAAAAAAAAAAAAAAAAAAAAAAAAAAAAAAAAAAAAAC | 14646663 |
| 141 | AGAAAGAGAGAGAGAGAGAGAGAGAGAAAGAGAGAAAGAAAGAGAGAGGGAT | 14680250 |
| 142 | AGAAGGAAGGAAGGAAGGAAGGAAAAGAAGGAAGGAAGGAAGGAAGGAAGAAAGGAAGGAAGGAAGGGT | 14706893 |
| 143 | AAAAGAAAGAAAGAAAGAAAGAAAGAGAGAGAGAGAGAGAGAGAGAGAAAGAAAGAAAGAAAAGAAAGAAAGAAAGAAAGAAAGAAAGAAAGAAAGAAAGAAAGAAAGAAAGAAAGAAAGAAAGAAAGAAAAGC | 15111965 |
| 144 | AAAAAAAAAAAAAAAAAAAAAGGAAGGAAGGAGGAGGGAGGGAGGAAAGAAAAGAAT | 15401057 |
| 145 | GGAAAGGAAGGAAGGAGAGAGAGAAAGAAGAAAAAAGAAAGAGAAGAAGGAGAGAGAGAGAGAC | 15401114 |
| 146 | AAAAAAAAAAAAAAAAAAGAGAGAGAGAAGAAAGGGAAGAGGAGGGGAGGGGAGGGAAGGGAAC | 15434898 |
| 147 | AGAAAGGGGAAGGAAGGGAGGGAGAGAAGGAAAGGAAGGGAAGGGAAGGGAGAAGGGAGGGAAGGGAAGAAGGAAAGGAAGGAAGGAAAGGAAGGGGAAGGGGAAGGGAGGAGAAGGAAAAGGAAGGGAAGGGAAGGGAGGGGAGGGAAGT | 15451344 |
| 148 | AAAAAAAGGGAAGGAAGGAAGGAGGAGGGGAGGGGAGGGGAGGGAGGGGAAGGGGAAGGAAGGT | 15453842 |
| 149 | AAAAAAAAAAAAAAAAAAAAAAAAAAAAAAAAAAAAAAAAAAAAAAAAAGAC | 15687577 |
| 150 | GGAAAGAAAGAAAGAGAGAAAGAGAGAGAGAAAGAGAGAAAGAGAGAAAGAGAGAGAGAGAGAAAGAGAGAGAGAGAGAAAGAGAGAAGGAGGT | 15974182 |
| 151 | AAAAAAAAAAAAAAAAAAAAAAAAGAAAGAAAGAAAGAAAAGAAAAAAGAAAAAGT | 15983665 |
| 152 | GGAAAGAAAGAAAGAGAGAAAGAGAGAGAGAAAGAGAGAAAGAGAGAAAGAGAGAGAGAGAGAAAGAGAGAGAGAGAGAAAGAGAGAAGGAGGT | 16131045 |
| 153 | AAAAAAAAAAAAAAAAAAAAAAAAGAAAGAAAGAAAGAAAAGAAAAAAGAAAAAGT | 16140528 |
| 154 | AAGGGAAGGAAAGGAGAGAGAAGAAGGAAGGAAGGAAGGGAGAGAGGGAGGGAGGGAGGGGC | 16336813 |
| 155 | GGAGAGAAAAAGAGGAGGGAAGGGAAGGAAGAGGGAGGAGAGGGAGGGAGGGAGGGAGGGAGGAAGGGAGGGAGGAGAGGGAGGGAGGAAAGGAGGAAGC | 16345001 |
| 156 | AAAGAAAGAAAGAAGGAAAGAAGGAAAGAAAGAAAGGAAGGAGGAAGGAAGGAAGGAAGT | 16475354 |
| 157 | AAAAAGAGGAGAGGAGAAGGGAGAGGAGGAGAGGGGAGGAGGGGAAGAGAAGC | 16484509 |
| 158 | AGAAGAAGAAAGAAGAAGAAAAGAAGAAAAGAAGAAGAAAGAAGAAGAGGAAGAAGAAGAAGAAGAAGAAAAGAAGAAGAAGAAGAAGAAGAAGAAGAAGAAGAAGAAGAAGAAGAAGAAGAAGAAGAAGAAC | 16522175 |
| 159 | AAGAAAAAAGAAGGGAAGGAAGGGAGGGAGGGAGGGAGGAAGGAGAAGGAAGGAGAAGGAAGGAAGAAAT | 16576818 |
| 160 | GAAGGAAGGAAGAAGGAAGAAAGGAAGGAAGGAAGAAAGGAAGGAAGGAAAGAAGAAAGGAAGGGAGGGAT | 16576888 |
| 161 | AGGAAAGGAGGGAGGAAGGGAAGAAAGAAAGAAAGAAAGAAAGAAAGAAAGAAAGAAAGAAGGGAAGAAAAAGAAAGAGAGAGAGAAAGAGAGAAAGGGAGAAAGAAAGGGAAAGGGAAAAGGAGAGAGAAAGGGAGAAAGAAAAGAAGAAAGGAAAGAAAGAAAGAAGGGGAAGGAAGGAAGGAAGC | 16580197 |
| 162 | AAAAAAAAAAGAAAGAAAAGAAGAAAGAGAGAGAAAGAAAGAAAGAGAAAAGAAAAC | 16698799 |
| 163 | AAAGAAAGAAAGAAAGAAAGAAAGAAAGAAAGAAAGAAAGAAAGAAAGAGAAAAGAAAGAAAAC | 16698856 |
| 164 | AAAAAAAAAAAAAAAAAGAAAGAAAGAAAGAAAGAAAGAAAGAAAGAAAGAAAAT | 16736275 |
| 165 | GAAGAGAGGGGAGGAGAAGGGAGAGAAAGGGAGGGAGGAGAGAGAAGAGGGAAT | 16876922 |
| 166 | GAGAGAGAAAGAAGAAGAAAAGAAGAAAGGAAGAAAGGAAAGAAGGGAGGGAAGGAGGGAAGGAGGAAGGAAGGGAGGGAT | 16992646 |
| 167 | GAAGAAAAAGAAGAAGAGGAGGAAGAAGAAGAAGGAGGAAGAGGGGGAAAAGAGGAGGAGAT | 17156921 |
| 168 | AGAAGAAAGGAGGAGAGGAGAGGGGAGGGAGGGGAGGAGAGGAGGAGAGGGGAGGGAGGGGAGGAGAGGAGGAGAGGGGAGGC | 17602530 |
| 169 | GAAGGAAAGAAGGAAGGGAGGGAGGGAGGGAGGAAGGGAAGGAAGGAAGGAAGGAAAGAAGGAAGGAAGGGAGGGAGGGAGGAAGGGAAGGAAGGAAGGGAGGGAGGGAGGGAGAGT | 17620663 |
| 170 | GGGAGGGAGGAAGGGAAGGAAGGAAGGAAGGAAGGAAGGAAGGAGGGAAGGAAGGAAGGAGGC | 17620780 |
| 171 | AAAAAAGAGAGAGAGAAAGAAAGAAAAAAAGAAAGGAAGGAAGGAAGAGGAAGAAAGAGAAAAGAAGGAAAGAAAGAAGAGGC | 17622295 |
| 172 | AAAAAAAAAAAAAAAAAAAAAAAGGAAAGGAAGGAAAGAAGGAAAGAAGGAAAGAAAGAAAGAAAAT | 17699030 |
| 173 | GAGAGGAAAGAAAGAAGGAAGGAAGGAAAGAGAGAAAAGAAAGAGAAAGAAGGAAAGAAAGAGGGAGAAAAAAAC | 18392735 |
| 174 | GAGAAGGAAGGAAAGAGAGAGAAGGAAAGAAGAAGGAAAGAGAAAGAAAGGAAAAGAAAAAAAAAGAAC | 18392873 |
| 175 | AAAGAGAGAAAGAGAAAGAAGAAAGAAAGAAAGAGAAAGAAGGAAAGAAAGAAAAC | 18394499 |
| 176 | AGGAAGGAAGGAAGGAAGGAAGGAAGAAAGAAAGAAAGAAAGAAAGAAAGAAAGAAAGAAAGAAAGAAAGAAAGAAAGAAAGAAGGAGAGGGAGGGAGGGAGGGAGGGAGGAGC | 18501350 |
| 177 | AAGAAGGGGAGAGAGAGAGAGAGAGAGAAAGAGAGAGGGAGAGGAGAGAGGAGGAGAGAGAGAAT | 18590663 |
| 178 | AAAAAAAAAAGAAAAAGAAAAGAAAGAAAGAGAGAGAGAGAAAGAGAAAGAAAAAGAAAGAAAGAAAGAAAGAAAGAAAGAAAGAAAGAAAGAAAGAAAGGAAGGAAGGAAGGAAGGAAGGAAGGAAGGAAGGAAGGAAGGAAGGAAGGAAGGAAAAGAGAAAGAAAGAAAGAAAGGGAAGGAAGGAAGGAAGGAAGGAAGGAAGGAAGGAAGGAAGGAAGGAAGGAAAAGC | 18744817 |
| 179 | AAAAAAGAAAGAGAAAAAGAAAGAAAGGGAGAAAGGGAGAGAGAGAGAGAGAGT | 18864020 |
| 180 | AAGAAGGAGGGAAGGAAGGAAGGGAGGGAGGGAGGGAAGGGAAGGGAGGGAGGGAGGGAGGAAAGAAGGGAGGGAGGAAAGAAGGGAGGGAGGGAGGAAGGAAGGAAGGAAGGAAGGAAGGAAGGAAGGC | 18864075 |
| 181 | AAAGAAAGAAAGAAAGAAAGAAAGAAAGAAAGAAAGAAAGGAAGAGAGAGAGAGAGAGAGAGAGAGAGAGAGAAAGAAAGAAAGAAAGAAAGAAAGAAAGAAAGAAAGAAAGAAAGAAAGAAAGAAAGAAAGGGT | 18888417 |
| 182 | GAAGGAGGGAGGGAAGGAGGGAGGGAGGGAGGGAAGGAAAGAAGGAGGGAAGGAAGGAAGGGAGGGAAGGAGAAAGAAC | 18889377 |
| 183 | AAAAGAAGGAAGGAAGGAAGGAAGGAAGGAAAGAAAGAAAGAAAGAAAGAAAGAAAGAAAGAAAGAAAGAAAGAAAGAAAGAAAGAAAGAAAGAAAGAAGGAAGGAAGGAAGGAAGGAAGGAAGGAAGGAAGGAAGGAAGGAAGGAAGGGGAAGGGAAGGGAAGGGAGGAAGGAAGGAAGGGAGGAAGGAAGGGGAAGGAAGGAAGGAAGAAGGGAGGAAGGGAGGGAGGGAGGGAAGGAAGGAAGGAAGGAAGGAAGGAAGGAAGGAAGGAAGGAAGGAAGGAAAGGC | 18925340 |
| 184 | GAAAAAAAAAGAAAAAGAAAAGAAAAGAAGAGAAAAAAAGAAAGAAGGAAAGAAAGAAAGGAAAGGAAAGGAAGAAAGGAAGAAAAGAAGT | 18937231 |
| 185 | GAGGGAAAAGAAAGGAAAGAAGAAGAAGGGAGGGAAGGAGGGAAAGAAAGAAGT | 18954112 |
| 186 | GGAAGGAGGAAGGAAGGAAGAAAGAAAGAAAGGAAAGAAGGAAGGAGGGAGGGAAGGAAGGGAGGAAAAGAAGGAGAGAAGGAGGGAAGGGAGGAAGGGAGC | 18954180 |
| 187 | AAAAAAAAAAAGAAAAAAAAAAAAAGAAAAGAAAGAAAGAAAAAGAAAAAAAGAAC | 18982003 |
| 188 | GAAAGAAGGAAGGAAGGAAGGAAAGAGAGAGAGAAAGAAAGGAGGGAGGGAGGGAGGGAGAAAGGAAGGAAAGAAAGGGAGAGAAGGAGGAAGGAAGGAAGGAAGGAAGGAAGGAAGGAAGGAAGGAAGGAAGGAAGGAAGGAAGGAAGGAAGGAAGGAAAGAAAGAAAAAAAGGC | 18996715 |
| 189 | AAAAAAAAAAAAAAAGGAGAAGAAGAAGAAGAAGAAAAAAGAAAAGAAAAAT | 19009773 |
| 190 | AGAGAGGAGGAGAAAAGGAGGAAGGAGAAGAAGAGAAGGAGAAAAAGAGGAGAC | 19011691 |
| 191 | GGAAGGAAGGAAGGAAGGAAAAAAGAAGGAGAGAAGGAAGGAGAAAGGAAGGAAGGAAGGAAGGAAGGAAGGAAGGAAGGAAGGAAGGAAGGAAGGAAGGAAGGAGC | 19052878 |
| 192 | GAAGAAGGAGAAGGAGGAGGAGGAGGAGGAGGAAGAGGAGGAGGAGGAGGAGGAGGAGGAGGAGGAGAAAAGGAGAAGAAGAAAAAT | 19144746 |
| 193 | GGAAGGAAGGAAGGAAGGAAGGAAGGAAGGAAGGAAGGAAGGAAGGAAGGAAGGAGGGAGGGAGGGAGGGAGGGAGGGAGGGAAGGAAGGAGGGAAGGAGGGAAAAAGGGAGGAAAAGAGAGAGGGAGGC | 19173370 |
| 194 | AGGGGAGGAGGGAGGGAGAGAAAGGGGGAGGGAAAGAAAGAGGGAGGGAGAGAAAGAGAGAGGAAGAGAAGGGGGAGGGAGGGAGGGAGGGAT | 19173500 |
| 195 | AAAAAGAAAGGAAAGAAAGAAAGAGAGAGAGAGAGGAAGGAAGGGAAGGAAAT | 19360066 |
| 196 | GAAAGAAAGAAAGAAAGAAAGAAAAAGAAAGAAAGAAAGAAAGAAAGAAAGAAAGAAAGAAAGAAAGAAAGAAAGAAAGAAAGAAAGAGGGAGGGAGGGAGGAAGGAAGGAAGGAAAGAGAGGAAGGAAGGAAAGAAGGAAGGAAAAGAAAGAGAGAGAGAGGAAGAAAGAAAGAAAGAGAAAGAAGAAAGAAAAAGAAAGAGAAAGAAGGAAGGAAGGAAGGAAGGAAAAGAAAGAAAGAAAGAGAAAGAAAGAAAGAAAGAAAGAAAGAAAGAAAGAAAGAAAGAAAGAAAGAAAGAAAGAAGAAAGAAAAAGAAAGAAAGAGAAAGAAGGAAGGAAGAGAGAAAGAAAGT | 19367414 |
| 197 | GAAGGAAGGAAGGAAGGAAGGAAGGAAGGAAAGAAGGAAGGAAGGAAGGAAGGAAGGAGGT | 19373282 |
| 198 | AAAAGGAAAGAAAAGAAAGAAAGAAAGAGAGAGAGAGAGAGAGGGAGGAAAGAAAGAAAGAAAGAAAGAAAGAAAGAAAGAAAGAAAGAAAGAAAGAAAGAAAGAAAGAAAGAAGGAAGGAAGGAAGGGGGGAAGGAAGGAAGGAT | 19374141 |
| 199 | AAAAAAAAGAAGAAGAAGGAGAAGAAGGAGGAGAAGAAGAAGAAGGAGAAGAAGAAGGAGAAGAT | 19385415 |
| 200 | GGAGAAGAAGAAGAAGAAGAAGAAGAGGAAGAAGAAGAAGAAGAAGAAGAAGAAGAAGAAGAAGAAGAAGAAGAAGAAGAAGAAGAAGAAGAAGAAGGAGGAGGAGGAGGAGGAGGAGGAGGAGGAAAGGGGAGGGGGAGGGGAGC | 19385480 |
| 201 | AAAAAAGAAAGAAGGAAGGAAGGAAGGAAGAGAAAGAAAGAAGGAAAGAAAGAAAGAAAGAAAGAAAGAGAGAGAGAGAGAAAGAAAGAAAAGAAAAGAAAAGAAAAGAAAAGAAAAGAAAAGAAAGGGGC | 19406339 |
| 202 | AGAAGGGAAGGAGGGAGGGAGGGAGGAGAGAGGGAGGGAGGAGAGAAGGAGGGAGGGAGGGAGGAGAGAAGAAGC | 19415704 |
| 203 | GGGGGAAGGAAGGAAGGGAGGGAGGGAGGGAGGGAGGGGAGGGGAGGGGGAGAGAGAGAGAGAAAGAAAGC | 19416205 |
| 204 | AAGAAGGAAAGAAAGAAGGAAGGAAAGAAAGAAGAAAGAAAGAGAGAGAGGGAGGGAAAGAGAGAT | 19416276 |
| 205 | AAGAAGGAAAGAAAGAAGGAAGGAAGGAAGGAAGGAAAGAAAGAAGGAAGGAAGGAAGGAAGGAAGGAAGGAAGGAAAGAAAGAAAAAGAAAGAAAGAAAGAAAGAGAAAAAGAAAGAAAAGAAAGAAAGGAAGGAAGGAAAGAAAGAGAGAGAGAGAAAGAGAGAAGAGAAAGAGAGGGAGGGAGGAAGGAAGGAAGGAAAGAGAGAGAAAGAAAGAAAGAAAGAAAGAAAGAAAGAAAGAAAGAAAGAAAAAGAAAGAGGAT | 19416367 |
| 206 | AAAAGGAAAGAAAAAGGAAGGAAGGAAGGAAGGAAGGAAGGAAGGAAGGAAGGAAGGAAGGAAGGAAGC | 19433905 |
| 207 | GAGAGAGAGAGAGAAAGAAAGAAAGAGAAAAGAAAAAAAGAAAAGAAAAGAAAGAAGGAAGGGAAAGGAAAGGAAGGAAGGAAGAAAAAAGAAAGAAAGGAAGGAAGGAAGGAAGGAAAGAAAGGAAGGAAGGAAGGAAGGC | 19528625 |
| 208 | AGGAAGGAAGGAAGGAAGGAAGGAAGAAAGAAAGAAAGAAAGAAAGAAAGAAAGAAAGAAAGAAAGAAAGAAAGAAAGAAAGAAAAT | 19528767 |
| 209 | GAGAGAAAAAAGAAAGAAAGAAAGAGAGAAAGAGAGGGAGGGAGGGAGGGAAAGAAAAT | 19895473 |
| 210 | AAAAAAAAAAAAAAAAAAAAAAAAAAAAAGAAAGAAAAGGGGGGAGGAAAGGAAGGGGAAGGGGAAGAAGAGAAAGGAAGGAAGAAAAAAGAAGC | 20045091 |
| 211 | AAAAAAAAAAAAAGAGAGAAGAAAGAAAGAGAGAGAGAGAGGGAGGGAGAGAGGGAGGGAGGGAGGGAGAGAGAGAGAAAGAGAGAGAAAGAGAGAGAAAAGAAAAGAAAAGAAAAGAAAAGAAAAGAAAAGAAAAGAAAAGAAAAGAAAAGAAAAGGAGAAAGAAGAAAAT | 20183391 |
| 212 | AAAAAAAAAAAAAAAAGAAGAAGAAAGGAAGGAAGGAAGGAAGGAAGGAAAGAAGGAAAGAAAAAAGGAAAAAAGGAAT | 20337452 |
| 213 | AGAAAAGAAAGAAAGAAAGAAAGAAAGAAAGAAAGAAAGAAAGAAAGAAAGAAAGAAAC | 20455190 |
| 214 | AAAAAAAAAAAAAGAAAGAAAGAAAAGAAAGAGAAAGAAAGAGAGAAAGAAAGAAAAAAGAAAGAGAGAAAGAGAGAGAGAAAAAGAAAGAAAGAAAGAAAGAAAGAAAGAAAGAAAGAAAGAAAGAGAAAGAAAGAAAGAAAGT | 20753668 |
| 215 | AGAAAGAAAAGAAAGAAAGAGAGAAAGAGAGAGAGAGAGGGAGGGAGGGAGGGT | 20819159 |
| 216 | GGGGGAGGGAGGGAGGGAGGGGGAGGAAGGGAGGGAGGGAGAGAGGAAGGAAGGAAGGAAGGAAGGAAGGAAGGAAGGAAGGAAGGAAGGAAGGAAGGAGT | 20819213 |
| 217 | AAAAAAAAAAAAAAAAAAAAAAAAGAAGAGGAAAAAGAAAAAAAGAAAGAAGAAAGAAAT | 20891112 |
| 218 | AAAAAAAAAAAGAAAAAGAAAAAGAAAAGAAAAGGAGAGGAAAGAAAGGAAAAAAAAT | 20892379 |
| 219 | AAAAAAAAAAAAAAAAGAAGGAAAGAGAAGAGGGAGGAAGGAGGGAGGGAGGAGAGGGGGAAAGGAAGGAAGGAAAGAAGGAAGGGAAGGAAGGAAGGAAC | 20898874 |
| 220 | AAGGAGAGGAGGGGAAGGGAGGGAAGGGGAGGGGGAGAAGGGAAGGGAAGGGAAGGAAAGGGAAGGGGAGAGGAGAAGGGAAGGGAAAGGAAGGAAAGGAAAGGGAAGGGGAGGAGAGAGGAAGGAGGAAGGAAGAAAGAAAGAAAGAAAGAAAAGAAGAGAAAGT | 20959577 |
| 221 | AGAAAAAAAAAAAAAAAGAAAGAAAGAAAAAGAAAAAGAAAGGAAAGGAAAC | 20959898 |
| 222 | AAAAAGAAAGGAGAGGGGAGAGGAGGAGAAAGGAGGGGAGGGGAGGGGAGGGGAGGGGAGGGAAGGC | 20987648 |
| 223 | GGAAGAAAAGGAAGGGAGGAAGGAAGGAAGAAAGGAAGAAAGGAAGGAAGGAAGGGAGGAAGGAAGGAAGGAAGGAAGGAAGGGAGGAAGGAAGGAAGGAAGGAAGGAAGGAAGGAAGGAAGGGAGGAAGGAAGGAAGGAAGGAAGGAAGGGAGGAAGGAAGGAAGGAAGGGAGGAAGGAAGGAAGGAAGGAAGGAAGGGAGGAAGGAAGGAAGAAAGGAAGGGAGGGAAGGAC | 21011487 |
| 224 | AAAAAGAGAGGAGAGGAGAGGAGGGGAGGGGAGGGGAGGGGAGGGGAGAGGAGAGGAGAGGAGAGGAGAGAT | 21374567 |
| 225 | AAAAAAAAAAAAAAAAAAGAAAAAGAAAAAGAAAGAAAAAGAAAAGAAAAAAGAAAAC | 21395949 |
| 226 | AAAGGAAAGGAAGAAGGAGAAGAAGGAGAAAGAGAAGGAAAGGAAAGGAAAGGGGAAAAGGAAAAGAAAGAAAAC | 21528856 |
| 227 | GAGGGAGGGGAGGGGAGGGGAGGGGAGGGGAGGGGAGGGGAGGGGAGGGGGAGAAAGAT | 21718237 |
| 228 | AGAAAGAAAGAAAGAGAGAGAAAGGAAGAAGAAGAAGAAGGAGGAGGAGGAGGAGGAGGGAAAAGGAAGGAAGGAAAGAAGGAAGGAAGGAGGGAAGGAAGGAAGGGAGGGAGGGAGGGAGGGAGGGAGGGAGGAAGGGAGGGAGGGAGGGAGGAAGGGAAAGC | 22022341 |
| 229 | AAAAAAAAAAAGAAAAGAAAAGAAAAAGAAAAAGAAAAAGAAAAGAAAAGAAAAGAAAAAAAGAAAAAGAAAAC | 22128073 |
| 230 | AAAAAAAAAAAAAAAAAAGAAGAAGAAGAAGAAGAAGAAAAAGAAAAAAGAAAAGAAAC | 22331498 |
| 231 | AAAAAAAAAAAAAAAAAGAAAGAAAGAAAGAAAGGAAGAAGGGAAGAAAGAAAAAAAAGAAAGAAAGAAAGGAAGAAGGGAAGAAAGAAAAAAAAGC | 22665444 |
| 232 | GGAAGGAAGGAAGGAGAAAGGGAAAGAAGGAAGAGAGAAAGGAGGGAGGGAGAAAGGAAGGAAGGAGT | 22716422 |
| 233 | AAAAAAAAAAAGAAAAAAGAAGAAGAAGAAGAAGAAAGAAGGAGAAGGAGAAGAAGAAGAGGC | 22732569 |
| 234 | GAAAGAAAAGAAAGAGAGAGAGAGGAGAGAGAGAGAAAGAGAGAGAGAGGAAGGAAGGAAGGAAGGAAGGAGAAAGAAAGGAAGGAAGGAAGGAAT | 22745908 |
| 235 | GAAGGAAGGAAGGAAGGAAAGAAGGAAGGAAGGAAGGAAGGAAGGAAGGAAGGAAGAT | 22746044 |
| 236 | AAGAAAGGGGAAGGAAGGAAGGGAGGAAAGAAAGAAAAAGAAAGAAAGAAAGAAAGAAAGAAAGAAAGAAAGAAAGAAAGAAAGAAAGAGAAAGAAAGAAAGAAAAAGGGAGGAAGGGAAGGGAGAGAAAGGAAGGGAAGGGAGGGGAGGGGAGGGAAGGGGAAGGGAGGAGC | 22779748 |
| 237 | GGAGAGAGGGAGGGAGGGAGGAAGAAGGGAAGGAAGGAAGGGAGGGAGGGAAGGAAGGAAAGGAGGGAGGGAGGAAGGAGGGAAGGAAGGAAAGGAGGGAGGGAGGAAGGAGGGAAGGAAGGGAGGGAGGGAAGGAAGGAGGGAGGGAGGAAGGAGGGAAAGAAGGGAGAGAGGGAGGAAGGGGGAGGGAAAAGAAAGGAGGGAAGGAAGGAGAAAGAAGAAAGGAAGGGAGGGGAGGGAAGAGGAGAGGAGGGGAGGGAAGGGAGGAAGGGAAGGAGGGAGGGAGT | 22985127 |
| 238 | GGAGGGAGGAAGGAAGGGAGGGAGGGAGGAAGGAGAGAAGGAGGAAGGAAGGAGGGAAGGAGAGAAGGAAGGAGGAGGGAGGGAGGGAAGGAGGGAGGC | 22985597 |
| 239 | GAGGAAAGGAAGGAAGGGAAGAGGAAAAGAAGGGAGGAAGGAAGAAGGGAAGGGAGGAGAAAGGAGAAGGAAGT | 22985813 |
| 240 | GGGGGAAGGAAGGGAGGGAGGGAAGAAGGAAGGAAGGAAAGAAAGAAAGAAGGAAAGAAAGAAGAAAAGAAGAAGAAAGGC | 23209789 |
| 241 | GAAAGAAAGAAAGAGAGAAAGAGAGAGAAAAAAAAAAAAAGGAAGGAAGGAAGGAAGGAAAGAAGGAAGGAAGC | 23363011 |
| 242 | AAAGAGAGAAAGAGGGAAGGAGAGAAAGAAAAGAAAGAAAGAAGGAAAGAAGGAAGGAAGGAAGGAAAGAAAAAGAGAAAGAAAGAT | 23363395 |
| 243 | GAAAGAAAGAAAGAAAGAAAGAAAGAAAGAAAGAAAGAAAGAAAGAAAGAAAGGAAGGAAGAAAGAAAAAGAAAGAGAAAGGAAGGAAGAAAGAGGT | 23363482 |
| 244 | GGGAAGGAGGGAGGGAGGAAGAGAGGGAGAGAGGGAGGGAGGGAGGGAGGGAGGGAGGGAGGGAGGGAGGGAGAGAGGGAGAGAGGGAGGGAGGGGGGAAGGAGGGAGGGAGGGGGGGAAGGAGGGAGGGAGT | 23489339 |
| 245 | AAAGAGAAGAAAAAAGAGAAAAGAAAAGGAAAAGAAAAGAAAAGAAAGAAAAGT | 23502129 |
| 246 | AAAAAAAAAAAAAAAAAAAAAAAAAAAAAAAAAAAAAAAAAAAAAAAAAAAAAAT | 23614146 |
| 247 | AGAGAGAGAGAAGAAAAGGAAAGAAAAGGAAAAGAAAGGAAGAAGGAAAGAAAGAAAT | 23731665 |
| 248 | AAGAAAGAAAGGAAGAAGGGAAAGGGAAAGGGAAAGAAAGAGAGAGAGAAAGAAAGAT | 24173852 |
| 249 | AGAAAGAAAAGAAGAAAGAGAGAAAGAAGGAAGGAAGGAGAAAGAAAGGAAGGAAGGAAGGAAAGAAGGAAGGAAGGAGAAAGAAAGGAAGGAAGGAAGGGAGGGAGGGAGGGAGGAAGGGAGGAAGGAAGGGC | 24211405 |
| 250 | AAAAAAAAAAAAAAAAAGAAAAGAAAAAAGAAAAAAAAAGGAAAAAGAAGGGAGGGAGGGC | 24211823 |
| 251 | AAAAAAAAAAGAAAGAAAGAAAGAAAGAAAGAAAGAAAGAAAGAAAGAAAGAAAGAAAGAAAGAAAGAAAGAAAGAAAGAAAGAAAGAAAGAAAAGGAAAGAAAAC | 24214999 |
| 252 | AAGGAAGAAAGAAAGGAAGGAAGGAAGGAAGGAAGGAGGAAGGAAGGAAGGAAGGAGGAAGT | 24411605 |
| 253 | AGAAAGAAAGAAAGAAAAGAAAAAAAAGAAAGAAAAGAAAGAAAGAGGGAAGGAAGGAAAGAGAGAGAGAGAAAGAAAGAAAGAGAAAGAAAGAAAGAGAAAGAAAGAAAGAGAAAGAAAGAGAAAGAAAGGAAAGAGAAAGAAAGGAAAGAAAGAAAGAAAGGAAAGAAAGAAAGAAAAGAAAGAAAGAAAGGAAGGAAGGT | 24442200 |
| 254 | AGGAGAAGGAGGAGGAGGAGGAGGAAGAGGAGGAGGAGGAAGAGGAAGAAAAAGAAGAGAGGGC | 24840376 |
| 255 | AAAAAAAAAGAAAGAAAGAAAAGAAGAAAGGAAAGAAAGAAAGAAAGAAGGAAGGAAGGAAGGAAGGAAGGAAAGAAGGAAAGAAAGAAAGAGAGAGAGAAAGAAAGAAAAAGAGAGAGAGAAAGAGAT | 24841079 |
| 256 | AAAGAAAGAAAGAAAAAAAGAAAGAGAAAGAAAGAGAAAGAAAGAAAAAGAAAGAT | 24841208 |
| 257 | AAAAAGAAAGAGAGAGAGAGAGAGAGAGAGAGAGAGAGAGAGAGAGAGAGAGAC | 24970640 |
| 258 | GGGGGAGGGAGGGAAGGAAGGAAGGGGAAAGGAAAGGAAGGAAAGGAAGGAAGAAAGGAAGGGAAGGAAAGGAAAGAAGGAAAGGAAGGAAGAAAGGAAGGGAAGGAAGGGAAGGAAGGGAAAGAAAGGAAAGAAAGGAAGAAAGAAAAT | 25029954 |
| 259 | AAAAAAAGAAAGAGAAGGAAGGGGGGGGGGGAGGGGGAGGGGGAGGGGGAGGAGAAGGGGGGGAGGT | 25139890 |
| 260 | AAAAGAGAAGAAAGAAGAAAGAAAGAAAGAAAGAAAGAGAAAGAAAGAAAGAAAGAAAGAAAGAAAGAAAGAAAGAAAGAAAGAAAGAAAGAAAAGAAAAAGAAAGAAAGAAAGAGAAAGAAGGAAAGAT | 25139971 |
| 261 | GGAGGAGGGGAGGGGGAGGGGGGAGGGGGGAGGGGGAGGGGGAGGGGAGGGGGAGGGGAGGGGGAGGGGAC | 25272702 |
| 262 | GGAAGGAAGGAAGGAAGGAAGGAAGGAAGGAAGGAAGGAAGGAAGGAAGAGGGAAAGGGAAAGGGAAAGGGAAAGGGAAGAAAT | 25517553 |
| 263 | GAAGGAAGGAAGAAGGAAGGAAGGAAGGGAGGAAGGAAGGAAGGAAGGAAGGAAGAAGGAAGGAAGGAAGGAAGAAGGAAGAAGGGAGGGGAAGGGAAGGGAT | 25587287 |
| 264 | AGAAGGAAGGAAGGAAGGAAGGAAGGAAGGAAGGAAGGAAGGAAGGAAGGAAGGAAGGAAGT | 25616867 |
| 265 | AGAGGGAGAGAGAAGAGAGAAAGAGGAGAGAGAGAAAAAAAAGAGAAGAGAGAGAAAGT | 25697004 |
| 266 | GGAAGGAAGGAAGGAAGGAAGGAAGGAAGGAAGGAAGGAAGGAAGGAGGGAGGGAGGGAGGGAGGGAGGGAGGGAGGGAGGAAGGAAGGAAGGAAGGAAGGAAGGAAGGAAGGAAGGAAGAAAGGAAGGAAGGAAGGAAGGAGGGAAGGGAGGGAGGGAGGGAGGGAGGAAGGAAGGAC | 25709148 |
| 267 | GGAAGGGAGGGAGGGAGGGAGGGAGGAAGGAAGGAGGGAAGGGAGGGAGGGAGGGAGGGAGGAAGT | 25709327 |
| 268 | AGAAAAGAAGAGAAGGAGGAGAAAAGGAAGGAAGGAAGGAGAGAGGGAGGGAAGGAAGAT | 25711831 |
| 269 | AAAAAAAAAAAAAAGAAAGGAAAGAAAGAAAAAGAAAAAAAAAAGAAAGAAAT | 25746120 |
| 270 | AAGAAAGAGAGAGAGAAAGAAAAAAGAAAAGAAAAGAAAAGAAAAAGGGAAGAAAGAAAAGGAAAGAGAAGAAAGAAAAGGAAAGAAAAGAAAAGAAAAAT | 25753860 |
| 271 | AGAAAAAGGAAGGAAGGAAGGGGAAAGAAAGAAAGAAAGAAAGGAAAAGAAAGAAAGGT | 26080926 |
| 272 | GGAAAGGAAAGGAAAGGGAAAGGGAAAAGAAAGGAGGGGAGGGAAGGGAGAGAGAAAGAAGGAAGC | 26358067 |
| 273 | AAAAAAAAAAAAAAAAAAAAAAAAAAAAAAAAAGAAAGAAAAAAGAAAAAAAAGAAAC | 26435462 |
| 274 | AAGAAGGAAGGAAGGAAGAAAGGAAGGAAGGAAGGAAGGAAAGAAGGAAGGAAGGAAGAGAGGGAGGGAGGGAGGGAGGAAGGGAAGAGGGAGGGAAAGAAAGAGAAAGAAAGGAGAAAAAGAGAAAGAGAAAAAGGAAGGAAGGAGGGAAGGAAGAAGGAGGAAGGAAGGAAGGAAAAGAGT | 26436818 |
| 275 | GAAAGGAAAGGAAAGGAAAGAAGGAAGGAAGGAAGGAAGGAAAGAAAGAAAGAAAGAAAAAAGAAAGAAAAAGAAAAAAAGC | 26437001 |
| 276 | AAAAAAAAAAAAAAAAAAAAAAAAAGAAAAGAAAAGAAAAAGAAAAGAAAAT | 26888931 |
| 277 | AAAAAAAAAAAAAAAAAAAAAAAAAAGAAGAAGAAGAAGAAGAAAAAGAAAT | 27095228 |
| 278 | AAAAAAAGAAAGAAAGAAAGAAGGAAGGAAGGGAGGGAGGGAGGGAGGAAGGAAGGAAAGAAAGAAAAAGAAAT | 27163637 |
| 279 | GAGAGAGAGAGAGGGAAGGAAGGAAGGAAAGAAAGGAAGGAAGAAAGAGAAAGAGGGAGGGAGGGAGAGAGAGAGAGAAAGGAAAGAAAGGAAGGAAGAGGGGGAGGAGAGGGAAGT | 27297399 |
| 280 | AAAAAAAAAAAAAAAAAAAAAGGAAAGGAAGGAAGGAAGGGGAGAGAAGAGGAGAGGGGAAGT | 27376749 |
| 281 | AAAAAAAAAAAAGAAAAAAAAAGAAAAGAAAGGGAGGGAGGGAAGGAAAGAAAGAAAGAGC | 27739756 |
| 282 | AAAAAAAAAAAAAAAAAAAGAAAAAAGAAAAAGAAAAAGAAAAAGGAAAGGGGAAAAT | 28012320 |
| 283 | AAAAAAAAAAAAAAAAAAAAAAAAAAAAGAAAGAAAGAAAGAAAAGAAAAGAAAAAC | 28102238 |
| 284 | AAAAAAAAAAGAAAGAGAAAGGAAGGAAAGAGAGAGGAGAAAGAAGGAAGGGAGGGAGGGAAAAAAGGAAGGAAGGGGAGGGAGGAAGGAAAGGAAAGGGAAGGGAGAGAGAGAGAAGGAAGGAAAGAGAGGGAGGGAGGAAGGAAGGAAGGAAAGAAGGAAGGAAAGAAAAGGAAGAAAAAAGAAAGGGAGGGAGAGAAGGAGGGAGGGAGGGAGAAAGAC | 28298162 |
| 285 | AGAGAGGGAGGGAGAGAGGAAGGGAGGGAGGGAGAGAGGGAGAGAGAGAAAGAGAGAAAGAGAGAGAGAT | 28455530 |
| 286 | GGGAGGGAAAGGAGAAAGGGAGGGAAGAGGAGAAGGAAGGAAGGGAAAGAGGGGGT | 28550842 |
| 287 | AAAAAAAAAAAAAAAGAAAAGAAAAAGAAAAAAGAAAGAAAAGAAAAAAAAT | 28599573 |
| 288 | AAAAAAAGAAAGAGAGAGAGAGAAAAGGAAGGAAGGGAGGGAGGGAGGGAAAT | 28663580 |
| 289 | AAAAGAAGAAAAGGAAAAGAGGGGAAAAAAAGAAAGAAAGAAAGAAAGAAAGAAAT | 28728350 |
| 290 | AAAAGAAGGAAGGAGAAGGAAGGAAGGAAGGAAGAAGGAGGAGGAGGAGGAAGAAAGGAAGGAAGGAAGGAAC | 28749928 |
| 291 | AGAGAGAGAAAGGAAGGAAAGGAAAGGAAGAAAGAAAAAAAGAAAAAAAAGAAAAGAAAT | 28875578 |
| 292 | GGAGGGAAGGGAAGGGAAGGGGAGGGGAGGGGAGAGGAGGGGAGAGGAGGGGAGAGGAGGGGAGAGGAGGGGAGGGGAC | 29515305 |
| 293 | AAAAAAAAAAAAAAAAAAAGAAAAGAAAAAGAAAAGAAAGAAAAGAAAAAAAAAAGT | 29750817 |
| 294 | AAAGAAAGAAAGGAGGGAGGGAGGAAGGGAAGGAAGAAAGGAAGGAGAAAGAAAGAAGAAAAGAAAGAAAAGGAAGGAAGGAAGAAAGAAAGAGAAAGGC | 29750874 |
| 295 | AAAAAAAAAAAAAGAAAAAGAAAAAAAAAAGAAAGAGAAAGGAGAGGAGAAGGAAAGAAGAAAGGAAGGAAGGAAGGAAGGAAAAAAGGAAGGAGAAAGAAAAAGGGAAAGAAAGAAGAAAGAGAAGAGGAGGAAGAAGGGAGGGAGGGAGGGAGGGAAAC | 29751251 |
| 296 | AAAAGAAAAAAAGGAAAGGAAAGGAAAAAGGAAAGAAAAGAAAAGAAGAAAAGAAAAAGT | 29755887 |
| 297 | AAGGGAAGGAGAGGAGAGGAGAGGAGGAGAGGGGAGGGGAGGGGAGGAGAGGGGAGGGGAAGAGAGGGGAGGGGAGGAGAGGGGAGGGGAGGGGAAGAGAGGGGAGGGGAGGGGAAGAGAGGGGAGGGGAGGGGAGGGGAGGGGAGGGGAAGAGAGGGGAGGGGAGGGGAAGAGAGGGGAGGGGAGGAGAGGGGAGGGGAGGGGAGGGGAGGGGAGGC | 30319893 |
| 298 | AGGAGAGGAGAGGGAGGGGAGGGGAAGAGAGGGGAGGGGAGGAGGGAGGGGGGAC | 30320111 |
| 299 | GAAGGAAGGAAGGAAGGAAGGAAGGAAGGAAGGAAGGAAGGAAGGAAGGAAGGAAGGAAGGAAAGAGC | 30871409 |
| 300 | AGGGGAGGGAGGGGAGGGGAGGGGAGGGGAGGGGAGGGAAGGGAAGGGAAGGGAGAC | 30913912 |
| 301 | GGAGGGAAGGGAAGAGGGGAGGGGAGGGGAGGGGAGGGGAGGGGAGGGGAGGGGAGGGAAGGGAAGGGAAGGGAAGGGAAGGGAAGGGAAGGGAAGGGAAGGGAAGGGAAGGGAAGGGAC | 30978123 |
| 302 | AAAAAAAAAAAAAAAAAAAAAAAGAAAGAAGAAAGAAAGAAAAAAAAAAGAAAAGAAAGAAGAT | 31077512 |
| 303 | AGAGAGAGAGAGAGAAAGAGAGAGAGAGAAAAGAGAGAGAGAGAAAGAGGAT | 31104682 |
| 304 | GAGAAGGGGAGGGGAGGGGAGGGGAGGGGAGGGGAGGGGAGGGGAGGGGAGGGGAGGGGAGGGGAGGGAAGGGAAGGGGAGGGGAGGGAAGGGAAGGGAAGGGAAGGGAAGGGAGGAAGGAGGGAGGGAAGGAAGGAAAAGAAGGAAGGAAGGAAGGAAGGAGGAAAGAAAGAAAGAGAGAAAAAGAAAGAAAGAGAAAGAAAGAAAGAAAGAGAGAGAGAGAGAGAAAGAAAGAGAAAGAAAGAAAGAAAAAGAGAAGAAGGAGAAGGT | 31137047 |
| 305 | AAGAAAGAGGGGGAGAGAGAGGAAGAGAAGGAAGGGAAGGAGGAAGAGGAAGAGGAGGAAAGGGAGGAGGAGGAGGAAGAGAAAC | 31137922 |
| 306 | AAGAAGAAAGGAAGAGAAGGGAAGGAAGAAAAGAAAGAAGGAAGGAAGAAAAGAAGGAAGC | 31206690 |
| 307 | AGAAAGAAGGAAGGAAGAAAAGGAAGGAAAGGAAAGAAGGGAGGGAGGGAGGGAGGGAGGGAGGAAGGAAGGAAGGAGGC | 31206761 |
| 308 | AAGGAAGGAAGGGAAGGAGGGAGGGAGAGAAGGAAGGAAGGAAGGGAAGGGAGGGAGGGAAGGGAGGAAAAGGC | 31206841 |
| 309 | GGAAGGGAGAGGAGGAAGAGGAAGAAGAGAAGAAGAGAGGGAGGAGGAGGGAGAGGAGGAAGAGGAAGC | 31305234 |
| 310 | AAGGAGGAAGAGGGGAGGAGGAAGAAGAGAGGAGGAAGAAAAAGAGGAAGAGAAGAGAGGAAGGAGAAAGAGAAAGAAGAGGAGGAGAAAAGAAGGAGGAAGAAGAGAAGAGT | 31305303 |
| 311 | AAGGAAAGGAAAGGAGAGGAGAGGAGAGAAGAGGAGAGGAAAGGAGAGGAGAGGAGAGGC | 31386354 |
| 312 | AAAGAAAGGAAAGAGGGGAAAGGAAAGGAAAGGAAAGGAAAGGAAAGGAAAGGAAAGGAAAGGAAAGGAAAGGAAAGGAAAGGAAAGGAAAGGAAAGGAAAGGAAAGGAAAGGAAAGGAAAGGAAAGC | 31388733 |
| 313 | GGAAAGGAAAGGAAAGGGGAAAGGAAAGGAAAGGGGAAAGGAAAGGAAAGGAAAGGAAAGGAAAGGAAAGGAAAGGAAAGGAAAAGGGAAAGGAAAGGGGAAAGGAAAGGGGAAAGAAT | 31388861 |
| 314 | AGAAAGAAAGGGAGGGAAGGAGGAAAAGAGGGAAGGAAGGAGGAAGAGAAAGAGAGGGAGGGGAGGAGGAAGAGAGGGAGAGGAGAAGGAAAAGAAGGAGAGAAGGAGGAAGAGAGGGAGGGAAGGAGGAAGAGAGAGAGGGAT | 31403813 |
| 315 | AAGGAGGAAGAGAAAGAGGGGAAGAGGAAAAGAAAGAGGAGAGGAGGAAGAGAAAGAGGGGAGGGGGAAC | 31404166 |
| 316 | AGAGGGAGGGGAGAAGGAAGAGAGGGAAGAAGGAAGGAGGGAGGGAAGAAGGAAGGGAGGGAAAAAGGAAGAGAGGGAGGGGAGGAGGAAGAGAGGGAAGGAAGGAGGAAGGGAGAAAGGAGAAAGAAAGAAT | 31404236 |
| 317 | AAAAAAGAAAAAGGGAGGAAGAAGGAAGGAAGGAAGGGAGGGAGGGAAGGAAGGAGGGAAGAAAGAAAGAGAGAGGGAGGGAGGGAAAGAC | 31412296 |
| 318 | GAAAAAAAGAAAGAAAGGAAAGAAAGAAAGAGAGAAAGGAAAGGAAAGGAAAGAAAGAAAGAAAGAAAGAAAGAAAGAAAGAAAGAAAGAAAGAAAGAAAGAAAAGAAAT | 31412387 |
| 319 | GAAAAGAAAAGAGAGAGAAAAAGAAAGAAGGAGGGAGAGGGAGAGAGGGAGGGAAAGAAAGAGAAAAAAAGAGAGAGAAAGAAAGAAAGAAAGAAAGAAAGAAAAGAAAT | 31412497 |
| 320 | GAAAAGAAAAGAGAAAGAGAGAAAGAAAGGGAGGGAGAGGGAGAGAGGGAGGGAAAGAAAGAGAAAGAAAGAAAGAAAT | 31412607 |
| 321 | AAAAGGGAAAGAGGGAAGGAGGAAGGAAGAAAGAAAGAAAGAGAAGGAAGGAAGGC | 31412686 |
| 322 | AGAAAGAAAAGAAAAGAAGGGAAGGGAAGGGAAGGGGAGGGGAGGGGAGGGGAGGGGAGGGGAGGGGAGGGGAGGGGAGGGGAGGGGAGGGGAGGGGAGGGGAGGGGAGGGGAGGGGAGGGGAGGGGAGGGGAGGGGAGGGGAGGGGAGGGGAGGGGAGGGGAGGGGAGGGGAGGGGAGGGGAGGGGAGGGGAGGGAAGAGAAGAGAAGAGAAGAGAAGAGAAAT | 31584786 |
| 323 | AAAAAAAAAAAAGAGAGAGAGAGAAAGAAAGAGAAAAAAAAAAAGAGGGGAAAAAAC | 31591638 |
| 324 | GGAGGAAGAGGAAGAGGAGGAGAAGAGGAAGAGGAAGAAGGAGAAGAAGAAGAAGAGAAGGAGGAGGAAGAGGAGAAGAAGAAGAAGAAGAAAGT | 32250824 |
| 325 | AAAAAAAGAAAGAAAGAAAGAAAGAAAGAAAGAAAGAAAGAAAGAAAGAAAGAAAGAAAGAAAGAAAGAAAGAAAGAAAGAAAGAAAGGAAAGAAAGAAAGAAAGAAAGAAAGAAAGAAAGAAAGAAGGAAGGAAGGAAGGAAGGAAGGAAGGAAGGAAGGAAGGAAGGAAGGAAGGAAGGGAAAGAAAGAAAGAAAGAAAGAAAGAAAGAAAGAAAGAAAGAAAGAAAGAAAGAAAGAAAGAAAT | 32357362 |
| 326 | AAAAGAAAGAAAGAGAGAGAGAGAGAGAGAGAAAGAAAGAGAGAGAAAGAAGAAAT | 32361605 |
| 327 | GGGGAGGGGAAGGAAGGGGAAGGGGAAGGGGAAGGGGAAGGGGAAGGGGAAGGGGGC | 32426295 |
| 328 | AGAAAAAGAGAGAGAGAGAGAGAAAGAAAGAAAGGAAAGAAAGAAAGAAAGAGAAAAAGAAC | 32512065 |
| 329 | AAAAAAAAAAAGAAGAAAGAAGAAAGAAGAAGAAGAAGAAGAAAAAGAAGAAGAGC | 32540217 |
| 330 | AAAGGAAAGGAAAGGAGAGGAGAAGAAAAGAAAGGAGAAGAAAGGAAAGGAAAGGGAAAGGAAAGGAAAGGAGAAGAAGGAGGAGGAGGAGGGAAAGAAAGAAGAAAGAAAGGT | 32565895 |
| 331 | AAAAGGGGAGGGGAGGGGAGGGGAGGGAGGGGGAGGGAAGAGAAGGGGAGGGAAGGGAC | 32569538 |
| 332 | AAAAAAGAGAAAAGAAAGAAGAAAGGAAGGAAGGAGGGAGGGAGGGAAGGAAGGAAGGAAGGAAAGAAAGAAGGAAGGAAGGAAGGAAGGAAAGGAAGAAAGAT | 33004537 |
| 333 | AAGAGAGAGAGAGAAAGAGAGAGAGAGAGAGAAAGAGAGAGAGAGAGAGAAAGAGAGAGAGAGAGAGAGAGAGAGAGC | 33034722 |
| 334 | AAAGAAGGAAGGAAGAAAGGAAGGAAAGAAGAGAGAAAAGAAAAAAAAAGAAAGAGAGAC | 33073062 |
| 335 | GAAAGGGAAGGGAAGGAGAAAAGAAGGAAGGAAGGAAAGAAGGAAGAAAGAAAGGAAGGAAGGAGAAAGGAAAGGAAAGGAAGGAAGGGAAGGAAGGGAAGAAAAAAAGAAAT | 33484628 |
| 336 | AAAAAAAAAAAAAAAAAAAAAAAAAGGGAGAGAGAAAGGGAAGGAAGGAAGGAAAT | 33552663 |
| 337 | GGAAAGGGGAGAGGGGGAGAGGGGGAGAGGGGGAGAGGGGGAGAGGGGGAGAGGGAGAGAGGGAGAGAGGGAGAGAGGGAGAGC | 33567366 |
| 338 | AGAGAAAGAGGGAGGGAGGGAGAGAGAGGAGAGAGAGAGAAAGAGAGAGAGC | 33687942 |
| 339 | AAGAAGAAGAAGAGGAGGAAGAAGAGAAAGAAGAAGAAGAGGAAGAAGAGGAAGAGGAGGAAGAAT | 33832854 |
| 340 | AAGAGGAAGAAGAAGAGGAAGAAGGAAGAAAAGAAAGAAGAGGAAGAAGAAAGAAGAAAGAAGGAGGAGGAAGAT | 33832947 |
| 341 | GAAGAAGGAAGAGGAAGAGGAAGAAGGAAGAGGAAGAGGAAGAAGAGGAAGAAAAGAAGAAGC | 33833022 |
| 342 | GAAGAAAAAAAAAGAAGAAAGAAGAAGGAAGAAGAAAGAAGGAGAAGGAGGAGGAGAAGGAGAAGAAGAAGAAGAGAAGAAGAAAAGAAGAAGAAAGAAGAAAGAAGGAAGAAGAAGAAGAAGAAGAAGAAGAAGAAGAAGAAGAAGAAGGAGAAGAAGAAGGT | 34012895 |
| 343 | GGGGAGAGGGAGAGGGAGAGAGAGAGGGAGAGGGAGAGGGAGAGGGAGAGAGAGAGGGAGAGGGAGAGGGAGAGGGAGAGGGAGAGC | 34272332 |
| 344 | GGAGGGAGAGGAGAAAAAGAGAGAGAGGGAGGGAAGGAGAGGAAGAGAGGGAGT | 34451160 |
| 345 | GGGAAGGGAAAGAAGGGGAAGGGAGGAGAGGAGGGAGGGGAAGGGAAGAGAGGGGAAGGGAAGGGAAAGGAGGGGAAT | 34627055 |
| 346 | AAAAGAAAAAGGAAAGGAAAGGGAAAGGGAGAAGGGAGGGAGGGAAGGAGGGAAGGAGGAAGGAAGGAAGGAAGGAAGGAAGGC | 34746930 |
| 347 | AGAAAGAGGAGGAGGAAGGAGGGGAGAAGGAGAAAGGGGAAGGAGGAAGAAGGGGAGGAGGAAAAAGAGGAGGGAGAGGAGGAGGAGGAAGAGGAGGGAGAGAAAAGAGGAGGAAAAGGAGAAGGAGGAAT | 34763111 |
| 348 | GGAAAGAAAGAAAGAAAGAAAGAAAGAAAGAAAGAAAGAAAGAAAGAAAGAAAGAAAT | 34843070 |
| 349 | AAAAGAAAGAAAGAAGGAAAGAAAGAAAGAAAGAGAAAGAAAAAGAAAAGAAAAGGAGGGAGGGAGGAAGGAAGGAGGGAAGGAAGGAAAGAAGGGAGGGAGGGAAAGAAAGAGAAAGAGAAGAAAGAAAGAGAGAAAGAGAAAGAGGGAGGGAAAGAAAGAAAGAGAAAGAGAGAAAAAGAAGGAGGGAGGGAGGAAGGAAGGAAGGAAGGAAGAAAGGAAGGGAGGGAGGGAGGGAAAAAGAAGAGGT | 35412323 |
| 350 | GAAAGAAAGAAAGAAAAGAAAGGAGAGAGAGAGAGAGAGGGAGAGAGAGAGAGAGAGAGAGGGAGAGAGAGAGAC | 35601249 |
| 351 | AGAGAGAGAGAGGGAGGGAGGGAGGAAGGGAGGGAGAGAGAGAGAGAGAGAGAGAAAGAAAGGAGGGAGGGAGGGAGGGAGGAAGAAAGAAAAGAGAAGAGGAGAGAAGAT | 35601324 |
| 352 | GAAAAGAAGAAAAGAAAAGAGAAGAGAAGAGAGGAAAAGAAAAGAAAAGAAAT | 35601474 |
| 353 | AAAAAAAAAAAAAAAAAGGAAAGAAAGAAGGAAGGAAAGGAAAGGAAGAAGGAAGGAAGGAAGAAAAAAAGAAAGGGAGAAAGAAAGAAAGGAAGGAAGGAAGGAAGGAAGGAAGGAAGGAAGGAAAGAAAGGAAAGAAAGGAAGAGAAAT | 35835400 |
| 354 | AAGAAAGAAAGGAAAGAAAAAAAGAAAAGAAGAAAAGAAAAGAAGGAAAGGAAAGGAAAC | 35835704 |
| 355 | AAAAAAAGAAAAGAAAAGAAAAAAAGGAAAGGAAAGGAGGAAGGAAGAAAGGAAGGAAGGAAAGGAGGGAGGGAGGAAGGAAGGAAGGAAAAT | 35835780 |
| 356 | AAAAAGAAAGAAAGAAAAAGGAAGGAAGGAAGGAAGGAAGGAAAAAGAAGAAAAAAGGAAAAGAAAGAAAGAGAAGAAAGAGAAAGAAAGAAAAGC | 36013005 |
| 357 | GGAGGGAAGGAGGAAGGGAGGGAAGGAAGGAGGGAGGGAGGGAGGGAGGGAAAGAAGGAAGGAAT | 36219000 |
| 358 | AAAAAAAAAAAAAAAAAAAAAAAAAAAAAAAAAAAAAAAAAAAAGAGAGGGAGAGAGAT | 36404364 |
| 359 | AAAAAAAGAAAGAAAAGAAAGAAAGAAAGAGAGAGAGAGAGAGAGAGAGAGAGAGAGAGAGAGAGAGAGAGAGAGAGAAAGAAAGAAAGAAAGAAAGAAAGAAAGAAAGAAAGAAAGAAAGAAAGAAAGAAAGAAAGAAAAT | 36467145 |
| 360 | AAAAAAAAAAAAAGAAAAGAAAAGAAAAGAAAAAGAAAAAGAAAAGAAAAGAAAAAC | 36496827 |
| 361 | AAAAAAAGAAAAAAAGAAGAAGAAGAAGAAGAGGAAGAGGAAGAAGGAAGAAGGAAGGAAGAAGGAAGAAGAAGAGAGAC | 36575898 |
| 362 | AAAAAAAAAAAAGAGAGAGAAAGAGAAAGAAGAGAGAGAGAAAAAAGGAAGGAAGGAAGGAAAGAAGGAAGGAC | 36576224 |
| 363 | AAAAAAAAAAAAAAAAAAAAAAAGAAAGAAAGAAAGAAAAAGAAAAAAAAAAT | 36578948 |
| 364 | AAAAAGAAAGAAAGAAAGAAAGAAAGAAAGAAAGAAAGGAAGAAAGGAAGGAAGGAAGGAAGGAAGGAAGGAAGGAAGGAAAGAAAC | 36580773 |
| 365 | AAAAAAAAAAAAAAAAAAAAAAAAAAAGAAAGAAAGAAAAGAAAGAAAAAAGAAAAT | 36628733 |
| 366 | AAAAAAAAAAAAAAGAGAGAGAGAGAGGGAGGGAGGGAGGGAAAGAAAGAGAAAAAGAGAGAGAAAGAAAAGAAAGAAAGGAAAT | 36773054 |
| 367 | GAAAGAAAAGAAAGAAAGAAAAAGAAAGAAGGAAAGGAAAGAAGGAAAGAAAGAAAGAAGGAAAGAGAAAGAAAGAAAGAAAGAAAGAAAGAAAGAAAGAAAGAAAGAAAGAAAGAAGGAAAGAGAAAGAAAGAAAGAAAGAAAGAAAGAAAGGAAAGAAAGAAAGAAAGAAAGAAAGAAAGAAAGAAAGAAAGAAAGAAAGAAAGAAAGAAAGAAAGAAAGAC | 36975994 |
| 368 | AAGGGAAGGAAGGAGGGAAGGAAGGAAGGGAAAGGAAGGAAGGGAGGAAGGAAGGGGGGAAGGAAGGGAGGGAGGAAGGAAGGGAGGGAGGAAGAAAGGAC | 37228030 |
| 369 | GGAGAAGAAGAAAAAGAAGAGGAGGAAGAAGAAGAAGAAGAAGAAGAAGAGT | 37308898 |
| 370 | AAGAAGAAGAGGAAGAAGAAGAGGAAGAAGAAGAAGAAGAAGAAGAAGAAGAAGAAGAAGAAGAAGAAGAAGAAGAAGAAGAAGAAGAAGAAGAAGGAGAAGAAGAAAAAGAAAAGAAGAAGAAAGC | 37308950 |
| 371 | AGAAGGGAAGGGGAGGGAAAGGAAAGGAAGAGAAAAAGGGGAAGAGAGGGGAAGGGAGGGAAGAGAAAGGGAAAAGGAAGGAGAAAAAGGGGAGGGGAAGGGAGGGGAGGGGAGGAGAGGGAGGAGGGGAGGGGAC | 37450434 |
| 372 | AGAGGGAGAGAAAGGGAAGAGAGAGAGGGAAGAGGAGAGAGAGAAAGGGAGAGAGGGAGAGGGAGAGGGAGAGGGAGGGGGAAGAGAGAGAGAGAGAGAGAAAAT | 37522825 |
| 373 | GGAGAAGGGAAGGGGAGGGAAAGAGAAGGGGAGGGGAGGGAAGGGGGAAGGGAAGAAT | 37541114 |
| 374 | AGAGAAGAGGGGAGGGAAGAAGAAAAGAGGAGGGAGGAAGAGAGGGAAGAGAGT | 37706619 |
| 375 | AGGAGGAGGAGGAAGAGAAGAAAAGAAGAGGAAGAAAAGAAGGAGGAGGAAGAGGAGAAGAAGAAGAAGAAGGAGGAGGAGAAGAAGAAGAAGAAGAAGGAGGAGGAGGAGGAGGAAGAGGAGGAGGAAGAGGAAGAGGAGGGAGGAGGAGGAAGAGGAGGAGGAGGAAGAGGAGGAGGT | 37864904 |
| 376 | AGAGGAGGAGGAGGAAGAGGAGGAGGAAGAAGAGGAAGAGGAGGAGGAGGAGAAAAGGAGAAGAGAAAGAAGAAC | 37865084 |
| 377 | AGGAGGAGGAGGAGGAGAAGGAAAGGAAGAAGAAGAAAAGAAGAAGAAGGAGAAGGAGGAGGAGGAC | 37865179 |
| 378 | AAGAAGGAGGAAGAGGGGAGAAGAAGGAGGAGGAGGAGAGAAGGAGAAGGAGGAGGAGGAGAGAAGGAGAAGAAGGAGGAGGAGAGAAAGAGAAGAGAAAGAAGAAGAGAAAGAAAAAGAGAGGAT | 37865281 |
| 379 | AGGAGGAGGAGAAGAAGAAGAAGGAAGAGGAGGAGGAGGAGGAAAGAGAAGAAAAAGAAAGAAAAAC | 37865408 |
| 380 | GGAGGGAGGGAGGGAGAGAAGGAGAAGAGGGAAGGAAGGAAGGGAGGGAGGGAGGC | 38044387 |
| 381 | GGGAGGGAAGAGGAAGGGAGGGGAGGGGGAAAGGAGGGGAGGGGAGGGGAAAGGAGGGGAAGGGAGGGGAGGGGAAGGGAGGGGAT | 38044483 |
| 382 | AAGAGAAGGAGGGAAAGAGAGAGGGAGAGAAAGGAGGAAGGAGGGAGGGAAAAGAGGAAGGAGGGAGGGAAAGAAGGAAGGAGGGAGGGAAAGAAGGAGGGAC | 38117200 |
| 383 | AAGAAAGAAAGAAAGAAAGAAAGAAAGAAAGAAAGAAAGAAAGAAAGAAAGAAAGAAAGAAAGAAAGAAAGAAAGAAAGAAAGAAAGAAAGAAGGAAGGAAGGAAGGAAGGAAGGAAGGAAGGAAGGAAGGAAGGAAGGAAGGAAGGAAGGAAAGAAAT | 38146618 |
| 384 | AAAAAAAAAAAAGAAAGAGGAGGGAAGGAAAGAAAGAAAGAAAGAGAGAGAGGGAGGGAGGGAAGGAAGGAC | 38189478 |
| 385 | AGAGAGAGAAAGAGAGAGAGAGAGAGAGAGAAAGAAAGAAAGAAAGAAAGAAAGAGAGAAAGAAAGAAAGAAAGAAAGAAAGAAAGAAGGAAAGAAGAAAGAAAGAAAGAAAAGAAAGAAAGAAAGAAAGAAAGAAAGAAAGAAAGAAAGAAGGAAGGAAGGAAGGAAGGAAAGAAAGAAAGAGGGAGGGAGGGAAAGAAGGAAGGAAGGGAGAGAGAGAAAGGAAGAAAGAAAGAAAGAAAGAAAGAAAGAAAGAAAGAC | 38189565 |
| 386 | AAAAAAAAAAAAAAAAAAAGGAAAGGAAAAGAAAAAAGAAAAGAAAGAAAAAGC | 38286982 |
| 387 | GAAAAAAAAAAGAAAGAAAGAAAGGAAAGGAAAGGAAAGGAAAGGAAAGGAAAGGAAAAAAAAAGT | 38461787 |
| 388 | AAAAAAAAAAAAAAAAGAAAGAAAGAAAGAAAAAGAAAAAGAAAAAAAAAAAAGAAAT | 38513422 |
| 389 | GAGAGAGAGAAAGAGAAAAAAAAGGGAGAGAGAAAGAGAGAGAGAGAGAGAGAGAGAT | 38612753 |
| 390 | GGGGAGGAGAGGAGAGAGGAGAGGAGAGGAGAGGAGAGGAGAGAGGAGAGGAGAGGAGAGGAGAGAGGAGAGAAGAGAAGAAGAGAAGAGAGGAGAAGAGAGGAGAGGC | 38896385 |
| 391 | AGGAGGAAGGGAAGAAGGAAGGGAGGGAGGGAGGGAGGAAAGAAGGAAGGAAAGAAGGAAGGAAGGAAGGAAGGAAGGGAGGAAGGAGAGGT | 39248879 |
| 392 | AAAAAAAAAAAAAAAAAAGAAAGAAAGAAAGAAAAGAAAAGGAAAAAAAAAGAAAGAGGAC | 39358463 |
| 393 | GGAAAAAAGAGAAAGAGGGAGGAGAGAAGGAGGAAAGAAGGGAAGGAAAGAGGAAGAAAGAGAT | 39388113 |
| 394 | AGAGAGAGAGAGAGAGGGAGGGAGGGAGGGAGGAAGGAAGGAAGAAAGAAAGAAAAAGAAAGAAAGAAAGAAAAAAGAAAGAAAGAAAGAGAAAGAAAGAAAGAAAGAAAGAAAGAAAGAAAGAAAGAAAGAAAGAAAGAAAAAAAGAAAAC | 39491783 |
| 395 | AAAAAAAAAAAAAAGAAAGAAAGAAAGAAAGAAGGAAAGAAAAAAAGAGAAAGAAAAC | 39663682 |
| 396 | GGAAGGGGAGGGGAGGGGAGGGGAGGGGAAGGGAGGGGAGGGGAGGGAAAAAGAAAT | 39710516 |
| 397 | AGGAGGGAGGAAGAAGGAGGAGGAAGAAAAAGAGGAAGAAGAAAGGAGGAAGGAAGAAGAAAGAAGAAT | 39805994 |
| 398 | GAAAAAAAAAAAAGGAAGGAAAGAAGGAGGAGGAGGAGAAGAAAGAAGAAGT | 39806301 |
| 399 | AAAAAAAAAAAAGGAAAGAAGGAGAAGAAAGAAGGAGGAGGAGGAGGAGGAGGAGAGAAGGAGGAGAAAAGT | 39806745 |
| 400 | AAAAAAGAAAGGAAAGAAAGAAAGGGAGGGAGGGAGGGAAAGAAAGAAAAGAGAGAGAGAAAGAAAGAGGC | 39864047 |
| 401 | AAAAAAAAAGAAGAAAAAGAAAGAGAGAGAGAGAGAGAGAAAGAGAGAGAGAAAGAAAAGAAAAGAAAGAC | 39864393 |
| 402 | AAAAAAGAAAGGAAAGAAAGAAAGGGAGGGAGGGAGGGAAAGAAAGAAAAGAGAGAGAGAAAGAAAGAGGC | 40020910 |
| 403 | AAAAAAAAAGAAGAAAAAGAAAGAGAGAGAGAGAGAGAGAAAGAGAGAGAGAAAGAAAAGAAAAGAAAGAC | 40021256 |
| 404 | AAAAAGAAAGAAAGAAAGAAAGAAAGGAAGGAAGGAAGGAAGGAAAAGAAAGAAAGAAAGAAAGGAAGGAAGGAAAAGAAAGAAAGGAAGGAAGGAAGGAAGGAAGGAAGGAAGGAAAAGAAAGAAAGAGAGAAAGAAT | 40215812 |
| 405 | AGAGAGAGAGGGAGGGAGGGAGAGAGAGAGAGAGAGAGAAAGAAAGAGAGAAAGAAAAGAAAGGAAGGAAGGAAAGAAAAGAAAGAAAGAGAAAGAAAGAAC | 40312911 |
| 406 | GGAAGAAGGAGAAGGAAAAGAAGAAGGAGAAGAGAAGAGAAAGAAAAGAAGGAAGAAAGAAGAAAGAAGAAAGAAGAAAGAAGAC | 40614485 |
| 407 | AAAAAAAAAAAAGGAAGGAAGGAAGGGAGGAAGGAAGGAAGGGAGGAGGGGAGGGAGGGAGGGAGGGAC | 40712696 |
| 408 | AAAAAAAGAAAAGGAAAGAAAAAGAAGGGAAGGGGAGGGGAGGGGAGGGGGAAGAAAGAAAAGAGAAGAGAAGAAAGGAAAAGAAAAGAAAAGAAAAGAAAAGAAAAAGT | 40740526 |
| 409 | AAAAAAAGAAAAAGAAAGAAAGAGGGAGAGAGAGAGAAAGAAAGAAAGAAAGAGGGAGAGAGAAAGAAAAAGAAAGAAAAAAAAGAAAGAAAGAAAGAAAAAGAAAAGAAAGAAAGAAAAT | 40742641 |
| 410 | GAAAGGAAAAAGAAAGAAAAGAAGAAAAGGAAAGAAAGAAGGAAAAGGAAAAGAAAGAAAGGGAGGAAAGAAAGGGAAGGAAAAGAAAAGAAAGAAAAGAAAAGAAAAGAAAAAGAAAAGAAAGGGAAGAGGGAGAGAGGGAGGGAGGGAAGGAAAGAAGAAAGGAAGGAAGGAAGGAAGGAAGGAAGGAAGGAGGAAGGT | 40785016 |
| 411 | AGAGAGAGAGAGAAAGAGAGAGAAAGGAAGGAAGGGAGGGAGGGAGGGAGGGAAGAAGGGAAGAAGT | 40791864 |
| 412 | GGGGAGAGGGAGAGGGAGAGGGAGGGGGAGGGGGAGGGAGAGGGAGAGGGAGAGGGAGAC | 40833113 |
| 413 | AAAAAAAAAAAAAAAAAAAGAAAGAAAAGAAAAGAAAGAAAGAAAGAAAAGAAAAT | 41070629 |
| 414 | AAAAAAAAAAAAAAAGAAGAAGAAGAAGAAAGAAGGAAAGAAAGAAAGAAAC | 41075401 |
| 415 | AAAAAAAAAAAGAAAGAAAGAAAAAGAGAGAAGAAAGAGAAAGAAAAAAAGAAAGAAGGAAAGAGAAAAAGAGAAAGAGAGAAAGAAAGGAAAGAAAGAAGAAAAAGAAAC | 41387394 |
| 416 | AGAAAGAAAGAAAGAAAGAGAGAGAGAGAGAGAGAAGGAAGGAAGGAAGAAAGAAAGAAAGAAAGAAAGGAAAGAAGGAAGGAAGGAAGGAAGGAAGAAAGAAAGAAAAAGAAAGAAAGAAAAAAGAAAAGAAGGAGGGAAAGGGAGGGAGGGAAGGAAGGAAGGAAGGAGAAAAAAAT | 41798069 |
| 417 | AAAAAAAAAAAAAAAAAAGAGAAAGAAAAGAAAAAAAAAAAAAGAAAGGAAAAGAAAT | 41801165 |
| 418 | AAAAAAAAAGAAAGAAAGAAGGAAAGAAGGAAGGAAGGAAAGAAGGGAGGGAGGGAAGGAGAAAGGAAGGAAGGAGAAGAGAGAGAAAGAGAGAGAAAGAAAAT | 41827100 |
| 419 | AAAAAAAAGAAAAGAGAGAGAGAAAGAGAGAGAAAGAAAGAGAGAAAGAGAAAGAAAGAAAGAAAGAAAAAAGAAAGAAAGAAAGAAAGAAAGAAAGAAAGAAAGAAAGAAGGAAAGAAAGAGT | 41833513 |
| 420 | AAGAAAGAAAGAAAGAAAGAAAGAAAGAAAGAAAGAAAGAAAGAAAGAAAGAAAGGAAGGAAGGAAGAAAGC | 41863902 |
| 421 | AAAAAAAGAAAAAAAGAAAGAGAAAGAAAGAAAGAAAGAAAGAAAGAAAGAAAGAAAGAAAGAAAGAAAGAAAGAAAGAAAGAAAGAAAAAGT | 42246086 |
| 422 | AAGAAAGAAAGAAAGAAAGAAAGAAAGAAAGAAAGAAAGAAAGAGGAAAAGAAAAGAAAAGAAAGAAT | 42294532 |
| 423 | AGAAAGAGAGAGAGAGAGAGAGGAGAGAGAGAGAGAGAGAGAGAGAGAAGAT | 42313239 |
| 424 | GGAGAGGGGAGGGGAGGGGAGGGGAGGGAAGGGAAGGGAAGGGAGGAAAGGAAGGAAAGGAAGGAAGAAAGAAAAGAAAT | 42524242 |
| 425 | AAAGAAAGAGAGAGAAAGAAAGAAAGAAAGAAAGAAAGAGGAAGGAAGGAAGGAGAGAGAAAGAAAGGAAAAGGAAAGAAAGAAAGAAAGGGAGAGAGAGAGAAAGAAAGAAAAAGGAAAGAAAGAAAGGGAGAGAGAGAGAAAGAAAGAAAAGAAAGAAAGGAAGGAAGT | 42557327 |
| 426 | AGAGAGAGAGAGAGAGAGAGAGAGAGAGAGAGAGAAAGAGAGAGAGAGAGAGAGAGAGT | 42743867 |
| 427 | GAGAGAGAGGGAGAGAGAGAGAGAGAGAGAGAGAGAGAGAGAGAGAGAGAGAGGT | 42817011 |
| 428 | AAAAAAGGAAGAAGGAAGGAGGGAGGGAAGGAAGGAAGGAAGGAAAGGAAGGAAGGGAAGAGAGAGAT | 42841869 |
| 429 | GGGGGAGGGGAGGGGAGGGGAGGGGAGGGGAGGGGAGGGGAGGGGAGGGGAGGGGAGT | 43102391 |
| 430 | AAAAAAAAAAAAAAAAGAAGAAGGGGGAGGGGGAGGGGGAGGGGGAGGGGGAGGAGGAGGAGAAGGAGAAGGGGGAGGGGGAGGAGAAGGAGAAGGAGAAGAAGAAGAAGAAGAAGAAGAAGAAGAAGAAGAAGAAGAAGAAGAAGAAGAAGAAGAAGAAAT | 43141033 |
| 431 | GAGGAAGGGAGGGAGGAAAGAAGGAAGGGAGGGAGGAAAGGAGGAAGGAAGGAAGGAAAGAAGGAAAGAAAT | 44659984 |
| 432 | GAGGAGAGGAGAGGGGAGGGGAGGAGAGGAGAGGGGAGGGGAGGAGAGGAGAGGGGAGGGGAGGAGAGGGGAAT | 44689294 |
| 433 | GAAGGAGGGAAGGAGGGGAGGGAAGGAAGGAGGGGAGGGAGGGAGGGAGGGGAGGGAGGGAAGGAGGGGAGGGAGGGAGGGGGAGGGAGGGAGAGAGGGAAGGAAGGAAGGGGAAAGAGAGAGAGAAAGC | 44858326 |
| 434 | AAAAGAAAAAGAAAAAGAAAAAAGAAAGAGAGAGAGAGAAAGAAAGAAAGGAAGGAAGGAAAGAAGGAAGGAAGGAAGGAAGGAGC | 44947837 |
| 435 | AGAAAGAAAAAGAAGAAAGAAAGAAAGAAAGAAAGAAAGAAAGAAAGAAAGAAAGAAAGAAAGAAAGAAAGAAAGAAAGAAAGAAAGAAAGAAAGAAAGAAAGAAAAGAAAGGGAGGGAGGGAGGGAGAC | 45018898 |
| 436 | AGGAAGGAAGGAAGGAAGGGAGGGAGGAAGAGAGAGAGGGAAAGAGGGAAAAGC | 45025081 |
| 437 | AGAAAAAAAAAAAAAGAAAGAAGGAAGGAAGGAAGAAAGGGAGGGAGGGAGGGAAGGAAGGGAGGAAAGGAGAGAC | 45126284 |
| 438 | AAAAAAAAAAAAAAAAAAAAAGGAAGGAAGGAAAGAAAGAAAAGGAAAAGAAAGAAAAGAAAAT | 45428969 |
| 439 | GAAAAAAAAAAAAGGAAAGAGAGAGAGAGAGAGGAGGGAGGGAGGGAGAAAAGAAGGAAGGAAGGGAGGGAGAGAGGGAGGGGAAGGAAGGAAGGAT | 45429317 |
| 440 | AAAAAGGAAAAAAGGAAGAAAAAGAAAGAGAAGGAAAGAAAGAGAGAGAGGAAGGAAGGAAGGAGGGAGGGAGGGAGAGAGGGAGGGAAAGAAGGAAGGAAGGAAGGAGAGC | 45476441 |
| 441 | AAAAAAAAAAAAAAAAAAAAAAAAAAAGAAAGAAAAGAAAGAAAGAAAGAAAAGAAAAGAAAAAGAAAGAAAGAAAGAAGAAAT | 45499114 |
| 442 | AAAAAAAAAAAAAAAAGAAAAGAAAGAAAGAAAAAGAAAAAGAAAAAGAAAT | 45708326 |
| 443 | AAGGAAAGAAGGAAGGAAGAAAAGAAAGAAAGAAAGAAAGAGAGAGAGAGAGAAAGAAAGAAGGAAGGAAGGAAGGAAC | 46144778 |
| 444 | GAGGGAGGGAGGGGGAAGGAAAAGGGAAGGAAAAGAGAAGGAAAAGGGAAGGAAAAGGGAAGGGAAGGGAAGGGAAAAGAAAGT | 46144879 |
| 445 | AAAGGAGGGAGGGAGGGAGGGAGGAAGGAAGAAAGAAGGAGGGAGGGAGGAAGGGAAGGAGAGAGAGAGAAAGGAAAGGAAGAAAAAGAAAGAGAGAAGGAAGGAAGGAAAGAAAGAAGAC | 46169707 |
| 446 | AAAAAGAAAGAAAGAAGAAAAAGAAAGAAAGAAAGAAAGAAAGAAAGAAAGAAAGAAAGAAAGAAAGAAGGAAAGAAAGAAAAT | 46198100 |
| 447 | GGAAGGAAGGAAGGAAGGAAGGAAGGAAAAAAGGAAAGGAAGAAGAAAGAGAGAGAC | 46384535 |
| 448 | AGAAGGGGAAAAGAAAAGAGGAGGAAAAAAAAGAAAGAAAGAAAGGAAGAAAAC | 47095200 |
| 449 | AAAAAAAGAAGGGGAAGAAGGGGAGGAAGGGAGGAAGGGAGGGAGGGAGGAGAAAAGAAAAGAAAAC | 47167275 |
| 450 | AAAAGGAAAGAGAGGGAGAGAAAGAAAAGGAGAGAAAGGAAAGGGAGGAAAGGGGAGAGGGAAAGGAGAGAAGAAAGGC | 47314473 |
| 451 | GGGGAAAGGAAAAGGGGAAAGGGGAAAGAAAGGGGAGAAAGGGGAGAGAGGAC | 47314561 |
| 452 | GAGGAAGGGAAGGGGAGAAGGGAAAGGAAGGGGAGGGGAGGAAGGGAAGGGAAGGGT | 47362893 |
| 453 | AAAAAAAAAAAAGAAAAAGAAAGAAAGAAAAGAAAAAGAAAAAAAGAAAGAGGGC | 47438532 |
| 454 | AAAAAAAAAAAAAAAAAAAAAAAAAAAAAAAAAAAAAAAAAAAAAAAAAAAAAAT | 47547260 |
| 455 | AGAAAAGAAAAAAAAAAAAAAAGAAAGAAAGAAAGAAAAAGAAAAGAAAGAAAAGC | 47574662 |
| 456 | AAAAAAAAAAAAAAAAAAAAAAGAAGAAGAAGGAGAGAGAGAGAGAGAAAGAGAGAGAGAGAGAGAAAAAAAAAGAAAGAGAGAGAC | 48282688 |
| 457 | AAAAAAAAAAAAAAAAAAAAAAAAAAGAAGAAGGAAAGGAAGAAAGAAAGAAAGAGC | 48994548 |
| 458 | AGAGAGAAGGGGAGAGAGAGAGAGAGAAGAAGAAGAAGAAGGAGAAGGAGAAGGGGAAGGAGAAGGAGAAGGAGAAGGAGGAGAAGGAGGAGAAGGAGAAGGGGGAGGAGAAGGAGAAGGAGGAGAAGGAGGAGAAGAAGGAAGAAGGAGGAGGAGGAGGAGGAGGAGGAGGAGGAGGAGGAGGAGGAGC | 49028288 |
| 459 | AAAGAAAAGAGAAGAGAAGAGAAAAGAGGGAGGGAGGAAGGAAGGAAGGGGAAAGGAAGAAAAGAAGAAGAAAGAAAAAGAAAAAGAAAGGAAGGAAGGAAGGAGAAGAAAGGAAGAAAAAGAAAGAAAGAAAAGAAGAAGAAGAAGAAAAAAAGGAGGAGGAGGAGGAAAGAAAAGGAAGGAAGGAAGGGGAAGGAAGGAAGGAAC | 49351677 |
| 460 | GAAGAGAGGGAGGGAGGGAGGGAGGGAAAGGAAAGAAAAAGAAAAGAAAAGAAAAAAGAAAAGAAAAGAAGGC | 49351884 |
| 461 | AGGGAGAGGGAGAGGGAGAGGGAGAGGGAGAGGGAGAGGGAGAGGGAGAGGGAGAGGGAGAGGGAGAGGGAGAGGGGT | 49503224 |
| 462 | AAGAGAGGAAGGAAGGAAGGAGGGAAGGAAGGAAGAAGGGAAGGAAGGAAGAAGGGAAGGAAGGAAGGAGGGAAGGAGGGAAGGAGGGAGGGAGGGAAAGAGAGAGAGAGGAGAGGAGAGGGGAGGGGAAGGAGAGGGAGGAAGGAAAAGGAGAGAGAAGAAAGGGAAGGAGAAAGAGGAAAGGGAAAGGGAAGGGAAGAAGAAAT | 49537607 |
| 463 | GAAAGAGAGGAGAGGGAGAAGGAGAGGGAGAGGGAGAGGGAGAAAGAGAGAAAGAGAT | 49668320 |
| 464 | AGAGAAGAGAAGAGAAGAGAAGAGAAGAGAAGAGAAGAGAAGAGAAGAGAAGAGAAGAGAAGGGAAGAGAAGAGAC | 49707672 |
| 465 | AGGGAGAAGGAGAGGGAAAGGAAAGGGGAGAGGAGAGGAGGGGAGGGGAGGAGAGAGGAGGGAAGGGGAGGGGAGGGGAGGGGAGAC | 49716534 |
| 466 | GGAAGGAAGGAAGGGAAGAAAGAGAAAGAAAGAGAGAGAAAGAAAGAGAAAGAGAGAAAAAGAAAGGAAGGAAGGAAAGAAAAGAAAGGAGAAAGAAAGAAAAAAAGAAAGAGGGAGGGAGGGAAGGAGGAAGGAAGGAAGGAAGAAAAAGAAAAAGAAAGAAAGAGAGAAAGAAAGGGAGAAAAAC | 49716653 |
| 467 | GGGGAGAGAGAAGAAAGAAAGGAAGAAAGGAGGGAGAAAAGGAGGGAGGGGGGAGC | 49735913 |
| 468 | AGAGGAAGGAGGAAAGGAAGGAAGGAAAAAGGAGGAAGGAGGAAGGAAGGAGGGAAGAAAGGAAGGAAGT | 49854331 |
| 469 | AAGAAAAAGAAAAGAAAGGAGAAAGAAAGAAGAAAGAAAGAAAGAAAGAAAGAAAGAAAGAAAGAAAGAAAGAAAGAAAGAAAGAAAGAAAT | 49965207 |
| 470 | GGAGAGGAGAGGAGAGGAGAGGAGAGGAGAGGAGAGGAGAGGAGAGGAGAGGAGAGGAGAGGAGGT | 50325733 |
| 471 | AGAAGAAAAGAAAGAAAAGAAAAAAGAAAAGAAAAGAAAAGAAAAAGAAAAGAGAAGAGAAGAGAAGAGAAAAGAAAAAAGAAAAGAAAAGAAAT | 50706125 |
| 472 | AAAAAAAGAAAGAGAGAGAGAGAGAGAGAGGGAGGGAGGGAGGGAGGGAGAGAGAGAGAGAGAC | 51237808 |
| 473 | AGAGAGAAAGAAAGAAAAGAAAGGAAAGAAAGAAAGAAAAAGAAAGAAAGAAAGAAAGAAAGAAAGAAAGAAAGAAAGAAAGAAAGAAAGAAAGAAAAAGAAAAAGAT | 51237872 |
| 474 | AAAAAGAAAGAAAGAGAGAGAGAAAGAAAGAAAGAAAGAAAAGAAAGAAAGAAAGAAAGAAAGAAAGAAAGAAAGAAAGAAAGAAAGAAAGAGAAAGAAAGAAAGAAAGAAAGAAGC | 51252434 |
| 475 | GGAAGGAAAGAAGGAAGGAAGGAAGGAAGGAAGGAAGGAAGGAAGGAAGGAAGGAAGGAAGGAAGGAAGGAAGGAAGGAAGAAAAT | 51412109 |
| 476 | GGAGGGGAAGGGAAGGGAGGAGGGAGGGAGGGAGGGAGGGAGGGAGGAAGGAAGGAAGGAAGGAAGGAAGGAAGGAAGGAAGGAAGGAAGGAAGGAAGGAAAGAAAAT | 51499715 |
| 477 | AAAAAGGAAGGAAGGAAGGAAGGAAGGAAGGAAGGAAGGAAGGAAGGAAGGAAGGAAGGAAGGAAGGAAGGAAGGAAGGAAAAAGAAAGAAGGAAGGAAGGAAGGAAGGAAAGGAAGGAAGGAAGGAAGGAAGGAAGGT | 51609256 |
| 478 | GAAGGAAGGAAGGAAGGAAGGAAGGAGGGAGGGAGGGAGGGAGGGAGGGAAGGAGGGAGGGAGGGAGAGAAGGAAGGAAGGAAGGAAGGAAGGAGGAGGGAAGGAGGGAGGGAAGGAAGGAAGGAAGGAAGGAAAGAAAGAAAAT | 51638222 |
| 479 | AAAGAAAGAAGAAGAAGAAGAAAGAAGAAGAGGAAGAAGAGGAAGAAGAAGGAAGAAGAAGAAAGAAGAAGAAGAAAGAAGAAAGAAGAAGAAGAAC | 51742822 |
| 480 | GAGGAAGAAAGAGGGAGGGGGAAGGGAAAGGGAGAAGAAGGGGAAAAGGGAAAAGGAGGAAAGAGAAGAGAGGAAGAT | 51778107 |
| 481 | AAAGAAGAGGAGGGGAGGGGAGGGAAGAGGAGGGAAGGGGAGAGGAGGGAAAGGGAGAGGAGGGGAGGGAGGGGAGGGGAGC | 51805417 |
| 482 | AAAAAAAAGAAAGAAAAAAGAAAAGAAAAAAGAAAAAAAGGAAAAGAAAGAAAAGAAAT | 51826568 |
| 483 | AAGGAAAGGAAAAAGGAAAGGAAAAGAAAGGAAAAAGGAAAGGAAAAGGGAAAGGAAAAAGGAAAGGAAAGGAAAGGAAAGGAAAGGAAAGGAAAGGAAAGGAAAGGAAAGGAAAGGAAAGGAAAGGAAAGGAAAAGAAAGAAAAC | 52036442 |
| 484 | AAAAAAAAAAAAAAAAAAAAAAAAGAAAAGAGAGAGAGAAGAGAAGAGAAGAGAAGAAAAGAAAAC | 52041333 |
| 485 | AAAGAAGGAAGGAAAAAGGGAAGGAAGGAAGGGAAGGAAGGAAGGGAAGGAAGGGAAGGAAGGAAGAGAAGGAAGC | 52133790 |
| 486 | AAGGGGGGAGGGAGGAAGAGGGAGGGAGGAAGGAAGGAAGGAAAGAAGGAAGGAAGGAAAT | 52133866 |
| 487 | AAAAAAAAAAAAAAGAAAGAAAGAAAGAAAAGAAAAGAAAAGAAAAAAGAAC | 52326587 |
| 488 | AAAAAGAGAAAAGAAAAGAAAAGAAAAGAAAAGAAAAGAAAAGAAAAGAAAAGAAAAGAAAGAGAGAAAGAGAGAGAGAGAGAGAGAGAGAGAAAGAGAGAAAGAGAGAAAGAGAGAGAGAGAAAGAGAGAAAGAGAGAAAGAGAGAAAGAGAAAGAGAGAAAGAAAGAAAGAAAGAAAGAAAGAAAGAAAGAAAGAAAGAAAGAGAAAAT | 52627055 |
| 489 | AAAAAAAAAAAAAAAAAGAAAGAAAGAGAAAGAAAAGAAAAAAAAAGAGGGAAGT | 52652360 |
| 490 | GAGAGAGAGAGAGAGAGAAAGAAAAAAGAAAAGGAAGGAAAGAAAGGAAAGAAAGAAAGAAAT | 52724865 |
| 491 | GGAGGGAGGGGGGAGGGGGGAAGGGGAGGAGGGGGGAGGGAGGGAGGAAAGAAGGAAGGAAGGAAGGAAGGAAGGAAGGAAGGAAGGAAGGAAGGAAGGAAGGAAGGAAGGAAGGAAGGAAAC | 52963642 |
| 492 | AAAAAAGAAAGAGAGGAAAGAAAAGAAAGAAAAGGAAGAAAGGGAAGGAAAGGAAGGAAGGGAAGGGAAGGAAGGAAGGAAGGAAGGAAT | 53025686 |
| 493 | AGAAAGAAAGAAAAAGAAAGAAGGGAAGGAAAGAGGGAAGGAAGGAAGGAAGGAAGGAAAAGC | 53136149 |
| 494 | AGAAAGAAAGAAAGAAAGAAAGAGAGAAAGAGAGAGAGAGAGAGGGAGGGAGGGAGGGAAGGAGGGAGT | 53762725 |
| 495 | AAAAAAAAAAAAAAAAAAGAAAGAAAGAAAGAAAGAAAGAAAGGAAGAAGGT | 53844801 |
| 496 | GAAGAAAGAAAGAAAGAGAGAAAGAGAGAAAGAAAGAGAGAGAGAGAAGGAAAGAAAGAAAGAAAAGAAAGAGAGAAAGAAAAAGAAAGAAAGGAGGGAGGGAGGGAGGGAAGGAAGGAAGGAAGGAAGGGC | 54283230 |
| 497 | AAAAAAAAAAAAAAAAAAAAGAAAAAAAAGAAAAAGAAAAAGAAAAAGAAAAAAAGAAAGAAGAAAAGAAAAGAAAGGAAAGGAAAAAAGAAAAGAAAAGAGGT | 54311154 |
| 498 | GAGGAAGGAAAGAAGGAGGAAGGGAAGGAGGGAGGAAAGGAAGGAAAGAAGGAAAGAAAAAAGAC | 54369341 |
| 499 | AAAAAAAAAAAAAGAAAGAAAGAAAAAGAAAGAAAGGAAGGAAGGAAGGAAGGAAGGAAGGAAGGAAAAAAGAGAGAAAT | 54449347 |
| 500 | AAAAAAAAAAAAAAAAAAAAAAAAAAAAGAAGAAGAAGGAAGGAAGGGAGGGAAGAAAGT | 54572407 |
| 501 | AAAAAAGAAAGAAGAAAGGAAAGAGAGAGAGAGAGAAAGAGAGGGGAAGGAAAGAAGGAAGGAAGGAAGGAGAAGGGAAGGGAAGGAAGGAAAGGAAGGAAGGGAAGGAAGGAAGGAAGGAAAGAAGGAAGGAAAGGGAAGGGAAGGGAAGGAAAGGAAGGAAAGGAAGGAAAGGAAGGAAAGGAAGGAAGGGAGGGAT | 54765685 |
| 502 | AAGAAAGAAAGAAAGAAAAGGAAGGAAGGAAGGAAGAAAGGAGAGAGAGAGAGAGAGAAAGAAAGAAAGAAAGAAAAGGAAGGAAGGAAGAAAGAAAAGGC | 54868358 |
| 503 | AGGGAAGGAAGGAAGGAAGGGAGGGGGGAAGGAAGGAAGGAAGGGAGAGAGAGAGGGAGGGAGGGAGGGAGGGAAGAAAAGT | 54980513 |
| 504 | AAGAAAGAAAGAGAAGGAAGGAAGGAAGGAGAAAAAGAAAGAAAAGAAAGAAAGAAGAAAGAAAGAAAGAAAGAAAGAAAGAAAGAAAGAAAGAAAGAAAGAAAAGAAAGAAAGAAAGAAAGGAAGGAAAAGAAAAGAAAAGAAAAGAAAAGAAAAGAAAAGAAAAAAAAAGAAAAAAAAAGAAAAC | 55249502 |
| 505 | AAAGGAAGGAAGGAAGAAAGGAAGAAAAGAAGGAGGGAGGGAGGGAGGGAGGAAAAT | 55251372 |
| 506 | AAAAAAAAAAAAAAAAAAAAGAAAGAAAGAAAGAAAGAAAGAAAGAAAGAAAAGAAAAAAGAAAT | 55413018 |
| 507 | AGGAAAGAGAGGGAAAAGGAAGGAAGGAAGGAAGGAAGGAAGGAAGGAAGGAAGGAAGGAAGGAAGGAAAAC | 55446078 |
| 508 | AAAAAAAAAAAAAAAAGAAGAGAGAGAGAGAAAGAAAGAAAGAAAAGAAAGAAAGAAAGAGAAGGAAGGAAGGAAAGAAAGAAAAGAAAGAAAAGAAAGAAGC | 55501861 |
| 509 | GAGGAGGGAGGGAGGGAAGGAAGGAAGGAGGGAGGGAGGAAGGGAAGGAAGGAGGGAAGGAAGGAAT | 55577552 |
| 510 | AGGAGGGAGGGAAGGAAGGAGGAAGGAAGGAAGGAGGGAAGGAAGGGAGGGAGGGAAGGAAAGAAGGAAGGGAGGAAGGAAGGAGGGAGGGT | 55577633 |
| 511 | AGGAGGGAGGGAAGAAAGGAAGGAAGGAGGGAGGGAAGAAAGGAAGGAAGGAGGGAGGGAGAGAGGAAGGAAGGGAGGGAGGGAGGAAGGC | 55577725 |
| 512 | AGGAAGGAGGGAGGGAGGGAAGGAAAGAAGGAAGGGAGGAAGGAAGGAAGGAC | 55577940 |
| 513 | AAAAGGAAGGAAGGAAGGAGGGAGGGAGAAGGAAGGGAGAAGGGAGGAAGGAAGAAAGGAAGAAAGAAAGAAAAAAAGAAAGAAAGGC | 55622739 |
| 514 | GAAAGAAAGAGAAAGAAAGAGAGAGAGAGAGAGAAAGAAAGAGGGAGAAAGAAAGAAAGAAAGAAAGAAAC | 55624590 |
| 515 | GGGGGAGAAAGAGAGAGAAAGAAAGAAAGGAGAGAGAGAGAGAGAGAGAGAGAGAGAGAGAGAGAGAGAGAGAGAGAAGAGGAGAGGAGAGAGAAGAGAGGAGAGAGGAGAGAGAGAGAGAGAGAC | 55855022 |
| 516 | GGGGGAGAAAGAGAGAGAAAGAAAGAAAGGAGAGAGAGAGAGAGAGAGAGAGAGAGAGAGAGAGAGAGAGAGAGAGAAGAGGAGAGGAGAGAGAAGAGAGGAGAGAGGAGAGAGAGAGAGAGAGAC | 56011885 |
| 517 | GAGAGAGAGAGAGAGAGAGGAGAGAGAGAGAGAGAGAGGAGAGAGAGAGAGAGAGAGAGGAGAGAGAC | 56202366 |
| 518 | AAAAAGAAAAGAAAAGAAAGGAAAAGAAAAGAAAAGAAAAAAGAAAAGAAAAGAAAGC | 56269730 |
| 519 | AGGGGGGAGGGGAGGGGAGGAGAGGAGAAGAGAAGGAGGAGGAGGAAGAGGAAGAGGAAGAAGAAAAGAC | 57259311 |
| 520 | AAAGAAGAAGGAGAAAAGGAAAAAGGAGAAGGAAAAGAGGAGAAAGAAAAGAGC | 57342777 |
| 521 | GGAAGGGAAGGAAGGAAAGAAGGGAGGGAGGGAGGAAGGAAGGAAGGGAGGGAGGGAGGGAGGGGAAAAAAGGAGGC | 57666407 |
| 522 | GGGAGGGAGAGAGGGAGGGAGGGAGGGAAAGAAGGAAGGAAGGAAGGAAGGGT | 57706966 |
| 523 | GGAAAGAGAGAAAGAGAAAGAAAGAAAGAAAGAAAGAAAGAAAGAAAGAAAGAAAGAAAGAAAGAAAGAAAGAAAGAAAGAGAAGGAAGAGAT | 57746820 |
| 524 | AGAGAGAAAGAAAGAAAGAAAGAAAGAAAGAAAGAAAGAAAGAAAGAAAGAAAGAAAGAAAGAGAGAGAGAGAGAGAAAGAGAGAGAGAGAGAGAGAGAGAGAGAGAGAGAAAGAAAGAAAGAAAGAAAAAAT | 57832687 |
| 525 | AAGAAAGAGAAAGAGAGAGAGAGGGAGGAAGGGAGGGAGAGAGAGAGAGAGGGAGAAAAGAAAC | 57865662 |
| 526 | GAGAAGGAGAGGGGAGGGGAGGGAGGAGGGGAGGGGAGAGGGAAGGGGAGAAGGAAAGGT | 58006039 |
| 527 | AAAGAGAGAGGAGAGAAAGAAGGAGGAGGAAGGGGAGAAGGAAGAGAAAGAGGAGGGGGAAGAGGGAAAGAAGGAAGAAGAGGAAGAC | 58085746 |
| 528 | AGAAAAGAAGAGGAAGAAGAAGAGGAAGAAGAAGAAGAGAAGAAGAAGGAGGAGGAGGAGGAGGAGGAGGAGAAGGAGGAGAAGGAGAAGAAAGAGAAGAAGGAGAAGGAGAAGAAGGAGAAGGAGGGGAAGGGGAAGAAGAAGAGGAAGAGGAAGAGGAGGAAGC | 58396832 |
| 529 | AGGGAAGGGAGGGGAAGAGAAGGAGAAAAGGAAAAGGGAGGAAAAAGAGGAAGAAGAT | 58564507 |
| 530 | AAAGAGGAAAGGAAGGAAGAAAGAAAGGAGAGAGGGAAGGAAGAAAGGAGGGAGGGAGGGAGGAAGGAAGAAT | 58716252 |
| 531 | GGGAAGAAGGAAGAAAAGGAGGAAGGAAAGAAGAGAGAAAGAGGGAGGGAGGAAGAGAGGAAGGGAAAAAT | 58728580 |
| 532 | GAAAAGGAAGAAAGAAAAGAAAAAAGAAGGAGGGAGGGAAGGAGAGAAGGAAGAAT | 58728718 |
| 533 | AGAGAAAGAGAGAGAGAAAGAAAGAGAAGGAAGGAAGGAAGAAAGAGAAAGAAAGAAAGAAAGAAAGAAAGAAAGAAAGAAAGAAAGAAAGAAAGAAAGAAAGAGAAAGAAAGAAAGAAAGAGAAAGGGAAAGAAAGGAAGGAAGGAAGGAAGGAAGGAAGGAAGGAAGAGAGAGAGAGAAGAGGC | 58829077 |
| 534 | AAAAGGAGGGAGAGGGAGAGGGAGAGGGAGGGGGAAGGGGAGAGGGAAGGGGAGGGGGAGAGAGAGAGGGAGAGGGAGAGGGC | 58942582 |
| 535 | AAGAAAGAAAGGAAGAAAGAAAGAAAGAAAGAAGGAAGGAAGGAAGGAAGGAAGGAAGAAAGGGAGAGAGAAGGAAAGAAAGAAAGAAAGAAAGAAAGAAAGAAAGAAAGAAAGAAAGAAAGAAAGAAAAGAAAGAAGAAAGAAAGAAAGAAAGAAAGAAAGAAAGAAAGAAAGAAAGAAAGAAAGAAAGAAAGAAAGAAAGAAAGAAAAAAGAAGAAAAGAGC | 58970964 |
| 536 | AGGAGGAAGAAAGAAAGGAAGGGAGGGAGGGAGGGAAAAAGGAAGGAAGGAAGGAAC | 58999967 |
| 537 | GAAGGAAGGAAGGAAGGGAGGGAGGGAGGGAGGAAGGAAGGAAGGAAGAGAGGGGAGGGGAGGGGAAGGGAGGGAAGGGAGGGAGGC | 59000024 |
| 538 | AAAAAAAAAAAAAAAAAAAGAAAGAAAGAAAGAAAAGAAAGAAAGAAAAAAGGC | 59088057 |
| 539 | AGAAGGAAGAAAGGAAGGAAAGAAGAAAGGAGGGGAAGAAGGAAGGGAGGAAGT | 59198102 |
| 540 | GAAGAAGGAGGAGGAGGAAGAGGAAGAAAAGAAGAAAGAGGAGGAAAAGAAGGAGGAGGAGAAT | 59257037 |
| 541 | AAAAAAAAGAAAGAAAGAAAGAAAGAAAGAAAGAAAGAAAGAAAGAAAGAAAGAAAGAAAGAAAGAAAGAAAGAAAGAAAGAAAAAAAAT | 59259917 |
| 542 | AAAGGAAGAAAAGAAGGAAAAGAAGGAAGGAGGAAGAGAGAGAAGAAAGGGAGGAAAGGGGAGT | 59275643 |
| 543 | GGGGAGGAGGGAGAGAGAAAAGAGAAGAAAGAAAGGGAGGAAGAGAGGGAGGGAGAAAGGGAGGGAGGAAAGGAGT | 59275707 |
| 544 | GAGAAAGAAAAAAAAGAAAGAAAGGAAAGAAAGAAAAAAGAAAGAAAGAAAGAAAAAAGAAAGAAAGAGAAAGAAAGAGAAAGAAAGAAAGAAAGAAAGAAAGAAAGAAAGAAAGAAAGAAAGAAAGAAAGAAAGAAAGAAAGAAAAGT | 59571856 |
| 545 | GAAAAGAAAGAAAGAAAGAAAGAAAGAAAGAAAGAAAGAAAGAAAGAAAGAAAGAAAGAAAGAAAGAAAGAGAGAGAGAGAGAGAGAGAGAGAGAGAGAGAGAGAGGAAGGAAGGAAGGAAGGAAGGAAGGAAGGAAGGAAGGAAGGAAGGAAGGAAGGAAGGAAGGAAGGAAGAGAAAAC | 59576280 |
| 546 | GGGGGAAAAAGAAGAAAGGGAGGGAGGAAGGAAGGAAGGAAGAGGAGAAGGAAGAGGAGGAGGC | 59603011 |
| 547 | GAAGGGGAAGAGGAGGGAAAGGAGGAGGGGGGGAGAGAGAGGGAGAGAGGGT | 59678359 |
| 548 | AGAAAGAAAAGAGGAGAGGGGAGAGGGGAGAGGGGAGAGGGGAGAGGGGAGAGGGGAGAGGGGAGAGGGGAGAGGGGAGAGGAGAGAGGAGAGGAGAAGAGAAGAGAAGAGAAGAGAAGAGAAGAGAAGAGAAGAGAAGAGAAGAGAAGAGAAGAGAAGAGAAGAGAAGGGAAGGGAAGAGAAGAGAAGGGAAGAAAAGAAAAGAAAAAAGAAAAGAGAAT | 59793922 |
| 549 | AAAGAAGAAAGAAAGAAAAGAAAAGAAAAGAAAAAAAAGAAAGAAAAGAAAAGAAAAGAAAAAGAAAAGGT | 59989557 |
| 550 | AAAGGAAAAAAGAGAGGGAGGGAGAGAGGGAAGAAGGGAGGGAAGGAGAAAGGAAGGAAGAAGT | 60136857 |
| 551 | GAGGGAGGAAGGGAGGAGGGAAGGGGGAGGAAGAAGGGAAGGAAGGAAGAGAGAGGGAGGC | 60137075 |
| 552 | AAAAAAAAGAAGAAAGAAAGAAAGAAAGAAAGAAAGAAAGAAAGAAAGAAAGAAAGAAAGAAAGAAAGAAAGAAAGAAAGAAAGAAAGAAAGAAAGAAAGAAAGAAAGAAAGAAAGAAAGAAAGAAAGAAAGAAAGAAAGAAAGAAAGAAAGAAAGAAAGAAAGAAAGAAAGAAAGAAAAAAAC | 60256837 |
| 553 | AAAAAAAAAAAAAAAAAAAAGAAAGAGAGAGAGAGGAAGGAAGGAAGGAAAGAAGGAAGGAAGGAGAT | 60361756 |
| 554 | AAAGAAAGAAAGAAAGAAAGAAAGAAAGAAAGAAAGAAAGAAAGAAAGAAAGAAAGAGAAAGAAAGAAAGAAAGAAAGAAAGAAAGAAGGAAAGAAAGAAAGAAAGAAAGAGAAGGAGAGAGGGAGGC | 60492387 |
| 555 | AAGGGGGGGAGAGAGAGAGAGAAAGAGAAAGAAGGAAAGAGAGAAAGAGAAAGAAGGAAAGAAAGAAAGAAAGAAAGAAAAGAAAGAGAAAGAAGAGAGAAGAGAAGAGAAGAGAAGAGAAAAGAAAAGAC | 60492532 |
| 556 | AAAGAAAGAGGAAGAGAGAGAGAAAGAAAGAGAGAAAGAAAAAAAGAAAGAAAGAAGAAAGAAAGAAAGGGAGGAAGGAAGAGGGGAAGGGAAGGGAAAGGAAAGGGAGGGGAGGGGAGGGGAC | 60513009 |
| 557 | GGAGAAGGAGAAGAGGAAGGAGAAGAAGAGGAAGGAAGAGGAGGAGGAAGAGC | 60969372 |
| 558 | GAAAGAGAAAAAGAGAGAAAGAGAGAAAGGAAAGAAAGAAGGAGGGAAGGAAGGAAGGAAGGAAGGAGC | 61134455 |
| 559 | GGGAGGGAGGGAAAGGAAGGAAAGGAAAGGAGAGGAGGAGGGAGGGGAGGGGAGGGGAGC | 61134526 |
| 560 | AGGGAAGGGAAGAGAAAAGAAGGGAAGAGGAGGAGAAGGGAGGAAAGGGGAT | 61874102 |
| 561 | GGGAGGAAAAGGGAGGGGAGGGGAGGGGGAAAAGGAAAGGGAAGGGAAGGGAAAAGAAGGAAAGGGGAGGGGAGAGAAGAGGAC | 61874184 |
| 562 | GGGGGGAGAAGGGAGGGAAGGGAAGGGAAGGGAAAAGAAGGAAAGGGGAGGGAAAAGAAGGAAAGGGGAGGAGAGAGAAGAGGAC | 61874274 |
| 563 | GGGAGGGAAGGGGGAGAAGGGAGGGAAGGGAAAGGAAAAAAGGGAAGGGGAGGGGAGAAGAGGGGAGGGAAAGGAAGGAAGGAGAAAGAGAGGAAGAAT | 61874359 |
| 564 | AAGAAAAAGAGGGAAGAGAGGAGGGAAGGAGAGAAGGAAAGAGGGAAGAAAGAAAAAT | 61874501 |
| 565 | GAAAGGAAAGAGAAAGGAAGGAAGAAAGGAAAGAAGGAAGGGAAGGGAAGGAAAGGGAAGGGGAGGGGAGGGGAGGGGAGGAC | 61985235 |
| 566 | AGAAAAGAAAGAAAGAAAAAGAAAGAAAGAAAGAAAGAAAGAAAGAAAGAAAGAAAGAAAGAAAGAAAGAAAGAAAGAAAGAAAGGAAGGAAGGAAGAAAGAAAGAAAAAGAAAGAAAGAAAGGAAGAAAGAAAAAAAGAAAAC | 62157481 |
| 567 | GGAAGGAAAGAGGGAAGGAAGGAAAGAAGGAAGGAAGGAAGAGAGGGAGGGAGGGAGGGGAGAAAGAGAT | 62195547 |
| 568 | GAAAGAAAGAAAAAAGAGAGAAAGAAAGAAAGAGAGGAAAGAAAGAAAGAAAGAAAGAAAAAGAAAGGAAGGAAGGAGAGAGAGAAGAAAGAAAAAAGGAAGGAAAGAAAGAGAGAGAAAGAAAAAGAAAGAAAGAAAGAGAAAGAAAGAAAAGAAAGAAAGAAAGAAAGAAAGAAAGAAAGAAAGAAAGAAAGAAAGAAAGAAAGAAAGAAAGAAAGAGGAAAT | 62301047 |
| 569 | GAGAGAGAGAAAGGGAGGGAGGAAGGAAGGAAAGGAAGGGAAGGAAGGAAGGAAGGAAGGAC | 62597142 |
| 570 | AAAAAAAAAAAAAAAAGAAAAGGAAAAAAGAAAGGAAGGAAGGAAGGAAAGAAGAGAAAGAAGAAAGGAGAGAAAGAAGGAAAGAAAGAAGAT | 62944685 |
| 571 | GGAAAGAAGGAAGGAAGGAAGAAGAGAGAAAGAAAGAAAGAAAAAGAAAGAGAAAGAGAAAAGAAAGAAGGAAAGAAAGAGAAAGAAAAGAAAAAAAAGAAAGAGAGAAAGT | 62968190 |
| 572 | AAGAAAGAGAGAGAGAGGGAGGGAAGGAAGGGAGGGAGGGAGGGGAGGGGAGGGGAGGGAAGAGGGGAGGGGAAGGT | 63188372 |
| 573 | AAAAAAAAAAAAAAAAAAAAAAAAAAAAAAAAAAAAAAAAAAAAAAAAAAAAAAAAAAAAAAAAAAAAAGAC | 63200937 |
| 574 | AAAAAAAAAAGAAGGAAGAAAGGAAGGAAGGGAGGGAGGGAGGAAGGGAGGAAGGGAAAGAAAAAAGAGAAAGAAAGGC | 63312609 |
| 575 | GAAGAAAAAAGAAAGGAAGGAAGGAAGGAAAGGAAGGAAGGGAGGGAGGGAGGGAGGGAGGGAAAGGAT | 63321934 |
| 576 | GAAGAAAGAAAGAGAGAAAGAGAGAGAGAGGGAGGGAGGGAGGGAGGAAGGAAGGGAGGAAGGAAGGGAGGAAGGAAGGAAGGGGAAGGGGAAGGGGAAGGGAAGGGAAAGGAAAGGAAGGGAAGGAAGGAAGAGAGAAAGAAAGAAAT | 63551552 |
| 577 | AAGAAAAAAAAAAAAGAAAAGGAGAAAGGAGAAAGAAAGAAAGGAAAGAAAGGAAAGAAAGGAAAAGAAAGAAAGAAGAAAGAAAAGAAAAGAAAAGAAAAGAAAGGAAAAGAAGC | 63600185 |
| 578 | GGGGAAGGGAGGGGAGAAGAGGGAAAGGGAGAGAAGGGGAAAGGGAGGGAAAGGGAGGGGT | 63625156 |
| 579 | GGGGAGGGGAGGGGGAAGGGAAGGGAGAGGAAGGGAGGGAGGGAGGGGAGAGAAAGGGAGGGGAGAGGAGGGT | 63625307 |
| 580 | AAAAAAAAAAAGAAAAAAAAGAAAAGAAAGAAAGAAAGGAAAGAAAGAAAGAAAC | 63691063 |
| 581 | GAGGAGGAGAAGAAGGAAGAAAGGAGGAAGAGAGAGGAAGAAGAGGGAGGAAGGAT | 63896841 |
| 582 | AGGAAGGAAGGAAGGAAGGGAGGGAGGGAGGAAGGGAGGGAGGAAAGGGAGGGAGGGAAAAGAAAAGAGAAAGGAAGGAAAGAGAGAGGGAGAGAGGGAAGGAAGGAAGGGAAGGGAAGGAAGGAAAT | 63994282 |
| 583 | GAGGAGGAGAAGAAGGAAGAAAGGAGGAAGAGAGAGGAAGAAGAGGGAGGAAGGAT | 64053703 |
| 584 | AGGAAGGAAGGAAGGAAGGGAGGGAGGGAGGAAGGGAGGGAGGAAAGGGAGGGAGGGAAAAGAAAAGAGAAAGGAAGGAAAGAGAGAGGGAGAGAGGGAAGGAAGGAAGGGAAGGGAAGGAAGGAAAT | 64151144 |
| 585 | GAGAAAGAAAGAAAGAAAGAAAGAAAGAAAGAAGAAAGAAAGAAGGAAAGAAAGAAAGAAAGAAAC | 64530427 |
| 586 | AAAGAAAGAAAGAAAGAAAGAGAAAAAGAAAGAAAGAAAGAAAGAAGGAAAAGAAGGAT | 64530493 |
| 587 | AAGAAGAAAGAAAGAAAGAAAGAAAGAAAGAAAGAAAGAAAGAAAGAAAGAAAGAAAGAAAGAAAGAAAGAAAGAAAGAAAGAAAGAAAGAAAGAAAGAGAAAGAAAAAGAAAAAGAAAGAAAGAAAGGAAGGAAGGAAGGGAGAGAGGAAGGAAGGAAGGAGAAAGAGAGAAAGAAAGAAAC | 64609956 |
| 588 | AAAAAAAGAAAGAAAGAAAGAAAGAAAGAAAGAAAGAAAGAAAGAAAGAAAGAAAGAAAGAAAGAAAGAC | 64619335 |
| 589 | AAAAAAAGGAAAAAAGAAAAGAAAAGAGAAAGAAAGGAAGGGAAAAAAGGAAAT | 64657098 |
| 590 | AGGAGAAAGAGAAGGAGGAGGAGGAGGAAGAGGAGGAGGGGGAAGAGGAGGAGGAGAAGGAGAAGGAAAAGGAGAAGGAGGAGGAAGAGGAGGAGAAGGGGAAGAAGGAGAAGAAGGAGGAGGAGGAGAAGGAGGAGGAGGT | 64699587 |
| 591 | AAAAAAAAAAAAAAAAAAAAAGAAAAGAAAAAAAGAAAGAAAGAAAGAAAAAAAT | 64773373 |
| 592 | GAAGGAAAGGAAAGAAGGAAGGAAGGAAGGAAAGGAAGGAAGGAAGGAAGGAAAGGAAGGAAGGAAGGAAGGAAGGAAGGAAGGAAGGAAGGAAGGAAGGAAGGAAGGGC | 64788951 |
| 593 | AAGAAGGAAGGAAGGAAGGGAGGGAGGAAAGAAGGAAGGAAGGAGAAGAAAGGAAGAAAGAAAGAGGGGAGGGAGAGAGAGAAGAAAGAAAGAAAGAAAAAGAAAGAAAGAAAGAAAGAAAAAGAAGGAAGGAAGGAAAGAAGGAAAGAAGAAAAAGAAAGAAAGGAAAT | 65142037 |
| 594 | AAAAAAAAAAAAAAGAAAAAAGAAGAAAAAGAAGAAGAAGAAGAGGAAGAAGAAGAGAAAGAAGAAGAAGAGGAGGAGGAGGAGGAGGAAGAAAAGGAAGGGAAAAGAAAAGAAAAAT | 65163768 |
| 595 | AGGAAGAAGAGGAGAAAGAGAAGAAAAAGAAGGAAAAGAAGAAAAGGAAGAGGAGGAAGGGGAC | 65174656 |
| 596 | AAAAAGAAGAAGAAGAAGAAGAAGAGGAAGAGGAAGAGGAAGAGGAAGAGGAAGAAGAAGAAGAAGAAGAAGAAGAAGAAGAAGAAGAAGAAGAAGAAGAAGAAGAAGAAGAAGAAAGAAGAAGAAGAAGAAGAAGAAGAAGAAGAAGAAAGC | 65671653 |
| 597 | AAGAAAAGGAAAAAGAAAAAGAGAAAGAAGAGAAGAGAAGAGAAGAAGGGAGGGAGGGAGGGAAAGAAAGAAAAAAAGAAAGAAAGGAAGAAAAAGAAAGAAAGAAAAGAAAGAAAGAAAGAGAAAGAAGAAAGAAAGAAGAAAGAAAAGGT | 65734948 |
| 598 | GAAAGAGAGGAAGGAAGGAAAGAAGGAAGGAAGGAAGGAAGGAAGGAAGGAAGGAAGGAAGGAAGGAAGGGAAAT | 65794733 |
| 599 | AAAAAAAAAGAAAAAGAAAGAAGAAAGAAAAAGAAAGAAAGGAAAGAAAAGAAAGAAAGGAAAGAAAGAAAGAAAGAGAGAAAGAAAGAAAAGAAAGAAAGAC | 65826558 |
| 600 | AAAAAGAAAGAAAGGAAAGAAAGAAAGAAAGAAAGAAAGAAAGAAAGAAAGAAGAAGAAAGAAGAAAAAGAAAGAGAGAAAGAAAAGAAAGAAAGAGAAGGAAAGAAAGAAAGGAAGAAAGGAAGAAAT | 65830839 |
| 601 | AAAAAAAAAAAAAAAAAAAAAAAAAAAAAAAAAAAAAAAAAAAAAAGAAAAAC | 65849442 |
| 602 | GAGGGAGGAGGAGGGGGGGAGGGGGAGGGGGGGAGGAGGAGGGGGGAGGGGGAGGGGGAGGGGGAGGGAGGAGGAGGAGGAGGAGGAGGAGGAGGAGGAGGAGGAGGAGGAGGAGGAGGAGGAGGAGGAAAGC | 66330073 |
| 603 | GGGAGGGAGGGAGGGAGGAAGGGAGGAAGGAAGGAAAGAAGGAAGGAAGGAAGGAAGGAAGGAAGGAAGGAAGGAAGGAAGGAAAGAAGGGAGGGAGGGAGGGAGGGAGGGAGGGAGGAAT | 66346229 |
| 604 | AAAAAGAAAGAAAGAAAAAGAGAAAGAAAGAAAGAAAGGAAGAAAGGAAGAAAAAGAAAGAAAGAAAGAAAAAGAAAGAAAGAAAGGAAAAGAAAGAAAGAAAGGAAAAGAAAGAAAGAAAGAAGGAGAAAGAAAGAGAGAAAGAAAGAC | 66405728 |
| 605 | GAGAAAGAAAGAGAGAGAAAGGAAGGAAGGGAGGGAAGGAGGGAGGGGAGGGGAAGGGAAGGGAAAGGAAGAGAAC | 66405903 |
| 606 | GGAAGGAAGAAAGGAAGAAAGGAAGGAAGGGAGGGAGGGAGGGAGGGAGGGAGGGAGGGAC | 66461065 |
| 607 | AAAAAAAAAAAAAAAAAAAAAAAAAAAAAAGAAAGAAAGAAGAAAGGAAAGGAGAAAAGGAAAAAGAAAGGAAAT | 66516710 |
| 608 | AAAAAGAAAAAGAAAGGAAGGAAGGGAGGAAGGGAGGGAGGGAGGGAGGGAGGGAGGAT | 66962130 |
| 609 | AAAGAAGGAAAGAAAAAAAGAGAGAGAGAGAAAGAAAGAAAAGGAAGAAAGAAAGAGAGAGAAAGGGAAAGGAAAGGAAAGGAAGGAAGGAAGGAAT | 67273914 |
| 610 | AAAAAAAAAAAAAAAAAAAAAAAAAAAGAAAGAAAGAAAGAAAAAGAAAGAAAGAAAGAAAAGAC | 67597146 |
| 611 | AAAAAAAAAAAAAAAAAAAAAAAAGAAAAAGGAAAAAAAGAAAAGAAAAGAAAGT | 67643206 |
| 612 | GAAAGAAAGAGAGAGAGAGAGAAAGAAAGGAAAGGAAGAAAGAAGGAAGGAAGGAAGGAAAGAAGGAAGGAAGGAAGGAAAGAAGGAAGGAAGGAAGGAAAGAAAGT | 67877166 |
| 613 | GGAAAGAAAGAAAGAAAGAAAGAAAGAAAGAAAGAAAGAAAGAAAGAAAGAAAGAAAGAAAGAAAGAAAGAAAGAAAGAAAGAAAGAAAGAAAGAAAGAAAGAAGGGAGGGAGGGAGGGAGGAAGGAAGGAAGGAAAGAAAGAGAGAAAGAAAGAGAAAGAAAGAAAGAGAGGAAAGAGAGGAAGAAGGAAGGAAGGGAAAGGAAAGGAAGGAAGGGAAAGGAAAGGAAGGAAGGAAGAAAGGAAGAAAGAAAGAAAGAAAGAAAGAAAGAAAGAAAGAAAGAAAGAAAGAAAGAAAGAAAAAAAGAAAGAC | 68063412 |
| 614 | AGAAAGAGAAAAAGAGAGAAAGAAAAAGAAAGAAAAAGAAAGAAAGAAAGAAAGAAAGAAAGAAAGAAAGAAAGAAAGAAAGAC | 68092527 |
| 615 | AGGAAGGAAGGAAGGAAGGAAGGAAAGAAAGAAAGAAAAGAAAGAAAGGAAGAAAGGAAGGAAGGAGGGAGGGAAGGAAGGAAGGAAGAAGGAAGGAAGGAAGGAAAGAAAGAAAGAAAGAAAGAAGAGAGAAAGAAAGAAAGAAAGAAAGAAAGAAAGAAAGAAAGAAAGAAAGAAAGAAAGAAAGAAAGAAAGAAAGAGAAAGAAAGAAAGAGGGAGGGAGGGAGGGAGGGAGGAAGGAAGGGAGGAAAGAGC | 68092627 |
| 616 | AGAAAGAAAGAAAGAAAAGAAAGAAAGAAAGAAAGAAAGAAAGAAAGAAAGAAAGAAAGAAAGAAAGAAAGAAAGAAAGAAAGAGAGAGAGAGAGAGAGAGAGAGAGAGAAAGAAAGAAAGAGAGAGAGAGAAAGAAAGAAAGAAAGAAAGAAAGAAAGAAAGAAAGAAAGAAAGAAAGAAAGAAAGAAAGAAAGAAAGAAGGAAAGAGGGAAGGAAGGGAGGAAAGT | 68268736 |
| 617 | AAAAAAAAAGAGGAAAGAAGAAAGAAGAAGAGGAAGAAGAAGAAGAGGAAGAGGAGAAGGAAGC | 68323214 |
| 618 | AGAGGAGGAAGAGGAGGAAGAGGAAGAAGAAGAAGGAGAGGAGGGGGAGGAGGAGGAGGGAGAGGAAGAGGGGGAGAAGGAAGAGGAGGAGGAGGAAGAGGAAGAAGAGGAAGAGGAGGAAGAAGAAGT | 68323278 |
| 619 | GAGGAAGAAGAGGAAAAGAAGAGGAAAAGAGGAAGAGGAAGAAAAGGAAGAGGAAGAAGAAGAGGAAGAGGAAGAAGAAGAAGAAGAAGC | 68323407 |
| 620 | AAAAAAGAAAGAAAGAGAGAAAGAGAAGAAAGAGAAGAAAGAGAAGAAAGAAAGAAAGAAAGAAAGAAAGAAAGAAAGAAAGAAAGAAAGAGAGAAAGAGAGAGAGGAAAGAAAGAAAGAAAGAGAGGAAAGAAAGAAAGAAAGAAAGAGAGAGAGAGAAAGAAAGAAAGAAAGAAAGAAAGAAAGAAAGAAAGAAAGAAAGAAAGAAAGAAAGAAAC | 68325518 |
| 621 | AGAGAAAGAGAAAGAAGAAAGAAGGAAAGAAAAAAGAAAGAGAAAGAAAGAAAGAAAGAAAGAAAGAAAGAAAGAAAGAAAGAAAGAAAGAAAGAAAAGAAAGGAAGGAAAGAAAGAGAAAGC | 68774100 |
| 622 | AAGGAAAAAAAAAGAAAGAGAGAGAGAAGAAAAAGAAAAAGAAAGAAGGAAGGAAGGAAAGGAAC | 68800005 |
| 623 | AAAGAAGGAAGGAAAGAAGGAAGGAAGGGGGAGGGAGGGAGGAAGGGAGGGAAGGAAGGAAGGGAAGGAAGGAAAAGT | 68800099 |
| 624 | AAAAAGAAGGAAGGAAGGAGAAAGAAAGGAAGGAAGGAAGGAAGGAAGGAAGGAAGGAAGGAAGAAAGAAAGAAAGAAAGAAAGAAAGAAAGAAAGAAAGAAAGAAAGAAAGAAAGAAAAGGAAAGAAGGAAAGAAAGGAAGGAAGGAAGGAAGGAAGAAAAGAAAAAAGAGAAAAGAAAAAGAGGGAGAGAGAGGGGGAGGGAGGGAGAGAAAAGAAGAAAGAAT | 68959104 |
| 625 | GAGAAAGAGGAAGAAAGAGAGAAAGAGAGAGAGAAAAGAAGAAAGAAAGAAAAGAAAGAAAGAGAAAGAAAGAAAGAAAGAAAGAAAGAAAAGAAAGAAAGAAAGAAAGAAAGAAAGAAAGAAAGAAAGAAAGAAAGAAAGAAAGAAAGAAAAGAAAGAAAGAAAGAAAGGAGGGGAGAGAAAGAAAGAAAAT | 69064013 |
| 626 | AAAAAAGAAAAGAAAAGAAGAAAAGAAGAAAGGAAGGAAGAAAGGAAGGAAGGAAGGAAGAAAGGAAGGAAGGAAGAAAGAAAGAAAGAAAGAAAGAAAC | 69106102 |
| 627 | AAAGAAAGAAAGAAAAAGAAAGAAAGAAAGAAAGGAAGGAAGGAGAGAAGGAAGGAAGGAAGC | 69106234 |
| 628 | GGAAGAAAAGGGAGGAAGGAAGGGAAGGGGGAAGGAAAAAAGAAGGAAGGGGGGT | 69234516 |
| 629 | AAGAAAGAAAGAGAGAAAGGAAGGGAGGGAGGGAAGGAGGGAGGGAGGAAAGGAGAAAAGGAAGGAAGGAGAAAAAAC | 69362885 |
| 630 | AGGAGAGAGAGAGAAGAAAGGAGAGAGAGAGGAGAAAGGAGAGAGAGAGAGAGAC | 70123733 |
| 631 | AAAAAAAAAAAAAAGAAAAAGAAAAAAAAGAAAAAAAAAGAAAAAGGAAAAGAGAAAGAAAAGAAAAT | 70339067 |
| 632 | AAGGAGGAAGGAAGGAAGGAAGGAAAGAAAGAAGGAAAGAAAAGAGGAAGGGAGGAAAGGAGGGAAGGAGGAAGGGAGGGAGGGAGGGAAGGAAAAGAAGAAAAGGAT | 70635333 |
| 633 | AAAAAGAAAGGAAGAAGAAGAAGGAGGAGGAGGAGAGGAAGGAAGGAAGGAGAGAGGGAGAGAGGGAGGGAT | 70809088 |
| 634 | GAAAAAAAGAAAAAAAGAAAGAAAGAAAGAAAGAAAGAAAGAAAGAAAGAAAGAAAGAAAGAAAGAAAGAAAGAAAGAAAGAAAAAAGAAAGAAAGGAAAGAAAGAAAGAAAGAAAGAAAGAGAGAAAAAGAGGAGGGAGGGAAGGAGGGAGAGAAAGAGAGAAAAAAAGAAAAGAAAGAAAGAAAGAAAGAAAGAAAGAAGAAAAAGAAAGAAAGAGAAAGAAAGAGGAGGGAGAGAAAGAAAGAAAAGAGAAGC | 71150368 |
| 635 | AAGAGAAAGAGAAGAGAGAGAAAAAGAAAGAGAAGAAAGAGAGAAAAAGAAAGAAAGAAAGAAAAGAAAGAAAGAGAGAGAAAGAAAGAAAGAAAGAAAGAAAGAAAGAAGAAAAGAAAAGAAAGAGAGAGAGAAAGAAAC | 71150675 |
| 636 | AAAAAAAAGAAGAAAGAAAGAAAGAAAGAAAGAAAGAAAGAAAGAAAGAAAGAAAGAGAGAGAGAGAGAGAGAGAGAGAGAGAGAGAAAGAAAGAAAGAAAGAAAGAAAGGAAGGAAGGAAGGAAGGAAGGAAGGAAGGAAGGAAGGAAGGAAGGAAGGAAGGAAGGGAGAGAAAGAAAGAGAAGGAAGGAAGGAAGAAAGAAAAGGAAGGAAGGAAGGGAAAGAAAGAAAAAGAAGAAAGAAAGAAAGAAAGAAAGAAAGAGAAAAGAAAAGAAAGAAAGAAAGAAAAAGAAAGAAAGAGAAAT | 71214290 |
| 637 | GAGGAGAGGAGGGGAGGGAAGAGGAGGGGAGGGGAGGGAAAGGGAGGGGAGGGGAGC | 71410897 |
| 638 | GGAAGAGAAGAGAAGAGAAGAGAAGAGAAGAGAAGAGAAGAGAAGAGAAGAGAAGAGAAGT | 71620448 |
| 639 | AAAAAAAAAAAAAAAAAAAAAAAAAAAGAAAAGAAAAGAAAAGAAGAAGGAAGGAAGGAAGGAAT | 71698435 |
| 640 | GGAAGGAAAGAGAGAGGGAGGAAGGGAGGGAAGAAGGAAGGAAGGAGGAAGC | 71698519 |
| 641 | AAAAAAAAAGAAAGAAAGAAAGAAAGAAAGAAAGAAAGAAAGAAAGAAAGAAAGAAAGAAAGAAAAAGAAGC | 72381480 |
| 642 | AAAAGAAAGAGAGAGAGAGAGAGAGGAGAAGAAAAGGAAGAAAAAGAAAAAAAAGAAGAGAAGAAGAGGAAGAAGAAGAAGGAGAAGAAGAAGAAGAAGGAGGAGGAT | 72403297 |
| 643 | AGAAAGAAAGAGAGAGAGAGAAAGAAAGAAAGAGAGGGAGGGAGGGAAGAAAGAAAGAAAGAAGGAAAGAAAGAAAGAAAGAAAAAAAAGAAAGAGAAAGAGAGAAAGAAAAAAAGAAAGAAAAGAGAGAGGAGGAGAGGGAAGGGAAGGGAAGGGAAAGGAAAGGAAAGGAAGGGAAGGGT | 72629509 |
| 644 | GAAGAGAAAAAAGAAAGAGAAGGGAGAGAAGAGAAAGAAAAAGGGAGAGGAGAGAAAGAAAC | 72707856 |
| 645 | AAAGGAAAGAAAGGAAAAGAAAAGAAAAGAGAGGAAGGAGAAAGAAAGAAAGAAAGAAAGAGAGAGAGAAGGAAGGAAGAGAGGAAGGAAGGAAGGAAGGAAGC | 73295250 |
| 646 | AAGGAAGGAAGGAAGGAAAGAAGGAAGGAAGGAAGGAAGGAAAGAAGGAAGGAAAT | 73466059 |
| 647 | AAAAAAAAAAAAAAAAAAAAAAAAAAAAAAAAAAAAAAAAGAAGAAGAAGAAC | 73507690 |
| 648 | AAAAAAAAAAAAAAAAAAAAAAGAAAGAAAGAAAGAAAAAAGAAAGAAAAAAAAAC | 73739353 |
| 649 | AAAAGAAAGGAGGGAGGGAGGAAGGAAGGAAGGAAGGAAGGAAGGAAGGAAGGAAGGAAGGAAGGGAAGAAGGAAGGAAGGAGAAAAC | 74058960 |
| 650 | AGAAGGGGGAAAGAAGGGAGGAAGAAAGGAAGGGAGGGAGGGAAGGAGGAGGAAAGGAAGGAAAAAAAGAAGAAT | 74060712 |
| 651 | AGAAAGAAAGAAAAAGAAAGAAAGAAAGAAAAAGAAAGAAAGAAAGAGAAAGAAAGAAAGAAAGAAAGAAAT | 74433013 |
| 652 | GAAAGGAGAAAGAGAGAGAGAGAGGAGGAAGGAAGGAAAGGAGGAAGGAAGGAAGGAAGGAAGGAAGGAAGGAAGGAAGGAAGGAAGGAAGGAAGGAAGGAAGGAAGGAAGGAAGGAAGGAAAGAAGAAGC | 74459231 |
| 653 | AAAAAAAAAAAAAAAAAAAAAAGAAAAAGAAAAAAGAAAAAAGAAAAAAGAAAAGAGAC | 74892871 |
| 654 | GGAAAGGAAGGAAGGAAGGAAGGAAGGAAGGAAGGAAGGAAGGAAGGAAGAAAGAAAGAAAGAAAGAAAGAAAGAAAGAAAGAAAGAAAGAAAGGAAAGAAAGAAAGAAAGAAAGAAAGAAAGAAAGAAAGAAAGAAAAGAAAAAGAAAGAAAGAAAGAAGAAAGAC | 75309444 |
| 655 | GGGGGAGGGAGGAAAGGAAGAAAGGGAGGGGAGGGGAGGAGAGGGAAGGGAAAAGGAAAGGAAC | 75416447 |
| 656 | GGGAGGGAGAGAGGGAGGAAGGAAAGAAGAAAGGAAGGAAGGAAGGAAAGAAAGAAAGAAAGAAAAAAAGC | 75444389 |
| 657 | GGAAAGAAGGAAGGGAGAAAGGAAGGAAGGAAGGAAGGAGGGAGGGAGGGAGGGAAGGAAAAAGC | 75728733 |
| 658 | AAAAAAAAAAAAAAAAAAAAAAAAAAAAAAAAAAAAAAAAAAAAAAAAAAAAAAAAT | 76090078 |
| 659 | GAGAGAGAGAGAGAGAGAGGAGAAAGAGAGAGAGAGGAGAAAGAGAGAGAGAGAGT | 76242895 |
| 660 | AAAAAAAAAAAAAAAAAAAAGAAAAGAAAAGAAAAGAAAGAAAGAAAAGAAAGAAAT | 76571356 |
| 661 | GAAAGAAAGAAAGAAAGAAAGAAAGAAAGAAAGAAAGAAAGAAAGAAAGAAAGAAAGAAAGAAAT | 76907846 |
| 662 | GAGAGAAAGAAAAGAAAGAGAAAGGAAGGAAAGGAAGAGAAAAGAAAGAAAAAGAAAGAAGGAAC | 76930953 |
| 663 | AAAAAAAAAAAAAAAAAAAAAAAGAGAGAGAGAGAGAAAGGAAAAGAAAAAGAAAAGAAAT | 76941322 |
| 664 | AAAAAAAAAAAAAAGAAAGAAAGAAGGAGGGAAGGAAGGAAGGGAGGGAGGGAGGGAGGAAAGAAGGAAGGGAAGAAGGAAGGAAGGAGAGAAAGAGAAAGAGAGAGAGAGAAAGAAAGAC | 77033790 |
| 665 | AGAGAAAGAAAGAAAGAAAGAAAAAGAAAAAAAGAAAGAGAAAGAAAGAAGAAAGAAAGAAAGAAAGAAAGAAAAAGAAAAGAAAGT | 77033911 |
| 666 | AAAAAAAAAGAAAAAAAAAAGAAAAGAAAAGAAAAAGAAAAGGAAAGAAAAAAAGAGAAGGAAGGAAGGGAGT | 77116610 |
| 667 | AAAAAAAAAGGGGGGGGGGAAGGAAAGAAGGAAGGAAGGAAGAAGGGGAGGGAGGAAGGGAGAGC | 77208546 |
| 668 | AAAAAAGAGAGAGAAAGAGAGAAAAGGAAAGAAGAAAAGAGAGAAAAAAAGAAAGAAAGAAGGAAAGAAAGAAAGAAAAAGGAAAGAAAGAAAGAAAAAGAAGGAAAGAAAGAAAGAGGGAGGGT | 77248777 |
| 669 | GGGAGGGAGGGAGGGAGGGAAGGAAGGAAGGAAGGAAGGAAGGAAGGAAGGAAGGAAGGAAGGAAGGC | 77248902 |
| 670 | AAAAAAAAGGAAAGAGAGAAGGGAGGAAGGAAGGGAGGGAGGGAGGAAGGAAGGGAGGGAAAAGAAAAGGT | 77355028 |
| 671 | AAAAGAAGGGAGGGAGGGAGGGAGGAAGGAAGGAAGGAAAGAGAAAAGAAAAGGT | 77355125 |
| 672 | AAAAAAAAAAAAAAAAAAAAAAAAAAAAAAAAAAAAAAAAAAAAAAAAGAGGT | 79072468 |
| 673 | GGAAGGAAGGAAGGAAGGAAGGAAGGAAGGAAGGAAGGAAGGAAGGAAGGAAAC | 79209960 |
| 674 | AAAAAAGAAAGAAAGAAAGAAAGAAAGAAAGAAAGAAAGAAAGAAAGAAAGAAAGAAAGAAAGAAAAAGGAAAAGAAT | 79340097 |
| 675 | AAAGAAAGAAAGAAGGAAGGAAGGAAGGAAGGAAGGAAGGAAGGAAGGAAGGAAGGC | 79365633 |
| 676 | GGGAAGGGGGAGAGGGAGAGGGAGAGGGAGAGGGAGAGGAGGGAGAGGGAGAGGGC | 79460563 |
| 677 | AGAAGGAAGGAAGGAAGGGAGAGGGGGGGGAGGGAGGGAGGAAGGAAGGAAGGAAGGGAGGAAGGGAGAGAGGGAGGGAGGGAGGGAGGGAGGAAGGAAGGT | 80747492 |
| 678 | AAAAAAAAAGGAAGAAGAAGGAGGAGGAGGAGGAGGAGGAGAAAGGAAAAGAAAAAGAAAGAAAGAAAAAGAAAAAAT | 80948126 |
| 679 | GAGAGAGAGGGGGAGGGGGGGAAGAGAAGAAGGGGAAAAAGAAGAAGAAGAGGT | 81266317 |
| 680 | AAAAGAAGAAAAAGAAGAAGGAAGGAAGAGGAGGAGGAGGAGAAAGAGGAGGAAGGGGAGGAGGAGGGGAAGAAGAAGAGGAAGAGAAGGAAGGAGGAAGAGC | 81266371 |
| 681 | AGAAGAAAAAAAGAAGAAGGAAGGAAGAGGAGGAGGAGGAGAAAGAGGAGGAAGGGGAGGAGGAGGGGAAGAAGAAGAGGAAGAGGAGGAAGGAGGAAGAGC | 81266497 |
| 682 | AGAAAGAGAGAAAGAAAGAAAGAAAGAAAGGGAGAAAGAGAGAAAGAAAGAAGGAAAGAAAGAAAGGAAAGAAAGAAAGAAAGAGAAGAAAGAAAGAAAGAAAGAAAGAAAGAAAGAAAAGAAAAGAAGAGT | 81867801 |
| 683 | GAGAGGGAGGAAAGAAGGGAGGGAGAGAGGGAGAGAGGGAGGGAAGGAGGGAGAGAGAC | 82161306 |
| 684 | AAAAAAAAAAAAAAAAAGGAAAAGAAAAGAAAAAGAAAGAAAGAAAGAGAGAC | 82358816 |
| 685 | AGAGAGAGAAGAAGAAAGAAAAAGAAAGAAAGAGAGAGAGAAAGAAAGAAAGAAAAGAAAGGAAAAAAC | 82358869 |
| 686 | AAAGGGAGAAAGAAGAAAGGAAGAGAGGGAGGGAAGAAAAAAAGAAAAAGAGAGAAAAAAAGGAAAAGAAAC | 82655054 |
| 687 | GAAAGGAGAGGAGAGAAGAGGGAAGGAAGGAAGGAAGGAAGGAAGGAAGGAAGGAGAGAAAGAAAAAAT | 82655126 |
| 688 | GAAGGAAGGAAGAAGGAAGGAAGGAAGGAAGGAAGGAAAGAAGGGAGGAGGGGAGGGAGGAGGGGAGGGAT | 82840382 |
| 689 | AAAGGAAAGAAGAGAAGGGAAGGAAAGAAGGAAGGAGGGAAAAAAGGAAGAGAGGAAGGAAGAAAAAGAAAT | 83107045 |
| 690 | AGAAAGAGAGAGAGAGAAAGAGAGAGAGAAAAAGAAAGAAAGAAAGAGAGAAAGAAAGAAAGAAAGAAGAAAGAAAGAAAGAAGAAAGAAAGAAAGAAAGAAAGAAAGAAAGAAAGAAAGAAAGAGAAGAAAGAAAGAAAGC | 83303740 |
| 691 | AAAAGAAAGGAAGGAAGGAAGGAAGAAAGAAAGAAAGAAAGAAAGAAAGAAAGAAAGAAAGAAAGAAAGGAAGGAAGGAAGAAAGGAAGGAAGGAAGAAAGGAAGAAAGGAAGAAAGGAAGAAAGAAAGAAAC | 83897787 |
| 692 | AAGAAAGGAGAGAAAAAGAGAGAAAAAAAGAAAGAAAAAAAGAAAAGGAAGGAAGAAAAGAAGGAAAGAAGGAAGGAGGGGGGAAGGAAGGAAGAAAGAAAGGAAGGAAGGAAGGAAGAAAGGAAGAAAGAAAGGAAGGAAGGAAT | 84297154 |
| 693 | AAAAGGAAGAAGGGAGGGAGGGAGGAAGAAGAAAAGAAAGGAGAGAAGAAAGGAAAGGAGAGAAGAGAAGGT | 84383520 |
| 694 | AAGAGAAGAAAAGAGAAGAGAAGAGAAGAGAAGAGAAGAGAAGAGAAGAGAAGAGAAGAGAAGAGAAGAGAAGAGAAAAGAAAGGAAAAGAAAAGAAAAC | 85172141 |
| 695 | GAAAGAAAGAAAGAAAGAAAGAAAGAAAGAAAGAAAGAAAGAAAGAAAGAAAGAAAGAAAGAAAGAAAGAAT | 85548096 |
| 696 | AAAAAGAGAGAAAGAGAAAGAAGGAAAAAGAAAAAAGAAAGAAAAAGAGAAAGAAAGAAAGAAAGAAAGAAAAGAAT | 85639218 |
| 697 | AAAAGAAAGAAAGAGAAAAAAGAGAGGGGAGGGGAGGGGAGAGGAGAGGAGAGGAGAGAT | 85639295 |
| 698 | GAGGAAGAAGAAAAGAAAGAAGAAGAAGAAGGAGGGGAAGGAGGGGAAGGAGAAGAAGGAGAAGAAGT | 86031122 |
| 699 | AGAAGAAGAAGAAGGAGGAGGAGGAAGAGGAGGAGGAGGAAGAAGAGGAAGAAGAAGAAGAAGAAGAGGAAGAAGAAGC | 86031191 |
| 700 | AAGAAGGAAGGAAGAAAGAAAAGAAGGAAGAAGGGAGGGAGGGAGGAAGAGAAAGGAGAGGGAAAGAAGGAAGGAAC | 86126802 |
| 701 | AAAAAGAAAGAAAGAAAGAAAGAAAGAAAGAAAGAAAGAAAGAAAGAAAGAAAGAAAGAGAAAAGAAAAGAAAAGAAAAGAAAAGAAAAGAAAAGAAAAGAAAAGAAAAGAAAAGAAAAGAAAAAAT | 86167426 |
| 702 | AAAAAAGAGAGAGAGAAAGAGGGAAAAAGAAAGAGGGAGAGAGAAGAAGGAAGGAAGGAAGGAAAGAAGAAC | 86267451 |
| 703 | AAAAAAAAAAAAAAAAAGAAAGAAAGAAAGAAAGAAAGAAAGAAAGAAAGGAAAGAAAGAAAGAAAGAGAAAC | 86306420 |
| 704 | AGAAAGAGAGAGAGAGAAAGAAAGAGAGAGAGAGGAAGAAAGGAAGGAAGGAAGGAAGGAAGGAAGGAAGGAAGGAAAT | 86544959 |
| 705 | GGAAAGAAAAGAAAAGAAAGAAGAAAGAGAAGAGAAGAGAGGGAGGGGGAGGGAGGGGGGAT | 86545217 |
| 706 | AGAGAAAGAAAGAAAGAAAGAGAGAGAAGGAGGGAGAGAGAGAGAAGAAAGAAAGAAAGAAAGAGAGAGAGAAAGAAAAGAAAAGAAAGAAAGAAAAAGAAAGAGAGAGAAAGAAAGAAAGAAAGAAAGAAAGAAAGAAAGAAAGAAAGAAAGAAAGAAAGAAAGAAAAAGAAAGAGAAAGAAAGAAAGAAAGAAAGAAAAAGAAAT | 86545279 |
| 707 | GAAAGGAAGGAAGAGAGAGAGAGGGAGAGAGAGAGAAAGAAAGAAAGAAAGAAAAAGAAAGGAAGGAAAGGAC | 86651711 |
| 708 | AAGGAAAGAGGGAGAGAAGAAGAGAAGGGAAGGAAGGAGGGAAGGAGAGAGGGAGGGAGGGAGGGAAGGAGAAAAAGGAGAGAGGGAGGGAGGGAAGGAGAAAAAGGAGAGAGGGAGGGAGGAGAGAGAAAGT | 86714025 |
| 709 | GGAGGGAGGGAAGGAAGAAGGGAGAGAGGGAGGGAAGGAAGGAAGAAGGGAGAGAAGGAGAGAGGGAGGGAGGGAAGGAAGGAAAAGAAAAAT | 86714161 |
| 710 | AAAAAAAAAAAAAAAAAAAAAAAAAGAAGAAGAAGAAGGAGAAGAAAAAGAT | 86874462 |
| 711 | AAAAAAAAAAAAAAAAGAAAAGAAAAAGAAAAAAGAAAAGAAAGAAAAGAAAGAAAAAAAAAAT | 86901346 |
| 712 | AAAAAAAAAAAAAAAAAAAAAAGAAAGAAAGAAAGAAAAAGAAAAAGAAAAT | 87512705 |
| 713 | AGAGGAAGAGAGAGGAAAAGAAAAAGGGAGAAAGAGAAGGAAAGAGAGAGAGAGAGAAGAGGAGGGAAGGAAAGAGAGGGGAGAGAGAGAGC | 87539752 |
| 714 | AAAAAGAAAAAAAAAAAAAAAAAAAAAAGAAAGAAAAAAGAAAAAAAGAAAAAAAAGAAAAT | 87958951 |
| 715 | AAAAAGAAAAAAAAAAAAAAAAAAAAAAGAAAGAAAAAAGAAAAAAAGAAAAAAAAGAAAAT | 88115814 |
| 716 | AAGAAAGGAAGGAAAGAAAGAGGGAGGGAGGGAGGGAAAGAAGGAAGGAAGGAT | 88672297 |
| 717 | GAAGAAGAAAGGAAGGGAGGGAGGGAGGGAGGGAGGGAAGGAAGGAAGGAAGGAAGGAAGGC | 89517656 |
| 718 | AAAAAAAAAAAAAAAAAAGAAAAGAAAAGAAAAAGAAAAGAAAAGAAAAAAGAGAAGAGAAGAAAGGAAAGGAAAGGAAAGGAC | 89950186 |
| 719 | AGGAAAAGAAAAGAAAAAAGAAAAGAAAGGAAAAGAAAAGAAAAGAAAAGAAAAGAAAAGAAAAGAAAAGAAAAGAAC | 89950270 |
| 720 | AAGGAAGGAAGGAAGGAGGGAAGGAGGGAAGGAGGGAGGGAGGGAAGGAAGGAGGGAGGGAAGGAAGGAAGGAAGGAGT | 90027912 |
| 721 | AAAAGGAAGAAAGAAGAAGGAAGGAAGGAAGGAAGGAAGGAAGGAAGGAAGGAAGGAAGGAAGGAAGGAAGGAAGAGAGGGAGAGGGAGGGAGGGGAGGGGAGGGAGGAGGAAGGAAGAGAGGGAGGGAGGGT | 90051632 |
| 722 | GAAAGAAAGAAAGAGAGAAAAAGAAAGAAAGAAAGAAAGAAAGAAAGAAAGAAAGAAAGGAAGAAAGAGAAAGAAAGAAAAAGAAAGAAAGAAAGGAGAAAAGAT | 90097412 |
| 723 | AAGGAAAGGAAGAAGGGAGAAAAGGAGAAAGGGAGGGAGGAAGGGAAGGGAT | 90153647 |
| 724 | AAAGGGAAGAGGAGGGAAAGAGGGAAAGAAAGAGGAGGGGAAAGGAAGGAAGGAAGT | 90253672 |
| 725 | AGGAGAAGGAGGAGAAGGAGAAAGAAGGAGGAGGAGAAGGAGGAGGAGGAAGAGGAGGAC | 90343988 |
| 726 | GAGGAGGGGGAGGAGGAAGAGAAAAAGGGAAGGAGGAGGGGAGGGAGGAGAT | 90344069 |
| 727 | GAAGAAGAAGGAGAAAGAGAAGAGGAGGAGGAGAAGGAAAAGGAGAAGAAGGAAGAAGGAAAAAGAAGAC | 90344190 |
| 728 | AAAAAAAGAGGAAGAAGGAGGAAGAGAAGAAAGAAGAAGGAGGAAGAGAAGAAAGAAGAAGGAGGAAGAGAAGAAAGAAGAAGGAGGAGGAGGAGGAGGAGAAGAGGAAGGAAGGAAGGAAGGAAGGGAGGGAGGGAGGGAGGGAGGGAGGGAAAGAGAAAGAAAAAGAAAGC | 90420584 |
| 729 | AAGAGAGAGAGAGAAAGAAAGAAAAAGAAAGGAAGGAAGGAAGGGAAAGAGAAAGAAAAAGAAAGAAAGAAAGAGAGAGAAAGAAAGAAAGAAAGAAAAAGAAAGAAAGGAAGGAAGAAAGAAAGAAAGAAAGGAAAGAAAGAAAGAAAAAGAGAAGGAAGAGGAGAAGGAGAGAAGGAAGGAAGGAGGGAGGGAGAAAGAAAGAGAGAGAAAAAAT | 90420757 |
| 730 | AAAAAAAAAAAAAAGAAGAAGAAAAAGGAAGGAAGGAAAGAAGGAAGGAAGGAAGGAT | 90531309 |
| 731 | AGAGAGAGAGAGAGAAGGAGAAAGAAAGAAAGAAAGAAAGAAAGAAAGAAAGAAAGAAAGAAAGAAAGAAAGAAAGAAAGAAAGAAAGAAAGAAGAAAAAT | 90544478 |
| 732 | AGGAAAGGAAAGAAGGAAGGAGGGAAGGAAGGAAGGAAGGAAGGAAGGAAGGAAGGAAGGAAGGAAGGAAGGAT | 90548468 |
| 733 | GGAAGGAAGGAGGAGAAGAAGGAAAAAGGGAAAAGAGAGGAGGGAGGGAAAAAAT | 90836553 |
| 734 | GAAAAGGAGGGAGGGAGGAAAGGGAGGAAGGAAAGAGGGAGAGAGAAAGGAAAGAAT | 90944193 |
| 735 | AGAGGGGAGAGGGGAAGGGGAAGGGGAAGAGAGAAGAGGGAGGAAGAGAGAGGGGAGAGAGAT | 91599538 |
| 736 | AGAGAGGAAGGAGGGGAAGGGAGGAGGAGGGAAGAAAGAAAGAAAAAAGAAAAGAGAAT | 91637578 |
| 737 | AGAGAGGAAGGAAGGAAAGAAGGAAGGAAGAGAGAGAAAGAGGAGGGAGGGAGGGAGGAAGGAAGGAAGGAAT | 92105054 |
| 738 | GAAAGAAAGAAAGGAAGGAAGGAAAGAAGGAAGGAAGAAAGAAAGAAAGAAAGAAGGAAAGAAAGAAAGAGC | 92198435 |
| 739 | GAGGAAGGAAGGAGAAAGAGAGGAAGGGGGAGAAAGAGAGAGGGAGAGAAAT | 92258963 |
| 740 | AAAGAAAGAAAGAAGAAAGAAAGAAAGAAAGAAAGAAAGAAAGAAAGAAAGAAAGAAAGAAAGAAAGAAGGAAAGAAAGAAGGAAAGAAAGAAAGAAAGAGAAAGAGAGAGAAAGGAAGGAAGGAAC | 92347288 |
| 741 | GGGAGAAAGGAAGGAAGGAAGGAGAGAAAGAGGAAGGAAGGAAGGAAGGAAGGAAT | 92347415 |
| 742 | GAAAAAAAAAAAAAAAAAAAAAAAAAAAAAAAAAAAAAAAAAAAAAAAGAAAGGC | 92400112 |
| 743 | AGAAGAGAGAGAGGAAGAGAGAGAGAGAGAGAAAGAAAGAGAGAGAGGGAAT | 92498748 |
| 744 | GGAAGGGAGGGAGGGAGGGAGGAAGGAAGGAAAGAAGGAAGGAGAGAAAAGT | 92542135 |
| 745 | AAAAAAAAAAAAAAAAAAAAAAAGAAAGAAAGAAAGAAAGAAAAAGAAAGAAAGAAAGAAAGAAAGAAAGAAAGAAAGAAAGAAAGAAAGAAAGAAAGAAAGAC | 92596130 |
| 746 | AGAAAAAGAAGGAAAGAAAAGAAAGAAAAGGAAAGGAAGAAAGAAAGAAAGAAAGAAAGAAAGAGAGAAAGGAAAAT | 92612274 |
| 747 | AAAAGAAAAAAAGAAAGGAAGGAGAGAAGGGAGGGAGGGAGGGAGGGAGGGAGGAAGGAAGGAAGGAAGGAAGGAAGGAAGGAAGGAAGGAAGGAAGGAAGGAAC | 92612351 |
| 748 | GAGGGAGGAGGAGGAGGAGGAAGGGGAGGAGGGAAAGGAGGAGGAGGAGGAGAAAAGAAGC | 92631864 |
| 749 | GGAGAGGGAGAGGGGGAGAGGGAGGGGGAGGGGAGGGGAGGGGGAGGGGGAGGGGAT | 92795600 |
| 750 | GAGAGGAAGGAAGGGAGGAGGGAAGGAAGGAAGGGAGGGAAAGGAGGGAGGGAGAGAAAAAAGAAGGGAT | 93026703 |
| 751 | GAAAAAAAGAAAGAAAGAAAGAAAGAAAGAGAGAGAGAGAGAGAGAGAGAGAAAGAAAGAAAGAAAGAAAGAAAGAAAGAAAGAAAGAAAGAAAGAAAGAAAGAAAGAAAGAAAGAAAGAAAGAAAGC | 93102673 |
| 752 | AAAGAAAAAAAAAAAAAGAGAGAAAGAAAGGAAGGAAGGAAGGAAGGAGGGAGGC | 93293505 |
| 753 | GAAGGAAGGAAGGGAGGGAGAGAGGGAGGGAGGGAGGGAGGGAGGAAAAGAAT | 93637106 |
| 754 | GGAGGGGAGGGGAGGGGAGGGGAGAAGGAGAGGGAGGGGAGAAGGAGAGGGAGGGGAGAAGGAGAGGGAGGGGAGAAGGAGAGGGAGGGGGAGGAAGAGAGAGAAAAAGAGAAAGAGAGAAAT | 93828756 |
| 755 | AAAAAAAAAAAAAAAAAAAAAAGAGAGAGAGAGAAGAAAGAAAGAGAGGAAGGAAGGAAGGGAGGGAGGGAGGGAC | 94248453 |
| 756 | AGAGAGAGAGAGAGAGAGAGAGAGAGAGAGAGAGAGAGAGAGAGAGAGAGAGAAAGAGAAGT | 94358562 |
| 757 | GAAAGAAAAGAAAAGAAAAAAGAAAAGAAAAGAAAGGAAGGAAGGAAAGAAT | 94382921 |
| 758 | GGGAGGGGAGGGAAGGGGAGAAGAAAGGAAAGGAAAGAGGAAAAAGAAAAGGAAAAAGAAGGGAAAGGAAAGGGAT | 94569694 |
| 759 | GAAAGAAAGAAGGAAAGAAAAAAAGAAAAAAAGAAAAAGAAAGAAGGAAGGAAGGAAGGAAAGAAAAGGAAAAGGAAGGAAGGAAGGAAGGT | 94714934 |
| 760 | AAGAAGAGAAAAGAAAAGAAGAAAAGGGGAAAAAAAAGAAAAGAAAAGGAGGGAGGC | 94859109 |
| 761 | GAAGAAGAGGAAGAAGAGGAAGAAGAGGAGGAAGAGGAAGAAGAGGAAGAAGAAGGAGGGGGGAGGGAT | 95158813 |
| 762 | AAAAGGAAAGAGGGAGGGAGGGAAAGAGAGAGGAAGAGAAGGGAGGAGGGAAAGGAGGAGAGAAAGGAGAAGGGAGGGAAGGAGAAAGT | 95730762 |
| 763 | GAGAGAGAGAGAGAGAGGGAGGGAGGGAGGGAGGAGAGGGAGGGAGGGAGGAGGGGGAGGGAGAGAGGGAGGGAGGGAGGGAAGAGAGGAGAGAGAGAGAGAAAAT | 95754994 |
| 764 | AAAAGAAAAAGGAAAGGAAAGGGAAGGAAGGAAGAAAGAAAAAAAGAAAGAAAAGAAAGAAAGAAAGAAAAAGAAAGAAAGAAAGAAAGAAAGAAAGAAAGAAAGAAAGAAAGAAAGAAAGT | 96385050 |
| 765 | GGAAGAGAAAGAGGAGGAGAAAGGGGGAAGAGAGAGGGAGAAGGAGGAAGAT | 96453213 |
| 766 | AAAAAAAAAAAAAAGAAAGAAAGAAAGAGAAAGAAAGAAAGAAAGAAAGAAAGAAAGAAAGAAAGAAAGAAAGAAAAAT | 96749619 |
| 767 | AAGGGAGGAAGAGAGGAAGAGAGGGAAAGAAGGAAGGGAGGAGGGAAGAAAGGAAGGAAGGAAGGAAGGAAGGAAGGAAGGAAGGAAGGGAGGGAGGGAGGGAGGGAGGGAGGGAGGGAGGGAGGGAGGGAGGGAGGGAGGGAAAAAAT | 96768117 |
| 768 | GGAAAGAGAAGAAAAGAAAAGAAAAAAGAAAAGAAAAGAAAAGGAAAGAAAGGGGT | 96803343 |
| 769 | AAAAAAAAAAAAGAAAAGAAAGAAAGAAAGAAGGAAGGGAAGGGAAGGAAAGGAAAAGAAAAGAAAAAGAAAAAGAAAGAAAGAAAGAAAGAAAGAAAGAAAGAAAGAAAGAAAGAAAGAAAGAAAGAAAGAGAAAGAAAGAAAGAGAAAGGAAGGAAGGAAGGAAGAAAGAAAGAAAGAGAAAGAAAGAAAGGAAAAGAAAGAAAGAAGGAAAGGAAAAGAAAAGAAAGAAAAAAAGAAAGAAAGAAGGAAGGAAAGGAAAAGAAAAAAAAAAGAAAGAAAGAAGGAAGGAAAGGAAAAGAAAAGAAGGC | 96809917 |
| 770 | GAAGAGAGAGGAGAGGAAGAAGAGAGAGAAGGAGGGGAGAGGGAGAAGGGAAT | 97103743 |
| 771 | AGAGAGAGAGAGAGAGAGAGAGAGAGAGGGAGAGAGAAGAAAGAAAGAAAGAAAGAAAGAAAGAAAGAAAGAAAAAAGAAGAAAGAAAGAAGGAAAGAAAGAAAGAAAGAAGAAAGAAAGAAAGAAAGAAAGAAAGAAAGAAAGAAAGAAAGAAAGAAAGAAAGAAAGAAAGAAAGAAAGAAAAAGAAAT | 97890432 |
| 772 | GAAAAAAAGAAGAAGAAAGAAAGAAAGAAAGAAAGAAAGAAAGAAAGAGAAAGAAAGAAAGAGAGAAGGAAGGAAGGAAGGAAAGGAGAGAC | 98140061 |
| 773 | AGAGAAAGAAAGAAAGAAAGAAAGAAAGAAAGAAAGAAAGAAAGAAAGAAAGAAAGAT | 98140153 |
| 774 | GAAAGAAGGGAAAGGGGAAGGGGAAAAAGAAGAAGAGGAAAGAGAAGGAGGAGGAAGAGGAAGAGGAGAAAGGAGGAAGAGGAAGAGGAGAGAGGAGGAGAAGGAC | 98255620 |
| 775 | AAAAAAAAAAAAAAGAAAAGAAAAAAGAAAGAAAGAAAAGAAAAAGAAAGAAAGAGAAAGAAAAAGAAAGAAAGAAAGAAAGAAAGAAAGAAAGAAAGAAAGAAAGAAAGAAAGAGAGAAAGAAAGAAAGGAAAGAAAGAAAGGAAAGAAAGAAAGAAAGAAAGAAAGAAAGAAAGAAAGAAAGAAAGAAAGAAAGAAAGAAAGAAAGAAAGAAAGAAAGAGAAAGAAAGAAAGAAAAT | 98463086 |
| 776 | GGGGAGAGGGAGAGGGGGGAGAGGGAGAGGGAAAGGGAGAGGGAGAGGGAGAGGGAGAGC | 98530541 |
| 777 | AAAGAAAAGAAGGAAGAAAGGAAGAAGAAAGAAAAGAAGGAAGGAAGGAAGGAC | 98638220 |
| 778 | AAAAAAAAAAAAAAAAAAAAAAAAAAGAAAGAAAGAAAAAAGAAAGAAAGAAAAGC | 99287266 |
| 779 | AGAGAAGAAGAGAGAGAGAGGGAGAGAGGGAGGGAGGGAAGGAAAGAGAGAGAGAGAGAGAGAGAGAGAGAGAGAGAAAGT | 99784844 |
| 780 | AAAAAAAAAAAAAAAAAAGAAAGAAAGAAAGAAAGAGAGAGAGAGAGAGAGAGAGAGAGAGAGAGGGAGGGAGGGAGGGAGGAAGGGAGGGAAGGAAGAGT | 99838090 |
| 781 | AAAAAAAAAAAAAAAAAAGAAAGAGAGAGGAAAGAAAGAAAGAAAGAAAGAAAGGAGAGAAAGAAAGAAAGAC | 99874838 |
| 782 | AAAGAGAGAAAGGAAAGGAAAAGGGAAAGGGAAAAGGAAAAGGAAAGGAGGAAGGAAGGAAGGAGAGAGAGAGAAAGAGGGAGAAAGAAAGAAAGAAAAGAGAAGAAAGAAAAGAGAAGAGAAGAGAAAAGAGAAGAGAAGAGAAAAGAAAAGAAGT | 99874911 |
| 783 | AAAAAAAAAAAGAAAAAGAAAAAGAAAGAAAGAAAAAAGGAAAAAAGGAAAAAAAAGAAAGAAAAGAAAAC | 100006236 |
| 784 | AGAAAGAAAGAAAGAAAGAAAGAAAGAAAGAAAGAAAGAAAGAAAGAAAGAAAGAAAGAAAGAAAGAAAGAAAAAAAGAAAGAAAGAAAGAAAAGAGAAGAGAAGAAAGGAAGGGAGAAAGGGAGGC | 100093968 |
| 785 | AGGGAAAGGGGAAGGAAGGAAAGAAAGAAGAAAAAAGAAAGGGGAAAAAAAGAT | 100262832 |
| 786 | AAGGAAGGGAGAGAGAGAAGGAAGGAAGGAAGGAAGAAAAGAAAAAGAAGGAAGAAAAGAAAAGGAAGGAAGGAAGGAGGGAAAGAAAGAAAGAAAAAAAGAAAGAAAGAAAGGAAAAGAAAAAAAGAAAAGAAAGGAAGGAAGGAAAGAAGAAGGGAAGAAAGGAAGAAAAGGAAGGAAGGAAAGAAGGAAGGGGAAGAAAGGAC | 100265598 |
| 787 | AAGGAGGAGGAGAAGGAAGAAGAAGAAGGAGGAGGAGGAGGAGGAGGAAAGAAGAAGAAGAAGGC | 100526772 |
| 788 | GAGAGAGAGAGAGAGAGAGAGAGAGAGAGAGAGAGAGAGAGAGGGAGAGGGAGAGAGGGAAGGAGT | 100916383 |
| 789 | AGAAAGAAGGAAAAGAAGGAAGGAAGAAAGGAAAGAAGAAAGGAAGGAAGGGAAGGAGGGAC | 100968985 |
| 790 | GGGGAGAGGGAGAGGGAGAAGGAGAGGGAGAGGGAGAGGGAGAGGGAGAGGGAGAGGGAGAAT | 101139375 |
| 791 | AGAAGGAGAAGGAGAAGAAGAAAAGAAGAAGAAGAAAGAGAAGGAGAAGAGAGGAT | 101144362 |
| 792 | AAGAAAGGAAGGAAGGAAGGAAAGAAGGAAGGAAGGAAAGAAGGAAGGAAGGAAGGGAGGAAGGAAGGAAGGAAGGAAGGAAAGAAGGAAGGAAGGAAGGAAGGAAGGAAAGGAAGGAAGGAGAAAAGAAAAGAAAAGAGAAGAAAAGAAGAGAAAAGGAAAAAAAAAGT | 101147865 |
| 793 | AAAAAAAGAAAAAGAAGAAAGAGAAAGAAAGAAGGAAGGAAGGAAGGAAGGAAGGAAGGAAGGAAGGAAGGAAGGAAGGAAAGAAAC | 101251626 |
| 794 | GAAAGGAAGGAAGGAAGGAAGGAAGGAAGGAAGGAAGGAAGGGAGGAAAAGAAGAAAT | 101379604 |
| 795 | AAAAAAAAGAAAAAAAAAGAAAGAAAAGAAAAGAAAAGAAAAGAAAAAAAGAGC | 101642460 |
| 796 | AAAAAAAAAAAAAAAAAGAAAAAGAAAGAAAAGAAAGAAAAAGGAAGGAAGGAAGGAAGAAAGGAAGGAAGGAAGGGAGAAAAAAT | 101683159 |
| 797 | AAAAGGAAGAGGAGAAGAGGGAGAAGAGAAGGGAGGGGAGGGAAGAGGAGAGGAAGGAAAGAAAGGAGAGGGGAAGAAAAGAAAGAGGAGGGGAGGGGAAGGAAGGC | 101696336 |
| 798 | AGAAAAGAGAAAAGGAAAGGAAAGAAAAAAGAAAGAAAAGAAAGGAAAGGAAAGGAAAGGAAAGGAAAGGAAAGGAAAAGAAAAGAAAAGAAAAGAAAAGAAAAGAAAAGAAAAGAAAAGAAAGAAAAC | 101712555 |
| 799 | AAAAAGAAAGAAGGAAAGAAAGAAAGAAAGAAAGAAAGAAAGAAAGAAAGAGAGAGAGAGAGAGAGAGAGAGAGAGAGAGAGAGAAAGAAAGAAAGAAAGAAAGAAAGAAAGAAAGAAAGAAAGAAAGAAAGAAAGAAAGAAAGAAAGAAAGAAAGAAAGAAAC | 101742941 |
| 800 | GAAAGAAAGAAAGAAGGAAAGAAAGAAAGAAAGAAAGAAGGAAAGAAAGAAAGAAAGAGAAAAGAAAAGAAAAGAAAAGAAAAGAAAAGAAAAGAAAAGAAAAGAAAAGAAAAAC | 101879970 |
| 801 | GAAAGAAAGAGAAAGAGAGAAAGAGAAAGAAAGAAAGAAAGAAAGAAAGAAAGAAAGAAAGAAAGAAAGAAAGAAAGAAAGAAAGAAAGAAAGAAAGAAAGAAAGGAGGGAGGGAGGGAAGGAAGGAAGGAAGAGAGAAAGC | 101886817 |
| 802 | AGAAAGAAAGAAAAAGGAAAGAAAAGAAGGAAAGAAAGAGAAAGAAAGAAAGAAAGAAAGAAAGAAGGAAAGAAAGAGAAAGAAAGAGGGAGGGAGGAAGGAAGGAAGGGAGGGAAAGAAAGAAAGC | 102806404 |
| 803 | AGAGAGAGAAAGAGAGAGAGAGAGAGAGAGAGAGAGAGAGAGAGAGAGAGAGAGAGAGAGAGAGAT | 102925098 |
| 804 | GGAAGGAGGGGAGGGAGGGAGAAAGAAAGAGAAAGAAAGGAAGGAAGGAAGGAGGGAGGGAGGAAGGGAAAGAGAAAAAGAAAGAAAAAAAGAAAGAAAGAAAGGAGGGAGGGAGGGAAAAGAAAAGAAAAGAAGAGAAAGAAGAAAGAAAGAAAGAGGAT | 103242975 |
| 805 | GGGAAGGGGAAAGAAGGGAGGGAAGGGAGAGGGAAGGAAAGAAAAGAAGAAAGAAGAAAT | 103243136 |
| 806 | AAAAGGAAGGAAGGAAGGAGGAAGGAAGGAAGGAAAAAAGGAAGGAAGGAAAAAAGGAAAAAT | 104591642 |
| 807 | AGAGAAGAAGAAGAAGAAGGAAGAAGAAGAAGAAGAAGAAGAAGAAGAAGAAGAAGAAGAAGAAGAAGAAGGAGAAGGAGAAGGAGAAGGAGAAGGGGGGT | 104627982 |
| 808 | AAGAAAAGGGAAGGAAGGAAGGAAGGGAAGGAGGGAGGGAGGGAGGAAGGGGAGGGGAGGGGAGGGGAGGGAGGAAGGAAGGAAGGAGAT | 104813790 |
| 809 | GGGGGGGAGAGAGAGAGAGAGAGAGAGAGAGAGAGAGAGAGAGAGAGAGAGAGAGAC | 104938327 |
| 810 | AAAAAAAAAGGAAAGAGAGAGAAAGGAAGGAAGGAAGAAAGGAAGAAAAGAAGGAGGGAGGGAGGAAGGAAAAT | 105122189 |
| 811 | GAAAGAAAGAAAGAAAGAAAGAAAGAAAGAAAGAAAGAAAGAAAGAAAGAAAGAAAAAGAAAGAGAGAGAAAGAAAGAAC | 106454174 |
| 812 | AGAGAGAGAGAGAGAGAGAGAGAGAGAGAGAGAGAGAGAGAGAGAGAGAGAGAGAGAGAGAGAGAGAGAGAGAGAT | 106801846 |
| 813 | AGAGAGAGAGAGAGAGAGAAAGAGAGAGAGGGAGAGAGAGAGAGAAAGAGAAAT | 106898898 |
| 814 | AAAAAAAAAAAAAAAAAAAAAAAAAAAAAAAAAAAAAAAAAGAAAAAGAAGAGT | 106909046 |
| 815 | GGAAGGAAGGAAGGAAGGAAGGAAGGAAGGAAGGAAGGAAGGAAGGAAGGAAAAGAAAAGAAAAGT | 107167178 |
| 816 | AAAAAAAAAAAAAAAAAAAAAAAAAAGAAAAAAAAAAAAAAGAAAAGAAAGAAAGAAAAGAAAAT | 107271819 |
| 817 | AAAAAAAGAAAGAGAAAAGAAAAGGAAAAAAGGAAGAAAGGAAAGAAGGAAGGAAGC | 107423263 |
| 818 | AGGAAGAAAGGGAGGAAGGAAAGAGGAAAGGAAGGAAGGAAGGGAAAGAAAAC | 107465750 |
| 819 | AAAAAAAAAAAAAAAAAAAAAAAAAAAAAAAAAAAAAAAGAAGAAGAAAGAAAAAT | 107619022 |
| 820 | AAGAAGAGGAAGAAGAAGAGGAGGAAGAAGGAGGGGAGGAAAAAGAAAAGGAAGAAAT | 107999795 |
| 821 | GAGAGAGAGAGAGAGAGGAGAGAGAGAGAAGAGAGAGAAAGAGGAAGAAGAAAAAGGAGGAGGAGGGGGAAAGGAAGGAAAGAAGGAAGGAAGGAAGGAAGGAAGGAAGGGAGGGAGGGAGGGAGGGAGGGAGGGAAGGAGGGAGGAAGGGAGGGAGGGAGGAAGT | 108030208 |
| 822 | GAAAGAAAGAAAAAGAAAGAAAGGAAAGAAAGAAGAAAGGAAGGAAAAAGGT | 108128685 |
| 823 | AGGAAAGAGAAAGAGAGAAAGAGAAAGGAAGGAAGGAAGGGAGGGAGGGAGGGAAAAT | 108334029 |
| 824 | AAAAAAGAAAGAAAGAAAGAAAGAAAGAAAGAAAGAAAGAAAGAAAGAAAGAAAGAAAGAAAGAAAGAGAGAAAGAAAAGAAAAGAAAAGAAAAAGAAGGAAGGAAGT | 108334087 |
| 825 | GGAAAAAAGAAAGAAAGAAAGAAAGAGAGAGAGAGAGAGAGAGAGAAAGAAC | 108393046 |
| 826 | AGAAAAAAAGAAAGAAAGAAAGAAAGAAAGAAAGAAAGAAAGAAAGAAAGAAAGAAAGAAAGAAAGAAAGAAAGAAAGAAAGAAAAT | 108403195 |
| 827 | AGGAAGAAGAAGAAGAAGAAGAAGAAGAAGAAGAAGAAGAAGAAGAAGAAGAAGAAGAAGAAGGAGGAGGAGGAGGAGGAGGAGGAGGAGGAGGAGGAGGAGGAGGAGAAGAAGAAAGAGGAAGAAGGAGAAGAGGAGAAGGAGGAGGAGGAGT | 109024827 |
| 828 | AAGAAAGAAAGAAAGAGAAAGAGAGAGAAAGAGAAGAGAAAAAGAGAGAAAGAAAGAAAGGAAGGAAGGAAGAAAGAAAGAGAGAGAAAGAGAAGAGAAAAAGAC | 109075231 |
| 829 | AGAGAAAGAAAGAAAGAGAGAGAAAGAGAAGAGAGAAAGAGAGAAAGAAAGGAAGGAAGGAAGAAAGAAAGAGAGAAAGAGAAGAGAGAAAGAGAGAGAAAGGAAGGAAGGAAGGAAAAGAAAGAAAGAAAGAAAGAAAGAAAGAAAGAAAGAAAGAAAGAAAGAGAAAGAAAGGGAAAGAAAGAAAAGAT | 109075336 |
| 830 | AGGAAGGAAGAAAAAGAAAGAAAGAAAAAGAAAGAAAGAAAGAAAGAAAGAAAGAAAGAAAGAAAGAAAGGAAGGAAGGAAGGAAGGAAGGAAGGAAGGAAGGAAGGAAGGAAGGAAGAAAGAAAAAGAAAGAAAGAAGGAAGGAAGGAAAGAAAGAAGGAAAGAAAGAAAGAAAGAGAGGGC | 109154700 |
| 831 | AAAAGAAAAAAAGAAAGAAAGAAGAAGGAAGGAAGGAAGGAAGGAAGGAAGGAAGGAAGGAAGGAAGGAAGGAAGGAAGGAGGGAGGGAGGGAGGGAGGGAGGGAGGGAGGGAGGGAGGGAGAGAAAT | 109155022 |
| 832 | AGGAGAAGGAGGAGAGAAAGAGAAGAGGAAGAGAAAGAGGAAGAGGAAGAAGAAGAAC | 109216731 |
| 833 | AGGGAGGGAGGGAGGAAGGAAGGAAGGAAGGAAGGAAGGAAGGAAGGAAGGAAGGAAGGAAAAAT | 109221185 |
| 834 | GAGAAGAGAAAAGAAGAGGAGGGAAGAGGAGGGGAGGGGAGGGGAGGGGAGGGGAGGGAAGGGAAGAAGAGC | 109332244 |
| 835 | GAGGAGGGGGGAGGAGGGGGAGGAAAGGAGAAAGGAGGAAGGAGGAAGGAAGAAAC | 109363336 |
| 836 | GGAAAGGAAGGAAGGAAAGAAGGAAGGAAGGAAGGAAAGAAGGGAGGAAGGGAGGGAAC | 109375394 |
| 837 | AAAAAAAAAAAAAAAAAAAAAAAAAAAAAAAAGGAAGAAAAGAAAAGGAAAAAAAAAT | 109795800 |
| 838 | AGAGAGAGAGAGAGGAGAGAGAGAGAGAGAAAGAGAGGAAAGAAAGAAAAAGAAAGAAAGAAAGAAAAAGAAAGAAAGGGAGAGAAGGAAAAGAAAAGAAAGAAAGAAAGAGAGAAAC | 110675425 |
| 839 | GGGAAGGAAGGAAAGGAAGGAAGGAAGGAAGGGAGGGAGGGAGGGAGGGAGAGAGGGAGGGAGGGGAGGGGAGGAGAGGGAGGGC | 110731620 |
| 840 | AGGGGAGGGGAGGGGAGGGGAGGGAGAGAAAGAGAAAGGGAGAAAAAAGAAAGAAAGAAAGAGAAAAAGGAAGGAAGGAAGGAAGAGAC | 110731705 |
| 841 | AGAGAGAGAGAGAGGAGAGAGAGAGAGAGAAAGAGAGGAAAGAAAGAAAAAGAAAGAAAGAAAGAAAAAGAAAGAAAGGGAGAGAAGGAAAAGAAAAGAAAGAAAGAAAGAGAGAAAC | 110749886 |
| 842 | GGAGAGGGAGAGGGAGAGGGAGAGGGAGAGGGAGAGGGAGAGGGAGAGGGAGAGGT | 110784700 |
| 843 | GGAAAGAGAGGGAGAGGAGGGAGAGGAGGGAGAGGAGGGAGAGGAGGGAGAGGAGGGAGAC | 110830691 |
| 844 | GGGAGAGGGAGAGGGAGAGGGAGAGGGAGAGGGAGAGGAGGGAGAGGAGGGAGAGGAGGGAGAGAGC | 110830752 |
| 845 | AGAAGAAAAAGAAAGAAGGAAGGAAGGAAGGAAAGAAAGAAAAGAAAGAGAGAGAGAC | 110945535 |
| 846 | AGAAAGAAAGAGAAAGAGAGAGAAGAGAAGAGAGGGGAAGGGAGGGGAGGGGAGGGGGAAAAAGAAC | 110945597 |
| 847 | AAGGGAAAGAAGGGAGGGAGGGAAGAAGGAAGAAAGGAGGGGGAAGAAAGAAGGAAGGGAAGAAGGAAGAAAGGGAGGGAGGGGAGAGGAAGT | 110962862 |
| 848 | AAAAAAAAAAAAAAAAAGAAAGAAGGAAGGAAAGGAAAGGGGAAAGGGGAGGAAGGGAGGGAGGAAAGAAGAT | 111291644 |
| 849 | GAGGGAGGGAGGGAGGAGGGAAGGAAGGGAAAGAAAAGGAAAGAAGGGAGGGAGGGAC | 111342318 |
| 850 | GGAGGGGGGAAGGAAGGGAAAGAAAAGGAAGGAAGGAAGGAGAGAAGGAAGGAAGGAAGGAAAGAAGGAAGGGAAAGAAAAGAAAGGAAGGAAGGAAGGAAAAC | 111342376 |
| 851 | AGAGAGAGAGAGAGAGAGAGAGAAAGAGAGAGAGAGAGAGAGAGAGAGAGAGAGAGAGAGAGAGAGAGAGAGAGAGGC | 111407277 |
| 852 | AAAAAAAAAAAAAAAAAAGAAAGAAAGAAAGAAAGAAAGAAAGAAAGAAAGAAAGAAAGAAAGAAAGAAAAAAGAGAAAGAT | 111805125 |
| 853 | AGAAAGAAAGAAAGAAAGAAAAGAAAGAAAGAAAGAAAGAAAGAAAGAAAGAAAGAAAGAAAGAAAAGAAAGAAAGAAAGAAAGAAAGAAAGAAAGAAAGAAAGAAAGAAAGAAAGGAAAGAAAGAAAAGAAAGAAAGAAAGGAAAGAAAGAAAGAAAGAAGGAAGGAAGGAAGGAAGGAAGGAAGGAAGGAAGGGAAGGAAGGAAGGAAGGAAGGAAGGAAGGAAGGAAGGAAGGAT | 111947959 |
| 854 | AGAAAGAAAGAAAGAAAGAAAAGAAAGAAAGAAAGAAAGAAAGAAAGAAAGAAAGAAAGAAAGAAAAGAAAGAAAGAAAGAAAGAAAGAAAGAAAGAAAGAAAGAAAGAAAGAAAGGAAAGAAAGAAAAGAAAGAAAGAAAGGAAAGAAAGAAAGAAAGAAGGAAGGAAGGAAGGAAGGAAGGAAGGAAGGAAGGGAAGGAAGGAAGGAAGGAAGGAAGGAAGGAAGGAAGGAAGGAT | 112104822 |
| 855 | GAAAGAAAGGAAGGGAAGGGAAGGGAAGGGAAGGGAGGGGAAGGGAAGGGAAGGGAAGGGAAGGGGAAAGAAAGGAAGAAAGAAAGAAAGAAAGAC | 112175803 |
| 856 | AAAAAAAAAAAAAAAAAAAAAAAAGAAAAAAAAGAAGAAGAAGAAGAAGAAAGGAGAAT | 112249564 |
| 857 | AGGAAGGGAAGGGAAGGGAAGGGGAAAGGGAGAAAGAAAGAAAGAAAGAAAGAGAGAAAGAAAGAAAGAAAGAGAAAGAAGGGAAGGAAGGAAGGGGAAAGGAAGGAAGGAAAT | 112883291 |
| 858 | AAAAAAGAAAGAAAGAAAGAAAGAAAGAAAGAAAGAAAGAAAGAAAGAAAGAAAGAAAGAAAGAAAGAGAAAGAAAGAAAGAAAT | 112955792 |
| 859 | GAGGAAGGAAGGAAGGAAGGAAGGAAGGAAGGAAGGAAGGAAGGAAGGAAGGAAAGGGAC | 113061765 |
| 860 | AGAGGAAGAGAGAGAGAGAAGAGAGAGAGAGGAGAGAGAGAAGAGAGAGAGGT | 113224520 |
| 861 | GAGAGAGAAGAGAGGAGAGAGAAGAGAGAGAGAGGAGAGAGAGAAGAGAGAGAGGT | 113224615 |
| 862 | GAGAGAGAAGAGAGGAGAGAGGAGAGAGAGAGAGGAAAGGGAGAAAGAGAGGAGAGAGGAGAGAT | 113224671 |
| 863 | AGGAGAGAGAGGAGAGAGGAGAGAGAGAGAGAGAAAGAGAGAGAGAGAGAGAGAC | 113224763 |
| 864 | AGGAGGAGGAAGAAGAGGAGGAAAAAGGAGAGGAGGAGGGGGAGAGGGAGGGAGAAGAGAGGGAGGAGAAAGAGGAGGAGGAAGAGGAGGGAAGAAGC | 113994399 |
| 865 | GGAAGGAAGGAAGGGGAAGGGGAAAGGGAAGAAGGAAGGAAGAAAGAAAGAAGGAAAGGAGGGAGGGAAGGAAGGAAGGAAGGAAGGGAGGGAGGAAGGAAGC | 114045250 |
| 866 | GAGGGAGGGAGGAAGGAGGAAGGAAGGAAGGAAGGAAGGAGGAAGGAAGGAAGGAAGGAAGGAAGAAGGAAGGAAGGAAGGGAGGGAAT | 114544540 |
| 867 | AAGAAAGAAAGAAAGAAAGAAAGAAAGAAAGAAAGAAAGAAAGAAAGAAAAGAAAC | 114621563 |
| 868 | AGAGAGAGAGGGAGGGAGGGAGGAAGGAAGGAAGGAAGGAAGGAAGGAAGGAAGGAAGGAAGGAAGGAAGGAAGGAAAGAAAAC | 114621621 |
| 869 | AAGAAAGAAAGAAAGAAAAGGAAGGGAAGGGAAGGGAGAAAGAAAGAAAGAAAGAAAGAAAGAAAGAAAGAAAGAAAGAAAGAAAGAAGAAAGAAAGAAAGAAAGAAGAAAGAAGGAAAAT | 114657170 |
| 870 | AAAAAAAAAAAAAAAAAAGAAAAGAAAAAGAAAAGAAAGGAAAAAAGAAAAAAAC | 114833925 |
| 871 | AGAGAGAGAGGAAGGAAGGAAGGAAGGAAGGAAGGAAGGAAGGAAGGGAGGGAAGGAAGGAGAAGAAGGAAAGAAAGAAGAAGGAAAGAAGAAAAAGAAAGAGAGAGAGAAAGGAAGGAAGGGAGAGAAAGAAAGAGAAAGAAAGGAAGGAAGGGAGGGAGGGAGGAAGGAAGGAAGGAAGGAAGGAAGGAAGGAAGGAAGGAAGGAAGGAAGGAAAAAAGAAAAGAAAC | 114838583 |
| 872 | AAAAAAAAAAAGGAGAGAAAGAGAAAGAGAGAGGGAGGGAGGGGAGGGAAGGGGAGAGAGAGAGAGGGAAGGAGGGAGAAAGGGAGAGGGAGAGGAAAGAAAAAGGAAAGGAAGAAAAAGAAAGAAAGAAAC | 114923703 |
| 873 | GGAAGGAGGAAGGAAGGAAGGGAAAAAAGAAAGAAAAAAGAGAAAGAAAGAAGAAGGAAGGAAGGAAAAGAAAGAGAGGAAGGGAGGGC | 114923837 |
| 874 | AGGAGAGAGGGAGGGAAGGAGGGAGGGAGAGGAAAGGAAGGAAGGAAGGAAGGAAAAGAGGGAGGGAGGGAGAGAGGGAAGGC | 114923935 |
| 875 | AAGAAAGAAAGAAGGAAAGAAAGAAGGAAAGAGAGAGAGAAAGAGAAAGAAAGAAAGAAT | 115210067 |
| 876 | AAGAAAGAAAGAGAGAGAGAGAAAGAAAGAAAAGAAAAGAGAAAGGAAGAGAGGAAGC | 115210128 |
| 877 | AAAAAAAAAAAAAAAAAAGAGAAAGAGAGGAAGGAAGGAAGGAAGGGAAAGAAAGAAGAGAAGAGAAGAGAAGAGAAGAGAAGAGAAGAGAAGAGAAGAGAAGAGAAGAGGGGAGGGGAGGGGGC | 115210527 |
| 878 | AAAAAAAAAAAGAAAGAAAGAAAGAAAGAAAGAAAGAAAAAAGAAAAGAAAAAAAAAGAAAC | 115236518 |
| 879 | AAAAAAAAAAAAAAAAAAAAGAAAAGAAAGAGAGAAAGAAAAAGGAAAAAAAGAAAGAAAAGAAAAGC | 115326460 |
| 880 | GAAGAAAGAGAGAGAGGGAGAGAGGGAGGAAGAGAGGAAGGAAGAGAGGAAGT | 115673908 |
| 881 | GAGGGAGGAAGGAGGGAAAAGAAAGGGAAGAGAAAGGAAGGGAGAAAGAAGGGAGAGAAT | 115673961 |
| 882 | GAAAAGAGGGAAAGAGGGAAGAGAGGGAGGGAGGGAAGAAGGGAAAAGGAAGAGAAAGGGAAAAAAAGAAAGAAGC | 116058955 |
| 883 | GGAAGGAAGGGAGGGAGGGAGGGAGGGAGGGAGGGAGGGAGGGAGGGAGGAAAGAAAAGAAAAAC | 116141834 |
| 884 | AAAGAAAGGAGAAAGAAAGGAAGGGAAGGGAGGGGAAGGGGAAGGGAGGAAGGAAGGAAAC | 116243544 |
| 885 | GAAGGGAGAAGGGGAAGGGAAGGGAAGGGAAGGGAAGGGAAGGGAAGGGAAGGGAAGGGAC | 116277134 |
| 886 | AAAAGAAGGAAGGAAGGAAGGAAGGAAGGAAGGAAGGAAGGAAGGAAGGAAGGAAGGAAGGAAGGAAGGAAGGAAGGAAAGAAAGGAAGAAAAAGAAAAAT | 116300944 |
| 887 | AGGAAGGAAGGAGGGAAGGAAGGAAGGAAGGAAGGAAGGAAGGAAGGAAGGAAGGAAGGAAGGAAGGGGAT | 116648302 |
| 888 | AAAAAAAAAAAAAAGAAAGAAAGAAAGAAAGAAAGAAAGAAAGAAAGAAAGAAAGAAAGGAAT | 116763954 |
| 889 | AAAAAAAAAAAAAAAGAGAGAAAGAAAGAAAGAAAGAAAAGAAGAGAAAAAT | 116807079 |
| 890 | AAAAAAAAAAAAAAAAAAAAGAAAAGAAAAGAAAAGGAAAGGAAAAGAAAAAGT | 117002985 |
| 891 | AAAAAAAAAAAAAAAAAGAGAAAAAAGAAAAAAAAAGAAAGAAAAGAAAGAAT | 117089509 |
| 892 | AAGAAAAGAAAAGGAAAAGGAAGAAGGAAGAGGGAAAAGGAAAGGGAGGAAGGAAAAAAAT | 117113699 |
| 893 | AGGAAAAAAGAAAGAAAAGAAAGGGAAGAAAGAAAAGAAAAGAGAAAAGAAAAAGAGAAAGAAAGT | 117161245 |
| 894 | AAAAAAAAAAAAAAAAAAAAAGAGAAAAAAGAAAAAAAAAAAGAAAGAGAAAAAGAT | 117307883 |
| 895 | AGAAAGAGGAAGGGAGAAGGGAAAAGAAGAGGAGGAGGAAGAAGAAGAGAAGGAGAGGAAGAAGAGAAGGAGGAGGAAT | 117779831 |
| 896 | GGAAGAAGAAGAAGAAGAGGAGGAGGAGGAGGGAGGAGGAGGGAGGAGAAGAAGAAAGAGGAGGAGAAT | 117863843 |
| 897 | GAAAGGAAAGAAGGAAGGAAGGAAGGGAGGGAGGGAGAGAGGGAGGGAGGAAGGAAGGAAGGAAGGAAGGAAGGAAGGAAGGAAGGAAGGAAGGAAGGGGGC | 117913895 |
| 898 | AAAAAAAAAAGAAGAAGAAGAAGAAGAAGAAGAAGAAGAAGAAGAAAGAAGAAGAAGAAGAAGAAGAAGGAGAAGAAGAAGAAGAAGAAGAAGGAGAAGAAGGAGAAGAAGAAGAAGAAGAAGAAGAAGAAGAAGAGT | 118212141 |
| 899 | AAAAAAGAGAGAAAGAGAAAGAGAAAAGGAAGGAAGGAAGGAGAAAGAAAGAAAGAAAAGGAAAGAAAGAAAAGAAAGAAAAAGGAAGGAAGGAAAT | 118315340 |
| 900 | AAAAAAAAAAAGAAAGAAAGAAAAAGAGAAAGAAAGAAAGAAGGAGGAAAGGAAAGGAAT | 118642145 |
| 901 | AAAGAAAAAAAGAGAAAGAAAGAAGAAAGAAAGAAAGAGAAGGAAGAGAAAGAAAGC | 118642275 |
| 902 | GAGGAGGGAAGGAGGAGGGGAGGGGAGGAGGGGAAAAAAGAGAGGAGGGGAGGT | 118672770 |
| 903 | AAAGAGAAAGAAAGAAAGAAAGAAGGAAGGAAGGAAAGAAGGAAGAAAGGAAGGGGAAGGGAAGGAGGGAGGAAGGAGGGAAGGAAGGAAGGAAGGGGAAGGGAAGGAAGGAAGGAAGGGAGGAAAGAAGAAAGAAAGAGAAAGAAAGAGAGAAAGAAGGAAGAGAT | 118799598 |
| 904 | GAAAGAAAGAAAGGAAGGAAGGAAGGAAGGAAGGAAGGAAGGAAGGAAGGAAGGAAGGAAGGAAGGAAGGAAGGAAGGAAGGAAAGAC | 118807004 |
| 905 | AGGAAGGGAGGGGGAGGGGAGGGAAAGGGAGGGGAGGGGAGGGAGGGAGGGGAGGGGAGAGAGGAAGGAT | 119017958 |
| 906 | AAAAAAAAAAAAAAAAAAAAGAAAGAAAGAAAGAAAGGAAGGAAGAAGAAAAT | 119090785 |
| 907 | AAAAAAAAAAAAAAAAAAAAAAAAGAAGAAGAAAGAAAAGAAAAGAAAGAAAGAAAAAGAAAGAAGGAAAGAAAGAAAAT | 119092243 |
| 908 | AGAGAGAAAGAAAGGAAGGAAGGAAGGGAGGGAGGGAGAGAAGGAAGGAAGGAAGGAAGGAAGGAAGGAAGGAAGGAAGGAAGGAAGGAAGGAAGGAGAAAGAAAGAAAAGAAAGAAAGAGAGAGAGAAAGAAAGAAAGAAAGAGAGAGAGAGAGAGAGAAAGAGAGAGT | 119121897 |
| 909 | AGAAGGGAAAGAAAGGAAAAAAGAAAGAAGGAAGAGAGAAAGGAGAGAAGAGAGAAC | 119129626 |
| 910 | GAAAAGGAAGGAGAAGAGGAGGAGGAGGAAGGAGGAAGAGGAGGAGGAGAAGGAGGAGGAGGAGGAGGGGGAGGAGGAGAAGAAGAAAGGAAAGAAAGAAGGAAAGAGAAGGAAT | 119320436 |
| 911 | AGAAAGAAAAGAAAGAAAGAGAGAGAGAGAGAAAGGAAGGAAGGAAGGAAGGT | 119479784 |
| 912 | AGAAAGAAAGAAAGAAAGAAAGAAAGAAAGAAAGAAAGAAAGAAAGAAAGAAAGAAAGAAAGAAAGAAAGAAAGAAAAGAAAGAAAAGAAAGAAAGAAAGAAAGAAAGAAAGAAAGAAAGAAAGAAAGAAAGAAAGAAAGAAAGAGT | 119479877 |
| 913 | AGAGAAAGAAAGAAAGAGAAAGAAAGAAAAGAAAGAAAAAAAGAGAGAGAGAAAGAGAAAGAGAGAAAGGGAAAGGGGAGAAGGGAAGAT | 119481175 |
| 914 | AAAAAAAAAAAAAAAAGAAAGAAAGAAAGAAAAAAGAAAAAGAAAAAAGAAAAAAAGAAAT | 119584743 |
| 915 | AAAAAAAAAAAAAAAAAAAAAAAAAAAAAAAAAGGAAGAAGAGAAGGAGGAAGAGAC | 119729617 |
| 916 | AAAAAAAAAAAAGAAAGAAAGAAAGAAAGAAAGAAAGAAAGAAAGAAAGAAAGAAAAAC | 119760337 |
| 917 | AGGAGAGGGGGAAGGGAAGAAAAGGAGGGGGAAGGGAGGAGAAGGGAAAGGGGGAAGGAAGGGGAAAGGAAGGAAGAAAAGGAGGGC | 119875821 |
| 918 | AGGAGAGGGGGAAGGGAAGAAAAGGAGGGGGAAGGGAGGAGAAGGGAAAGGGGGAAGGAAGGGGAAAGGAAGGAAGAAAAGGAGGGC | 120032684 |
| 919 | AAAAAAAAAAAAAAAAAAAAAAAAAAGAAAAAGAAAAAGAAAAAAAGAAAAGAAAAAAAAGGT | 120276273 |
| 920 | GAAGAAAAAGAAAGAAAGAAAGAAAGAAAGAAAGAAAGAAAGAAAGAAAGAAAGAAAGAAAGAAAAGAAAGAAAGAAAC | 120356748 |
| 921 | AAAAAAAAAAAAAAAAAGAAAGAAAGAAAGAAAGAAAAGAAAGAGAAAAAAAGAAAGGAAAGAAGGAAGGAAGAAAGAAAGAAAGAAAGAAAGAAAGAAAGAAAGAAAGAAAGAAAGAAAGAAAGAAAGAAAC | 120411745 |
| 922 | AGAAAAAGAAAGAAAGAAAGAAAGAAGGAAAGAAGGAAAGAAGGAAGGAAGGAAGT | 120643799 |
| 923 | AAAGAAAGGAAGAAAGAAAGAAAGAAAGAAAGAAAGAAAGAAAGAAAGAAAGGAAGGAAGGAAGGAAGGAAGGAAGAAAGAAAGAAAGAAAAAGAAAGAAAAT | 121459604 |
| 924 | GAAAAGAAAGAAAGAAAGGAAGAAAGAAAGGAAGAAGGAAGGAAGGAAGGAAGGAAGGAAGGAAGGAAGGAAGGAAGGAAAGAAAGAAAGAAGAAAGAAGGAAGGAAGGAAGGAAGGAAGGAAAGAAAGAAAGAAAGAAAGAAAGAAAGAAAGAAAGAAAGAAAGAAAGAAAGGAAGAAGGAAAGAAAGAT | 121500848 |
| 925 | GGAAGGAAGGAAGGAAGGAAAGAAGGAAGGAAGGAAAGAAAGGAAGGAAGGAAGGAAGGAAAGAAAGAAAGAAAGAAAGAAAGAAAGAAAGAAAGAAAGAAGGAAAGAAAGAT | 121501039 |
| 926 | GGAAGGAAGGAAGGAAGGAAAGAAGGAAGGAAGGAAAGAAAGAAAGGAAGGAAGGAAGGAAAGAAAGAAAGAAAGAGAGAGAGAAAGAAAAAGAAAGAAAGAAAGGGAGGGAGGGAAGGAAGGAAAAT | 121501152 |
| 927 | AAGAAAGAAAGAAAAGAAAGAAAGAAGAGAGAGAGAAGGAGGAGGAGGAGAAGAGGGAGGGAGGGAGGAAGGAAGGAAGGAAAGAAAGAAT | 121663294 |
| 928 | GAAAGAAGAAAGAAAGAAAGAAAAAGAGAAAGAAAGAAAGAAAAGAAAGAAAGAAAGAAAAT | 122058659 |
| 929 | GGAGGGAAGGAAAAAAGGAAGGAAGGAAGAAAGAAGGAAAGAAGGAGGAAGAAAGGAAGGGAGGGAAGGAGGGAAGAAGGAAAGAAGGAAAGAAGAAAGGAC | 122087889 |
| 930 | GGAAGGAAGGAAGGAAGGAAGGAAGGAAGGAAGGAAGGAAAGAAGGAAGGAAGGAAGGAAGGAAGGAGGGAAGT | 122088059 |
| 931 | AAAAAAAAAAGAGAAGAAAGAAAGAAAGAAAGAAAGAAAGAAAGAAAGAAAGAAAGAAAGAAAGAAAGAAGAAAGAGAAAGAAAGGAAAGAAGGAAGGAAGGAAAGAAGGAAGGAAGGAAGGAAGAAAGAAAGAAAGAAAGAGAGGAAGGAAGGAAAC | 122177433 |
| 932 | AAAAAAAAAAAAAAAAAAAAAAAAAAAAAAAAAAAAAAAAAGAAAAAGAAAGAAAGAAAGAAAAAGAAAAAAAAAGAAGAAAGAAAAGAAAT | 122445686 |
| 933 | AGAAAGAGAGAGGGAAAGAGAGAGAAAGAGAGAAAGAGAGAGAAAGAAAGAAAGAAAGAAAGAAAGAAAGAAAGAAAGAAAGAAAGAAAGAAAGAAAGAAAGGAAGGAAGGAAGGAAGGAAGGAAGGAAGGAAGGAAGGGAGAAAGGAAGGAAGGAAGGGAGAAAGAAAGAAAGAAT | 122607489 |
| 934 | AAAGAAAGAGAGAGAGAGAAAAGAAAGAAAAGAAAAGAAAAAAAAGAAAGAAAAAAAGAAAAGAGAGAGAGGAAGGGAAAGGGAAAGAGAGAGGAAGGAAGC | 122649445 |
| 935 | AAAAGAAAGAAAGGAGAGAGAGAAAGGAAAGGAAAGGAAAGGAAAGAAAAGGGAAAGAGAGAAAGAGC | 122649547 |
| 936 | GAAGGAAAGGAAGGAAGGAAAGAAGGAAGGGAGGGAGGGAGGAAGGGAAGAAAGGAAGAAT | 122749066 |
| 937 | AAAGGAAAGAAGGAAGGAAGGGAGGGAGGGAGGGAGGGAGGGAGGGAGGAAGGAGGGAGAAAAAAAGAAAT | 123156210 |
| 938 | GAAAGAAAGAAAGAAAAGAAAAGAAAGAGAGAGAGAGAAAGAAGGAAAGAAAGAGGC | 145232942 |
| 939 | AAAAAAAAAAAAAAAAAAAAAAAGAAGGAAAGAAAGAGAGAGGAAAGAAAGAAAAGAAAAGAAAAGAAAAGAAT | 145233282 |
| 940 | AAAAAAAAAGAAAGAAAGAAAGAAAGAAGGAAAGAAAGAAAGAAGGAAGGAAAGAAGGAAGGAAGGGAGGGAGGGAGGAAGGAAGGAAAGAAAGAAAGAAAGAAAGAGAAAAAGAAAGAAAGAAAAGAAAAAAGAAGAGAAAAAGAAT | 145720665 |
| 941 | GAAGGAAGGAAGGAAGGAAGGAAGGAAGGAAGGAAGGAAGGAAGGAAGGAAGGAAAAGT | 145725002 |
| 942 | AAAAAAAAAAAAAAAAAAAAGAAAGAAAAGAAAAGGAAAAGGAAGAAAAAAT | 145760339 |
| 943 | AAAAAAAAAAAAAAAAAAAAAAAAAAAAGAAAAAAGAAAAGAAAGAAAGAAAGAAAGAAAAAAAGC | 145876478 |
| 944 | GAGAGAGAAGAGAGAGGGGAGAGAGGAGAGAAAAAAGAAAAGAAAAGAAAAAGAAAAAGAAAGAAAGAAC | 145930883 |
| 945 | AGAGAGAGAGAGAGAGAGAGAGAGAGAGAGAGAGAGGGAGGGAGAGAGAGAGAGAGAT | 146099761 |
| 946 | GAAAAGAAAGAGAGAGAAAGAAAGAAAGAAAGAAAGAAAGAAAGAAAAGAAAGAAAGAAAGAAAGT | 146255791 |
| 947 | GAGGGAGGAAGGGAGGGAGGAAGGGAGGGAGAGAGAGGAAGGAAGGAAGGAAAGAAGGAAGGAGGGAAGAAGGGAAGGAGGGAGGGAGGGAGGAAGAAAAT | 146255857 |
| 948 | GAAAGAAAGAAAGAGAAAGGGAAAGAAAGAAAGAAAAGAAAGAAAGAGAAAAAAAAAGGAAAGAAAGAC | 146255958 |
| 949 | AAAAAAAAAAAAAAGAAAGAAAGAAAGAAAGAAAGAAAGAAAGAAAGAAAGAAAGAAAGAAAT | 146273644 |
| 950 | GGAGGGAGGGAGGGAAGGAGGGAGGGAGGAAGGAAGGAAGGGAGGGAGGAAGGAAGGC | 146365515 |
| 951 | GGAAGGAAGGAAGGGAGGGAGGGAGGGAGGAAGGAAGGAAGGAAGGAAGGAAGGAAGGAAGGAGGGAAGGAAGGAAGT | 146570379 |
| 952 | AAAAAGAAAGAGAGAGAGAAAGAAAGAAAGAAAGAAAGAAAGAAAGAAAGAAAGAAAGAAAGAGAAAGAAAGAAAAGAAAAT | 146579243 |
| 953 | AAAAAAAAAAAAAAAAGAAAAAAAAAAGAAAAAGAAAAAGAAAAAGAAAAAGAAAAAAGAAAAAGAAGAAAGAAAAAGAGAAAGAAAGAAAAT | 146858948 |
| 954 | AGAAAAGAAAGAAAAAGAGAGAGAGAGAAAGAAAGGAAAGAAAGAAAGGAAGAGAGAGAGAAAGAAAGAGAAAGAAAGAAAGAAAGAAAGAGAAAGAAAGAAAGAAAGAAAGAAAGAAAGAAAGAAAGAAAGAAAGAAAGAAAGAAAGGAAAAAC | 147420316 |
| 955 | GAAAAGAAAGAGAGAGAAAGAAAGAAAGAAAGAAAGAAAGAAAGAAAGAAAGAAAGAAAAGAAAGAAAGAAAGAAAGT | 147552780 |
| 956 | GAGGGAGGAAGGGAGGGAGGAAGGGAGGGAGAGAGAGGAAGGAAGGAAGGAAAGAAGGAAGGAGGGAAGAAGGGAAGGAGGGAGGGAGGGAGGAAGAAAAT | 147552858 |
| 957 | GAAAGAAAGAAAGAGAAAGGGAAAGAAAGAAAGAAAAGAAAGAAAGAGAAAAAAAAAGGAAAGAAAGAC | 147552959 |
| 958 | AAAAAAAAAAAAAAAAAAGAAAGAAAGAAAGAAAGAAAGAAAGAAAGAAAGAAAGAAAGAAAGAAAGAAAGAAAT | 147570644 |
| 959 | GAAAAAAAAAAAAAAAAAAAAAAAGAAAAGAAAGAAAGGGAAAGAAAAGAAAGT | 147679338 |
| 960 | AAAGAGAGAGAGAGAGAGAGAGAGAGAGAGAGAGAGGGAGAGAGAGAGAGAGAGAGAGAGAGAGAGAGAGAGAGAGAGAGAGAGAGAGAGAGC | 147690570 |
| 961 | GAAAAGAAAGAAAGAGAAGGAAGGAAGGAAAGAAAGAAAGAGAAAGAAAGGAAGGAAGGAAGGAAAGAAAGAAAGAGAGAAAT | 147738817 |
| 962 | AAAGAAAGAAAGAAAGAAAAAGAAAGGGAGGGAGGGAAAGGAAAGAAGGAAGGAAGGAAGGAAGGAAGGAAGGAAGGAAGGAAGGAAGGAAGGAAGGAAGGAAT | 147738974 |
| 963 | AAAAAGAAAGAGAAGGAAGGAAGGAAAAGGAGGGAAAGAGGGAGGGAGGGAAC | 147761548 |
| 964 | AAAAAAAAAAAGAAAAGAAAAGAAAGAAGGAAAAAAGGAAGGAAGGAAGAAGGAAGGAAGGAAGAAAAC | 147765956 |
| 965 | AAAAAAAAAAAGAAAAAGAAAGGAGAAAGAAAGGAAGAAGAGGAAGGAAGGAAGGAAGGAAGGAAGGAAAAGAAGAAAGAAAGAAGAAAGAAAGGAAC | 148236147 |
| 966 | AAAGAAAGAAAGAAAGAAGAGAAAAAGAGAGAGAGGAAGAAAGAAAGAGAGAAAGAAAGAAAAGAAAAAGGAAGGAAAGAAAGGAAGAAAGAAAGAAAAAGAGAGAAAGAAGAAAGAAGAAAAGGAAAGAAAGAAAGAGAGAGAGAAAGAAGGAAAAGAAC | 148236245 |
| 967 | AAAAAAAAGAAGAAGAAAGAAAGAAAGAAAGAAAGAAAGAAAGAAAGAAAGAAAGAAAGAAAGAAAGAAAGAAAGAAAT | 148246832 |
| 968 | AAAAAAAAAAAAGAAAAAGAAAGGAGAAAGAAAGGAAGAAGAGGAAGGAAGGAAGGAAGGAAGGAAAAGAAGAAAGAAAGAAGAAAGAAAGGAAC | 148398179 |
| 969 | AAAGAAAGAAAGAAAGAAGAGAAAAAGAGAGAGAGGAAGAAAGAAAGAGAGAAAGAAAGAAAAGAAAAAGGAAGGAAAGAAAGGAAGAAAGAAAGAAAAAGAGAGAAAGAAGAAAGAAGAAAAGGAAAGAAAGAAAGAGAGAGAGAAAGAAGGAAAAGAAC | 148398274 |
| 970 | AAAAAAAAAAAAAAAAAAAAAAGAAGAAGAAAGAAAGAAAGAAAGAAAGAAAGAAAGAAAGAAAGAAAGAAAGAAAGAAAT | 148408834 |
| 971 | AGAAAGAAAGAAAAGGGAAGGAAGAAAAGAAAGAAGAAGAGAGGGAGGGAGGGAGAGAGGGAGGGAGGGAC | 148555043 |
| 972 | AAGAAAGAAAGAAAGAAAGAAAGAAAGAAAGAAAGAAAGAAAGAAAGAAAGAAAGGAAGGGAGGAAGGAAAGGAGGAAGAGAGAAT | 148632127 |
| 973 | GAAGGAAGGAAAGAAAAAGAGGAAAGGAAGGGAGGGAGGAAGGAAGAAAAAAGGAAAGAAAGC | 148632256 |
| 974 | AAAGAAGGAAAGAAGGAAGGAAGGAGAAAAAAAGAAGGAAAAGAAAGAAAGGAAAAGAAAAAAGAAAAGGAAGAGGAAAAGAAGAAAGGAAGGAAGAAGGC | 148632420 |
| 975 | GGAAAGAAAGAAAGAAAGAAAGAAAGAAAGAAAGAAAGAAAGAAAGAAAGAAAGAAAGAAAGGAAGGAAGGAAGGGAAGGAAGGAAGAAAAGAAAGAAAGAAAGAAAGAAAGAAAGAAAGAAAGAAAGAAAGAAAGAAAGAAAGAAAGAAGGAAGGAAGGAAAGAAAGAAAGGGAGAGAGAGAGAGAAAAGAAAAGAAAAGAGGAAGAAAC | 148788741 |
| 976 | GAAAGAAAGAAAAAGAAAGAAAGAGAGAGAGAGAAAGAGAGAAAAGAAAGAAT | 149030965 |
| 977 | GAAAAAAGAAAAAGAAAGGAAAGAGAAAGAAAAGAAAAGAAAAAAAAAGAGAAAAAAC | 149150151 |
| 978 | AAAAAGAAAGGAGAAAGAAAGGAAGAAGAGGAAGGAAGGAAGGAAGGAAAAGAAGAAAGAAAGAAAGGAAC | 149178106 |
| 979 | AAAGAAAGAAAGAAAGAAAGAAGAGAAAAAGAGAGAGAGGAAGAAAGAAAGAGAGAAAGAAAGAAAAGAAAAAGGAAGGAAGGAAAGGAAGAAAGAAAGAAAAAGAGAGAAAGAAGAAAGAAGAAAAGGAAAGAAAGAAAGGAAGAAAGAGAGAAAGAAGGAAAAGAAAGAAAAT | 149178177 |
| 980 | AAAAAAAAAAAAAAAAAAAGAAGAAAGAAAGAAAGAAAGAAAGAAAGAAAGAAAGAAAGAAAGAAAGAAAT | 149189308 |
| 981 | GAAAGAAAGAAGAAAGAAAGAGAGAGAGAGAGAGAAAGAGAGAAAAGAAAGAAT | 149415966 |
| 982 | AAAAAAAAAAAAAAAAAGAAAAAAGAAAAGAAAAAAAAGAAAGAAAAAGAAAAAAC | 149444364 |
| 983 | AAAAAAAGAAAGAGGAAGGAAGGGAAGGAAGGGAAGGAAGAAAGGAAGGAAGGAAGGGAGGGAGGGAGGGAGGGAGGGAGGGAGGGAAAGAAAGAAGAAAAGAAAAAAAGAAAAGC | 149466767 |
| 984 | AAAAAAAAAAAAAAAAAAAAGAAAGAAGAAAGAAAGAAAGAAAGAAAGAAAGAAAGAAAGAAAGAAAGAAAAAAT | 149474104 |
| 985 | AGGGAGGAGGAGGAGGAGGAAGAGGAGGAGGAAGAGGAGGAGGAGGGGGAGGGGGAGAAGAAGAAAAGC | 149589614 |
| 986 | AAGAAGAAGAAGAAGGAGGAGGAGGAGGAGGAGGAAGAAGAAGAAGAAGAAAGT | 149641253 |
| 987 | AAGAGAGGAAGAGAGGAAGAGAGGAAGGAAGGGAGGGAGGGAGGGAGGGAGGAAAGGAAGGAGAGT | 149830371 |
| 988 | GGGAGGAAGGGAGGGAGGGAGGGAGGGAGGGAAGGAAGGAAGGAAGGAAGGAAGGAAGGAAGGAAGGAAGGAAGGAAAT | 149830486 |
| 989 | AAGAAAGAAGGAAAGAAGAAAGAGAGAGAGAGAAAGAAAAGAAAAGAAAGAGAGGAAGGAAAGAAGGAAGGGAGGGAAGT | 150148798 |
| 990 | GGAGGGAGGGAGAGAGAGAGAAAGAAAAGAAAGAAGGGAGGGAGGGAGGAAGGAAGGAAGGAAGGC | 150148878 |
| 991 | AAAAAAAAAAGGAAAAGAAAGAAAAAGGAGAGAAAGAAAGAAAGAAGAAAGAAAAAAAGAAAGAAAGAGAAAAGAGGGGAGGAGAGAAGAAAGAAAAGAAAGGT | 150151484 |
| 992 | GGGGGAAAAAAAAGAAAAAGAGAGAAAAAGAAGAGAGAAAGAGAGGAGAGAGAGAGGGAAAGAAGGAAAGAAGAGAT | 150204481 |
| 993 | GAGGAAAAAAAGGAAGAGAAGAGAAGGGAGGGGAGGGGGAGGGGGAGGGGGAGGGGAGGGGGAGGGGGAGGGGAGGGAGGGGGAGGGGAGAGGGGGAAGAGGC | 150204803 |
| 994 | AAAAAAGAAGGAAGGAAGGGGGGAGGGAGGAAGGGAAAAAAGGAAGGAGGGAGGGAGAGGAAAT | 150451590 |
| 995 | AAAAAAAAAAAAAAAAAAAAGGGAAGGGGAAAGAGAGGGAAGGAGGGAGGGAAAGGT | 150463941 |
| 996 | AAAAAAAAAAAGAAAGAAAGAGAGAGAGAGAGGAAGGAAGGGAGGGAGGGAGGGGAGT | 150763562 |
| 997 | GAAAAAGAAGAAAAGAAGGAGGAGGAGGAAGGAAGGAGAAGAAGAGGAAGAAGGAGAAGAAAGAAAAAGAGGAGGAGGAAGAAT | 150971054 |
| 998 | AAGGAGAAGGAAAAGAGGAAGAAGGAGGAGGAGGAGAAGAGGAAGAGGAGGAAGAGGC | 150971138 |
| 999 | AAAAAAAAAAAAAAAAAAAAAAAAAGAAAAAGAAAGAAAAGAAAAAAAAAAAGAC | 151025294 |
| 1000 | GAAAAAAAAAAAAAAAAAAGAAAAAGAAAAAAGAAAGGAAAAAGAAGGAAAC | 151480408 |
| 1001 | AAAAAAGAAAAAAGAAAAAGAAAAAAAGGAAAGAAAGGGAAGGAGGGAAGGAAGGAAGGAGGGAAGGAAGGAAGGAGAC | 151587690 |
| 1002 | GGAGAGGAGAGGGAGAGGGAGAGGGAGAGGGAGAGGGAGAGGGAGAGGGAGAGC | 151754777 |
| 1003 | AAGAAAGAAAGAAAGAAAGAAAGGAAGAAAAGAAGGAAAAGAAAAAGAAAGAAAGAAAT | 152171864 |
| 1004 | AAAAAGGAAGAGAGAGAGAGAGAAAGAGAGAGAGAGGAGAGAAGAGAAGAGAGAT | 152397657 |
| 1005 | AAAAAGAAAAAGAAAAAGAAAGAAAAGAAAAAAGAAAGGAAGAAAGAAAGAAAGGAAAGGAAAGAAAGAAAGAAAGAAAAAAGAAGGAAGGAAGAAAGAAAAAAAGAGAGGAAGAAGGAAGGC | 152605716 |
| 1006 | AAAAAGGAAAAAAGAAAGAAAGAAGAGAGAGAAAAAAGAAAAGAAAGAGAGAGAGAAAGAAAGAAAGAAAGAGAAAGAAAGAAAGAAAGAAAGAAAGAAAGAAAGAAAGAAAGAAAGAAAGAAAGAAAGAAAGAAAGAAAAGAAAGAGAGAGAGAGAGGGAGGGT | 152684175 |
| 1007 | AAGAAGAAGAAGAAGAAGAAGAAGAAGGAGGAGGAGGAGGGAGGAGGAGGGAGGAGGAGGAGGAGGGAGGAGGGAGAAAGGAGAGAC | 152684654 |
| 1008 | GAAAGAAAGAAAGAAAGAAAGAAAGAAAGAAAGAAAGAAAGAAAGAAAGAAAAGAAAGAAAGAAAGAAAGAAGGAAAAAAT | 152729981 |
| 1009 | GAAAGGAAGGAAGGAAGGAAGGAAGGAAGGAAGGAAGGAAGGAAGGAAGGAAGGAAAGAAAGAAAAAGAAAGAAAGAAAGAAAGAAGAAAGAAAGAAAGAAAGAAAGAGAAAGGAAGC | 153063444 |
| 1010 | AAAGAAGGAAGGAAGGGAGGGAGGGAGGGAGGGAGAGAGAGAGAGGGAGAGAGAGAGAAAGAAAGAAAGAAAGGAAAGAAAGAAAGAAAAAAGAAAGAAAGAAAGAGAGAAAGAAAGAAAGAAAGAAAGAAAGAAAAAAGAAAGAAAGAAAGAGAGAAAGAAAGAAAGAC | 153116506 |
| 1011 | AAAAAAAAAAAAAAAGAAAGAAAGAAAAGAAAAAAGAAAGAAAGAAAAGAAAGAAAGAAAGAAAGAAAGAAAGAAAGAAAGAAAGAAAGAAAGAAAGAAAGAAAGAAAGAAAAC | 153206793 |
| 1012 | AGAGAGGGAGAGAGAGAGGGAAGGAAGGAAGGAGAAAAAAGGAAAGAAAGGAAGAAAGAAAAAGAAAGAAAGGAAGAAAAGAAAGAGAGAGAAAGAAAGAAAGAGAAAGAGAGAGAGAAAGAAAGAAGAAAGAAAGAAAGAAAGAAAGAAAGAAAGAAAGAAAGAAAGAAAGAAAGAAAAGAAAGAAAGAAAGAAAGAAAGAAAGAAAGAAAGAAAAGAAAGAAGGAAAGAGAAAGAGAGAGAAGC | 153271030 |
| 1013 | GAAAAAGAGAAAGAAAGAGGAGAGAGAGAGAGAGAGAGGGAGGGAGGGAGGGAGGGAGGGGGAGGGAGGGAGGGAGGGGGAGAGAGAGAGAGGGAGGGAGGGAGGGAGAGAGAGAGAGAGAGAGAAGAGAGAGAGAGAGAGAAAAAGAAAGAAAGAAAGT | 153286103 |
| 1014 | AAAAAAAAGAAAAAGAAAGAAAGAGAGAGAGAGAGAAAGGAAGAAAGGAAGGAAGAAAGAAGAAAGAAAGAAAAGAAAAGAAAGAAAGT | 153291194 |
| 1015 | GGGAGGGAAGGAGGGAGAAAGAGAGAGAAAGAAGGAAAGAAGGAAAGGAAGGGAGGAAGGAAGGAAGGAAGGAAGGAAAGAAAGAAAGAAAGAAAGAAAGAAAGAAAGAAAGAAAGAAAGAAAGAAAGAAAGAAAGAAAGAAAGGC | 153342624 |
| 1016 | AGAAAGAGGAAGGAAGGGAGGAAGGAAGGAAAGAAGGGAGGAAGGGAGGAAGGAAAGGGGAGGAAGGGAGGGC | 153473657 |
| 1017 | AAAAGAAAAGAGAAAGAAAGAAAAAGAAAAAGAAGAAAGAAAGAGAAAGAAAGAGAAAGGGAGGGAGGGAAAGAAAAGAAAAGGAAGGAAGGAAAGAAGGAAGGAAGGAAGAAAAAGAAAGAAGGAAAGAAAAAGAAAGAAAGAAAAGAAAGAAAGAGAAAGAAAGAAAGAAAGAAAGAAAGAAAGAAAGAAAGAAAGAAAGAAAGAAAGAAAGAAAGAAAGAAAGAGAGAGAGAAAGAAAT | 153473745 |
| 1018 | AAAAAAGGAAAAGAAAGGAAAGGAAAAAGGGAAGGGAAAGGAAAGAAAGAGGT | 153657144 |
| 1019 | AAAAGAAAGAAAGAAGAAAAGAAAAGAAAAGAAAAGAAAAGAAAAGAAAAGAAAAGAAAGAAAGAAAAAAAGAAAGAAAGAAAAAGAAGGAAGGAAGGAAGGGAAGGGAAGGAAGGAAAT | 153798884 |
| 1020 | GAAAAAAAAAAAAAGAAAAGAAAAAAAAAGAAAAGAAAAAGAAGGAAGAAAT | 153846921 |
| 1021 | GAAAGGAAGGAAGGAAGGAAGGAAGGAAGGAAGGAAGGAAGGAAGGAAGGAAGGAAGGAAGGAAGGAAGGAAGGAAGGAAGGAAGGAAGGAAGGAAGGAAGGAAGGAAGGAAGGAAGGGAGGGAGGGAAAT | 153891567 |
| 1022 | AAAAAAAAAAAAAAAAAAAAAAAAAAAAAAAAAAAAAAAAAAAAAAAAAAGC | 153955078 |
| 1023 | AAAAAAAAAAAAAAAAAAAAAGAAAGAAAGAAAGAAGGAAGGAAGGAAGGAAT | 154064145 |
| 1024 | AGGAGGGGAGGGGAGGAGAGAGGAGAAAAGAGAAGAGAAGAGGAGAAAAGAGAAGAGAAGAGAAGAGAAGGT | 154494562 |
| 1025 | AAGAAAGAAAGGAAGAAAGGAAGGAAGAAAGGGAGAAAGAAAGGAAGAAAGAAAGAAAGAAAGAAAGAAGAAGAAAGGAAAGGAAGGAAGGAAGAAAGAAAGAAAGAGAGAGAGAGAAAGAAAGAAAGAAGGGAGGGAGGGAGGGGGAGGGAGGGAGGGAAGGAAGGAAAGAAAGAAAAGAAAGAAAGAAGAAAGGAAGGAAGGAAGAAAGAGGGAGAGAGGGAGAAGGAGAAGGGAGAAGAAGAAGAGGAAGAGGAAGAAGGAAAAGGAGAAGAAGAAGGC | 154521407 |
| 1026 | AAAGAAGAAGAAGGAGGAGGAGGGGGAAAGAAGGAAGAAGGAAGAAGAAGGAAGAGGAAGAT | 154521762 |
| 1027 | GAGGAGGAAGAGGAGGAAGAAGAAGGAGGAGAAGAAGAAGAGAAGAAGAAGAGGT | 154521824 |
| 1028 | GAAAAGGAGGAGGAGGAGGAAGAGGAAGAGGAAGAAGAAGGAGGAGGAGGAGGGGGAAAGAAGGAAGAAGGAAGAAGAAGGAAGAGGAAGAT | 154521980 |
| 1029 | GAGGAGGAGGAAGAGGAGGAAGAAGAAGGAGGAGAAGAAGAAGAGAAGAAGAGGT | 154522072 |
| 1030 | GAAAAGGAGGAGGAGGAGGAAGAGGAAGAGGAAGAAGAAGAAAAAGAAGAAGAGGAGGAGGAAGAAGAAGAAAC | 154522228 |
| 1031 | AAGAAAAGAAAGAGAGAGAGAGAGAGAGGAGAGAGAGAGAGAGAAAGGGAGGGAGGGAGGAGGGAGGAAGGGAGGAAAAGAAAGGAGGAAAT | 154869897 |
| 1032 | GGGAGGAGGGGAGAGGAGGGGAGAGGGGGAGAGGAGGAGAGAGGGGAGAGAGGGGAGAGGGGAGAGGC | 154896406 |
| 1033 | AAAAAAAAAAAAAAGAAAGAGAAAAGAAAAGAAAGAGAGAGAGAGAGAAGGAAGGAAAGAAAGAAAGAAAAAGAAAAAGAAAGAAAGAAAGAAAGAAAGAGGAAGAAAGAAAAAT | 154989540 |
| 1034 | GGGGAGGAGAGGGGAGGAGAGGAGGAGGGGAGGAAGGGAGGAGGGGAGGAGGGGGAT | 155596988 |
| 1035 | AAAAAAAGGGGAAAAAAAGAAAAAGAAAGAAAGAAAAAGAAGGAAAGAAAGAC | 155620011 |
| 1036 | AAAAAAAAAAAAAAAAAAAAAAGAAAGAAGAAAGAGAGAGAGAGAGAAAGAAAGAGAAGAGAAAAGAAAAGAAAAAT | 156175132 |
| 1037 | AAAAAAAAAAAAAAAAAAAAAAAAAAAAAAAAAAAAAAAAAAGAGAGAGAGAGAGAGAGAAAGGAAC | 156211163 |
| 1038 | AAAAAAAAAGAAAAGAAAAGAAAAAAGAAAAAAAAAGGAAAAGAGGAAAAAC | 156807814 |
| 1039 | AGAAAGAGAGAGAAAGAGAAAGAGAGAGAGAGAGAGAGAGAGAGAGAAGAGT | 157236773 |
| 1040 | AGAAAGAAAGAAAGAAAGAAAGAGAGAAAGAGAGAAAGGAAGAAAGGAAGGAAAC | 157258081 |
| 1041 | AAAAAGAAAGAAAGAAAAGAAAGAAAGAAAGAAGGAAGGAAGGAAGGAAGGAAGGAAAGAAAGAAAGAAAGAAGGAAAGAAAGAAAGAAAGAAAGAAAGAAAGAAAGAAAGAAAGAAAGAAAGAAAGAAAGAAGAAAGAAT | 157523885 |
| 1042 | GAAAAAAGAAAGGAAGGAAGGAAGGAGGGAAGGAAGGAAGGAAGGAGGGAAGGAAGGAAGGAAGGGC | 157524026 |
| 1043 | AAAAAAAAAAGAGAGAGAGAGGGAGAGAAAGAAAAGAAAGAAAGAAAGAAAAAGAAAAAAGAAAGAAAAGAAAGAAAAAGAAAGT | 157641968 |
| 1044 | GGAAGGAAGAAAGGAAAGAAGGAAGAAGGAGGAAGAGAAGGAGGGGAAAAGAAT | 157685548 |
| 1045 | AAAAAAAGGAAGAAGAAGAAGAAGAAGGAGGAGGAGGAGGAGGAGGAGAAGAAGAAGAGGAGGAGGAGGAGGAAAAGGT | 157719704 |
| 1046 | AAAAAAAAAAAAAAAAAGAAAAGGAAAGAAAAAAGAAAAGAGAAGGGAAGGGAAGGGAAAGGAAGAGAAAAGAAGAAAAGAGAAAAGAAAAGAAAAGAAC | 157723141 |
| 1047 | AAAGAAAGAAAGAAAGAAAGAAAGAGAGAGAGAGAGAGGGAGGAAGGAAGGAAGGAAGGAAGGAAGGAAGGAAGGAAGGAAGGAAGGAAGGAAGGAAGGAAGGAAGGAAGAAAAAAAAC | 157745294 |
| 1048 | AGGAGGAGGAAGAAGAGGAAGAAGAAGAGGAGAAGGAGGAGGAGGAAGAGGAGGAC | 157926798 |
| 1049 | GAAAGAAAGAAAGAAAGAAAGAGAAAGAAAGAGAGAGAAAGGAAAGGAAAGGAAAGGGAAGT | 159406208 |
| 1050 | AAGGGAGGGAGAGAGGGAAGGAAGGAAGGAAGGAAGAAAGGAAGGAAGGAAGGAAGGAAGGC | 159406280 |
| 1051 | AAAAAAAGAGAAAGAAAAGAGAGAGGGGGAGGGGAGGGGGAGGGGAAGGGGGAGGGGAGGGGGGAGGGAGGGGGAGGGGAGGGGAGC | 159406646 |
| 1052 | AAAAAAAAAGAGAAAGAAAGAAGGAAAGAAAAAAAGAAAGAAAGAAAGAAAGAAAGAAAGAAAGAAAGAAAGAAAGAAAGAGAAAGAAAGAAGGAAAGAAGGAAAGAAGGAAAGAAAGAAAGAAAGAGAAAGAAAGAAAAGAAGAT | 159740141 |
| 1053 | GAAAGAAAGAAAGAGAGAGAGAGAGAGGGAGGGAGAGAGGGAGGGAGGAAGGAAGGAAAGAT | 159778746 |
| 1054 | AGAAAAAAAAAAAAAAAAGAAAGAAAGAAAAGAAAAGAAAGAAAGAAAAAGAT | 159908767 |
| 1055 | AAAAGAAAGAAAGAGAGAAAGAAAGGAAGGAAGAAAGGAAAGAGAGAAAGAAAGAAAAGAAGAAAT | 159912339 |
| 1056 | AGAAAAAAAAAAAAAAAAGAAAGAAAGAAAAGAAAAGAAAGAAAGAAAAAGAT | 160065629 |
| 1057 | AAAAGAAAGAAAGAGAGAAAGAAAGGAAGGAAGAAAGGAAAGAGAGAAAGAAAGAAAAGAAGAAAT | 160069201 |
| 1058 | AAAAAAAAAAAAAAAAGAAGAAAGAAAGGAAGAAAGAGAGAGAGAAGGGAAGAGAAGAGAGGAAAAAGAAAAAGAAGGGAGGGAGGGAGGGAGGGAGGC | 160602580 |
| 1059 | AGAGAGGGGAGGGGGGAGAGAGAGAGAGAGAGAGAGAGAGAGAGAGAGAAAGAGAT | 160687397 |
| 1060 | AGAAAGAGAAAGAAAGAGAGAGAAAGAAAGGAAGGAAGGAAGGAAGGGAGGGAGGGAGGGAGGGAGGGAGGGAAT | 160739932 |
| 1061 | AAGAAAAAAAAAAGAAGAAAGAAAGAAGAAAGAGAGAAAGAAAGAGAGAAAGGAAAGAAAGGAAAGGAAAGAAAGAAAGGAAGGAAGGAAGGAAAGAAAGAAAGAAAGAAAGAAAGAAAGAAAGAAAGAAAGAAAGAAAGAAAGAAAGAAAGAAAGAAAAGAAAGAAAGAAAAAGAAAAGAAAAGAAAAAAT | 160748875 |
| 1062 | AGGGAAAAAAAAAAAAAAGAAGAAAGAGGAGGAGGGGGAAAAGGAGGGAAAAC | 160933252 |
| 1063 | GAGAGAGAGAGAAAGAAAGAAAAAGGGAAGGAAAAGGAAGGGGAGGGGAGGGGAGGGAAGGGAGAAGGAAGGC | 160952048 |
| 1064 | AAGAAAGAAAGAGAAAGAAAGAAAGAAAGAAAAAGAAAGAAAGAAAGAAAGAAAGAAAGAAAGAAAGAAAGAAAGAAAGAAAGAGAAAGAAAGAAAGAAAGGAAAGGAAGGAGAGGAAGGAGAGGAAGGAGAGAAAAGAGAGAGGAAGGAAGGGAGGGAGGGAAGGAAGAGAAAAGGAAAGGAAGGGAAGGGAAGGGAAGAAGGGAAGGGAAGGGGGAAAAAGC | 160984871 |
| 1065 | AAAAAAAAGAAAGAAAGAGAGAGAGAAAAGAAAGAAAGAAAAAGAAAGAAAGAAAGAGAAAGAGAAAGAAAGAAAGGAAGAAAGAAAGAT | 161350942 |
| 1066 | AGAAAGAAAGAAAGAAAGAAAGAAAGAAAGAAAGAAAGAAAGAAAGAAAGAAAGAAAAGC | 161351032 |
| 1067 | GAAAGAAAGAAAGAAAGAAAGAAAGAAAGAAAGAAAGAAAGAAAGAAAGAAAGAAAGAC | 161362975 |
| 1068 | GAAAGAAAGAAAGAAAGAAAGAAAGAAAGAAAGAAAGAAAGAAAGAAAGAAAGGAAGAAAGAAAGAAAGAAAC | 161377757 |
| 1069 | GAAAGAAAGAAAGAAAGAAAGAAAGAAAGAAAGAAAGAAAGAAAGAAAGAAAGAAAGAAAC | 161392546 |
| 1070 | AAGAAAGAAAGAAAAAGAAAGAGAAAGAAAGAAAGGAAGAAAGAAAGAAAGAAGGAAGGAAGGAAGAAAGGAAAGAAAGAAAGAAAGAAAGAAAGAAAGAAAGAAAGAAAGAAAGAAAGAAAGAAAGAAAGAAAGGAAGGAAGC | 161499848 |
| 1071 | AGAGAGAGAGAGAGAGAGAGAGAGAGAGAGAGAGAGAGAGAGAGAGAGAGAGAGAGAGAGAGAGAGAGAGAGAGAGAGC | 162100176 |
| 1072 | AAAAAAAAAAAAAAAAGAAGAAAAAAGAAAGAAAGAAAAGAAAAGAAAAGAAAC | 162201152 |
| 1073 | AAAAAAAGAGAGAGAGAAAGAAAAGGAAGGAAGGAAGGAAGGAAGGAAGGAAGGAAGGAAGGAAGGAAGGAAGGAAGGAAGGAAAGAAAGGAAAAT | 162230857 |
| 1074 | GAAAGGAAGGAAGGAAGGAAGGAAGGAAGGAAGGAAGGAAGGAAGGAAGAAAGAGAAAGAAAGAAAGAAGAGAAAGAGAAAGAAAGAAAAAGAAAAGAAAGAGAGAAAGGGAAGGAAAGAAAGAGAGAAAGAAAGAAGAAAGAGAGAGAAAGAAAGAAAAAGAAAGAAAGAAAGAAAGAAAGAGAAAGAAAGAAAGAGGGT | 162254974 |
| 1075 | GGAGAAAGAAAGAAAAAGAAAAAAAAGGAAAAGAAAAGAAAAGAAAAGAAAAGAAAAGAAAAGAAAAGAAAAGAAAGGAAGAAAGAAAGAAAGAAAGAAAGAAAGAAAGAAAGAAAGAAAGAAAGAAAGAAAGAAAGAGAGAGAAGT | 162283052 |
| 1076 | AAAAAAAAAAAAAAGAGAAAGAAAGAAAGAAAAAAGGAGAGAGAGAAAGAAAGAAAGAAAGAAAGAAAGAAAGAAAGAAAGAAAGAAAGAAAGAAAGAAAT | 162326586 |
| 1077 | GAGAGAGAGAGAGAGAGAAAGAGAGAGAGAGAGAGAGAGAGAGGGAGAGAGAGAAT | 162376096 |
| 1078 | AGAAAAGAAAAAGAAAGGAAAGAAAGGAAGAAGGAAGGAAGGAAAAGAGGGAGGAAGGGAGGGAAAGGAAGGAAAGGAAGAAAT | 162449513 |
| 1079 | GAGAGGAGAAGAAAAGAGGAGAGGAAAGAAGAGAAGAGAGGAGGAAAGGAGAAGAGAAGAC | 162467937 |
| 1080 | AGAGAAAGAAAGAGAGAAAGAAAAGAAAGAAAGAAGAAAGAAAGAAAAAGAAAGAAAGAAAGAAAGAAGGAAGGAAGGAAGC | 162678513 |
| 1081 | AAAAAAAAAAAAAGGAAGGAAGGAAGGAAAGAAGGAAGGAAGGAAGAAGGAAGGAAGGGGGGAGGGAGGGAAGGAAGGAAGGAAGGAAGGAAGGAAGGAAGGAAGGAAGGAAGGAAGGAAGGAAGGAGGGAAGGC | 162678887 |
| 1082 | GAAGGAAGGAAGGGAGGGAGGAAGGAAAGAAGGAAGGAAGGGAGGAAGGAAGGAAAGT | 162943803 |
| 1083 | AAAGAGAAAGAGAAAGAAGGAAAGGAAGAAGAAAGAAAGAGGAAAGAAAAGAAAGAAAGGAAAAGAAAGAAAAAAGAAAGAAAGAAGAGAAAGAAAGAAAAGT | 163606155 |
| 1084 | AGGAAGAGAGAAAGGGAGGGAGGGGGGGAGAGAGAGAGAGAAAGAAAGAGAAGAAAAAGAAGAAAAAAGT | 164010763 |
| 1085 | GGAGGGAGGGAGAGAGAGAGAGAAAGAAAGAGAAAGGAAAGAAAGAAAGGAAGGAAGAGAGAGAGAAAGAAAGAAAGAAAAAAAGC | 164109890 |
| 1086 | GAAAGGAGGAAGAAAGGAAGGAAGGAAGAAGGAAGGAAGGAAAAGAAAAAGGAAGAAGAGAAAGAAAAGAGAGAGAAAGAAAGAAAGAAAAGAAAGAGAAAGAAAGAAGAAAAGAAAGAAAGAAAGAAAGAGAGAAAGAAAGGAAGAAAGAT | 164110008 |
| 1087 | AGAAAGGAAGAAAGAAAAAGAAAAGGAAGGAAGGAAGGAAAAAAGAAGAAAGAAAAGAAAGAAAGAAAAAGAAAGAAAGAAAGAAAGAAAAGGAAGGAAGGAAGGAAGGAAGGAAGGAAGGAAGGAAGGAAGGAAGAAAGAAAGAAAGAAAGAAAGAAAGAAAGAAAGAAAGAAAGAAAGAAAAGAAAGAAAAGAAAGAGAAAGGAAGAAAGAAAGAGAAAGAAAAAAGAGAGAGAGGAAGGGAAGGAGT | 164114545 |
| 1088 | GAGGGAGGGAAGAAAGAAAGAAAGAGAGAAAGAAGAGGAAGGAGGGAGAGAAGGGAAAAGAAAAGAAAAAGAAAGGAAAGAGAAAGAAAGAAAAAT | 164114795 |
| 1089 | GGAAGGGGAGGGGAAGGGAGGGGAGGAGAGGGGAAGAGAAGGGAGGGGAGAGC | 164192527 |
| 1090 | GGAAGGAAAGGAAGGAGAGGAGAGGAGAGGAGGGAGGAGGGGAGGGAAGGGGAGGAGAGAAGAGAGGAGAGGAGAGGAGGGAGGGAGGGAGGAAGGAAGGAAGGAAGGAAGGGGT | 164278119 |
| 1091 | AAGAGAAAGAAGAAAAGAAAAGAAGAGAAGAGAAAAGAGGAGAGGAGAGGAGAAGAGAAGAGAAAAGAGGAGAGGAGAGGAGAAGAGAAGAGAAAAGAAGAGAGAGAGAGAGGGAGGGAGAGAGGGAGGGAGGGAGAGAGAGAGGGAGGAC | 164291827 |
| 1092 | AGGGAAGGGAAGGGAAGGGAAGGGAAGGGAAGGGAAGGGAGAAAAGAGGAAAGGAAGGGAGAGGAAAGGAAAGGAAGAAAGAGAGAGAAAGAAAAAGAAAAGAAAGAGGGGGAAGGGAAGGGAAGAAGGGAAGGGAAGAGAAAAAAGAGAGAGAGAAGAT | 164292005 |
| 1093 | AGAAAGAAAGAAAGAAAGAAAGAAAGAAAGAAAGAAAGAAAGAAAGAAAGAAAGAAAGAAAGAAAGAGAGAGAAAAGAAAGAAAGAAAGAAAGAAAGAAAGAAAAAAAGAAAGAAAGAGAGAGAAAGAAAGAAAGAAAGAGAGAGAGAGAAAGGAAGAAAGAAAGAAAGAAAGAAAGAAAGAAAGAAAGAAAGAAAGAAAGAAAT | 164345751 |
| 1094 | GGGGGGAGGGGAGAGGAGAGGAGAGGAGAAGAGAGAAGAGAAGAGAAGAGAAGAGAAGAGAAGAGAAGAGAAGAGAAGAGAAGAGAAGAGAAGAGAAGAGAAGAGAAGAGAAGT | 164379248 |
| 1095 | AGAGAGAAAGGAAGGAAAGAGGGAAGGAAGGAAGGAAGGAAGGAGGAAAAGAAGGAAGGAAGGAAGGAAGGAAGGAAGGAGGAAAAGAAGGAAGGAAGGAAGGAAGGAAGGAAGGAGGAAAAGAAGGT | 164719437 |
| 1096 | GAGAGAGAGAGAGAGAGGAAGGAAGGGAGGAAGGAAAGGAAAGGAAAGGAAAGGAAAGGAAGAAGAAAAAAGGGGAGGAAAAGT | 164719717 |
| 1097 | AGGAAAGAAGGAAGGAAGGAAGGAAGGAAGGAAGGAAGGAAGGAAGGAAGGGAGGGAGGGAGGGAGGGAGGGAGGGAGGGAGGGAGGAAGGAAGGAAGGAAGGAAGGGAGAGAGGGAGGGAGGGAGGGAGGAAGAAAGGAAGAAAC | 165023630 |
| 1098 | GAGGAGAAAGAAAGAAAAAAGAAAGAAAGAAAGAAAGAAAGAGAGAGAGGAAGGAAGGAAGGAAGGAAGGAAGGAAGGAAGGAAGGAAGGAAAGAAGGAAAGGAAAGAGAGAGAAAGAAAGAAAGAGAAAGAGAGAGAGAAAGAAGAGAAGGAGAGAAAGAGAGAC | 165319805 |
| 1099 | AGGAGGGAGGGAGGGAGGAAGGAAGGAAGGAAGGGAAGAAGGGAGGGAGGGGAGAC | 165319971 |
| 1100 | AAGGAAGGAAGGGAAAGAAAGGAAAGAAAGAAAGAAAGAGAGAGAAAGAAAAGAAAAGAAAAAAAGAAAGAAAAGAAAGAGAGAGAGAAAGAAAGAAAGAAGGAAGGAAGGAAGGAAGGGAAGGAAGGAAGGT | 165320075 |
| 1101 | AAAAAGAAGGAAGGGAGAAAGAGAGGAAAGGAGGAAGGGAAGGAGGGAGAGAAT | 165418366 |
| 1102 | AAAAAAAAAAAAAAAAAAAAAAAAAAAAAAAAAAAAAAAAAAAAAAGGAAAAC | 165819819 |
| 1103 | GAGAGAGAGAGAGAGAGAGAGAGAGAGAGAGAGAGAGAGAGAGAGAGAGAGAGGGGAAGGAT | 165884875 |
| 1104 | GAAGGGAAAAGAAAAGAAGAAAGGAAAGAAGGAAAGAGGGAAAAAAGAAAGAAAGAAAGAAGGAAGAAAGAAAGAAAAGAAGAT | 166226569 |
| 1105 | GGAAAAGAAAGAGAGAGAGGAAGGAAGGAAGGAAGGAAAGAAGGAAGGGAGGGAGGGAGGGAAGGGAAGGGAGGAAAAAGGAGT | 166233901 |
| 1106 | GAAGAAGGAGAAGGAGAAGGAGAGAGAAGGAGAAGGAGAAAGGAGAAGAAGAGGGAAGAGGAGGAAAAAAGAAGAAGGAGAAAGAGGAGAAGGAAAAGC | 166282302 |
| 1107 | GGAAGGAAAAAAAGAAGGAAGGAAGGGAGGGAGGGAGGGAGGAAGGAAGGAAGGGAGAT | 166876712 |
| 1108 | AAAAAAAGAAAGAAAAGAAAAAAAGAAAAAGAAAGAAAGAAAGAAAGAAAGAT | 166902439 |
| 1109 | AAAAAAAAAAAAAAAAAAAAAAGAAAAAGAAAAAGAAAGAAAGAAAGAAAGAGAAAAC | 166983061 |
| 1110 | AGAAAAAGAGAAAGAAAGAGAAAGGAAGGAAGGAAGGAAGAAAGAAAGAAAGAAAGAAAGAAAGAAAGAAAGAAAGAAAGAAAGAAAAAGAAAGAAAGAAAGAAAT | 167398980 |
| 1111 | AGGAAAAAGAGAAAGAAAGAAGGGAAGGGAAGGGGAGGGGAGGGGAGGGGAGAAAGAAAGAAAGGAAAGAAAGAAAGAGAGAGAAAGC | 167459996 |
| 1112 | GAAGAAAGAAGGGAGAGGGAGAGGGGGAGAGGGGGAGAGGGGGAGGGAGAAGGAGAC | 167645151 |
| 1113 | GAAAGAAAGGAAAGGAAAGAAAAGAAAAGAAAAGAAAAGAAAAGAAAAGAAAAGAAAAGAAAAGAAAAGAAAAGAGAC | 167948679 |
| 1114 | GAAAGAAAGGAAAGGAAAGAAAAGAAAAGAAAAGAAAAGAAAAGAAAAGAAAAGAAAAGAAAAGAAAAGAAAAGAGAC | 168105542 |
| 1115 | GGGGAAGGAAGGAGGGAGAAAGAGAGAAAGGGAAGAGAGAGGGAGAGAGAGAGAGAGAGAGGT | 168363505 |
| 1116 | AAAAAAAAAAAAAAAAAAAAGAAAGAAAAGAAAAAGGAAAAAGAAAGAAAGAT | 168790226 |
| 1117 | GGAAGGAGGGAAGGAAGGAGGGAAAGAAGGGAGGGAGGGAGGAGAGAGAGAGAAAGAGAAAGAGAAAGAAAAAAGAAAAGAAAGAAAGAAAGAGAAAGAAAGAAAGAAAAAGAAAAGAAAGAGT | 168814857 |
| 1118 | GAGAAAGAGAAAGAAAGAAAGAGAGAAAGAGAGAAAGAAAGAAGGGAGGGAGGGAAGGAAGGAAGGAAGGAAGGAAGAGAAAGAAAT | 168867350 |
| 1119 | AGAAGGAAGGAAGGAAGGAAGGAAGAAGGGAAGGAAGAGGAAGGAAGGAAGGAAGGAT | 168896896 |
| 1120 | AAAAAGAAGAAAGAAGGAGGAGGAAGAAGGAAGAAGAAGAAAGAAGGAAGAAGAAAGC | 168998300 |
| 1121 | AAAAAAAGAAAGAAAGAAAGAAAGAGAGAGAGAGAGAGGAGGAGGAGAAGGAAGGAAGGAAGGAAGGAGAGAGAGAGGGAAGGAAGGAAGAAAGGAAGAGAGAGGGAGAAAGAAAGAGAGAGAGAAAGGAAGGAAGGAAGGAGAAGAGGAGGAAGGAAGGAAGGAGAGAGGGAAGGAAGGAAGGAAGGAAAAGAGAAAGAAAGAGAGAT | 169019307 |
| 1122 | GAAGGAAGGAAGGAAGGAAAAGAGAAAGAAAGAGAGAGGAAGGAAGGAAAGGAAGGAAGGAGAAGAGGAGGAGGAAGGAAGGAGAAGAGGAGGAGGAAGGAAGGAAAGGAAGGAAGGAGAAGAGGAGGAGGAAGGAAGGAAGGAGAGAGAGAGGGAAGGAAGGAAC | 169019516 |
| 1123 | GAAGGAAGGAAGGAAGGGGGAGGGAGGGAGGGGAGGAAGAGGAGGAGGAGGAAGGAT | 169019682 |
| 1124 | AAGAAAGAAAGAAAGAAAGAAAGAAAGAAAGAAAGAAAGAAAGAAAGAAAGAAAGAAAGAAAGAAAGAAAGAAAGAGAGAGAGAGAGAGAAAGAAAGAAAGAGAAAGAAAGAAAGAAAGAAAGAAAGAAAGAAAGAAAGAAAGAAAGAAAGAAAGAAAGAAAGAAAGAAAGAAAGAAAAGGAAGGAAAGAAAGAAAGAAAGAT | 169023884 |
| 1125 | AAGAAAGAAAGAAAGAAGAAAGAAAGGAAGGAAAGGAAGGAAAGGAAGGAAAGAAAGGAAAGAAAGGGAGAAAAGAAAGAAAGAAAGAAAGAAAGAAGGAAGGAAGGAAGGAAGGAAGGAAGGAAGGAAGGAAGGAAAGAGC | 169345170 |
| 1126 | AAAAAAAAAAAAGAAAGAAAGAAAAAGAAAGAAAGAGAAAAAGAGAGAAAGAAGGAAT | 169582580 |
| 1127 | GAAAGAAAGAAAGAAAGAAAGAAAGAAAGAAAGAAAGAAAGAAAGAAAGAAAGAAAGAGAAGGGAAT | 170559945 |
| 1128 | AAGGAAGGAAGGAAGGAAGGAAAGAAGAGAGAGAGAGAAAGAAAAGAAAGAAGGAGAAGAAAGAGAGAAAGAAAGAAAGAAAAAGAAAAAGAAAAGAGAGAGAAAGAAAGAAAAT | 170560012 |
| 1129 | GGGAAGGGGAAAGGAAGGAAGGAAGGAAGGAAGGAAGGGAGGGAGGGAGGGAGGGAGGGAGGGAGGAAGGAAGGAAGGAAGGAAGAGGAGAAGGAAGAC | 170585676 |
| 1130 | AAAAAAAGGAAGAAGGAAGGAAGGAAGAAAGAAAGGAAAGAAGGAAGGAAGGAGAGAGAC | 170721774 |
| 1131 | AGGAAGGGAAGGGAAAGGAAGGGAAGGGAAGGGAAGGGAAGAGAAGGGAAGAAAT | 170912409 |
| 1132 | AAAAAAAAAGAAAGAAAGAAAGAAAGAAAGAAAGAAAGAAAGAAAAGAGAAAGAAAGGAAGGAAGGGAAAGAAAGAAAGGAAAAAT | 170928417 |
| 1133 | GAAAGAAAGAAAAAGAAAGGAAGGAAGGAAGAAAGAGAAAGAAAGAGAGAGAGAGAGAGAGAAAGAAAGAAAGAAAAAAAGAGAGAGAAAGAAAGAAAC | 170948740 |
| 1134 | GGAGGGAGAGAGGAAAAAGAGAGGGAGGGAGAAAAAGAAAGAAGGAAGGAAGGAAGAC | 171032169 |
| 1135 | GGGAAAGAAAGAAAGAAAAAGAAAAGAAAAGAAAAAGAAAAAAGAAAGGGAGGAAAGGAAAGAAAAAAAAGGAAGGAAGGAAGGT | 171069061 |
| 1136 | AAGAAAAGAAGGAAGGAAGGAAGGAAGGAAGGAAGGAAGGAAGGAAGGAAGGAAGGAAGGAAGGAAGGAAGGAGGGAAGGAGGGAGGGAGGGAGGGAGGGAGGGAGGGAAGGAT | 171169788 |
| 1137 | AAAAAAAAGAAGAAGAAGAAGGAGGAGGAGGAGGAGGAGGAGGAGGAGGAGGAGT | 171299689 |
| 1138 | AGAGAAGAAAGAAAAAGGAAAGGAAAGGAGGGGAGGGGAGGAGAGGGGAAGGGAGGGGAGGAAAT | 171456694 |
| 1139 | GAGGGAAGGAAGGAAGGAAGGAAGGAAGGAAGGAAGGAAGGAAGGAAGGAAGGAAAAAGAAAGAC | 171456883 |
| 1140 | AAAAAAAAAAAAAAAAAGAAAAGAAAAGAAGGAAAGGAAAGAAAAAGAAAGAAAGAAAGAAAGAAAAGGAAAGAAAAAGAAAGAAAGAAAGAAAGAAAGAAAGAAAGAAAGAAAGAAT | 171471223 |
| 1141 | AAGGAAGGAAGGAAGGGAGGGAGGGAGGGGAGGGGAAGGGAGGGAGGGAGGGAGGGGAGGGGAGAAGGAGGGAGGGAGGGGAGGGGAGGGGAGGGAAAT | 171478193 |
| 1142 | GAGGAAAGGAGGAGAAGGAGGAGGAAAAGGAAAGGAAAAGGAGGGGAAGAAGAAGGGGGT | 172231501 |
| 1143 | AAGAGAGAAGAGAGGAAGGAAGAGAAGAAGAAAGGAAGGGAAGGAAGGAAGGGGGGGGAGAGAGAGGGAGGGAGGGAGGC | 172839702 |
| 1144 | AAAAAAAAAAAAAAAAGAAGAAGAAAGAAGGAAAGAAAGAAAGAAAGAGAGAGAGGGAAGGAAGGAAGGAAAGAAGAAAGAAAAAAAAAGAAAGAAGGAAGAAAGAAAGAGC | 173076337 |
| 1145 | AAAGGAAGAAAGAAAGAAGGAAAGAAAGAAGGAAGGAAGGAAGGAAAGAAAGAAGGAAAGAGGGAGAAAGAAAGAGAGGAGAAAGAGAGAGGGAGAAAGAAAGAAAAAGAAAGAAGGAAGGAAGGAAGGAAAGAAAGAAAGAAAGAAAGAGAAAGAAGGAAGGAAGGAGAAAAGAAAAGAAAAT | 173243282 |
| 1146 | GAAAGAGAGAGAGAGAGAGAGAGAGAGAGAGAGAGAGAGAAGGAAGGGAAGGGAAGAGGAGGGGAGGGGAGGGGAGGGGAGC | 173545788 |
| 1147 | GGAGAGGGAAGAGGAGGAGGAGAGAGAAGAGGAGGGGGAGGGGAGGGGAGAGGAAGGGAGGGGAGGGGAGAGGAAGGGAGGGGAGGGGAGAGGAAGGGAGAGGAAGGGAGGGGAAGGGAGGGGAAGGGAGGGGAAGGGAGAGGAAGGGAGAGGAAGGAGAAGGGGAAGGGAAGGGAAAAGGGAGAAGGGAGAAGGAGGGT | 173603726 |
| 1148 | GAAAGAAAGAAAAGAAAGAGAGAGAGGGAGGAAGGAAAGAAGGAAAGAAGGAAAGAAGGAAGGAAGGAAAGAAGGAAGGAAGGAAGGAAGGAAGGAAGGAAGGAAGGAAGGAAAAGT | 173607168 |
| 1149 | GAGAGAGAGAGAGGGAAAGAGGGAGAGGGAGAGAGAGAGAGGAGGGAGGAGGGAGGGAGGT | 173775910 |
| 1150 | AGAGAAAGAGAGAAGGAAGGAGAGAGAGAAAGAAAAAGAAAGAAGGAAAGAAAGT | 174371050 |
| 1151 | GAAGGAAAGAAAGGAAGAAAGAAAGAGAAGGAAGGAAGGAAAGGAAAGAAGGAAGGAAGGAAGGGAAAGAAGGAAGGAAGGAAAAAAT | 174371105 |
| 1152 | AAAAAAAAAAGAAGAAGAAGAAGAGGAAGAGGAGGGGGAGGGGGGAGGAGGAGGAAAGAAGAAGAAGAGGAGGAGGGGGAGGGGGAGGAGGAAGAAAGAAGAAGAGGAAGAGGAGAGGGAGGGGGAGGAGGAGGAAAGAAGAAGAAGAGGAGGAGGGGGAGGGGGAGGAGGAGGAAAGAAGAAGAAGAAAGAAAAGGGGGC | 174736956 |
| 1153 | AAAAAAGAAAAAAGAAAAAGAAAAAGAAAAAAGAAAAAGAAAAGGAAAAGAAAAAAGAGGT | 174827210 |
| 1154 | GAAGGAGAGGAGGGGAGAGGGAGGGAGGGGGAGAGAGAGAGAGAGAGAGAGAGAGAGAGAGAGAGAGAGAGAGAGAGAGAGAGAT | 174860998 |
| 1155 | AAAAAAAGAAAGAAAGAAAGAAAGAAAGAAAGAAAGAAAGAAAGAAAGAAAGAAAGAAAGAAAGAAAGAAAT | 175411923 |
| 1156 | GAGGAAGAAAGAAAGAGAGAGAGAGAGAAGGAAGGAAGGAAGGAGAGAGAAAGAAAGAGGGAGAGAGAAAAAAGGAAAGAGAAGAAAGAAAGAAAGAAAAAAGAAAGAAAGAAAAAAGAAAGAAAGAAAGAAAGAAAGAGAAAGAAAGAAAGAAAAGAAAAAAAGAAAAGAAAAGAAAAAGAGGT | 175475221 |
| 1157 | GAGAGAGAGAGAGAGAGAGAGAGAGAGAGAGAGAGAGAGAGAGAGAAAGAGAGAGAGAC | 175511928 |
| 1158 | AGAAGGAAAGAAAGAAAGAAAGAAAGAAAGAAAGAAAGAAAGAAAGAAAGAAAC | 175542475 |
| 1159 | AAAGAAAGAAAGAAAGAGAAAGAAAGAAAGGAAGAAGAAAGAAAGAAAGAAAGAGAGAGAGAAAGAAAGAAAAAAGAAAGAGAAAAGAAAGAAAAGGGT | 175542537 |
| 1160 | AAGAAGAAGAGGAGGAGGAGGAGGAGGGAGAAGGAGAAGGAGAAGGAGGAGGAGGAGGAGAAGGAGAAGGAGAAGGAGAAGAAGAAGAAGAAGAAGAAGAAGAAGAAGAAGAAGAAGAAGAAGAAGAAGAAGAAGAAGAAGAAAC | 175767945 |
| 1161 | AGGAAAAGGAGGAAGAGGAGGAGGAGGAGGAGGAGGAGGAGAGGAAAGAAAAT | 175768189 |
| 1162 | AGAAAGAAAGAAAGAGAGAAAGAAAGAAAAAGGAAGGAAGGAAGGAAGGAGAGAAAGAAAGAAAGGAGAGAAAGAAAGAAGGAAAGAAAGAAAGAAGGAAGGAAAGAAAGAAAAAGAAAGAAAGAAAGAAAGAAAGAAAGAAAGAAAGAAAGAAAGAAAGAAAGAAAGAAAGAAAGAAAC | 176303020 |
| 1163 | AGGAAGGGAGGGAAGAAGGAAGGGAGGGAGGGAGGGAGGGAGGGGAAAAAAGC | 176320261 |
| 1164 | AAAGAAGAGAAGAGAAAAAGGAGAGGAGAGAAAGAGGGAGAGGAGAGAAAGAGGGAGAGGGAGAAGAGAGGGAAAGAGGAAGGGAGGGAGGGAGGGAAGGGAGGAAGGAAGC | 176346865 |
| 1165 | AGAAGAAAGGAAGGAAGGAAAGAAGGAAGGAAGGGAGGGAGGGAGGGAGGGAC | 177091607 |
| 1166 | AGAGAGGAAGAGGGAGAGGGAGAGGAAGAGAGAGAGAGAGGGAGAGGAAGAGAGAGAGGGAGAC | 177160214 |
| 1167 | AAGAGAGGGAGGAAGAGAGAGAAAGAAGAAGGAGGAGGAAGAGAAGAAGAAGGAGGAGGAAGAGAAGAAGAAGGAGGAGGAGGAAGAGGAGAAGGAGGAT | 177160337 |
| 1168 | AAAAAAAAAAAAAAAAAGAAAGAAAGAAAAAGAAAAAGAAAAGAAAGAAAGAAAAAGAAAT | 177228057 |
| 1169 | GGGAAGGAGGGAAGAGAGAAAGGGGGAAAAGGAGGAAGGAAGGAAGAAAGGAAGGAAGGGAAGAGAAGAT | 177510582 |
| 1170 | AGGAAGGAAGGAAGGAAGGAAAGAAGGAAAGAAGGAAGGGAGGAAGGAAGGAAAGAAAGAAGGAAGGAAGGAAGGAAC | 177713191 |
| 1171 | AAGAGGGAGGGAAGGGAAAAGAAGGGGAGGGGAGGGGAGGGGAGGGGAGGGGAGGGAAAGAAAAAGT | 178061522 |
| 1172 | AGAAGAAGAAGAAGAAGAAGAAGGAGGAGGAGGAGGAGGAGGAGGAGGAGGAGGAC | 178064161 |
| 1173 | AGAGGAAGGAAAAAAGAAAGGAGGGAAGGAAGGAAGGAGGAAGGAAGGAAAGAAGGAAGGAAGGAAGAAAAGGAGGAT | 178102626 |
| 1174 | AAAGGAGGGAGGGAGGGAAGGAGGGAAGGAAAGAAGGAAGGAAGGGAGGGAGGGAGGGGAGGGGAGGGAGGAC | 178102706 |
| 1175 | AGAGAGAAAGGAAGGGGAGGGGAGGGGAGGGAAGGAAGGGAAGAAAGGAAGAGAGAAGAGAGAGAGAAAT | 178531167 |
| 1176 | AAAAAAAAGAAAAAAGAAAAAGAAAAAAAGAAAGAAAGAGAAAGAAAGAAAAGT | 178617580 |
| 1177 | GAAAAGGAAGGAAGAGAAGGGAAGGAAAGAAGGAAGGAAGGAAGGGAGGGAGGGAGGAAGGAAAGAGAGGGAGGAAC | 178647433 |
| 1178 | AAGAAAGAGAGAGAGAAGGAAGGAAGGAAGGAAGGAAGGAAGGGAAAGAAAGAAGGAAAGGGAAAGGAAAGAAAAGGAAAAGAAAAGAAAGAAAGAAAAAGAAAGGAAGGAAGGGAGAGAGAGAGAGGGAGGGAAGAAGGGAGGAAGGGAGAAAAGAGAC | 178770600 |
| 1179 | AGAGGGAGGGAGGGAAGGGAGGAAGGAAGGAAGGAAGGAAGGAAAGAGAGAC | 178798358 |
| 1180 | GAAAGAAAGAAAGAAAAGAAAGAAAGAAAGAAAGAAAGAAAGAAAGAAAGAAAGAAAGAAAGAAAGAAAGAAAGAAAAGAAAAGC | 178864212 |
| 1181 | GAAAGAAAGAAGAAAAGAAAGAAAGAGAAAGAAAGAAAGAAAGAAAGAAAGAAAGAAAGAAAGAAAGAAAGAAAGAAAGGAAGAAAGAGAGGGAAAGGGGAGGGGGGGAAGGAGAGGGGAT | 178864430 |
| 1182 | GGAAGGGGGAAAGGGAGGGAGGGAAGGAAAGAAGGAGGAAAAAGAAAGAGAAAGAAAGAGAGAGAAAGAGAAAAAAAGAGAAAGAAAAAGAAAGAGAGAAAGAAAAAAGGAC | 178864593 |
| 1183 | AGGAAGGAAGGAAGGAAGAAGGAAGGAAGGAAGGAAGGAAGGAAGAAGGAAGGAAGGAAGGAAGGAAGGGAAAGAAAGAAAGAAAGAGAAAGAGGGGGGAGGAAGGGAAGGAGGGAGGGAGAGAGAGAGAAGGAAGGGAGGAAGGAAGGGAGGGAGGAGGGAAGGGAGGGAGGGAGGGAGAAAGGAAAT | 178864885 |
| 1184 | AGGGAAGAAGAAAAAAGGAGAGAAAGGAGAAAAAAGAGAGGAGAGGGAGGGAGGAAAGAAGGAGGGAAAAAGAGGAAGAAGAAAGGGGGAAAAAC | 179083339 |
| 1185 | AAAGAAAGAAAGAAAGAAAAAGGAAAGAGAGAGAGGGAGGAAGGAAGGAAGGAAGGT | 179399819 |
| 1186 | AGGAAGGAAGGAAGGAAGGAAAGAAAGGGAGGGAAGAAAGGAAGAGAGAGAGGGGAGGAAAGAAGGAAGGAAGAGAGAGAGAAGGGAAGGAAGGAAGGAAGGAAAAGAAAAGAGT | 179399876 |
| 1187 | AGAAAGAAAGAAAGAAAGAAAGAAAGAAAGAAAGAAAGAAAGAAAGAAAGAAAGAT | 179439105 |
| 1188 | AAAAAAAAAAGGAAGAAAGGAAGGAAGGGAGGGAGGGAGGGAGGGGAGGGAGGGGAGGGAGGAGAGGGAT | 180191025 |
| 1189 | AGAGAGAAGGAGGAAGAAGAGGAGGAGGAGGGGGGGAGGAAGAGGAGGAGGAGGGGGGGAC | 180666335 |
| 1190 | GAAAGAAAGAAAGAAAGAAAGAAAGAAAGAAAGAAAGAAAGAAAGAAAGAAAGAAAGAAAGGAAGGAGGAAAGAAGGAAAGAAGGAAAGAAAAGAAGT | 180691952 |
| 1191 | GAGAGAGAGAGGGAGAGGAGGAGGGGGAGAGGGAGAAGAAGAGGGGAGGAGGGAGGAGGGAGAAAAAGGGGGAGAGAAGAAGAGGGGAGGGGGAGGAGGGGGAGAGGGAGAGGGAGAGGAAGAAT | 180694853 |
| 1192 | AGAAAGGAAAGAAAAGAGGGGGAGGGGGAGAGGGAGAGGGAGAGGGAAAAAAAGGAAGAT | 180931461 |
| 1193 | GAAGGAAGAGAGGGAGGGAGGGAGAAAGGAAAGGAAGGAAAGGAAAGGAAAGGAAGGAAGGAGGGAGGGAGGGAGGGGGGAGGAAGGGAGGGAAAGAAAT | 181048769 |
| 1194 | AAAAGAAAAGAAAAGAGAAAAAAGAAAAGAAAGAAAGAAAGAGAAGAAAAGAAC | 182126334 |
| 1195 | AGGAAAAGAGAGAGGGGAGAGAGAGAGAGAGAGAAAGAGAGAGAGAGAGAAAAAAGAAGAAGGAGGAGGAGGGT | 182225851 |
| 1196 | GGAAAGAGAGAAAGAAAAGAAAGAAAGAAAAGAAAAAAGAAAGAAAGAAGGAAGAC | 182275808 |
| 1197 | AAAAAAAAAAAAAAAAAAAAAAGAAAAGAAAAGAAAAGAAAAGAAAAGAAAAT | 182336753 |
| 1198 | AAAAAAGAAAAAGAAAGAGAGAAAGAGAGAAAGAAAGGAAGGAAGGAAGGAAGGAGAGAGAGAAAGAAAGAAGGAAGGAAGGAAGGAGAGAGAGAGC | 182451563 |
| 1199 | AGGAAGGAAGGGAGAGAGAAAGAGAGAAAGAGAAAGGAGAGGAGAGGGGAGAGGAAGGGAGGGGAGGGGAGGGGAGGGGAGAGGAGAGGAGAGT | 182451681 |
| 1200 | AGAGGAAGAGGAGGAAGAAGAGGAAGAAGAAGAGGAAGAGGAAGAAGAAAAGAAGAAGAAAGAAGAAGAAGAAAAGAAGAAGAAGAAGAAAAGAAGAAGAAAAGAAGAAGAAAAGAAGAAGAAAAGAAGAAGAAGAAGAAGAAGAAGAAGAAGAAGAAGAAGAAGAAGAAGAAGAAGAAGAAGAAGAAGAAGAAGAGGAAGAAGAAGAAGAGGAAAAGAAAGGAGAAGGAGAAGGAGAAGAAGGAGAAGGAGAAGAAGAAAAAGAAGAC | 182468212 |
| 1201 | AAAAAAGAAAGAAAAAAGAAAGAAAGAGAGAAAAAGAGAGAGAGAGAAAGAGAC | 182486758 |
| 1202 | AAAAAAAAAAGAAAGAAAAGAAAGAGGGAAAGGAAGGAAGGAAGAAGAAAGAAAAGAAAGAAAGAAAGAAAGAAAGAAAGAAAGAAAGAAAGAAAGAAAGAAAGAAAGAAAGAGAAAT | 182506974 |
| 1203 | AAAAAGAAAGAAAGAGAGAGAGAGAGAAAGGGAGGAAGGGAGGGAGGGAGGAAGGAAGAAAGT | 182518636 |
| 1204 | AAAAAGAAAAAGAAGAAAGAAAGAGAGAAAAGAAAAGAAAGAAAAGAAAAGAAGAAAGAAAGAAAGAAAGAAAAGAAAAGAAGAAAGAAAAAAAGAAAGAAAGAAGAAAGAAAGGAAGGAAGGAAGAAAGAAAGAAAGAAAGAAC | 182745938 |
| 1205 | GAAAGAAAGAAAGAAAGAAAGAAAGAAAGAAAGAAAGAAAAAAGAAAGAAAGAAAGAAAGAT | 182746083 |
| 1206 | AAAAAAAGAAAGAAAGAAAGAAGGAGGAAAGAAAGAAAGAAGGAAGGAAGGAAAGAAAGAGAGAGAGAGAGAAAGGGAGGGAGGGAGGAAGGAAGGAAAT | 182898427 |
| 1207 | AAAAGAAAGGAAAGGAAAGGAAAGGAAAGGAAGAAAGAAAGAAAGAAAGAAAGAAAGAAAGAAAGAAAGAAAGAAAGAAAGAAAGAAAGAAAGAAAGAAAGAAAGAAAGAAAGAAAGAGAGAAAGAAAGGAAGGAAGGAAGGAGGGAAGAGAGGGGT | 183076449 |
| 1208 | GGGGGAAGGAGGGAGGAAGGGAGGGAGGAAGGAAGGAAGGAAAGAAGGAAGGAAGGGAAAGAC | 183076606 |
| 1209 | AAAGAGAGAGAGAGAAAGAAAGGAAAGGAAGGAAGGAAGGAAGGAAGAAAGAAAGAAAGAAAGAAAGAAAGAAAGAAAGAAAGAAAAAAAAAGGAAGAAGGAAAGGAAGGAAGAAAGGAAGGAAGGGGAGGAAGGGGAGGAAGGGAAGGAAGGGAAGAAAGGGAAGGAAGGGAAGGAAGGGAAGGAAAGGAAGGAAAGGAAGGAAAGAAGT | 183076669 |
| 1210 | AAAAAAGAAAGAAAGAAAGAAAGAAAGAAAGAAAGAAAGAAAGAAAGAAAGAAAGAAAGAAAGAAAGAAAGAAAGAAAGAAAGAAAGAAAGAAAGAAAGAAAGAAAGAAAGGAAAAAAGAGAAAGAAAGAAAGAAAGAAGGGAGGGAGGGAGGGAGGGAGGGAGGGAAGGAAGGAAGGAAGGAAAGAAAGAAAAT | 183541511 |
| 1211 | GAAAGGAAGAGAAAGAAAAGAAAGGAAGGAAGGAAGGAAGGAAGGAAGGAAGGAAGGAAGGAAGGAAGGGGAAGGGGAAAGGGAAGGGAAAGGAAAAGGGAAAGGGAAGAAAAAGGAAAGGGAAGGAAAAAAGAAAAGGAAAAGAAAGAGGAAGGAC | 183589910 |
| 1212 | AAAGAAAAGAAAAGAGAGGGGAGGGGAGGGAAGAGGGAAGGGGAAGGGAGGGAAGGGGAGGGGAGGAGAGGGC | 183615996 |
| 1213 | AAAAAAAAAAAAAAAAAAAAAGAAAGAAAGAAAGAAAAAGAAAAAGAAAAGAAAGAAC | 183618209 |
| 1214 | AGAGGAGGAAGAAGAAAAAGGAAAGAGGAAGGGAGAGAGGGAGAGGAAAAAT | 184252097 |
| 1215 | GAAAGAAAGAAGGAAAGAGAGAAAGAGAGAGAGAGAGAGAGAGAGAAAGAAGAGAAGAGAAGC | 184286429 |
| 1216 | GGGGAGGAGGAAGAGGAAAAGGAGAAGGAAGGAAGGAAGAGAAAGAAAGAAGAAAAGAAT | 184397077 |
| 1217 | AGAAAGAAAAAAAGAAGGGAAGAAAGGAAAGAAAGAAGGGGAGGGAGGGAGGAAGT | 184406564 |
| 1218 | GGGGGAAGGAGGGAAGGAAGGAAGGAAGGAAGGAAGGAAGGAAGGAAGGAAGGAGGGGAGGGGAGGGGAGGGAGGGAGGGAGGGAGGAAAGGAAGGAAGGGAAGAAAGGGAGGGAAGGGAAGAAAGGGAAGGAAGGGAAGGAAGGAAGAAAGAAAGAGAGAAAAGAAAGT | 184421052 |
| 1219 | AAGAGAAAGAAAGAAAGAAAAGAAAAGAAAGAAAGAAAGAAAGAAAGAAAGAGAGAGAAAGGAAGGAAGGAAGGAAAAGAAAGAT | 184448122 |
| 1220 | AGGAAGGAAGGAAGGAAGGAAGGAGGGAGGGAGGGAAAGAAAAGAAAAGAAAAGAAAGAAGGAAGGAAGGAAAGAGAAAGAAGAAAGGAAAGGAAAGGAAAGGGAAGAAAGGAAAGGAAAGGAAAGGGAAGAAAGGAAAGGAAAGGAAAGGAAAGGAAAGGAAAGGAAAGGAAAGGAAAGGAGGAGAC | 184660317 |
| 1221 | AGGAAGGGGGGAAGAGGAGGGAGGAAGGGAGGAAGGGAGGGAGGAAGGGAAAGAAGGAGAGAAGGAAGGAAGGAGGGAAGGAAGGAGGGAAGGAAGGAAGGAAAGGAGAAT | 185170149 |
| 1222 | GAAGGAAGGAAAGAGAAAAGGAGAAGGAAAAGGAAGGAAGGAAGGAGGAAGGGAGGGAGGGAAGGAGT | 185170260 |
| 1223 | GAGGGAAGGAAAGAAGGAAGGAAGGGGAAAAGGAGAGAAGGAGAAGGGGAGGGAGGAAGGGAGGAAAGAAGAAAGGAGGGAGGGAGGAAAGAAGGAAAGAAGAAGGGAGGGAAGAAGAGAGAGAAGGAGGGAGGGAAGT | 185170328 |
| 1224 | AAAAAAAAGAAAGAAAGGAAAGAAGAAAGAAAGAAAGAGAAAGGAAGGAAGAAAGAAGGAAGAAGGAAGGAAGGAAGGAAGGAGGGAAGT | 185307321 |
| 1225 | AAAAAAAAAAAAAAAAAAGAAAGAAAGAAAAGAAAAGAAAAAAGAAGAAAAAAT | 185876788 |
| 1226 | GGAGAGAGAAGGAAGGAAGGAAGGAAGGAAGGAAGGAAGGAAGGAAGGAAGGAAGGAAGGAAGGAAGGAAGGGAGT | 186372080 |
| 1227 | GAGAAGGGAGGAAGGGAGGGAGGGAGGGAGGGGAGGAGGAGGGGAGGGAGAAGGGAAGGAGGAAGGGAGGGAGGGAGGGGAGGT | 186501849 |
| 1228 | GGAAGAGAAAGAAGAGGAAGAAGAAGAAGAAGAAGAAGAAGAAGAAGGAGAAGGAGAAGGAGAAGGAGAAGGAGAAGGAGAAGAAGAAGAAGAAGAAGAAGAAGAAGAAGAAGAAGAAGAAGAAGAAGAAGAAGAAAT | 186940198 |
| 1229 | AAAGAAAAGAAAGAAAGAAAGAAGGAAAGAAAGAAAGAAAGAGAGAAAGAAAGAAAAAAGAGAGAC | 186963375 |
| 1230 | AGAAAGAAAGAAAGGAAAGAAAGAAAGAAGAAAGAAAGAAGAAAAAGAAGGAAAAAGAAAGAAAGAGAAAGAAGGAAGGAAGGAAGGAAAGAAGAAAGAAAGAGAAAGAAAGAAAGAGAAAGAAAGAAAAGAAAGAAAGAAAGAAAAAAGAAAAAGAAAGAAGAAAGAAAGAAAGGC | 187257882 |
| 1231 | AAAAAAAAAAAAAAAAAAAAAAGAAAGAAAGAAAAAGAAAAAAGGAAAGAGGAAGC | 187361962 |
| 1232 | AAAAAAAAAAAAAAAAAAGAGAGAGAGAGAGAGAAAAGAAAGGAAAAAAAAAGT | 187557539 |
| 1233 | AGAGAGAGAAAGAGAAAGAAAGAAAGAGAAAGAAAGAAAGAAAGAAAGAAAGAAAGAAAGAAAGAGAAT | 187942915 |
| 1234 | AAGAAGAAGAAGAAAAAAAAAGGAAGAGGAAGAGGAGGAGAAAAAGGAGAAGAGGT | 187976421 |
| 1235 | AAAAAAAAAAAAAAAAAAAGAAAAAAGAAAGAAAGAAAAAAAGAAAGAAAAAT | 188271713 |
| 1236 | AAAGGGGAAGAGGAGAGGAGAGAAGAGGAGAGGAAAGGAGAGGGGAGGGGAGGGGAGAAGGGGAGGGGAT | 188331553 |
| 1237 | AGGAGAGGAGAAGGGGAGGGGAGGGGAGAAGGGGAGGGGAGAGGAGGGGAGGGGAAGGAT | 188331623 |
| 1238 | AAGAAAGAAAGAAAGAGAGAGAGAGAGAGGAAGAGAAGAGAAGAGAAGGAAGAGAAGAGAAGAGAAGAAAAGAGAAGAGAAGAGGAAAGAAAGAAAAGAAAAAC | 188507764 |
| 1239 | AAGAGAAGGGAAGGGAAGGGAAGGGGAAGGGGAAGGGAAAAGAGAAGAGAAGAGAAGGGAAGGGGAAGGGGAAGGGAAAAGAGAAGAGAAGGGAAGAGAAGGGAAGAGAAGAGAAGAGAAGAGAGAGAAGAGAAGAAAGAAAGAGAAAGAAAGAAAGAAAAGGAAAGAAAGAAAAAGAAAGAAAGAAAGAAAGAAAGAAAGAAAGAAAGAAAGAAAGAAAGAAAGAAAGAAAGAAAGAAAGAAAGAAAAT | 189005800 |
| 1240 | GAGAGAGGGAAGAAAGGAAGAAAGAAAGGAAGGAAGGGAGGGAGGGAGGGAGGGAGGGAGGAAGGAAAGAAAAGAAAGAAAGAAAGAAAGAAAGAAAGAAAGAAAGAAAGAAAGAAAGAAAGAAAGAAAGAAAAGGAAAGAAAAGAGGGAGGAAAAGAT | 189097899 |
| 1241 | AAAAAAAGAAAGAAAAGGAAAAGAGAGAAGAGAAGAGAAGAAAAAGAAAAGAGAGAAGAGAC | 189209343 |
| 1242 | AGAGAGGAAGGAAGGAAGGAAGGGAGGGAGGGAGGGAAGGAGGGAAAGAAAGGC | 189212763 |
| 1243 | AAAAGAAGGAAGGAAAGAAGGAAGGAAGGAAGGAAGGAAGGAAGGGAGGGAGGGAGGGAGGGAGGGAGGGAGGGAGGGAGGGAGGGAAAGAGGGAGGGAGGGAC | 189286949 |
| 1244 | GGAGGGAAGGAAGGAAGGAAGGAAGGAAGGGAGGGAGGGAGGGAGGGAAAGAGGGAGGGAGGGAC | 189287053 |
| 1245 | GGAAGAGGGAAGGAAAGAAGGAAAGAAAGAAGGAAAGAAGGAAGGAAGGAGAAAAGGT | 189409537 |
| 1246 | AAGAAGAGAGAAAGAAAAAGAAAAAGAGAAAAGAAAAAGAAAAGGAAAAAGGAAAGGAAAGGAAAAAGGAAAAGGAGGGGT | 189858397 |
| 1247 | AAAAAAAAAAGAAAGGAGAGAAAAGGAAGGAAGGAAGGAAGGAAGGAAGGAAGGAAGGAAGGAAGGAAGGAAGGAAGGAAAAAGAAAAGAAAGAAAGGGAAGGAAGGAAGGGAGGAAGGAAAGAAAGGT | 190107382 |
| 1248 | AGAAGAGAAAAGAAGGAAGGAGGGAAGGAAGGAGGGAAGGAGAAAAGGAAGAAGGAAGGAAGGAAGGGAGGGAGGAAGGAGGAAGGAAGAAAGGAGGAGAGGGAGGGAGGGAGT | 190354858 |
| 1249 | AGGAAGGAAGGAAGGAAGGAAAGGAGGGAGGGAGAGAGAGAGGGAGGGAGGGAC | 190354995 |
| 1250 | AAAAAAAAAAAAAAAAGAAAAAGAAAAAAGAAAAAAAAAAGAAAAAAGAAAAAGAAAAAAGT | 190383580 |
| 1251 | AAAAAAAAAAAAAAAAAAAAAAAAGGAAAAAAAAAAAAGAAAGAAAGAAAAGAAAAGAAGAC | 190635962 |
| 1252 | AAAGGAGAAAGAAAAGAAGGAGAAGAAAAGGGAGAGAGAAAGAAAGAGAAAGAC | 190809107 |
| 1253 | GAAGAAAAAGAAAGAAAAGAAAGAGAGAAGGAAGGAAGGAAGGAAGGAAGGAAGGAAGGAAGGAAGGAAGGAAGGAAGC | 191132052 |
| 1254 | AAAAAAAAAAAAAAAAAGAAAAAGAAAAAGGAAGGAAGGAGGGAAGGAAGGAAGGAAAAGAAAAAGGGGAAAT | 191155836 |
| 1255 | AGGGAGAGGGAGAGGAAGAAGAAGAAGAAGAGGAAGAGGAAGAGGAAGAGGAAGAGGAAGGAAGGGAAAGGAAGGGAAGT | 191469235 |
| 1256 | AAAAAAAAAAAAAAAAAAAAAAAAAAAAAAAAGAAAAGAAAGAAAAAGAAAAGC | 191642976 |
| 1257 | AAAAAAAAAAAAAAAAAAAAAAAAAAGAAAAAGAAAAAGAAAAAGAAAAAAGAAAC | 191744469 |
| 1258 | GAGAAGAAAGAAAGAAGAAAGAAAGAAAGAAAGGAAGAAAGAAAGAAAGAAAC | 191852261 |
| 1259 | AAAGAAAGAAAGAAAGAAAGAAAGAAAGAAAGAAAGAGAAAGAAAGAAGAAAGAAAGAAAC | 191852314 |
| 1260 | AGAAAGAAAAGAAAGAAAGGAAGAAAGGAAGGAAGGAGAAAGAAGGAAGAGAAGAAAAGAAAGAAC | 191852375 |
| 1261 | GAGAAGAAAGAAAGAAGAAAGAAAGAAAGAAAGGAAGAAAGAAAGAAAGAAAC | 192009123 |
| 1262 | AAAGAAAGAAAGAAAGAAAGAAAGAAAGAAAGAAAGAGAAAGAAAGAAGAAAGAAAGAAAC | 192009176 |
| 1263 | AGAAAGAAAAGAAAGAAAGGAAGAAAGGAAGGAAGGAGAAAGAAGGAAGAGAAGAAAAGAAAGAAC | 192009237 |
| 1264 | GAAGAAAGAAAAAAAAAGAAGGAAGGAAGGGAAGGAAGGAGAGAAAGAGAGAGAGAGAGAAGAT | 192466238 |
| 1265 | AAGAAGGAAGGAAGGAAGGAAGGAAGGAAGGAAGGAAGGAAGGAAGGAGGGAAGGAAGGAAGGAAGAAAGGAAGGAAGGAAGGAAGGAAGAT | 192473956 |
| 1266 | AAGAAAAAAAAAAGAAGGAGAAGGAGAAGGGGAAGGGGAAGGGGAAGAAGAGGAAGAGGAAGAAGAAGAAGAAGAAGAGGAAGAAGAAGAAGAGGAAGAAGAAAGGAGAAGGAGGAGGAGGAGGAGAAGGAGAAGGAGGAGGAGAAGAAGGAGAAGGAGGAGGAGAAGAAGGAGAAGGAGAAGGAGAAGGAGAAGGGGAAGGGGAAGGAGAAGAAGAAGAAGAAGAGGAAGAGGAAGAGGAAGAGGAAGAAGAAGAAGAAGAAGAAGAAGAAGAAGAAGAAGAAGAAGAAGAAGAAGAAGAAGAAGAAGAAGAAAAAGAAGAAGAAGAC | 192513993 |
| 1267 | GAAAAAAAAAAAAAAAAGAAAGAAAGAGAGAGAGAGAAAGAGAGAAAGAAAAGAAAGAAAGAAAGAGAAAGAAAGAAAAAGAAAGAAAGAAAGAGAAAGAAAGAAAGAAAC | 192580496 |
| 1268 | GGGAAGGAAGGAAGGAAGGAAGGAAGGAAGGAAGGAAGGAAGGAAGGAAGGAAGGAAAGAAGGAAAGAAGGAAAGAAGGAAAGAAGGAAGGAAGGGC | 192978911 |
| 1269 | AAAAAAAGAAAGAAAGAAAGAAAGAAAGAAAGAAAGAAAGAAAGAAAGAAAGAAAGAAAGAAAGAAAGAAAGAAAGAAAGAAAAAGAAAGAAAGAAAGAGAGAGAGGAAGGGAGAGAGGAAGGGAGGGAGGGAGGGAAAGAC | 193022070 |
| 1270 | GAGAGAAAGAGAGAGGAGAGAGGGAGGGAGGAGGAGGGAGGGGGAAAGAGAGAAAGAAAGAAAGAC | 193071273 |
| 1271 | AGAAAGAAAGAAAGAAAGAAAGAAAGAAAGAAAGAAAGAAAGAAAGAGGGAGGGAAGGAAGGAAGGAAAT | 193071379 |
| 1272 | AGGAAGGAAAGAGAGAAAGAAAGAGAGAGAGAGAAAGAAAGAAAGAAAGAAAAAAGAAGGAGGGAGGAAGGAAGAAGGGAAGGAAGGAAGGAAGGGGGAAGGAAGGAAGGAGGGAAGGAGGGAAGGAAGGAAGGAGGGAAGGAGGGAAGGAAGGAGGGAAGGAGGGAAGGAAGGAAGGAGGGAAGGAAGGAAGGAGGGAAGGAAGGAAGGAAGGAAGGAGC | 193071510 |
| 1273 | GGAAGGAAGGAAAGAAAGAAAGAGGAAAGAAAGAGAGAGAAAGAAAGAAAAGAAAGAAAAAAAAGAAAAAAGGAAAGAAAGAAAGAAAGAGAGAAAGAAAGAAAGAAAGAAAGAAAAGAAAGAAAAGAAAGAAAGAAAGAAAGAAAGAAAGAAAGAAAGAAAGAAAGAAAGAAAGAAAAGAAAGAAAGGAGGGAGGGAGGAAGGAAGGAGAGAAAGAAAGAGAGAGAGAGGGGAAAAGGGAGGGAGGAAGGGAAAGAGGAAAGC | 193106629 |
| 1274 | GAAAGAAAGAAAGGAAGGAAGGAAGGAAGAAAAGAAAGGAGAAAAGAAAAGAAGGAAAGAGAGAGT | 193288641 |
| 1275 | GAGAAAGAAGAAAAGGAAAGAAAGAGGAAGGAAGGAAGGGAGGGAGGGAAGGAGGGAGGGAGGGAGGGAGGGAGGAAGGGAGGGAGGGAC | 193296389 |
| 1276 | AGAGAGAGAGAGAGAGAGAGAGAGAGAGAGAGAGAGAGAGAGAGAGAGAAAGT | 193473346 |
| 1277 | AAAAAAAAAAAAAAAAAAAAAAAAAAAGGGGAAGAAGAAGAAAGAAGGGAAGGAT | 193475828 |
| 1278 | AAAAAGAAAGAAAGAAAGAAAGAGAGAGAGAGAGAGGGAGAGAGAAAGAAAGAAGGAAAGAAAGAGGAAGAAAGAAAGAAAGAGAAAGAAAGAAGGAAAGAAGGAAAGAAAGGAAAGAAAGAAAGAAGGAAAGAAAAAGAAAGAAAGAAAGAC | 193582836 |
| 1279 | AAAAGAAAGAAAGAAAGAAAGAAGAAAGAGAAAGAAAGAAAAGAAAGAAAGAAAGAAAGGAAAGAAAGAAAGAGAC | 193847119 |
| 1280 | AGAAAGAAAGAAAGAAAGAAAGAAAGAAAGAAAGAAAGAAAGAAAGAAAGAAAAAAAGAAAGC | 193847203 |
| 1281 | GAAGGAAAGGAAAGGAAAAGAGAGGAGAGGAGGAGAGGAGAGGGGAGAAGAGAAGAGAGGAGAGAGGAGGGGAGAT | 194796694 |
| 1282 | AAAGAAAAGAAAAGAGAGGAGAGGGGAGGGGAGGGGAGGAGAGGGGAGGGGAGGGGAGGGGAGGAGAGGAGAGGGGAGGGAAGGGGAGGGGAGGGGAGGGGGAGAGGAAAGAGC | 194836060 |
| 1283 | AAAGAGAAAGAAAGAAAGAAAGAAAGAAAAAGAAAGAAAGAAAGAAAGAAAGAAAGAAAGAAAGAAAGAAAGAAAGAAAGC | 194837433 |
| 1284 | GAAAAAAAAAAAAAAGAAGAAGAAGAAGAAGAAAAGAAAGAAAAAGAAAGAAGAT | 194856998 |
| 1285 | AAGAAAGAAGAAAGGAAGGAAGGAGGGAAGGAGGGAAGGAGGGAAAGAGGGAAGGAGGGAAGGGGAAGGAAGT | 195095384 |
| 1286 | GGGGGAAAAAAAAGAGAAAGAGAAAGAGAGGAAAAGAAGGAAGAAAGGAAAAAAAGGAAGGAAGGAAAGGAGGAAGGAAAGGT | 195221494 |
| 1287 | GGGAAGAAGGGGGAAAGAGGGAGAGGAGAGAGAGAGAGAAAGAGAGAGAGAGAAGT | 195234611 |
| 1288 | AGAAAGAAAGAAGGAAGGAAAGAAAGAAGGAAAGAAGGAAAGAAAGGAAGGAAGGAAC | 195608467 |
| 1289 | GAAGGAAGGAAGGAAGGAAAGAAAGAAAGAAAGAAAGAAAGAAAGAAAGAAAGAAAGAAAGAAAGAGAAAGAAAGAAAGAAAGAAAGAGAAAGAAAGAAAGAAAGAAAGAAAGAAAGAAAGAAAGAAAGAAAGAAAGAAAGAAAGAAAGAGAAAGAAAGGAAGGAAGGAAGGAAGGAAGAAAGGGAGGGAGGGGGAAGGAAGGGGAAGGAAGGAAGGAAAAAAAGAAAC | 195608525 |
| 1290 | AAGAAAAAAGGAAGAAAGAGAGGGAGGAAGGGAGAGAGGGAAGGAGAGAAGAGAGAAT | 195691862 |
| 1291 | AGGAAGAAAGAGAGAGAGAAGAAAGAAAGAGAGAAGAAGGAAAGAGGAAGGAAGGAAGGC | 195866866 |
| 1292 | AAAAAAAAAAAAGAAAGAAAGAAAGGAAGAAAGGAAGGGAGGAAGGAAGGAAAGAAGAAAGGAAGGAAGGAAAAC | 196054173 |
| 1293 | AGGGAAAGAAAGAAGGAAAGGAGGGAGGGAGGGAGGGAGGAAGGAAGGAAGGAAGGAAGGAAGGAAGGAAGGAAGGAAGGAAGGAAGGGGGGAAGGAAGGAAGGAAAAGAAAGC | 196660802 |
| 1294 | AGAAGGAAGGAAAGAAAGAAAGAAAGAGAGAAAGAAAGAAAGAAAGAAAGAGAAAGAAAGC | 196660983 |
| 1295 | AAAGGAAGAAAGGAAGGAAGGGAGGGAGGGAAGGAGGGGGAGAGAGAGAGAGAGAGAC | 196965664 |
| 1296 | GGAGAGAGAGAGAGAGAGAAAGAAAGAAAAAGGAAGGAAGGAAGGAAGGAAGGAAGGGAGGGAGGGAGGGAGGGAGGGAGGGAGGGAGGGAGGGAGGGAAAGAAAGAAAAAGAAAC | 196965722 |
| 1297 | AAAAAAAAAAAAAAAAAAAAAAAAAAGAAAGAAAGAAAGAAAGAAAGAAAGAAAAT | 197103757 |
| 1298 | AGAGAGAGAGAGAGAGAGAGAGAGAGAGAGAGAGAGAGAGAGAGAGAGAGAT | 197180166 |
| 1299 | AAAGAAAGGAAGGGAAAGAGAGAGAGAGAAGAAGAAAAAGAAAGAGAGAGGAAGGAAGGAAGGAGGGAAGGAAGGAAGC | 197476002 |
| 1300 | AAGAAGGAAAGAAGGAAGGAAGGGAGGGAGAGAGGGAGAAGGGGGAAGAAAAAAAGGAGGAGGGAGAAAGAAGGAAGGGAGT | 197551723 |
| 1301 | AAAAAAAGAAAGAGAGAGAGAGGAAGAGGAAGAGGAAGAAGAGGAAGAGGAAGAAGAGGAAGGAGAAAAAC | 197725647 |
| 1302 | AAAAAAAAAAAAAAAAAAAAAAGGAGAAGAAAAGAAAAGAGAAAAAAAGAAAAGAGC | 197816862 |
| 1303 | GGAGAAGGAAAGAAAGAGAAAGAGGAAGGAAGGAAGAAGAGGAAGGAAGGAAGGAAGAAGGAAGGAAAGAAGGAAGGAAGGGAGGGAGGGAGGGAC | 197968133 |
| 1304 | GAAGAGGAAGAGGAAGAAGAGGAAGAGGAGGAGGAAGAGGAGGAGAAAGAAGAAGAGGAAGAGGAC | 198010203 |
| 1305 | GAGGAGGAGGAAGAAGAAGAAGAGGAGGAAGAAGAAGAAGAGGAAGAGGAGGAAGAAGAGGAAGAGGAGGAAGAAGAGGAAGAAGAGGAAGGAGGAGAAGGAGAT | 198010269 |
| 1306 | GAAGAGGAAGAGGAAGAAGAAGAAGAAAGAAGAAGAAAAGAAGAAGAAAAGAGAAGAAGAAGAAGGGAGAAGAGAGAAGGGAGAAGGAGAAGGAGGAAGGAGGAAGGGGAAGAAGGAAGAAGGAAGAC | 198010374 |
| 1307 | GAAAGAAGGAGGAGAAGGAGAAGGAGAAGGAGAAGAAGAAGAAGAAGAAGAAT | 198010517 |
| 1308 | AGGGGAAGAAGGAGGAGGAGGAGGAGGGAGGAGGAGGAGGAGGAGGGGAGAAGGAAAAGAAGGAGAAGAAAGAGAAGAAGGAGGAGGAGGAGGAAGGGGGGAGGAGGAGGAGAAGGAGAAGAAGAAGAAGAAGAAGT | 198217359 |
| 1309 | AGAAGGAGGAGAAGAAGGAAGAGAAGGAGGAGGAGAAGGAGAAGAGGAGGAAGAAAGC | 198217496 |
| 1310 | AAGAAAAAGAAAAGAAGGAGAAGAGGAAGAAGAAAGAAAGAGAGAAAAGAAAAGAAAT | 198304237 |
| 1311 | AAAAAAAAAAAAAAAAAAAAGAAAAGAAAAGAAAAGAAAGAAGGGGGAGAGAGAGAGAGAGAGAGAGAGAGAGAGAAAGAAAAAAGT | 198312947 |
| 1312 | AGGAGAGAAAGAGAGAGAGAGAGAGAGAGAGAGAGAGAGAGAGAGAGAGAAT | 198526513 |
| 1313 | AAAGAAAGAAAGAAAAAGAAAGAAAGAAAGAAAGAAAGAAAGAAAGAAAGAAAGAAAGAAAGAAAGAAAGAAAGAAAGAGAGAGC | 198879738 |
| 1314 | AAAGAAAAGAAAGAGAGAGAGAAGAGGGAGGGAGGGGGAGAGAGAGAAAAAGT | 199087457 |
| 1315 | AAAAAAGAAAGAAAGAAGGAAGGAAGGAAGGAAGGAAGGAAGGAAGGAAGGAAGGAAGGAAGGAAGGAAGGAAGGAAT | 199300635 |
| 1316 | AAGAAAAGAAAGGAAAGAGGAAGGAAGGAAGGAAGGAAAAAAGGAGAGAAAGAGGGAGAGAGAAAGAGGGAGAGAGAAAGAGGAAGAGAAAGAC | 199339372 |
| 1317 | GAAGAGGAAAAAAAGAAGAAGAAAGAAGAAGAAAGGAGAAGGAGAAAGGAGGAGAAGGAGGAAGGGGAGGAAGAGGGAGAGGGAGAGGGAAAGGC | 199618318 |
| 1318 | AAGGAAGGAAGGGAGGGAGGGAGGAAGGAAGGAAGGAAGGAAGAAGGAAGGAAGGAAGGAAGGAAGGAAGGAAGGAAGAAGGAAGGAAGGAAGGAAGGAAGGAAGGAAGGAAAAAT | 199626462 |
| 1319 | GGAAAAGAAGGAAAGAAGGAAGGAAGAAAGAGGAAGAAAAGAAGGGAAAAAGAAAGAAAT | 199833211 |
| 1320 | AAAAAAGAGAGAAAGAAAAGAAAAGGAAGGAAGGAAGGAAGGGAAAGAAAGAGAGAGAGAGAGAGAAAGAAAGAAAGAAAGAAAGAAAGAAAGAAAGAAAGAAAGAAAGAAAGAAAGAAAGAAAGAAAGAGGGAAGGAAGGAAGGAAGGAAGGAAGGAAGGAAGGAAGGAAGGAAGGAAGGAAGGAAGGAAGGAAGGAAGGAAGGAAAGGAAGGAAGGAAGGAAGGGAGAAAGAGAGAGAGAAAGAGAGAAGGAAGGAAGGAAGGGAAGGAAGGGAAGGAAGGGAAGGAAGGGAAGGAAGGAAAGGAAT | 200184817 |
| 1321 | GAAGAAGGAAGGAAGGAAGGAAGGAAGGAAGGAAGGAAGGAAGGAAGGAAGGAAGGAAGGAAAT | 200304538 |
| 1322 | AAAAAAAAAAAAGAAAGGAAGGAAAAGAAAAGAAAGGAGAGGAGAGAGGAGGGGAGGGGAGGGGAAGGGAAAGGGT | 200342009 |
| 1323 | AAAAGGAAGAAGGAAGGGAAGAAGGGAAGAAGGAAAGAAAGGAAGAAGGGAAGGGGAAGGGGAAAAGGAAGGGGAAAGGAAGGGAAGGGAAGAAGGAAAT | 200394327 |
| 1324 | AAAAAAAAAAAAAAAGAAAGAAAGGAAAGAAAAGAAGAAAGAAAGAGAGAGAGAGAAAGAAAGAAAAAGGAAGGAAGGAAGGAAGGAAGGAAGGAAGGAAGGAAGGAAGGAAGGAAGGAAGGAAGGAAAGAAAGAAAGAAAGAAAGAAAGAAAGAAAGAAAGAAAGAAAGAAAGAAAGAAAGAAAGAAAGAAAGAAAGAAAGAAAAT | 200473200 |
| 1325 | AAAAAAAAAAAAAAAAAAAAAAAGGAAAAGAAAAGAAAAGGAAAAAAAAGAGT | 200483409 |
| 1326 | AAGAAAAGAAAGAGAGAGAGAGAAAGAAAAAGAAAGAGAGAGAGAGAGAGAGAGAGAAAGAAAGAAAGAAAGAAAGAAAGAAAGAAAGAAAGAAAGAAAGAAAGAAAGAAAAGAAAAGAAGAAGAGAAT | 200563450 |
| 1327 | GGAGGGAGGGAGGGAGGAAGGAAGGAAGGAAGGAGGGAAGGAAGGAAGGAAGGGAAAAGAAAAAAC | 200623281 |
| 1328 | AAAAAGAAAGGAAAGAAAAGGAAGAAAGGAAGAAAGAAAGGAAGAAAGGAAGAAAC | 200637466 |
| 1329 | GGAAAGGAAAGGAAGGAAGGAAGGAAGGAAGAGAGGAGGAGAGGGAGGGAAGGAGGGAGGGAAGAAAGAGAGAAGGAAT | 200637522 |
| 1330 | AGAAAAAAGAAAGAGAGAGAGAGAGAGAAAGAAAGAAAGAAAAGAAAGAAAGAAAGAAAAGAAAGAAAGAAAGAAT | 200637874 |
| 1331 | AAGAAAGAAAGAAGAAAGAAAAGAGAAAGAAGGAAAGAAGGAAAGGAAGGAAGGAAGGAAGGAAGGAAGGAAGGAGAGGGGGAAGGGAGGGGAGGGGC | 200647062 |
| 1332 | GAGGGGAGGAGGGGAGGGGAGAGGAGAGGAGAGGAAAGGAGAGGGAGAAAAAGAGAGAGAGAGAGAGAAAGAGAGAGAGAGAGAAGAT | 200647160 |
| 1333 | AGGAAGAAGAAGAAGAAGAAGAAGAAGAAGAAGAAGAAGAAGAAGAAAAAAAAAAT | 200752635 |
| 1334 | AAAAAAGAGAGAAAGAAGGAGAAAGAAAGAAGAAGAAAGAAAGAAGAAGAAAGAAAGAAAGAAAGAAAGAAAGAAAGAAAGAAAGAAAGAAAGAAAGAAAGAAAGAAAGAGAAAGAAAGAAAGAAAGAAAGAAAGAAAGAAAGAAAGAAAGAAAGAAGGAAGGAAGGAAGGAAGGAAGGAAGGGAAGAAAGAAAGGAAAGAAAAGAAAGAAAGAAAGAAAGAAAGAAAGAAAGAAAGAAAGAAAGAGAAAGAGGAAGGAAAGAAGGAAGGAAGGAAGGAAAGAAGGAAGGAAGGAAGGAAGGAAT | 200907086 |
| 1335 | AAAAGAAAGAAAGAAAGAGAGAAAGAAAGAAAGAAAGAAAGAAGAAAGAAGAAAGAAAGAAAGAAAGAAAGAAAGAAAGAAAGAAAGAAAGAAAGAAAGAAAGAAAGAAAGAAAGAAC | 200907739 |
| 1336 | AAGGAAGGAAGGAGAGAGAGAAGAGAGAGGAAGGAAGAAAGGAAGGAAGGAAAGAAAAAAGGAAGGAAGGAAGGAT | 200907906 |
| 1337 | AAGGAAGGGAAGGGAAGGGGAGAGGAGGAGAGGGGAGGAGAGGGGAGAGAGAGAAAGAAT | 200908083 |
| 1338 | AAAAAAAAAAAAAAAAAGAAAAGAAAGAAAAAGAAAAGAAAAAAAAGAAAGAAAAGAAAAAAAT | 200958081 |
| 1339 | AAAAAAGGAAAGAAAGAAAGGAAAAAGAAAGAAAGGAGAAAGGGAAAGGAAAGGAGAAGAAAAGAAAAAGAGAGAAC | 201056087 |
| 1340 | GGGGGGGGGGGGGGGGGGGGGGGGGGGAGGGAGAGAGAGAGAGAGAGGGAGAGAGAGAGAGAGAGAGAAC | 201110231 |
| 1341 | AAAAAAAAAAGAAAAGAAAAAGAAAGAAAGAAAAAGAAAAGAAAAAAAAGAAAT | 201126485 |
| 1342 | AAGAGAGAAAGAAAAAAGAAAGGAAGGAAAGAAAGAAAGGAAGGAAAGAAAGAAAGAAAGAAAGAAAGAAAGAAAGAAAGAAAGAAAGAAAGAAAGGAAAGAAAGAAGGAAAGAAAGAAGGAAAGAAAGAAAGAGAAGGAAGGAAAGAAAGAAAGAAAGAAAGAAAGAGAAGGAAGGAAGGAAAGAAAGAAAGAAAAAGAAAAAAGAAAGAAAGAAAGGAGGGAAGGAAGGAAGGAGGAAGAAAGC | 201127489 |
| 1343 | GAGAGAAAGAAGAAAGAAAGAAAGAAAGAAAGAGAAAGAGAGAAAGAGAAAGAAGAGAGAAAGGAAGGGAAGGGAGAGAGGAAGGAAGGAAGGAAGGAAGGAAGGAAGGAAAGAAAAAAGGAAGGAAGAAAAGAAAGAGAAGAGAGAGAAAGGAAGGAAAGT | 201127758 |
| 1344 | AGAAAGAAAGGAAAGAAGGAAGGAAGGAAGGAAAGAAAGAGAAAGAGAGAGAAAGAAAGAAAGAAGAAAAGT | 201344754 |
| 1345 | AAAAAAAAAAAAAAAAAAAAAAAAAAAAGAAAGAAAAAAAAGAAAAGAGAAAAGAAAT | 201505280 |
| 1346 | AAAAAAAAAAAAAAAAAAAGGAAGGGAAGGGGAGGGGAGGGGAGAGGAGGAAAGGGGAGGGGAGGGGAC | 202055141 |
| 1347 | AAGGAAGGAAAGGAGAGAGGAAGGAAGGAAGGAAGGAAGGAAGGAAGGAAGGAAGGAAGGAAGGAAGGAAGGAAGGAAGGAAGGAAAGAAAGGAGGGAGGGGGGGAGAGGGAGGGAGGGAGGAGAGT | 202139085 |
| 1348 | AAAAAAGAAAGAAAGAAGAAAGGAAAGAAGGAGGGAGGGAGGGAGGAAGAAAGGAAGGAAGGAAGGC | 202186455 |
| 1349 | AGAAAAAAAAAAAAAAGAAGAAGAAAGAAAGAGAAAGGAAAGAAAGAGAAAGAAAGAAAGAAAGAAAGAGAGAGAGAGAGAAAT | 202227037 |
| 1350 | AAAAAAGAAAAAAAGAAGAAGAAGAGGAAGAAGAAGAAGAAGAGGAGGAGGAGGAGGAGGAAGAGGAGGAGGAGGAGGAAGAAGGAGAAGGAGAAGAAGAAGAAGAAGAAGAAGAAGAAGAAGAT | 202356767 |
| 1351 | AAAAAAAAGAAGAAAGAGAAGAAGAGGAGGAGGAGGAGGAAGAAGAGAAAGAGGAGGAAGAGGAAGAAGAT | 202377780 |
| 1352 | GAGAGGAAAGAAAGAAAGAGAGAGAGAGAGGAAGAAAAAGAAAGAAAGAAAC | 202400280 |
| 1353 | AAGAAAGAAGAAAAAGAGAGAGAGAGAGAGAGAGGGAGGGAGAGAGAGAGAGAGAGAGAGAGAAAGAAAGAAAAGGAAAGAAAGGAAAGAAAAAGAAAAAGGAAAGAAAAGAAAGAAAAAGAAAAGAAAGAAAGAGAAAAGAAAGAAAAAGAAAAGAAAGAGAGAGGC | 202546228 |
| 1354 | AAGGGAAGGAGGGAGGGAGAGAGGGAGGGAGGGAGGGAAGGAAGGAAGGAAGGAAGGAAGC | 202546703 |
| 1355 | AAGGAAGGAGGGAGGGAGGGAAGGAAGGAAGGAAGGAGGGAGGGAGGGAAGGAAGGAAGGAAAAGAAAGAAGAAAGAAAGGAAGAAT | 202546908 |
| 1356 | AAAGAAAGAAAGAGAGAAAGAAAGAAAAAAAAGAAAGAAAGAAAGAAAGAAAGAAAGAAAGAAAGAAAGAAAGAAAGAAAGAAAGAAAGAAAGAAAAGAAAGAAAT | 202546995 |
| 1357 | AGGAGAGGGAGAGAGAGAGAGAGAGAGAGAGAGAGAGAGAGAGAGAGAGAGAAT | 202569532 |
| 1358 | AAAAAAGAAAGAAAGAAAAAGAAGAGAGAGAAAGGAAAGAAAAGAAAAGAAGAGAAAGAAAGAGGC | 202577104 |
| 1359 | AAAAAAAAAAAAAGAAAGAAAGAAAAAGAAAGGAAAGAAAGAAAGAGAGAGAGAGAGAGAGGAAGAAAGAAAGAAAAAAAAGAAAGAAAGAAAGAAAGAAAGAAAGAAAGAAAGAAAGGGAGAGAGAGAGAAGGAAGGAAGAAAGGAAAGAGAAAGAAAGAAAGAAAGGAAAGAAAGAAAGAAAAGAAAAAAAAAAAAAAAAGAAAAC | 202650208 |
| 1360 | AAAGAGGGGAGAGAGAGAGGAGAAGGAGGGGGGAGAGAGAGAGAGAAGGGGC | 202830734 |
| 1361 | AGAAAAAAAAAGGAAGGAAGGGGAAAGAAAAGAGAAAGGAGAAAAAAGAAAT | 203017113 |
| 1362 | AGAGAGAGAGAGAGAGAGAGAGAGAGAGAGAGAGAGAGAGAGAGAGAGAGAGAGAGAGAC | 203263093 |
| 1363 | GAGGGGGAGAGGGAGAGGGAGAGGGAGAGGGAGAGGGAGAGGGAGAGGGGGGGAGAGAGAGAGAGAGAGAGAGAGAGAGAGAGAGAGAGAGAGAGAGAGAGAGAT | 203314039 |
| 1364 | AAAGGGAAAAGGAAAGGAGAAAGGAAAAGGGGAAAGGAAAGGAGGAAAGGGAAGGGGAAAGGGGAAT | 203411907 |
| 1365 | AGAGAGAGAGAGAGAGAAGAAGAAGAAGGAAAGGAGGAAGAAGAAGAAAGAAAAAGGAT | 203866344 |
| 1366 | GGGAGGGAAGGGGAAGAAAGGGAAGGGGAGGGAAGGGGAAGGAAGGGGAGGGAAGGGGAAGGAAGGGGAGGGAAGGGGAAGGAAGGGGAGGGAAGAAAAGAAC | 204043691 |
| 1367 | AAAGAAGAAGAAGAAGAAGAAGAAGAAGAAGAAGAAGAAGAAGAAGAAGAAGAAGAAGAAGAAGAAGAAGAGGAGGAAGAGGAAGAGGAAGAGGAAGAAGAAGAAAGAAGGAAGAAGAAGAAC | 204184266 |
| 1368 | GGAGGGAGGGAGGGAGGGAGGGAAGGAGGGAAGGAAGGAAGGAGAGAGAGAAGGAGGAAGGGAGGGAGGAAGGAAGGAGAGAGAGAGAGGAAGGAAGGAAAGAGAGAAAGGGAGGGAGGGAGAGAGGGAGGGAGGAAGGAAGGAAGGAGAGAGAGAAGGAGGAAGGGAGGGAGGAAGGAAGGAGAGAGAGAGAGGAAGGAAGGAAAGAGAGAAAGGGAGGGAGGGAGAGAGGGAGGGAGGAAGGAAGGAAGGAAGGAAGGAAAGAAAGAAAGAAAGAAGGAAGGAAGGAGAGAAAAGC | 204206177 |
| 1369 | GAAAAAAAAGAAGGAAGGAAGAAGGAAGGAAGGAAGGAAAGGGAGGAGGGAAGGGAGGGAGGAAGGAAGAGAGGAAGGAAGGAC | 204246446 |
| 1370 | GGAAAAAAGGAAGAGAAAAAGAAAGAAAGAGAAAGAAAGAGAGAAAGAAAGAAAGAAAGAAGGAAGGAAGGAAGGAAGGAAGGAAAGAAAGAAAGAAAGAAAGAAAGAAAGAAAGAAAGAAAGAAAGAAAGAAAGAAAGAAAGAAAGAGGGAGGGAGGAAGGAAGGAAGGT | 204246530 |
| 1371 | GGAAGGAAGGAAGGGGAAAGAAAGGGAGAAAGGAAGGGAAGGGAAGGGAAGGGGAGGGAAAAGGAGGGAAGGGGAGGGAAAAAGGAGGGAAGGGAAGAGGGAGGGAAGGGAAGAGGGAGGGAAT | 204246728 |
| 1372 | AAGAAAGAAAAAGAAAGAAAAGAAAGAAAAGAAAGGAAGAAAGAAAGAAAAAAAAGAAAAGAAAAGAAGAT | 204309610 |
| 1373 | AAAAGAGAAAAAAAGAAAAGAAAGAAGGAAGGAAGGAAGGAAGGAAGGAAGGAAGGAAGGAAGGAAGGAAGGAAGGAAGGAC | 204376284 |
| 1374 | GGAAGGAAGGAAGGAAGGAAAGAAAAGAAAAGAAAGAGAAAGAAAGAGAGAAAGGAAAGAAGAAAGAGAGAAAGGAAGGAAGGAAGGAAGGAAGGAAGGAAGGAAGAAAGAAAGAAAGAAAGAAAGAAAGAAAGAAAGAAAGAAAGAAAGAAAGAAAGAAAGAAAGAAAGAGAAAGAAAGAAAC | 204376366 |
| 1375 | AAAAAAAAAGAAAGAAAGAAAGAAAGAAAGAAAGAAAGAAAGAAAGAAAGAAGGAAAGAAAGAAAAGAAAAGAAAGAAAGT | 204391750 |
| 1376 | AAAAAGAAAGAAAGAAAGAAAGAGAGAGAGAGAGAGAGAGAAAGGAAGGAAGGAAGGAAGGAAGGAAGGAAGGAAGGAAGGAAGGAAGGAAGGAAGGAAGAAAGAAAGAAAGAAAGAAAGAAAGAAAGAAAGAAAGAAAGAAAGAAAGAAAGAAAGAGAT | 204392772 |
| 1377 | AAGAAAAAAGGAAGGGAAGGGGAGGGGAGGGGAGGGGGAGGAAGGAAGGAAGGAGGGAGGGAGGGAAGGAGGGAAGGAAGGAAGGAGAGAGAGAGAGAAAGAAAAGGAAAGAAAGAGAAAAAGAGAGAGGAAAGAAAGAAAGAAAAAGGAAGAAAGAAAGAGAAAGAAAGAAAGAGAAGGAAAGAAAAAGGAAAGGAAGGAAGGAAGAGAGAGAAAAAGGAAGAAGT | 204559978 |
| 1378 | GAAGGAAGGGAGAGAAAAAAGAAAGGAAAGGAAGAAAGGAAGGAAGAGAGAGAAAGAAGAAAGAAAGAAAAGAAAAAGAAAGGAAGAAAGGAAGGAAGGAAGAGAGAGAAAGAAAGAAAGT | 204560205 |
| 1379 | AAGGAAGGAAGAAAGAAAGGAAGGAAGGAGGAAGGGAGGGAGGGAAGGAAGGAAGGAAAGAAAGAAGGAAGAAAC | 204634944 |
| 1380 | AGAAAGAAAGAAAGAAAGAAAGAAAGAAAGAAAGAAAGAAAGAAAGAAAGAAAGAAAGAAAGAAAGAT | 204635019 |
| 1381 | AAGAGAAAGAGAGAGAGAGAGAGAGAAAGAAAGAAGGAAAGAGAGAGAGAGAAGGAAGGAAGGAAGGAAGGAAGGAAGGAAGGAAGGAAGAAGGAAGGAAGGGAGGGAGGGAGAAAGGAAT | 204666895 |
| 1382 | GAAGAAGGAAGGAAGGAGGGAGAAAGGAAGGAAGGAGGAAGGAAGGAGGAAGGAAGGAAGGAAAGAAGGAAGGAAGGAAAT | 204667020 |
| 1383 | GGAAGGAAAGAAAAGAAGAAAGAGGGAGGGAGGGAGGGAAGGAAGGAAGGAAAC | 205039010 |
| 1384 | AAAGAGAAAAAAAGGAAAGAAAAGGAAGGAAAGGAAAGGAAAAGAAGAGAAAGGAAGAAT | 205039064 |
| 1385 | GGAAGGAAGGGAGGGAAAGGAAGGAAGGAAGGAAAGAAGGGAGGGAGGGAGGGAAAGAAAGAAGGAAAGAAAAGGAAGAAGGAAAGGAAAGGAAGGGAGGGAGGGAGAGAGGGAAAGAAAGAAAAAAAGGGAAGAAGGAAGAAGGAAGAAGAAAGGGAGGGAGGGAGGGAAAAGGAAGGAAGGAAGAAAGGAAAGAAAGAAGAAAAGGAAGAAT | 205039142 |
| 1386 | GGGGGGAGGGGAGAGGGAGGGGAGAGAGAAGGAAGGAAGGGAGGGAAAAGAAAGAAAGAAC | 205039406 |
| 1387 | GAAAGAAAAAAAAAGAAGAAAGGAAGGAAGGAAAGAAGGAAGGAAGGAAAGAGAGGT | 205128197 |
| 1388 | GAAGAGAGAGAGAGAGAGGAGAGAGAGAGAGAGAGAGAGAGAGAGAGGGAGAGAGAGAGAGAGGGAGAGAGAGAGAGAGAGAGAGAGAGAGAAAGAAAGAAAGAGGGC | 205315401 |
| 1389 | AGAAAGAAAAAGAAAGGGAAAGAAGGAAGGAAGGAAGGGAGGGAAGGAGGGAGGGAGGGAGGGAAGGAAC | 205427116 |
| 1390 | AAAAAAAAAAAAAAAAGAAAGAAGGAAAAGAAAGAAAAGGAAGGAAGGAAGGAT | 205486206 |
| 1391 | GGAAAGAAGAAAGGGAAAGGAAAGGAGGAAGAAAGAGAGAGAGAAAGAGAGAAAGAAAGAAAGAGAAAGAAAAGAAAGAAAGAAAAAGAAAGAAAC | 205486260 |
| 1392 | AAAAAAAAAAAAAGAAGAAGAAAGAAAAGAAAAGAAAAAGAAAGAAAGAAAGAAAGAAAGAAAAGAAAAGAAAAC | 205685988 |
| 1393 | GGGAAGGGGAGAGGGGAAAGGGAGAAGGGGAAGGGAGAGGGGGAAGGGAGAAGGGGAAGGGAAGGGAAGGGGAAGGGGAAGGGGAAGGGGC | 205824423 |
| 1394 | AGGGAAGGGGAGGGGAGGGGAGGGGAGGGGAGGGGAGGGGAGGGGAAGGGAGGGGGC | 205825900 |
| 1395 | AAAAAAAAAAAAGAAGAAGAAAGAAAGAAAGAAAGAAAGAAAGAAGGAAGGGAAAGAAAGAAAGAGAGAGAGAGAAAGAAAGAAAGGAAGAGAAAGAAAGAAGGAAAGAAAGAGGC | 205905774 |
| 1396 | AGAGAAAGAAAGAAGGAAAGAAAGGAAGGAAGGAAAGAAAAGAAAAGGAGGGAGGGAGAGAGGAAGGAAGGAAGGGAAAGAAGGAAGGAAGGC | 206033316 |
| 1397 | GAAGGAAAAAGAAGAAAGAAAAAAGAAAGAAAGGAAGGAGGGAGGGAGGGAAAGAAAGGAAGGAAGGAAAAAGAAAGAAGAAAGAGAGAGAGAAAGAAGGAAAGAAGGAAAGGAAGGAAGGAAGGAAGAAAGAAAGAAAGGAAGGAAGGAGGGAGGGAGGGAAAGGAAGGAAGGAAGGAAGGAAAAAGAAAGAAGAAAGAAAGAGAGGGC | 206033437 |
| 1398 | AGGAAGGAAAGAAGGAAAGAAGGAAGGAAGGAAGGAAAAGAGAAAGAAAGGAAAGAAAGAAAGAAAGAAAAGAAAGAAAGAAAGAAAGAAAGAAAAAGGAAGAAAGAAAGAAAGAGAAAGAAAGAAAAGAAAAGAAAGAC | 206033962 |
| 1399 | GAAAGAAAGGAAGGAAGGAAGGAAAGAGGGAGGGAGGGAAGGAAGGAAGGAGAAAGC | 206094786 |
| 1400 | AAAAAAAAAAAAAAAAAAAAAGGAAGAAGAAGGAGAAGAAGAAGGAGGAAGAGGAGGAGAAGGAGAAGGAGC | 206107364 |
| 1401 | AGGAGAGGGAGGAGAAGAAGGAGAAGAAGAAGAAGGAGGAGGAGGAGAAGGAGAAGAAGAAGGAGGAGGAGAAGGAGAAGGAGAGGGAGAAGAAGGAGAAGAAGGAGAAGAAGGAGGAGGAGGAGGAGGAGGAGGAGGAGGAGGAGAAGAC | 206107436 |
| 1402 | AAAAAAAAAAAAGAAGAAGAAGAAGAAGGAGGAGAAGGAGGAGGAGGAGGAGGAAGAGGAAGAGGAAGAGGAAGAAT | 206162276 |
| 1403 | AAAAAAAAGAAGGAAGGAAGGAAGGAAGGAAGGAAGGAAGGAAGGAAGGAAGGAGAAAGAAAGAAAGAAAGAAAGAGAAAGAAAGAAAGAAAAGAGAAAT | 206173276 |
| 1404 | AAAAAGAAAAGAAAGAAAAGAAAGAAAGAAAGAGAGAGAAAGAGAGAGAGAGAAAGAGAGAAAGAAAGAGAAAT | 206713870 |
| 1405 | AGAGAGAGAGGGGGGAGAGAGAGAGAGAGAAAGAAAAGGAAGGAAGGAAGGAAAGAAAGAAAGGAGGAT | 207064923 |
| 1406 | AAGAAGAAGAGGAAGAAAGAAGAAAAAGAAGAGGAAGAAAGAAGAAGAAGAAGAAGAAGT | 207111939 |
| 1407 | AGGAGAAGGAGAAGAAGGAGAAGAAGAAGAAGAAAGGAGAAGAAGAAAGAAGAAGAAAGAAGAAGAAGAAAGAAGAAAGT | 207111999 |
| 1408 | AAAAAAAAAAAAAAAAAAAAAAAAGAAAAAAAAAGAAAAAAAAGAGAGAGAGGAGGAAGT | 207264728 |
| 1409 | AAAAAAAAAAAAAAAGAGAGAGAGAAAGAAAGGAAGGAAGGAAGGAAGAAAC | 207369663 |
| 1410 | AAAAAAAAAAAAAAAAGAAAAGAAAAGAAAGAAAAAAAGAAAGAAAGAAAAGAAAC | 207442789 |
| 1411 | AAAAGAAAAGAAAAGAAAAGAAAAGAAAAGAAAAGAAAAGAAAAGAAAAGAAAAGGAAAGAAAAGAAAAGGAAAGGAAAGAAAAGAAAAGAAAAGAAAAGAAAAGAAAAGAAAAT | 207670426 |
| 1412 | AAGAAGAAGAAGAAGAAGAAGAAGAAGAAGAAGAAGAAGAAGAAGAAGAAGAAGAAGAAGAAGAAGAAGAAGAAAAAGAAAAAAAGAAGAAGC | 207890697 |
| 1413 | AAAAAAAAAAAAAAAAAAAAGAAGGGGAGGGGAGGGGAGGGGAGGGGAGAGGAGGGGAAGGAAAGGGAAGGGAAGGGAAGGGAGGGAAAAGAC | 207909856 |
| 1414 | AAAAAAGAAAAAGAAAGAAAGAAAGAAAGAAAGAAAGAAAGAAAGAAAGAAAGAAAGAAAGAAAGAAAGAAAGAGAGAAAGAAGGAAAGAAAGAAAGAAAAAGAAAT | 207912702 |
| 1415 | AAGAAGAAGAAGAAGAAGAAGAAGAAGAAGAAGAAGAAGAAGAAGAAGAAGAAGAAGAAGAAGAAGAAGAAGAAAAAGAAAAAAAGAAGAAGC | 208047560 |
| 1416 | AAAAAAAAAAAAAAAAAAAAGAAGGGGAGGGGAGGGGAGGGGAGGGGAGAGGAGGGGAAGGAAAGGGAAGGGAAGGGAAGGGAGGGAAAAGAC | 208066719 |
| 1417 | AAAAAAGAAAAAGAAAGAAAGAAAGAAAGAAAGAAAGAAAGAAAGAAAGAAAGAAAGAAAGAAAGAAAGAAAGAGAGAAAGAAGGAAAGAAAGAAAGAAAAAGAAAT | 208069565 |
| 1418 | AGAGAGAGAGAGAGAGAGAGAGAGAGAGAGAGAGAGAGAGAGAGAGAGAGAT | 208257789 |
| 1419 | GGAAGAAAAAGAGGGAAAGAAAGAAAGAGAAAGGAAGGAAGAAAAGAAGAAAAGGAGT | 209305560 |
| 1420 | AAAAAAAAAAAGGAAAAGAAAGAGAGAGAGAAAGAAGAAAGAAAGAAAGAAAAAGAAAGAAAGGAAAAGAAAGAAAGAAAGAAAGAAAGAAAGAAAGAAGGAAGGAAAGAAAGAGAAAGAAAGGAGGAGAAAAC | 209484785 |
| 1421 | AAAGAGAGAGAGAAAGAAAGAAAGAAAGAAAAAGAAAGAAAGGAAAAGAAAGAAAGAAGGAAAGAAAGAGAAAGAAAGAAAGGAGGAGAAAAC | 209484957 |
| 1422 | AGAAAAGAAGAAAGGAAGGGAAGAAGGAAGAAAAGAAGGAAAGGAGGAAGGC | 209508480 |
| 1423 | AAAAAAAAAAAAAAAAAAAAAAAAAAAGAAAAAGAAAAGAGAAGAAAGAAAGAAAAC | 209534880 |
| 1424 | GGAAGAAAAAAAAGGAAGAAAGAAAAGGAAGGGAAAAAGAAAGGAAAGGAGGGAGGGAC | 209783581 |
| 1425 | AAAAGAAGAAGGAAAGAAGGAAGGAGGGAAGGAAAAGGGAAGGGAGGAAAGGC | 210196734 |
| 1426 | AAAGAGAAAGAAAGAGAGAGAGGAAGGAAGGAAGGAAGGAAGGAAGGAAGGAAGGAAGGAAGGAAGGAAGGAAGGAAAAAAAGGGAAGGAAGGAAGGAAGGAAAAAAGAAGGAAAAGAAGGAAGGAAGGAAGGT | 210265037 |
| 1427 | AGGAGAGAGAGAGAGAGAGAGAGAGAGAGAGAGAGAGGGAGAGAGAGAGAGAGGAAGGAAGGAAGGAAGGAAGGAAGGAAGGAAGGAAGGAAGGAAGGAAGGAAGGAAGGAAGAAAT | 210405353 |
| 1428 | AAAAAAAAAAAAAAAAAAGAAGAAGAAAGAAAAGAAAGAAAAAGAGAAAAGGT | 210505131 |
| 1429 | GGAAGGAAGAAAGGGAGGGAGGGAGGGAGGAAGGGAGGGAGGAAGGAAAGAGAGAAAGAGT | 210521004 |
| 1430 | AGAAAGGGAGGAAGAAAGGAGGGAGAGAGAGAGGAAGGGAGGAGGAGAAGGGAAGGAAGGAGGAGAC | 210806738 |
| 1431 | AGAGGGAGGAAGGAAGGAAAAGAAGGAAGGAAAAGAAGGAAGGAAGGAAGGAGAGAGAAAGGAGAAAGAAAGAAAAGAAAGAAAGAAAGAAAAAAGAAAAAGAAAGAGAAAGAAAAAGAAAGAAAAAAAGAAAGC | 210847860 |
| 1432 | AAAAAAAAAAAAAGAAGGAAGGAAGGAAGGAAGGAAAGAAAGAAAGGAAGGAAGGAAGGAAAGAAAGAAAGAAAGAAAGAAAGAAAGAAAGAAAGAAAGAAAGAAAGAAAGAAAGAAAGAAAGAAAGAGAAAGAAAGAAGGAAAGAAGGAAAGAAAGAAAGAAAGAAAAAAGAAAGAC | 210857093 |
| 1433 | AAAAGAAGAAAGAGAGAAGGAAGGAAGGAAGGAAGGAAGGAAGGAAGGAAGGAAGGAAT | 210924606 |
| 1434 | AAAGAGAGAAAAAGAAAGAGAAAGAAAGAAAGAAGAGAGAGAGAGAGAGAGAAAGAGAGAGAGAGAGAGAGAGAGAAAT | 211349863 |
| 1435 | AGAGAGAGAGAGAGAGAGAGAGAGAAAGAAAGAAAGAAAGAAAGAAAGAAAGAAAGAAAGAAAGAAAGAAAGAAAAGAAAAGAAAGAGAAAC | 211557381 |
| 1436 | AAAGGAAAGAAGGAGGGAAAGAAGAAGAGAGGGAGGGAGAAAGGGAGAGAGT | 211855951 |
| 1437 | AAGAAAAGAAAGAAAGAAGGAAGGAAGGAAGGAAGGAAGGAAGGAAGGAAGGAAGGAAGGAAAGAAGAAGAAGAAAAAAGAAAAGAAAAGAAAAAAAGAAT | 212340335 |
| 1438 | AAAAAAGGGAAGGGGAGGGGAGGGGAAGAGAGGGGAGGGGAGGAGAAAGAAAAGAAGGGAAAAGAAAAGAAGAGAAGAGAAAAGAAAAGAAAT | 212441883 |
| 1439 | GAAAAGAGAGAGAGAGAGAGAAAAGAAGAAAGAAAGAAAGAAAGAAAGAAAGAC | 212558974 |
| 1440 | GGAAGGAAGGAAGGAAAGAAAGGAAGGAAGGAAGAAAGGAAGAAAGAAAGAAGGAAGGAAAGAAAGAAGAAC | 212559073 |
| 1441 | AAGAAAGAAAGGAAGAAGGAAAGAAAGAAGAAAAGAAAGAAAGAGAGAGAAAGAAAAAGAAAGAAAGAAAGAAAAAGAAAAAGAAAGAAAGAAAGAC | 212559145 |
| 1442 | GGGGAGAGGGAGAGGGAGAGGGAGAGGAGGGAGAGGGAGAGGGAGAGGAGGGAGAGGGAGAGGAGGGAGAGGGAGAGC | 212962392 |
| 1443 | GAGAGAAAGAGAGGAGAGAGAGAGAGAGAGAGAGAGAGAGAGAGAGAGAGAGAT | 213175612 |
| 1444 | AGAGAGAGAGAGAGAGAGAGAGAGAGAGAGGGAGAGAGGGAGGGAGGGAGGAAGGGAGGAAGGGAGGGAGGGAGGAAGGAAGGGAGGAAGGAAGAAGAAGGAAGGAAGGAAGGAAGC | 213189621 |
| 1445 | AGAGAGAGAGAGAGAAAGAAAAAGAAAAGAAAAGAAAGAGAGAAAGAAAAAGAGAGAGAGAAAGAAGAAAAGAAAGAAAGAAGGAAGGAAGGAAGGAAGGGAGGGAGGT | 213279890 |
| 1446 | GAAAGAAGGAAGGGAAGGGAAGGGGAGGGGAGGGGAGGGGAGGGGAGGGGAAT | 213315873 |
| 1447 | AAAAAGAGGAAGAAGAAAGAGAGAAAGAAAAAAAAAGAAAGAAAGAAAGAAAGAAAGAAAGAAAGGAAGGAAGGAAGGAAGGAAGGAAGGAAGGAAGGAAGGAAGGAGAGAGAAAGAGAAAGAGAGAAAAGAAAAAAAAAAAGAAAAAGC | 213328108 |
| 1448 | AAAAAAAAAAAAAAAAAAAAAAAAAAAAGAAAGAAAGAAAAAAAAAAGAAAC | 213405017 |
| 1449 | GAAAAAGAGAGAGAAGAGAAGAGAAGAGAAGAGAAGAGAAGAAAGAGAAAGAGAAAGAAAGAT | 213464695 |
| 1450 | GAGAGAGAGAGAGAGAAGGAAAGAAAGAGAGAGAGAAAGAAAGAAAAGAAAGAAAGAGGC | 213495954 |
| 1451 | GAAAGAAAGAAAGAAAGAAAGAAAGAAAGAAAGAAAGAAAGAAAGAGAAAGAAAGGAAGGGAGAAAGAGAGAGAC | 213565896 |
| 1452 | AGAAAGAAAGAGAAAGAAAGAAAGAGAAAGAAAGAGAGAAAGAGAGAGAAAGAGAAAGAAAGAGAAAGAAAAGGAAGGAAGGAAGGAGGAAGGAAAGAAGGAAGGAAGGAGGAAGGAAGGAAGGAAGGAAGGAC | 213565971 |
| 1453 | GGGAGAAAGGGAGAGGGAAAGGGGAGGAGGAAAAGAGGAGAAAAGGAAGAAAGAGGGAAAGGGAGAGAGAGGGAGGGAAGGAAGGAAGGAT | 214402192 |
| 1454 | AAGAAGGAAGGAAGGAGGAAGGAAGAAAGGAAGGAAGGGAGGGAGGGAGGGGAGAGGT | 214545254 |
| 1455 | GAGAGAGAGAAAGAGAGAGAGAGGAAAGAAGAAAGAAAGAAAAAGAAAGAAAGAAAAAGAGAAAGAAAT | 214798950 |
| 1456 | AAAAAAAAGGAAAGAGAGAGAGAAAGAAAGAAGGGAAGGAAGGAAGGAGGGAGGGAGGGAAGAAAGGAAGGAAGGAAGGGAGGGAGGGAGGGAAGAAAGAAAGAAAGAAAGAAGAAAAGAT | 214799019 |
| 1457 | AGAAAAGAAAGAAGGAAGGAGGAAAGGAGAGGAGGAAGGGAAGAAGAAAAGGAAAGAGGAAAAGGAAAT | 214960215 |
| 1458 | AAGGAAGGAAGGAAGGAAGGAAGGAAGGAAGGAAGGAAGGAAGGAAGGGAGGGAGGGAAGGAAGGAAC | 215042805 |
| 1459 | GGAAGGGAAAGGAAAGGAAAGGAAGGGAAGGGAGGGGAGGGGAGGGGAGGGGAGGGAAGGGGAGGGGAGGGAAGGGGAAGGAAGGGAAGGAAAGAAAGGAAGGAAAGAAAGAAAGAAAAGAAAGAAAGGAAGGAAGGAAGGGAAGGAAAGGAAGGAAGGAAGGGAAGGAAAGGAAAGAAAGGAAGAAAGGAAGAAAGGAAGGAAGGAAGAAAGAAAGAAAGAAAGAAAAAC | 215131003 |
| 1460 | AGAGAGAGAGAGAGAGAGAGAGAGGGAGAGGGAGAGGGAGAGAGAGAGAGAGAGAGAGAGAGAGAGAGAGAGAGAGAGAGAGAC | 215351772 |
| 1461 | AAAAAAAAAAAAAAAAAAAAAAAAAGAAGAAGAAGAAGAAGAAAGAAGAAAAAAAAAAGT | 215981171 |
| 1462 | AAAAAAAAAAAAAAAAAAAAAAAAAGAAGAAGAAGAAGAAGAAAGAAGAAAAAAAAAAGT | 216138034 |
| 1463 | AAAAAAAGAAAAGAAAAGAAAAGAGAAAGAAAGAAAGAAGGAAAGAAAGAGAAAGAAGGAAGGAAGGAAGGC | 216194021 |
| 1464 | AGGAAGGAAAGAAAGAAAAGAAAGAAAGAAAAGAAAGAAAGAAAGAAAGAAAGAAAGAAAAGAAAGAAAGAAAGAAAGAAAGAAAGAAAGAAAT | 216194093 |
| 1465 | GAAGGAAAGGAAGAAAAGAAAGAAAGAGAAAGAAAGAAAGAGAGAGAAAGAGAAAGAGAGT | 216694732 |
| 1466 | AAAAAGAAAGAAAGAAAGAAAGAAAGAAAGAAAGAAAGAAAGAAAGAAAGAAAGAAAGAAAGAAAGAAAGAAGGAAAGAAAC | 217152342 |
| 1467 | AGGGGAGGGGGGAAGAGAGAAGAAAGAGAAAGAAAAAAAAAAGAAAGAAAGAAAGAGAAGGAAAGAAAT | 217174745 |
| 1468 | AAAAAGAAAGAAAGAAAGAAAGAAAGAAAGAAAGAAAGAAAGAAAGAAAGAAAGAAAGAAAGAAGGAAAGAAAGAAAGGAAGGAAGGAAGAAAGAAAGAGAAAGAAAGAGAAAGAAAGAGAAAAAAGAAAGAGAAGGAGAGAGGAAC | 217174842 |
| 1469 | GAAGGAAAGGAAGGAAGGAAGGAAGGAAGGAAGGAAGGAAGGAAGGGAGGGAGGGAGGGAGGGAGGGAGGGGGAGGGAGGGGGAGGGAGGGAGGGAGGAAAGGGAAGGAAAGGGAGAGAAAAT | 217272711 |
| 1470 | AAGAGGAAAGGAAAGGAAAGGGAAAAGGAAAGGGAAAAAGGAAAGAAAGGGAAAGGAAAGGGGGAAGGGAGGAT | 217303480 |
| 1471 | AGAGAGAAAGGAAGGAAGGAAGGAAGGAAGGAAGGAAGAAGGAAGGAAGGAAGGAAGGAAAGAAGGAAAGAAGAGT | 217366111 |
| 1472 | GGAAGAGGAGGGAAGGGAAGGGAAGGGAAGGGAAGGGAAGGGAAAGGAAAGGAAAGGAAAGGAAAGGAAAGGAAAGGAAAGGAAAGGAAAGGAAAGGAAAGGAAAGGAGGGAGGAAGGAAAGAAGGAAGGAAGGAAAGAAGGAAGGAAAAGAAGAGC | 217366187 |
| 1473 | AAAAGAAGAGAAGAGAAGAGAAGAGAAGAGAAGAGAAGAGAAGAGAAGAGAAGAGAAGAGAAGAGAAGAGAAAT | 217455882 |
| 1474 | AAAAAAAAAGAAAAGAAAAGAAGGAAGGAAGGAAAGAAAGAAGGAAGGAAGGAAGGAGGGAAAGAAGGC | 217469043 |
| 1475 | AGGGGGAGAGGGAGAGAAAGAGGGAGAGGAAGGGAGGAAGGAAAGAAGAGAAAGAGAT | 217628561 |
| 1476 | AGGAGAGAAGGGAGGGAAGGGAAGGGAAGGGAAGGGAAGGGAAGGGAAGGGAAGGGAAGGGAAGGGAAGGGAAGGGAAGGGGAGT | 217670904 |
| 1477 | GGGGGAAGAGGGGAGGGGAGGGGAGGGAGGGAGGGGGAAGGAGGGAGGGAGGGAGT | 217670989 |
| 1478 | AAAAAAAGAAGAAAAGAAAAGAAAAGAAAAGAAAAGAAAAGAAAAGAAAAGAAAAGAAAAGAAAAGAAAAGAAAAGAAAAGAAAAGGAGAGGAGAGGAGAGGAGAGGAGAGGAGAGGAGAGGAGAGGAGAGGAGAGGAGAGGAGAGGAGAGGAGAGGAGAGGAGAGGGGAGGGGAGGAGAGGGGAGGAGAGGAAAGGAGGGAAGGAGGGAAGGAC | 217709136 |
| 1479 | AAAAAAAAAAAAAAGAAAGAAAGAAAAAAAAAAAAGAAAGAAAGAAAAGAAAAGAAAGAAAC | 217799337 |
| 1480 | AAAAAAAAAGAAAGAGAAAGAAAGAAAGAAAGAAAGAAGGAAAGAAAGAAAGAAAGAAAGAAAGAAAGAAAGAAAGAAAGAGAAT | 217985195 |
| 1481 | AAAAAAAGAAAGAAAGAAAGAAAGAAAGAAAGAAAGAAAGAAAGAAAGAAAGAAAGAAAGAAAGAAAGAAAGAAAGAAAGAAAGAAAAGAAAC | 218120803 |
| 1482 | GGGGAGAGGGGAGAGGGGAGAGGGGAGAGGGGGGAGGGGGGAGGGGGGAGGGGGGAGGGGGGAGGGGGAGGGGGGAGAGGGGAGAGGGGAGAGGGAGAT | 218321884 |
| 1483 | AAAAGAAGAAAGAAAGAAAAGGGAGGAAGGAAGGAAGGAAGGAAAGAAAGAAAGAAGGAAGGAAGGAAGGAAAGAAGGAAGGAAGGAAGGAAGGAAGGGAGGT | 218368964 |
| 1484 | AAAGGAAAAGAGAAGAGAAGAGAAGAGAAGAGAAGAGAAGAGAAGAGAAGAGAAGAGAAGAGAAGAGAAGAGAAAGGC | 218802133 |
| 1485 | AAGAAGAAAGAAAAAGAGAGAGAGAGAGAGAGAAAGGAGAGAGAGAGAAAGAGAAAGGGAAAAGAAAGGAAAGGAAGAAGGAAGGAAGGAAAGAGAGAAGAAAGAAAGAAAAAGAAAGAAAAAAAGAAAGAAAAGAAAGAGAAAGAAAGAAC | 219002694 |
| 1486 | AAAAAAAAAAAAAAAAAGAAAAAGAGAGAGAGAAAGAAAAAGAAAGAAAGAAAGAGAGAGAGAGAGAAAGAAAAAGAAAGAAAGAAAGAAAGAAAGAAAGAAAGAGAAAGAAAGAAAGAAGAAAAGAAAAGAAAAGAAAAAAGAAAAGAAAGGT | 219060756 |
| 1487 | GAGGGAAAAGAAGAGAAGAAAGAAAAAGAAGAGAAGAAAGAAAAAGAAGAGAAGAAAAAGAC | 219623482 |
| 1488 | AGAGAAAGGAAGAAAGGAAAGGAAGGAGGGAGGGAGGGAGGAAAGAAGGGAGAGAAGGAGGAAGGAAGGAAGAAAGGAAGGAAGGAAAGAAGGGAGGGAGGGAAGGAGAGAGGGAGGGAAGAAGGAAGGGAGGGAAGAAAGAAGGAAGGAAGAAGAGAGAGC | 219847399 |
| 1489 | AGGAGAAAGGAAGAAAGAAGAGGGGAGGAAAGGAGAGGAGAGAGAAGGGAAGGGGAGAAAGGGGAGGGGAAAAC | 219847602 |
| 1490 | AGAGAAGGAGGAGAGAGGGAGGGAGGGAAAGAGGGAGAGAGAGAAGAGAGAAAAGAGAAAT | 220201106 |
| 1491 | AAAAAGAAAGAAAGAAAGAAAGAAAGAAAGAAAGAAAGAAAGAAAGAAAGAAAGAAAGAAAGAAAGAAAGAAAGAAAGAAAGAAAGAAAGAAAGAAAGAAAGAAAGAAAGAAAGAAAGAAAGAAAGAAAGAAAGAAAGAAAGAGAAAC | 220272568 |
| 1492 | AGAAAAAGAAAGAGAGAAAGAAAGAAAGAAAGAAAGAAAGAAAAGAAAAAGAAAGAGGGAAAGAAAGAAAT | 220272716 |
| 1493 | AAAGGAAGGAAAGGAAAGGAAAAAGGAAAAGGAAAGGAAAGAAAAAGAAAAGAAAAAAGAAAC | 220367182 |
| 1494 | GAGAAGAGAAAGAAAAGAAAAGAAAAGAAGAAAGGAAGGAAGGAAGGAAGAAAGGC | 220523281 |
| 1495 | AAAGAAAGAGAGAAAGAAGGAAGGAAGGAAGGAAGGAAGGAAGGAAGGAAGGAAGGAAGGAAGGAAGGAAGGAAGAAAGGGAGGAGGGAGGGAGGGAGGGAGGGAAAGAAAGGGAAGGAGGAAGAAAGGAAAGAAAGGAAGGAAGGAAAAAAGGAAAGGAGGGAGGGT | 220556051 |
| 1496 | AAAGAAAGAAAGGGAAAGAGAGAAAGAAAAGAAAAGAAAGAGAGAAAGGAAGGAAAGAGAGAGGGAGAGAGAGAGGGAGGAGGGAGGGAGAGAGAGGAAGGAAGGGAGGAAGGAAGGGAGGGAGAC | 220556341 |
| 1497 | AAAAAAAAAAAAAAGAAAGAAAAAAAAAAGAAAGAGAAAGAGAGGGAAAGAGAGAAAGGAGAGAGAGAGGGAGGGAGGGAGGAAGGAAGGAAGGGAGGGAGGAGGGAGGGAGGGAGGGAGGGAGAGAGGAT | 220596115 |
| 1498 | GAAAAAAAAAAAAAAAAAAAAAAAAGAAGGAGGAGGAAGGAGGAGGAAGAAGAAGAAGAAAAAAGAAGAAAGAAGAAGAGT | 220886137 |
| 1499 | AAAAGAAAGAAAGAAGGAAGGAAGAAAAAGAAAAGAAAAGAAAAAAAAAGAAAAGAGAGAGAGAGAGGC | 221245950 |
| 1500 | GAGAGAGAGAGAGAGGGAGAGAGAGAGAGAGAGAGAAAGAGAGAGAGAGAGGGAAT | 221853032 |
| 1501 | AAAAAGAAAGAGAAGGAAGGAAAGAAAGAAAGAAGGAAGGAAGGAAAGAAAGGAAAAGAGAAGAAAAGAAAAGAAAAT | 221943295 |
| 1502 | AAAGAGAAGAAGGAGGAGGAGGAGGAGAAGGAGGAGGAGGAGGAGGAGGAGGAGGAGAAAAAAAGT | 221979851 |
| 1503 | AGGGAGGAAGGAAGGAGAAAGAAGGGAGGGAGGAAAAGGAAGGGAAGAAAGGAGGAGAGAAAGAAGGGAGGGAGGAAGAGAAAGGAAGGGAAGAAAGAAGGAAAAAAAGGGAGGGAGGGAAGGAAGGAAT | 222033004 |
| 1504 | GAAAGAAAAAAAGAAAGAAGAAGAAGAAAAAGAAAGAAAGAGAAAGAAAGAAAGAAAAAGAAAGAAAAAAC | 222198285 |
| 1505 | AAAGAAAGAAAGAAAGAAAGAAAGAAAGAAAGAAAGAAAGAAAGAAAGAGAGAGAGAGAGAGAGAGAGAGAGAGAGAGAGAGAGAGAGAGAGAGAGAGAGAGAGAGAAAGAAAGAAAGAAAGAAAGAAAGAAAGAAAGAAAGAAAGAAAGAGAAAAGGAAC | 222321223 |
| 1506 | AAAAAAAAAAAAAAAGAAAGAAAGAAAAAGAAAGAGGAAGAAAGAAAAGAAAGAAAGAAAGAAAGAAAAGAAAGAAAGAAAGAAAGAAAGAAAGAAAGAAAGAAAAGAAAGAAAGAAAGAAAGAAAGAAAGAAAGAAAGAAAGAAAGAAAGAAC | 222323808 |
| 1507 | AAAAAAAAAAAAAAAAAAGAAAGAAAGAAAGAAAAGAAAGAAAAGAAAAGAAAAGAAAAAGAAC | 222361615 |
| 1508 | GAAAAGAAAAGAAAAGAAAAGAAAAGAAAAGAAAAGAAAAGAAAAGAAAAGAAAAGAAAAGAAAAAGAGAAT | 222440116 |
| 1509 | GGAAGGGAGAGGGAAGGGAAGGGAGAGGGAAGGGAAGGGAGAGGGAAGGGAAGGGAGAGGGAAGGGAAGAGGGAGGGAAGGAAAGAGGGAGGGAAGGAAAAGGAAGGGAGGGGAAC | 222497156 |
| 1510 | GAAGGAAAGAAAGAAAGAAGAAAGAAAGAGAAAGAAAGAAAAGAAAGAAAGAAAGAAAGAAAGGAAGAAAGAAAGAAAGAAGGAAGGAAGGAAGGAAAAAGGAAAAGAAAGAAAGAGAGAGAGAGAAAGAAAGAGAAAGAAAGAAAGAAGGAAAGAAAGAAAGAAAGAAAGAAAGAAAGAAAGAAAGAAAGAAAGAAAGAAAGAAAGAAAGAAAGAAAGAAAAGAGAGGGAGGGAGGGAGGAAGGAAGAAAGAGAGAAAGAAAGAAGGAAAAGT | 222623527 |
| 1511 | GGGAAAGAAAGAAAGAGAGAAGGGAGAAAAGGAAGGAAGGAAGGAAGGGAGGAAGGGAGGGAGGT | 222655433 |
| 1512 | AAAAAAAAAAAAAAAAAAAAAAAAAAAGAAAAGAAAAGAAAAGAAAGAAAGAAAAAAAGAAAAAGAAAAC | 222805768 |
| 1513 | GAAAGAAAGAAGAAAGAAAGAGAGAGAGAGAAAGAAAGAAAGAAAGAAAGAAAGAAAGAAAGAAAGAAAGAAAGAAC | 222836741 |
| 1514 | AGGAGGAGAGGGAGAGAGAGAGAGAAAAGAAGGAAGAGGAGGAGGAAGAGGAGAGGAAAAC | 222965565 |
| 1515 | AAAAAAAAAAAAAAAAGAAGGAAAGAAGGGAAGAAGGGAAGAAGAGAAGAAGGGAAGAAGGGAAGGGAAGGGAAGGGAAGGGAAGGGAAGGGAAGGGAAGGGAAGGGAGAAAGGAAAAAGAAAGT | 222986307 |
| 1516 | AAAAAAAAAAAAAAAAAAAAAAAAAAAAAAAAAAAAAAAAAAAAAAAAAAAGAGGGC | 223258177 |
| 1517 | AAAAAGGAAAAAAGGAAGGAAAGAAAAAAGGAAGGAAGGAGGGAAGGAGGGAAGGAAAGGAAGGAAGGAAAAGAAGGAAAGGAAGGAAGGGAAAGAGAAAGAGGGAAGGAGGAAGGGAGGGAAGGAGGGAGGGAGGGAGGGAGAGAGAGAGGGAGGGAGGGGAAGGGAAGAAAAGGGAGGAGAAGGGAAAGGAGGAAGAAAAGGAAAGGAAAGGAAT | 223305138 |
| 1518 | AAAAAAAAAAAAAAAAAAAAAAGAAAGAAAGAAAAGAAAAAAAAAGAAAAGAAAGT | 223336564 |
| 1519 | AAAAAAAAAAAAAAAAAAAAAAAAAAAAAGGAAGGAAGGAAGGAAGGAAGGAC | 223735770 |
| 1520 | AGAGAGAGAGAGAGAGAGAGAGAGAGAGAGAGAGAAAGAGAGAGAGAGAGAT | 223922243 |
| 1521 | GAAGGGGAAAAGGAGGAAGAGGAAGAAGGGGAGGAAGGGGGAGGAAGAGGAAGAGAAAGAGAAGT | 223969226 |
| 1522 | AGAGAGAGAGAGAGAGAGAGAGAGAGAGAGAGAGAAAGAGAGAGAGAGAGAT | 224079105 |
| 1523 | GAAGGGGAAAAGGAGGAAGAGGAAGAAGGGGAGGAAGGGGGAGGAAGAGGAAGAGAAAGAGAAGT | 224126088 |
| 1524 | AAAAAAAAAAAAAAAAAAAAAAAAAAAAAAAAAAAAAAAAGAAGAAGAAGAAGAAAAGAAAGAAAGAAAGAGAAAAGAAGAAAAAAGAAAAT | 224352932 |
| 1525 | AGGAAAAAAAAAAAAAAAAGAGAAAAGAAGAAAGGAAGGAAGGAAGGAAGGGAGGGAGGGAGGGAGAAAGAGGGAGGGAGGGAGGGAGGAAAT | 224354404 |
| 1526 | AGAAAGGAAGGAAGGAAGGAAGGAGAAAGAAAGAAAGAAAAAGAAAGAAAGAAAGAC | 224382823 |
| 1527 | AGAGGGAGAGAAAGAAGGAAGGAAGGAAGGAAGGAAAGAAGGAAGGAGAAAC | 224382925 |
| 1528 | GGGGAGAAGGGAAAAGAGGAAAAGGAAGAGAAGAGGAGAAGGAGGAGGGGAGAAAGGGT | 224413973 |
| 1529 | AAGAAAGAAAGAAAGAAAGAAAGAAAGAAAGAAAGAAAGAAAGAAAGAAAGAAAGAAAGAAAGAAAGAAAGGAGGGAGGGAGGGAGGGAGGGAGGGAGGGAGGGAGGGAGGGAGGAAGGGAGGGC | 224559169 |
| 1530 | AGAGAGAGAGAGAGAGAGAGAAAGAGAGAGAGAGAGAGAGAGAAAGAGAGAGAAAGGC | 224562342 |
| 1531 | AAAAAAAAAAAAAAAAAAAGAGAGGGAAGGAAGGGAGGGAGGGAGGGAAGAAGGAAGGAAGGAAGGGAGGGAGGGAGGGAGGGAGGGAGGGAGGAAAGAAGGAAAGAAGGAAGGAAGGAAGGAAGGAAGGAAGGAAAGAAGGAGGC | 224588599 |
| 1532 | GAAAGAGAGAGAGAGAGAGAAAGAGAGAAGGAGAGAAGGAGAGGAAGGGAAGAAGGGAGGGAGAAAGAAAGAAAAAAT | 224595376 |
| 1533 | AGAGAAAAAGAGAAGGAAGGAAGGAAAAGAAAGAAAAGAGAAGAGAAAAGAAAAAAAAAGAGAGAAAGAC | 224595454 |
| 1534 | GAGGGGGAGGGAAAGGAGGAGGGGGAGGGAAAGGAGGAGGGGGAGGGAAAGGAGGAGGGGGAGGGAAAGGAGGAGGGGGAGGGAAAGGAGGAGGGGGAGGGAAAGGAGGAGGGGGAGGGAAAGGAGGAGGGGGAGGGAAAGGAGGAGGGGGAGGGAAAGAAAGAAGAAGAAAAAC | 224703839 |
| 1535 | AAAAAGGAAAAAAGGAAGGAAAGAAAAAAGGAAGGAAGGAGGGAAGGAGGGAAAAAGGGAAGGAGGGAAGGAAAGGAT | 224867220 |
| 1536 | GGGAAAGAAGGAAAGGAAGGAAGGGAAAGAGAGAAAGAGGGAAGGAGGAAGGGAGGGAAGGAGGAAGGGAGGGAAGGAGGGAGGGAGGGAAGGAGGGAGGGAGGGAAGGAGGGAGGGAGGGAGAGAGAGAGGGAGGGAGGGGAAGGGAAGAAAAGGGAGGAGAAGGGAAAGGAGGAAGAAAAGGAAAGGAAAGGAAT | 224867298 |
| 1537 | AAAAAAAAAAAAAAAAAAAAAAAAAAAGAGAGAGAGAGAGAAAGAAAGAGGGC | 224875860 |
| 1538 | AAAAAAAAAAAAAAGAAAGAAAAAAAAAGGAAAAAAAAAAAAGAAAAGAAAAT | 224988314 |
| 1539 | GAGAGAGAGAAAGAGAGAGAGAGAGAAAAGAGAGAGAAAGAAAAGAAAGAAT | 225058945 |
| 1540 | AAGAAAGAAAGGAAGGAAGGAAAGAAAAAAAAGAAGGAAGGAAGGAAGAAAGGT | 225058997 |
| 1541 | AAAAAAAAAAAAAAAAAAGAAAAAAAAAAAGAAAAAAGAAAAAAAAAGAGAAAAAC | 225288270 |
| 1542 | AAGAAGAAGAAAAAGGAAGGAAGGAAGGAAGGAAGGAAGGAAGGAAGGAAGGAAGGGAGGGAGGAAGGAAGGGAGGGAGGGAGGGAGGGAGGGAGGGAGGAAGGGGAAAGGAAGGGAAGGAAAGGAAAGGAAAGAAGGAAGAGAAGGGGGAAAT | 226163529 |
| 1543 | AAAAAAAAAAAAAAAAAAGAAAGAAAGAAAAAAGAAAGGAAGGAAGAAAAGAAT | 226353156 |
| 1544 | GGGGAAGGAAGAAAAAGAAGGGAAGGAAGGGAGGAAGGGAGGGAGGGAGGGAGGGAGGGAGGGAGGGAGGGAGGGAGGGAGGGAGGGAGAC | 226407486 |
| 1545 | AAAAAAAAAAAAAAAAAGGAAGAAAAAGAGGAGAAGGAGGGGGAAGAAAAGAGGGGAGAGGAGGGGGAAGGAAAAGGGAAAAGT | 226472840 |
| 1546 | AAGAAAGAAAGAAGGAGAGAGAGAGAGAGAGGGAGGGAGAGAGAGAGAGAGAGAGAAAGAGAAAGAAAGAAAGAAAGAAAGAAAGAAAGAAAGAAAGGAAAGAAAGAAGAAAGAAAGAAAGAGAAAC | 226545708 |
| 1547 | AAAAGAGGAAAAGAGAAAGGAAAGGAGAGGAGAGGAGAGAAGAGAGGAGAAGAGGGGAGGGGAGGGGAGGGC | 226644458 |
| 1548 | GAAAGAGAGAGAGGGAGGGAGGAAGGAAGGGAGGGAGGGAGGGAGGGAGGGAGGGAGGGAAGGAAAGGAAGGC | 226683425 |
| 1549 | AAAAAGAAAAAAAGAAAAGAAGAAGAAGAAGAAAAGAAAGGAAAAGGAAAGAAAGAGAGAGAAAGAAAGGAAGGAAGGAAGGAAGAAAAAAAAAAT | 226894236 |
| 1550 | AAAAAGAAGAAAGAAGGAAGGGAGGGAGAGAGAGAGAGAGAAAGAAGAAGAAAGAAAGAGAAAAGAAAAGAAAAAAGAAAAGAGAAGAGAAAAGAAAAGAAAAGAGGGAGGGAGGAGGAAGGGAGGAAAGGAAAC | 227517336 |
| 1551 | GAAGAGGGGGAGGAGGAAGAGGAGGGAGGAGGGAGGAAGAAGAGGAGAGAGAAGGGAGAGC | 227541531 |
| 1552 | AGGAAGGAAGGAAGGAAGGAAGGAAGGAAGGAAGGAAGGAAGGAAGGAAGGT | 227731762 |
| 1553 | GAGAGAGAGAGAGAGAGAGAGAGAGAGAGAGAGAGAGAGAGAGAGAGAGAGAGAGAGT | 227831290 |
| 1554 | AAGAAAGGAAGGAAGGAAGGAAGGAAGGAAGGAAGGAAGGAAGGAAGGAAGGAAGGAAGGT | 228013058 |
| 1555 | AAGGAAGGAAGGGAAGGAGGAAGGAAGAAAGGGAAGGAAGGAAAGAAGGGAAGGAAGGGAGGAAAGGGAT | 228252213 |
| 1556 | GGGGAAAGAGAGAGAGGAGAGAGGGAGAAAGAGAGAGAGGAGAGAGAGAGAGAGAGAC | 228320401 |
| 1557 | GAGAGAAGGAGGAGAAAGAAAAGAAAGAAAAGAAAGGAAGAAAGAAAGAGAAGAAAGAAAAGAAAGAAAAGAAAGAAAAGAAAGGAAGAAAGAAAGAGAAGAAAGAAAAGAAAGAAAAGAAAGGAAAGAAAGAAAGAAAGAGAGAGAGAAAGAAAGAAAGAAAGAAAGAAAGAAAGAAAGAAAGAAAAAGAGAAAGAAAGAAAGAGAAAGAGGAGGGGAGGGGAGGGGGGAGGGGAGAAAAAGAGAGGC | 228323921 |
| 1558 | AAAGAAAGAAAAGAAGGAAGGAAGGAAGAGAGGAAAAGAAAGAAAGGAAAAAT | 228467943 |
| 1559 | GAGAAAGAAGGAAAGAGGAAAAAAGAAAAAAGGAAGAAGGGAGGGGGAAAAGAAGAGAAAGAGAGGAAGAAAT | 228795931 |
| 1560 | GAGGAGGGGAGGGAGAAGGGAAGAAGGAAGGGAGGAGGGGAGGAAGGAGGGAAGGAGGGGAGGGAGGAGGGAAGGAGGGGAGGAAGGAGGGGAGGGAGT | 228796155 |
| 1561 | AAGGGAGGAAGGAGGGGAGGAGGGAAGGAAGGAGGGGAGGAGGGAAGGAAGGAGGGGAGGAAAGGGGAGAAAGGAAGGAAGGGAGGAGGGGAGGGAGGAGGC | 228796290 |
| 1562 | AGGAGGGGAGGGAGGAGGGAAGGAGGGGAGGAAGAGGGGAGGAAGGAAGGAAGGAGGGAAGGAAGGAGGGGAGGAGGAGGC | 228796392 |
| 1563 | AGGAGGGGAGGGAGGAGGGAAGGAAGAAGGGGAGGAAGGAGGGGAGAAAGGAGGGAAGGAAT | 228796473 |
| 1564 | AGAAAGAAGGAAGGAAGGAAGGAAGGAGGGAAGGAGGGAGGGAGGGAGGGAAGGAAGGAAGGAGGGGGAGGT | 228964604 |
| 1565 | GAAGAAAAGAAGGAAAAAAGAAGAGAGGGAAGGAAGGAAAAGAAAGAAAGAAGGAAGT | 229355322 |
| 1566 | AAAAGAAAGAGAAAAAGGGGGAGAGAGAGAGAAGGAGAAAGAGAAGAAGGAT | 229601841 |
| 1567 | AGGGAAGGAGAGGAGAGGGGAGGGGAGGGGAGGGGAGGGGAGGGGAGGGGAGGGGAGGAGAGGAGAGGAGAGGAGAGGAGAGGAGAGGAGAGGAGAGGAGGAT | 229862825 |
| 1568 | AGAGAAGGAAGGAAGGAAGGAAGGAAGGAAGGAAGGAAGGAAGGAAGGAAGGAAGGAAC | 229896159 |
| 1569 | GAAGGAAAGAAGGAAGGGAGGAAGGAAAAAAGGGAGGAAGGAAGGAAGGAAAGAAGGAAGGAAGGAAAAAAAGT | 229896218 |
| 1570 | GAGGAGAAGAGGAGGAGGAGGAGGAAGAAGAGAAGAGAAGGAGGAGGAGGAAGAAGAGAAGAGGAGGAGGAGGAAGAAGAGAAGAGGAGGAGGAGGAAGAAGAGAAGAGGAGGAGGAGGAAGAAGAGAAGAGAAGGAGGAGGAAGAGGAGGAGGGGAT | 230260413 |
| 1571 | AAAGAAAGAAAGAAAGAAGGAAAAAAAGAAAGAAAGAAAGAAAGAAAGAGAAAGAAGGGAAGGAAGGAAGGAGAAGGGGAGGGAAGGGAGGGC | 230646797 |
| 1572 | AGGGGAGGGGAGGGAAGGGAAGGGGGAAGGAGAGAGAGAGAAGGAAAAAGAAGAAGAAAGAAAGAAGGAAAGAAAGAAAAAGAAAGAAAGAGAAAGAAAAGAAAGAAAGAAAC | 230646890 |
| 1573 | AAGGAAGGAAGGAAGGAAGGAAAGAAAGAAAGGGAAGGGAAGGAAGGAAGAGAGGGAAAAGAAGGAAT | 230647053 |
| 1574 | AGAGAGGGAGAGGGAGGGAGAGAGGGAGAGGGAGGGGGAGAGGGAGAGGGAC | 230767230 |
| 1575 | GAAGAGAGAAGAAGGGAGGAAGGGAGGAAAAGAGGGAGGGAAGAAGGAAAAGAGAT | 230786229 |
| 1576 | AAAAAAAGAAAAAGAAAGAAGAAGGAAGGAAGGAAGGAAGGAAGGAAGGAAGGAAGGAAAGAAAGAAAGAAAGAAAGAAAGAAAGAAAGAAAGAAAGAAGGAAGGAAGGAAAGAAGGAAAGAGAAAGAAAAGAGAGAGAGAAAGAGAGAGAGAAAGAAAGAGAGAAAGGAAGAAAGAAAGAAGAAAAAGAAAGAAAGAAAGAAAGAAAGAAAGAAAGAAAGAAAGAAAGAAAGAAAAGAAAAGAAAAGAAAAGAAAGAGAAC | 230872814 |
| 1577 | GGGGGAGGGGAAGAGGAGAAGGGAAGAAGAGAGGGAGGAAGAGGAGGGAGGGAGAAAGAGAAGGAAT | 231147856 |
| 1578 | GGAGGAGGAAGAGGAAGGAGGAAAGGGGAAAAAGAAGGGGAAGGGGAAAAAAGAAGGGT | 231227717 |
| 1579 | AGGGGAGGAAGAAGAGGAGGAAGGAAAAAGGGAAGAGAAGGGGAAGGGAGGGGGGGAGGAGGGAGGAGGC | 231227776 |
| 1580 | GAAAGGAAGAAAGGGAGGAAGGAAAGGAAAGGAAGGAAGGAAAAAGGGAGGGAGGGAGGAAAGAAGGAGGAAAGGAAAGAAGGGAAAAAGGAGAGAGGGAGGGAGGAAGAAAGGAAAGAAGGAAGGAAGGAAGGAAGAAAAAGAAAAGGAAGGAAGAGAAGAGAAAGAAT | 231329291 |
| 1581 | AAAAAAAAAAAAAAAAAAAAAAGAAAGAAAAAAAAAAAAAAAGAAAAGAAAAAGC | 231471020 |
| 1582 | GAAAAAGAAAGAGAGAAAGAAAGAGAAAAAGAGAGAAGGAGAGGAAGAGAGAGAGGGAAAGGGAAGGAAGGGAAGGGAGGAAGGGAGGAAGGAAGGAAAGAAGGAAGGAAGGAGAGAAAGAGAAAGAAAGAT | 231586050 |
| 1583 | AAAGAAAGAAAGAAAGAAAGAAAGAGAAAGAAAGAAAGAAAGAAAGAAAGAAAGAAAGAAAGAAAGAAAGAAAGAAAGAAAGAAAGAAAGAAAGAAAGAAGGGT | 231586228 |
| 1584 | AAAAAGGAAAAAAAGGAAGGAAAGAAGGAAGGGAGAGAGGGAGGAAGGGAGGGAGGGAGGAT | 231591592 |
| 1585 | GGAAGGAAGGAAGGAAGGAAGGAAGGAAGGAAGGAAGGAAGGAAGGAAGGAAGGAAAGGAGAAAAT | 231591654 |
| 1586 | AAGAAAGAAAGAAGAAAGAAAGAAAGAAAGAAAGAAAGAAAGAAAGAAAGAAAGAAAGAAAGAAAGAAAGAAAGAAAGAAAGAAAGGAAAAAC | 231634863 |
| 1587 | AGGGAAAGAAAGAAGGAAGAAAGGAAGGAAGGGAGAGAAGGAGGGAAAGAAAAGT | 231860365 |
| 1588 | GAGGGAGGGAGGGAAGGAAGGAAAGAAGGAAAAAGAAAGAAAGAGGGAGAGAGAGAAAGAAAGAAAAC | 231880439 |
| 1589 | AAGAAAGAAAAAGAAAGAAAGAAAGAAAGAAAGAAAGAAAGAAAGAAAGAAAGAAAGAAAGAAAGAAAGAAAGAAAGAAAGAGGGAAAGAAAGAAAGAAAGAAAGAGGGAAAGAAAGAGAGAAGAGGGAGGGAGGGAGGAAGGGAAGGAAGAAAGAAAGAC | 231880507 |
| 1590 | AAAGAAGGAAGGAAAGAGAAAGAGAGAGAGAGAGAGAGAAAAGGAGGGAAGGAAGGAAGGAAGGAAGGAAGGAAGGAAGGAAGGAAGGAAGGAAGGAAGGAAGGAAGGAAGGGAAGGAAGGAAGGAAGGAAAGGAAGGAAGGAAGGAT | 231922559 |
| 1591 | AGGGAAAGAAAGAAGGAAGAAAGGAAGGAAGGGAGAGAAGGAGGGAAAGAAAAGT | 232017228 |
| 1592 | GAGGGAGGGAGGGAAGGAAGGAAAGAAGGAAAAAGAAAGAAAGAGGGAGAGAGAGAAAGAAAGAAAAC | 232037302 |
| 1593 | AAGAAAGAAAAAGAAAGAAAGAAAGAAAGAAAGAAAGAAAGAAAGAAAGAAAGAAAGAAAGAAAGAAAGAAAGAAAGAAAGAGGGAAAGAAAGAAAGAAAGAAAGAGGGAAAGAAAGAGAGAAGAGGGAGGGAGGGAGGAAGGGAAGGAAGAAAGAAAGAC | 232037370 |
| 1594 | AAAGAAGGAAGGAAAGAGAAAGAGAGAGAGAGAGAGAGAAAAGGAGGGAAGGAAGGAAGGAAGGAAGGAAGGAAGGAAGGAAGGAAGGAAGGAAGGAAGGAAGGAAGGAAGGGAAGGAAGGAAGGAAGGAAAGGAAGGAAGGAAGGAT | 232079422 |
| 1595 | AAAGAAAAGAAGAAGAAGAAGAAGAAGGAGAAGGAGAAGGAGAAGGAGAAGAAGGAGAAGAAGAAAGGAAAAGT | 232189331 |
| 1596 | AAAGAAGGAGGGAGGGAAGGAAGGAAGGAAGGAGGGAGAGAGGAGAGAGAGAGAGAGAAC | 233022612 |
| 1597 | AGAGAGAGAGGAAGGAAGGAAGGGAGAAAGAGAGAGAGAAAGAGAGGAAGGAAGGAAGGAGAAAGAAAGAT | 233022701 |
| 1598 | AAAGAAAGAAAAAGAAAGAAAAGAGGAAGGAAGGAAGGAGAGAGGGAAGGAGAGAAAAGGAAAGGAAAAGGAAAAGAGGAAAGAAAAGGAAAGGGAAGGGAGAGGGAAAGGAGAAGGGAAAGGAAGAGAAGGGAAGAAGGGAAGGGAGAAGAGAC | 233022772 |
| 1599 | AAGGGAAAGGAGAAGGGAAAAGGGAAAGGAGAAGGGAAGGGAGAAGGGAAGGGAGC | 233022927 |
| 1600 | AAAAGGGAAGGAAAGAAAGAAAGGAAAGGAAAGGAAAGAAAGAAAAGGAAAGGAAAGAAAGAAAAC | 233022983 |
| 1601 | GAGAGGAAAGAAGGAAGGAAGGGAGGGAAGAAAGGAAGGAAGAAAGAAAAGAGGGAAGAAGGAAAAGAAT | 233138865 |
| 1602 | AGAGAGAGAAAGAAAGAGAAAAGGGAGGGAGGGAGGGAGGGAAGAAGGAAGGAAGGAAGGAAGAAAGGAAAGAAGGAAGAAAGGAAGAAGGAAGGAAGGAAGGAAGAAGAGT | 233170752 |
| 1603 | AAAAAAAAAAAAAAAAAAAAAAAAAAAAAAAAAAAGAAAAGAAAAAAGAAAAGAAAAAAGAAAAGAAAAAAAAAGAAGAAAAGAAAGGGGAC | 233212172 |
| 1604 | AAAAAAAAAAAAAAAAGAAGAAGAAGAAGAAGAGGAGGAGGAAGGAGAAAGGAAGAAAAAGAGAAGAAAC | 233317378 |
| 1605 | GGAAGGAAGGAGAGGGGAAGGAAGGAAAGGAAGGAAAGAAAAGAAAGAAAGAAGGAGGAAAGGAGGAAGAGAT | 233626856 |
| 1606 | GGGGGAGAGAGAGAGGGAGGGAGGAAGGAGGGAGGAAGGAAGGAGGGAGGGAGGAC | 233626929 |
| 1607 | AGAAGGGAGGGAAGGAAAAAGAAAAGAGAGGAAAGGAAGGAGAAGAGGAGGGAAGGAGAGGGAGAGAAGGAGAGAGGGAGGGAAGAAGAGAGGGAGGGAGGAAGGAAAAC | 233626985 |
| 1608 | AGAAAGGGAAAAAGGAAGGGAGAGAGAAAAAAGGAAAAAAGGAAGGAAGGAAAGAGGGAGGGAAGGAAGGAAGGGAGGGAGGGAGGGAAAAAAT | 233666014 |
| 1609 | AAAAAAAAAAAAAGGAAGGGAAAGGGAGGGGAGGGGAGGGGAGGGGAGGGGAGGGGGGGAGGGGAGGGGGGGAGGGGGGGAGGGGAGGGGGGAGGGGGGGAGGGGAGGGGAGGGGGGAGGGGAGGGGAGGGGGGGAGGGGGGGAGGGGGGGAGGGGAGGGGAGGGGGGGAGGGGGGGAGGGGAGGGGAGGGGAGGGGAGGGGGGAGGGGAGGGGAGGGGAGGGAGC | 233716740 |
| 1610 | AGAAAGAAAGAAAGAAAGAAAGAAAGAAAGAAAGAAAGAAAGAAAGAAAGAAAGAAAGAAAT | 233900083 |
| 1611 | AGGAGAAGGAAGGAAGGGAGGGAGGGAGGGAGGGAGGAAGGAAGGAAGGAAGGAAGGGAGGGAGGGAAGGAGGGAAGGAGGGAGGAAGGAAGGAGAAAGGAAGGAAT | 233934005 |
| 1612 | AAAAAAAAAAAAAAAAAAAGGAAGGAAGGAAGGAGAAAGAAAGAGAAAGAAAGAAAGAAAC | 234384395 |
| 1613 | AAAAAAAAAAAAAAAAGAAAAGAAAAGAAAAAGAAAAGAAAAAAAAGAAAAAGAAAT | 234542666 |
| 1614 | AAGAAAGAAAGAAAGAGAGAAAAAGAAAGAAAAGAAAAGAAAGAAAAAAGAAAAAGGAAAGAC | 234649104 |
| 1615 | AAAAAAAAAAAAAAAAAAAAAAAAAAAAGAAAGAAAAAAAGAAAAGAAAAAAGAAAC | 234784133 |
| 1616 | GAAAGAAAGAAAAGAAAAGAAAGAGAAAGAGAGAAAGAAAGAAAGAAAGAAAAGAAAAGAAAAGAAAAGAAAAGGAAGGGAGGGAAGGGAGGAAAAGAAGGGAAGGAAGGGAAGGAAGGGAAGAAGT | 234865737 |
| 1617 | AAGAGGGAGGGGAGAGAGAGAGAGGGAGAGAGGGAGAGAGGGAGAGAGGGAGAGAGAGAGAGAGAGAGAGAGAGAGAAT | 234939685 |
| 1618 | AAGAAAAAAAAAAAAAAAAAGAAAGAAAGAAAAAGAAAAGAAAAAAGAGAGAAAAAAGAAT | 235172841 |
| 1619 | AAAAAAAAGAGGAAAAGAGGAAGAAGAAAGAAGAAGAAGAGGAAGAAGAAGAAGAAGAAGAAGAAGAAGAAGAAGAAGAAGAAGAAGAAGAAGAAGAAGAGGAAGAGGAAGAGGAAGAGGAAGAAGAAAGAAGAAGAAGAAGAAGAGGAAGAGGAAGGAGC | 235317440 |
| 1620 | AAGAAAGAAAGAGAGAGAGAGAGAGAAGGAAGGGAGGGAGGGAGGGAGGGAGGGAGGGAGGGAAGGGAAT | 235374512 |
| 1621 | GGGGAGGAGGGGGAAGAGGGGGGAGGAGGGGGAAGAGGGGGGAGGAGGGGGAAGAT | 235388905 |
| 1622 | GGGGGAAGAGGGGAGGGGGAGGGGGAAGAGGGGAGGAGGGGGGAAGAGGGGAGGAGGT | 235389132 |
| 1623 | GAGGGAAGAGGGGGAGGGGGAGAGGGGGAAGAGGGGGAAGAGAGGGAGGAGGGAGAAGAGGGGGGAGGGGGAAGAGGGGGAGGAGGGGGAGGAGGC | 235389190 |
| 1624 | GGGGGAAGAGGGGGAGGGAGGGGGAGGGGAGGAGGGAGGGGAGGGGGAGGAGGGGGAGGAGAGGAAT | 235389307 |
| 1625 | AGAGAGGGAGGGAAGAAGGAAGGAGAGGGAGGGAGGGAGAGAGAGAGAAAGAAAAAGAAAGAAAGAAAGAAAGAAAGAAAAAAGAAAGAAAGAAAGAAAGAAAGAAGAAAAAGAAAGAGAGAAAGAAAGAAAAAGAAAGAAAGAGAAAAAAGAAAGGGGGAGAGAGAAGGAGGGAGGGAGGGAGAAAGGAAGGAAGGAAGGGAAGGAAAGAAAGAAAGAGAAAGAAAGAGAGGGAGAGT | 235631976 |
| 1626 | GAGGGAGGAAGGAAGGAAGGAAGGGAGGGAGGGAGGAAAAGAAAGAAAGAAAAGAGAAAGAAAGAAAGAAAGAAAGAAAGAAAGAAAGAAAGAAAGAAAGAAAGAAAAAGAAAAAAGGAAT | 235632215 |
| 1627 | GGAAGGAGAAAGAGAAGAAAGGAAAAAGGAAAGGAAGGAAGAAGAGAGGGAAGGAAGGAGAAAAAAAGAAGGAAAGAT | 235671976 |
| 1628 | AAAAAAAAAAAAAAAAAAAGGAAAGGAAGGAAGAAGAAAGAGAAAGAAAGAGAGAAAGAGAAAAAGAAAGAAAAAGAAAGGAAGAAAGAGAGAAAGGAAGAAAGAGAGAAAGAAAGAGAGAGAAAGAAAGAAAGAAAGAAAGAAAGAAAGAAAGAAAGAAAGAAAGAGAGAAAGAAAGAGAGAAAGAAGT | 235732455 |
| 1629 | AAGGAAAGAAAGAAAGAAAAAAGAAGGGAAGGAAGGAAAGAAGGAAGGAAGGAAGGAAGAAAGAAAAGAAAAGAAAAGAAAAGAAAAGAAAAGAAAAGAAAAAAGAAAAGAAAAGAAAGAAAAGAAAAGAAAAGAAAAAAAT | 235775529 |
| 1630 | GGGAAGAAGAAGAAGAAGAAGAAGAAGAAGAAGAAGAAGAAGAAGAAGAAGAAGAAGAAGGAGGAGGAGGAGGAGGAGGAGGAGGAGGAGGAGGAGGAGGAGGAGGAGGAGGAGGAGGAGGGGGGC | 235829575 |
| 1631 | GGGAGGGGGAGGGGAGGGGAGGGGGAGGGAAAGGGGAGGGAAGGAGAGGGGAGAGGAGGGGAGGGGAGGGGAGGGAAGAGGC | 235829739 |
| 1632 | GAGGAAAGGAAAGGAAAGGAAAGGAGGAAAGGAAAGGAAGAAAGAGAGAGAAAGAGAC | 235860463 |
| 1633 | AAGGAAGGAAGGAAGGAAAGGAAGGAAGGAAAGAAAGAAAAGAAAGAAAGAAAAGAAAAGGAT | 235860536 |
| 1634 | AGGAAAAGAAAGAGAGAGAAAAAGAGAAAGAAAGAAGAAAGAAAGAAAGAAAGAAAAAGAAAGAAAGAAAAGAAAGAAAGAAAGAGGC | 236118817 |
| 1635 | GAAAAAAAGAAAGAAAGAGAGAGAGAGAGAGAGAGAGGGAGAGGGAGAGGGAGAGAGAGAGAGAGAGAGAAAGAAAGAAAAGAAAGAAAAAGAAAGAAAGAAGGAGGGAGGGAGGGAAGGAAGGAAGGAAAGAAGAAAGAAAGAAAGAAAGGGAAGGAAAGAAGGAAGGAAGGAAGAGGAAGGC | 236119172 |
| 1636 | AAAAAAAGGAAGGGAAGGGGAGGGGAGAGGAGGGGAGGGGAAGGGAGGGGAGAGGAGGGGAGGGGAGGGGAGGGGGAGGC | 236139932 |
| 1637 | AGGGAAGGGGAGAGGGAGGGAGGGAGGGAGGGAAGGGAGGAAAGGGAGGAAAGGAAAGAAAAT | 236140018 |
| 1638 | AAAAAAAAAAAAAAGGAGGAAGGAAGGAAGGAAGGAAGGAAGGAAGGAAGGAAGGAAGGAAGGGAGGAAGGAGGGAGGGAAGGAAGGGAGAGAGT | 236335156 |
| 1639 | AAAGGGAGGGAGGGGAGGAAGGAAGGAAGGAAGGAAAAAGGGAAAGGGAAGT | 236340643 |
| 1640 | AAAAAAAAAAAAAAAAAAAGAAAGAAAGAAAGAAAAGAAGAGAGAAAAGAAAAGAAAAAAGAAGGGGAGT | 236393160 |
| 1641 | AAAAAAAAAAAAAAAAAAAAAAAAAAAGGAAAAGAAAAAGAAAAAAAAAAAAAAGAAAT | 236692161 |
| 1642 | AGAAGAAGAAGAGGAAGAAGAAGAGGAAGAAGAAGAGAGAAGAAGAAGAAGAAGAGAGAAGAAGAAGAAGAAGAAGAAGAAGAAGAAGAAGAAGAAGAAGAAGAAGAAGAAGAAGAC | 236954365 |
| 1643 | AAAAAAAAAAAAAAAAAAGAAAAAAAAAAAAGAAAAAGAAAAAAGGAAGAAAAAAAAAGAAAT | 236965316 |
| 1644 | GGAAAGAAAGAAAGAAAAAGAGAGAGAGAGAAAGGAAAGGAAAAAAAAGAAAAGAAAAGAAAAAAAGAAAAAGAAAAC | 237096083 |
| 1645 | AAAAAAAAAAAAAAGAAAGAAAGAAAGAAAAAAAAAAGAAAGGAAAGAAAAAGAAAGAAAGAAAAGAAAGAAAGAAAGAAAGAAAAGAAAGAAT | 237111481 |
| 1646 | AGAAAGGAAGAAAGGAAGGGAAGGGGAAAAGAAGGGAAGGGAAGGGAAGGGAAGGGAAGGGAAGGAAGGAAAGGAAGGGAAGGAAGGAAGGGAAAGGAAGAAAGGAAGGGAAGGGAAAAGAAGGGAAGGGAAGGAAGGAAAGGAAGGGAAGGAAGGAAGGGAAGGGAAGGAAGGAAGGAAGGAAAGGAAGGGAAGGAAGGAAAGGAAGGGAAGGAGGGAAGGGAAGGAAGGAAGGAAGAAAGGAAGGAGAAAC | 237136687 |
| 1647 | AGGGAAAGAGAGAGAGAGAGAGAGGAAGGAGGGAGGGAGGGAAGGAGGAAGAAAGGAAGGAAGGAAT | 237211760 |
| 1648 | AAAAAAGGAAGGAAAGAAGGAAGGAAGGAAGGAAGGAGGGAGGGAGGGAGGGAAGGAGGGAAGGAAGGAAGGAAGGAAGGAAAT | 237533513 |
| 1649 | GAAAAAAGAAAGAAGAAAGAAAGAAGAAAGAGAAAGAGAAAGAAAGGAGAGAAAAAGAAAAAAGAAAAGAAAAAC | 237541215 |
| 1650 | GAAAGAAAGAAAAGAAAAGAAGAAAGAAAAGAAAGAGAGAAAGGAAGGAAGGAAGGT | 237541315 |
| 1651 | AAAGAAAGAGAGAGAGAGAGAGAAAGGAAAGAAAGGAAGAAAGGAAGAAAGAGAAAGGAAAGAGAAAGAAAGAGAAAAAGAAAGAAAGAAGGAAAGAAAGAAAGAAAAAAGAAAGAAAAAGAAAGAGAAAGGAGAAAAAGAAT | 237554856 |
| 1652 | AAAAAAAAAAAAAAAAAAAAAAGAAAAGAAAAAAGAAAAAGAAAAAGAAAAAGAAAAAAC | 237976997 |
| 1653 | AGGGAAGAGAAAGAGAAAGAAAGAAGGGAAGAGAGGAGAAAGGAAGAAAGGGGAGGAGAGAGGAGAC | 237986489 |
| 1654 | AGAAAAAAGGAAGAAGGGAGGAAGGAAGGGAAAGAGAAGGGAAGGGAGGGAAAT | 238020275 |
| 1655 | GGGGGAAGAGGGAGGGAAGAAAGAAGGGAAGGAAAGAAAGAAAGAGAAAGAAAGAAAGAAAAAAAGAAAGAAAGGAAGGAAGGAGAGAT | 238022270 |
| 1656 | AAAGAAAGAAAAAGAGAAAGGAAGGAAAAAGGGAAAGGAAGGAAGGAGGGAGGGAGGAAGAAAAGGAAAGC | 238022403 |
| 1657 | AAAAAAAAAAAAAAAAAAAAAAAAAAGAAAGAAAGAAAGAAAGAAAAGAAAAAGAGAAAGAAC | 238195661 |
| 1658 | GAAAGGAAGGAAGGAAGGAAGGAAGGAAGGAAGGAAGGAAGGAAGGAAGAAGGGAAGGAAGGAAGGGAGGGAGGGAGGGAGGGAAAGC | 238531188 |
| 1659 | AAAAAAAAAAAAAAAGAAAAGAAAAGAAAAGAAAAGAAAAGAAAAGAAAAGAAAAAAC | 238915065 |
| 1660 | GGGGGAGGGAGAGAGAGGGAGAGAGAGGGAGAGAGAGGGAGAGAGAGGAAGAGAGAGGAAGAGAGAGGAAGAGAGAGGAAGAGAGGGAGAAAGGC | 238922090 |
| 1661 | AAAGAAAAGAAAAGAGAGAAAGAAGAGAAGAGAGAGAAGAGGAGAGGAAAGGAGAGGAGAGGAGAGAT | 239454131 |
| 1662 | GGAAGGAAGGAAGGAAGGAAGGAAGGAAGGAAGGAAGGAAGGAAGGAAGGAAGGGAGGAAGGGAGGGT | 239459551 |
| 1663 | GGGAGGGAGGGAAAAAGAAGAAAGAAAGAAAAGAGAGAGAGAGAAAGAAAGAAAGAAAGAAAAGAAC | 239459619 |
| 1664 | AGGGAGAAAGAGGAGAGAGAGAGAGAGAGAGAGAGAAAGAAAGAGAGAGAGAGAAAT | 239526856 |
| 1665 | AGGGAAGGGAAGGGGAGGGAAGGGGAGGAAAGAGGAGGGAAGGGGAGGGAAGGGGAGGGAAGGGGAGGGAAGGGGAGGGAAGGGGAGGGAAGGGGAGGGAAGGGAAGGGAAGGGGAGGGGAGGAAAC | 239795512 |
| 1666 | GGGAGGGAGGAAGGAAGGAAGGAAGGAAGGAAGGAAGGAAGGAAGGAAGGAAGGAAGGAAAGAAGGAAGGAAAGAAGGGAGGGAGGGAGAC | 239832898 |
| 1667 | AAAAAAAAAAAAAAAAGAAAAGAAAAAAAAAAGAAAAAGAAAAGAAAGAAAGAAAT | 239948540 |
